# Chemoselective Ru-Catalyzed Oxidative Lactamization *vs* Hydroamination of Alkynylamines: Insights from Experimental and Density Functional Theory Studies

**DOI:** 10.1021/acs.joc.2c02770

**Published:** 2022-12-29

**Authors:** Andrés M. Álvarez-Constantino, Andrea Álvarez-Pérez, Jesús A. Varela, Giuseppe Sciortino, Gregori Ujaque, Carlos Saá

**Affiliations:** † Centro Singular de Investigación en Química Biolóxica e Materiais Moleculares (CiQUS), Departamento de Química Orgánica, Universidade de Santiago de Compostela, 15782 Santiago de Compostela, Spain; ‡ Departament de Química and Centro de Innovación en Química Avanzada (ORFEO-CINQA), Universitat Autònoma de Barcelona, 08193 Cerdanyola del Vallès, Catalonia, Spain

## Abstract

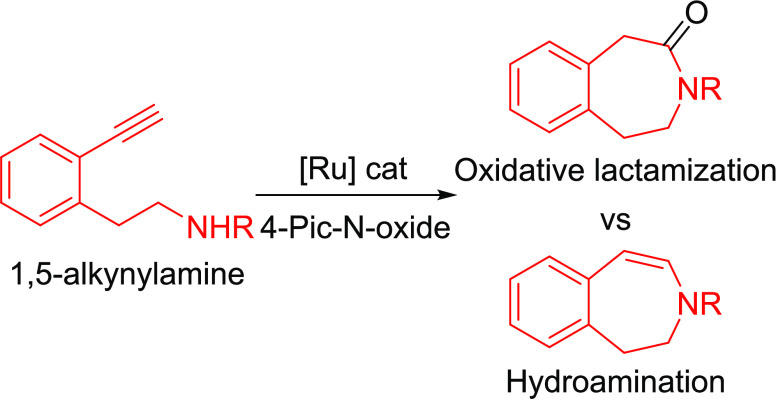

The Ru-catalyzed intramolecular oxidative amidation (lactamization) of aromatic alkynylamines with 4-picoline *N*-oxide as an external oxidant has been developed. This chemoselective process is very efficient to achieve medium-sized ε- and ζ-lactams (seven- and eight-membered rings) but not for the formation of common δ-lactams (six-membered rings). DFT studies unveiled the capital role of the chain length between the amine and the alkyne functionalities: the longer the connector, the more favored the lactamization process *vs* hydroamination.

## Introduction

**Scheme 1:**
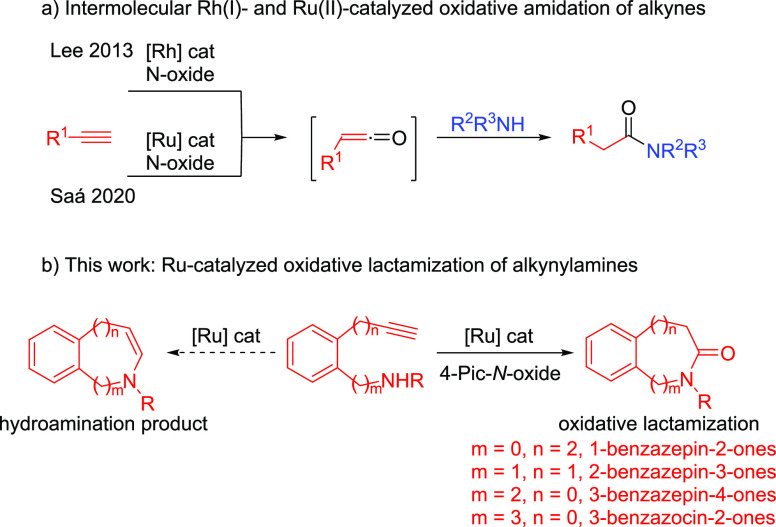
(a) Metal-Catalyzed Oxidative Amidation of Alkynes and (b) Ru-Catalyzed Oxidative Lactamization of Alkynylamines

Amines and their derivatives are present in a myriad of bioactive natural products and pharmaceuticals.^1^ Among all the synthetic strategies to these compounds,^2^ transition metal-catalyzed addition of amines (hydroamination) and amides (hydroamidation) to unsaturated C–C bonds have emerged as powerful sustainable tools to access such functionalities.^3^ In the particular case of alkynes, their electrophilic activation by π-coordination to transition metals make them prone to receive nucleophilic attacks, generating enamines or enamides.^4^ Apart from chemoselectivity, efficient strategies to control the regioselectivity of terminal alkynes have been developed to produce Markovnikov-type products.^3d^ On the contrary, generation of catalytic metal-vinylidenes from the initially coordinated metal-alkyne allows the polarization of the unsaturated bond in such a way that the carbon α to the metal center is now electrophilic affording exclusively the anti-Markovnikov addition products.^5^ In cases where an oxidizing nucleophile is present, the oxygen atom is transferred to the carbon-metal bond of the vinylidene complex, entailing a very reactive ketene intermediate, which could be subsequently trapped by other nucleophiles (e.g., amine) present in the media (Scheme 1a).^6^ A pioneer work by Kim and Lee and Zhang et al. showed that ketenes generated from Rh- and Ru-vinylidenes in the presence of oxidants could be intermolecularly trapped with heteronucleophiles (e.g., amines, alcohols) or be involved in [2 + 2] cycloadditions, respectively.^7^ Recently, our group has successfully developed a general Ru-catalyzed intermolecular oxidative amidation of alkynes^8^ with all types of aliphatic and aromatic amines, which allowed for the synthesis of a variety of primary and secondary amides in good to excellent yields.

More challenging would be the intramolecular processes since the desired intramolecular oxidative amidation (oxidative lactamization) would have to compete with the intramolecular hydroamination reaction (capture of the electrophilic vinylidene).^9^

Herein, we describe the chemoselective Ru-catalyzed oxidative lactamization^10^ of aromatic 1,5- and 1,6-alkynylamines, which provides efficient access to valuable seven-membered 1-, 2-, and 3-benzazepin-2-, 3-, and 4-ones,^11^ privileged scaffolds in natural products and pharmaceuticals,^12^ and eight-membered 4-benzazocin-5-ones, respectively^13^ (Scheme 1b).

## Results and Discussion

We started our investigation by testing the oxidative lactamization of *N*-(2-ethynylphenethyl)propan-1-amine **1a** (a 1,5-alkynylamine) under our previously optimized intermolecular oxidative amidation conditions for secondary amines (Table 1).^8^

**Table 1:**
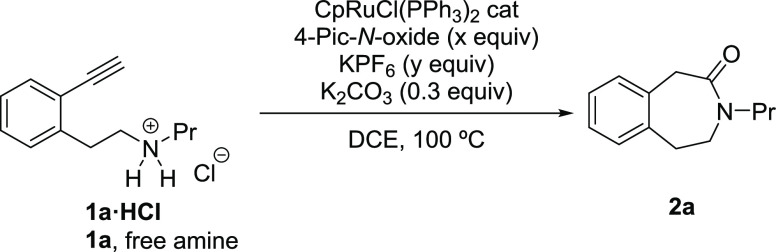
Optimization of the Reaction Conditions^a^

entry	substrate	cat (%)	*N*-oxide (equiv)	KPF_6_ (equiv)	2a (% yield)
1^b^	**1a**	5	2	1	dec
2^b^^c^	**1a**	5	2		dec
3	**1a·HCl**	10	2		30
4	**1a·HCl**	10	2	0.3	50
5	**1a·HCl**	10	2	1	58
6	**1a·HCl**	10	1.1	1	92 (90)^d^
7	**1a·HCl**	3	1.1	1	(97)^d^^e^^f^

a Typical conditions: 0.2 mmol of substrate, 2 mL of DCE, sealed tube, NMR yields.

b Without K_2_CO_3_.

c Similar results were obtained at 70 and 50 °C. At rt, the starting material was recovered.

d Isolated yield.

e 100 °C, 30 min.

f 60 °C, 1 h. dec: decomposition.

To our initial surprise, heating a solution of **1a** in DCE at 100 °C for 4 h in the presence of 5 mol % CpRuCl(PPh_3_)_2_ and 2 equiv of 4-picoline *N*-oxide with or without 1 equiv of KPF_6_ (entries 1 and 2) led to decomposition. Variation of temperature conditions from 100 °C to rt resulted again in decomposition^14^ or starting material recovery (entry 2 and Table S1a).^15^ Interestingly, using the ammonium salt **1a**·**HCl**, to favor a controlled release of the amine during the reaction, allowed us to obtain the desired seven-membered 3-benzazepin-4-one **2a** albeit in a low yield (entry 3 and Table S1b).^16^ We then analyzed the use of salt additives in the course of the reaction (Table S1b).^8^ Thus, addition of KPF_6_ (0.3 equiv) gave a moderate yield of **2a** (entry 4), which could be slightly increased to 58% yield when 1 equiv of KPF_6_ was added (entry 5).^17^ These results would suggest that KPF_6_ might be involved not only in the formation of a cationic ruthenium catalyst but also as a counterion of the ammonium substrate.^18^ Interestingly, the amount of oxidant employed had a significant effect in the efficiency of the reaction (Table S1e). Thus, when the amount of *N*-oxide was reduced to 1.1 equiv (entry 6) an excellent 90% yield of 3-benzazepin-4-one **2a** was obtained.^19^ To our delight, excellent yields of **2a** were also obtained when loadings of 3% of catalyst were used even at 60 °C (entry 7 and Table S1f).^20^

**Scheme 2:**
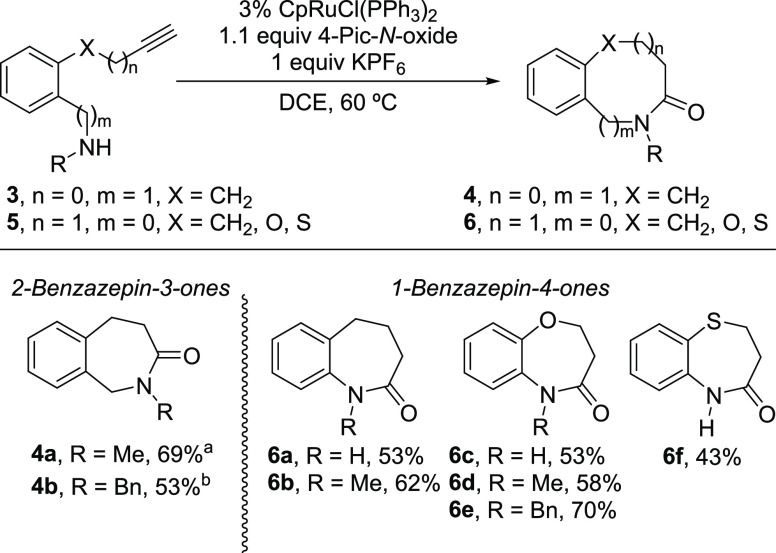
1- and 2-Benzazepin-2- and -3-ones by Ru-Catalyzed Oxidative Lactamization of Aromatic 1,5-Alkynylamines Ammonium salt **3a·HCl**, 0.3 equiv K_2_CO_3_. Ammonium salt **3b·HCl**, 0.3 equiv K_2_CO_3_.

Thus, the ammonium salts of (*o*-propynylphenyl)methanamines **3a·HCl** (R = Me) and **3b·HCl** (R = Bn) smoothly underwent the oxidative lactamization to give the corresponding 2-benzazepin-3-ones **4a** and **4b** in reasonable good yields (Scheme 2). As in the case of intermolecular oxidative amidation of anilines,^8^ the free primary and secondary *o*-butynylanilines **5a** and **5b** (R = H and Me, X = CH_2_) were readily converted into the corresponding 1-benzazepin-2-ones **6a** and **6b** in fairly good yields. The presence of a heteroatom in the linker is well tolerated. Thus, the free primary and secondary (*o*-propynyloxy)anilines **5c** (R = H, X = O), **5d** (R = Me, X = O), and **5e** (R = Bn, X = O) smoothly cyclized to give the corresponding 1,4-benzoxazepinones **6c**–**e** in moderate to good yields. In the case of the free primary (*o*-propynylthio)aniline **5f** (R = H, X = S), a slow conversion was observed to give the 1,4-benzothiazepinone **6f** in a moderate 43% yield.

**Scheme 3:**
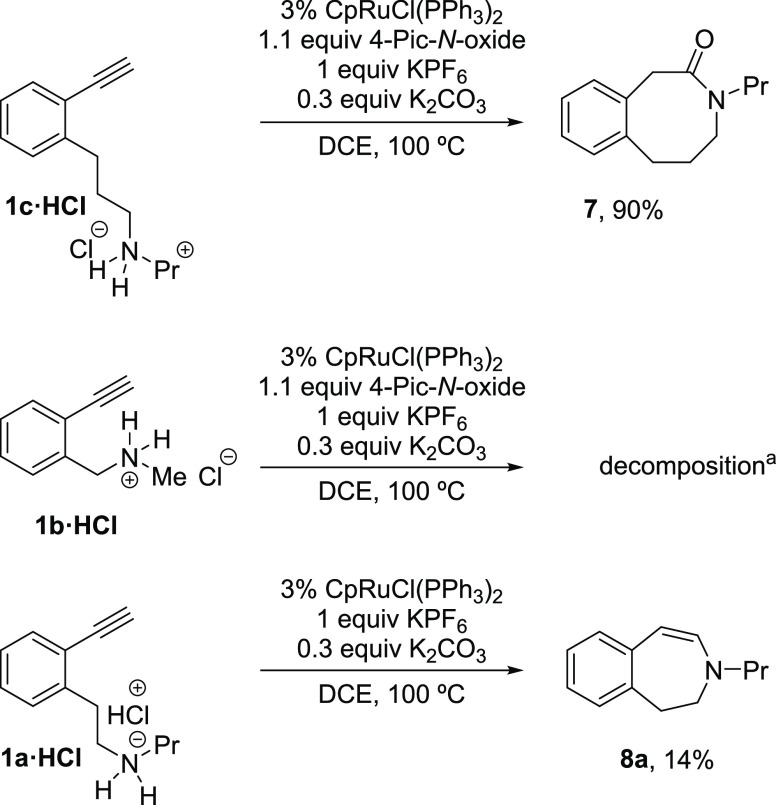
Ru-Catalyzed Oxidative Lactamization of Aromatic 1,4- and 1,6-Alkynylamines **1b·HCl** and **1c·HCl** and Hydroamination of Aromatic 1,5- and 1,4-Alkynylamines **1a·HCl** and **1b·HCl** Same result without 4-Pic-*N*-oxide.

Pleasingly, oxidative lactamization seems a very reliable process for the construction of medium-sized lactams since the higher homolog 1,6-alkynylamine, as ammonium salt **1c·HCl**, smoothly afforded the corresponding eight-membered 3-benzazocinone **7** in an excellent 90% yield (Scheme 3). Unfortunately, the next homolog member 4-(2-ethynylphenyl)-*N*-propylbutan-1-amine failed to react due probably to an unfavorable Thorpe–Ingold effect. Conversely, the ammonium salt of the lower homolog 1,4-alkynylamine **1b·HCl** slowly decomposed under the same conditions (Scheme 3). This last unexpected result led us to examine the competitive intramolecular hydroamination reaction of alkynylamines **1a·HCl** and **1b·HCl** (Scheme 3). Thus, while the 1,5-alkynylamine **1a·HCl** gave the desired 3-benzazepine **8a** but in a low 14% yield, the lower homolog 1,4-alkynylamine **1b·HCl** gave, as somewhat expected, decomposition.^9a^

**Scheme 4:**
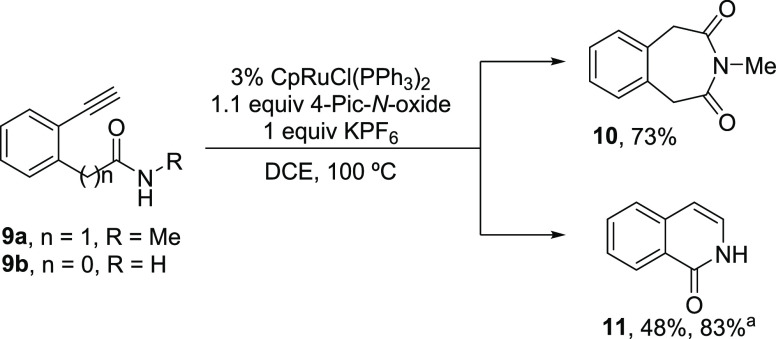
Ru-Catalyzed Oxidative Lactamization and Intramolecular Hydroamination of 1,5-Alkynylamide **9a** and 1,4-Alkynylamide **9b** In the absence of 4-Pic-*N*-oxide.

We speculate that the benzylamine derivatives could undergo competitive intramolecular C–H activation processes that lead to complex mixtures or decomposition. For this reason, we turned our attention to the evaluation of alkynylamide derivatives in the cyclization processes (Scheme 4). Thus, the 1,5-alkynylamide **9a** underwent an oxidative lactamization to afford the imide **10** (benzazepine-2,4(3H)-dione) in a quite good yield, and the lower homolog 1,4-alkynylamide **9b** gave the hydroamination product **11** (isoquinolin-1-one) in a moderate 48% yield under the same oxidative conditions or a 83% yield in the absence of 4-Pic-*N*-oxide (Scheme 4).^21^ By contrast, the reaction of 1,5-alkynylamide **9a** in the absence of 4-Pic-*N*-oxide gave a complex mixture.

**Scheme 5:**
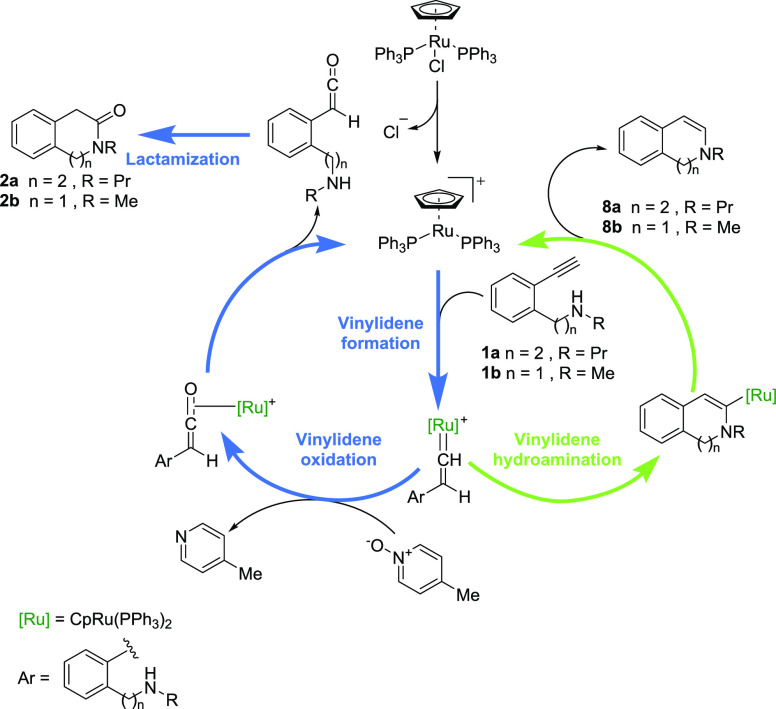
Competitive Mechanisms for the Ru(II)-Catalyzed Oxidative Lactamization (blue) and Ru(II)-Catalyzed Alkyne Intramolecular Hydroamination (Green) of 1,5- and 1,4-Alkynylamines

The novelty of the transformation prompted us to undertake a DFT mechanistic analysis to unveil the origin of the chemoselectivity observed toward oxidative lactamization *vs* intramolecular hydroamination of alkynylamines.^22^ For this study, both 1,5-alkynylamine **1a** and its lower homolog 1,4-alkynylamine **1b** have been used as model substrates. Following our previous mechanistic proposal,^8^ the catalytic cycle for the oxidative lactamization can be divided in three main steps (Scheme 5, blue): (i) Ru(II)-catalyzed vinylidene formation, (ii) Ru(II)-vinylidene oxidation by external 4-picoline *N*-oxide, and (iii) metal-free lactamization of the generated ketene. On the other hand, the competitive Ru(II)-hydroamination processes toward the formation of dihydro-3-benzazepine **8a** and dihydroisoquinoline **8b** (not experimentally observed) would proceed through trapping of the initially formed Ru-vinylidenes by intramolecular hydroamination (Scheme 5, green).

**Figure 1:**
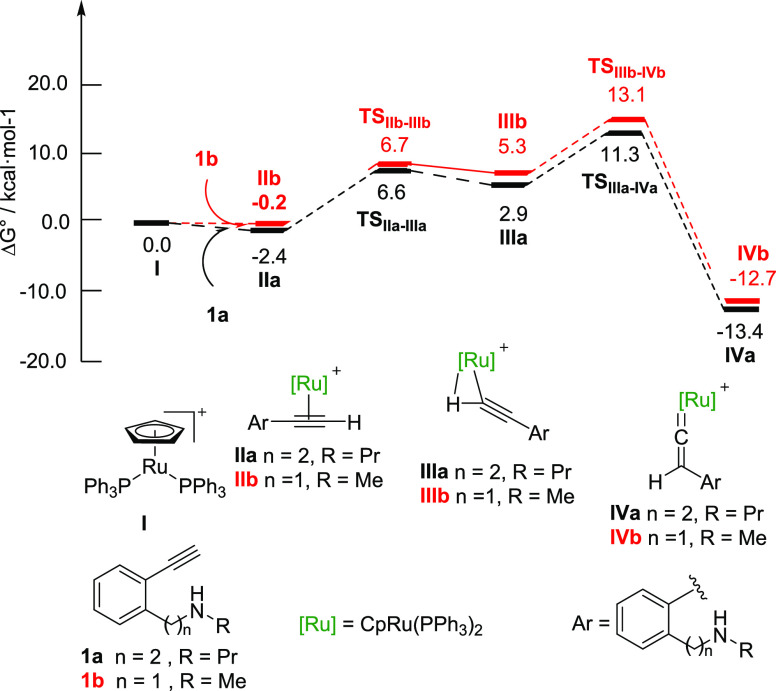
Gibbs energy profiles (DLPNO-CCSD(T)/def2-TZVP//B3LYP-D3/6-31G(d,p)-SDD(Ru)_DCE(SMD)_ 373 K) for the Ru(II)-vinylidenes **IVa** and **IVb**. Energies are relative to [CpRu(PPh_3_)_2_]^+^**I** and alkynylamines **1a** and **1b**, respectively, and are mass balanced.

The computed Gibbs energy profile for the formation of Ru-vinylidenes from **1a** (black color) and **1b** (red color) is depicted in Figure 1.

The DFT computed mechanism starts with the chloride release forming the active catalyst [CpRu(PPh_3_)_2_]^+^, **I**, entailing the formation of the η^2^-alkyne intermediate **IIa** and **IIb** at −2.4 and −0.2 kcal·mol^–1^, respectively. The activation of the substrates goes through the well-established 1,2-hydrogen migration favored by the agostic interaction with the methylidyne C–H bond in intermediates **III**.^23,24^ The energy barrier, **TS_II–III_**, is quite affordable requiring 6.6 and 6.7 kcal·mol^–1^ for the formation of the agostic intermediates **IIIa** and **IIIb** falling at 2.9 and 5.3 kcal·mol^–1^, respectively. Intermediates **III** evolve through a 1,2-hydrogen migration overcoming energy barriers of 11.3 and 13.1 kcal·mol^–1^ to the Ru(II)-vinylidene intermediates **IVa** and **IVb** at −13.4 and −12.7 kcal mol^–1^, respectively. Experimentally, in a deuteration study of the Ru-catalyzed heterocyclization of aromatic bis-homopropargyl alcohols, we had already shown results that indicate the formation of ruthenium vinylidene species as key intermediates.^25^ In addition, other competitive cyclization experiments also showed the involvement of vinylidene intermediates. Thus, heterocyclization of 1,4-alkynylanilide with catalytic CpRuCl(PPh_3_)_2_ gave rise to the corresponding 1,4-dihydroquinolinone, a product derived from hydroamination through a Ru-vinylidene intermediate, while with catalytic RuCl_2_(*p*-cymene)_2_/PBu_3_ gave the indol derivative, most likely through a typical alkyne activation/aromatization.^9a^

**Figure 2:**
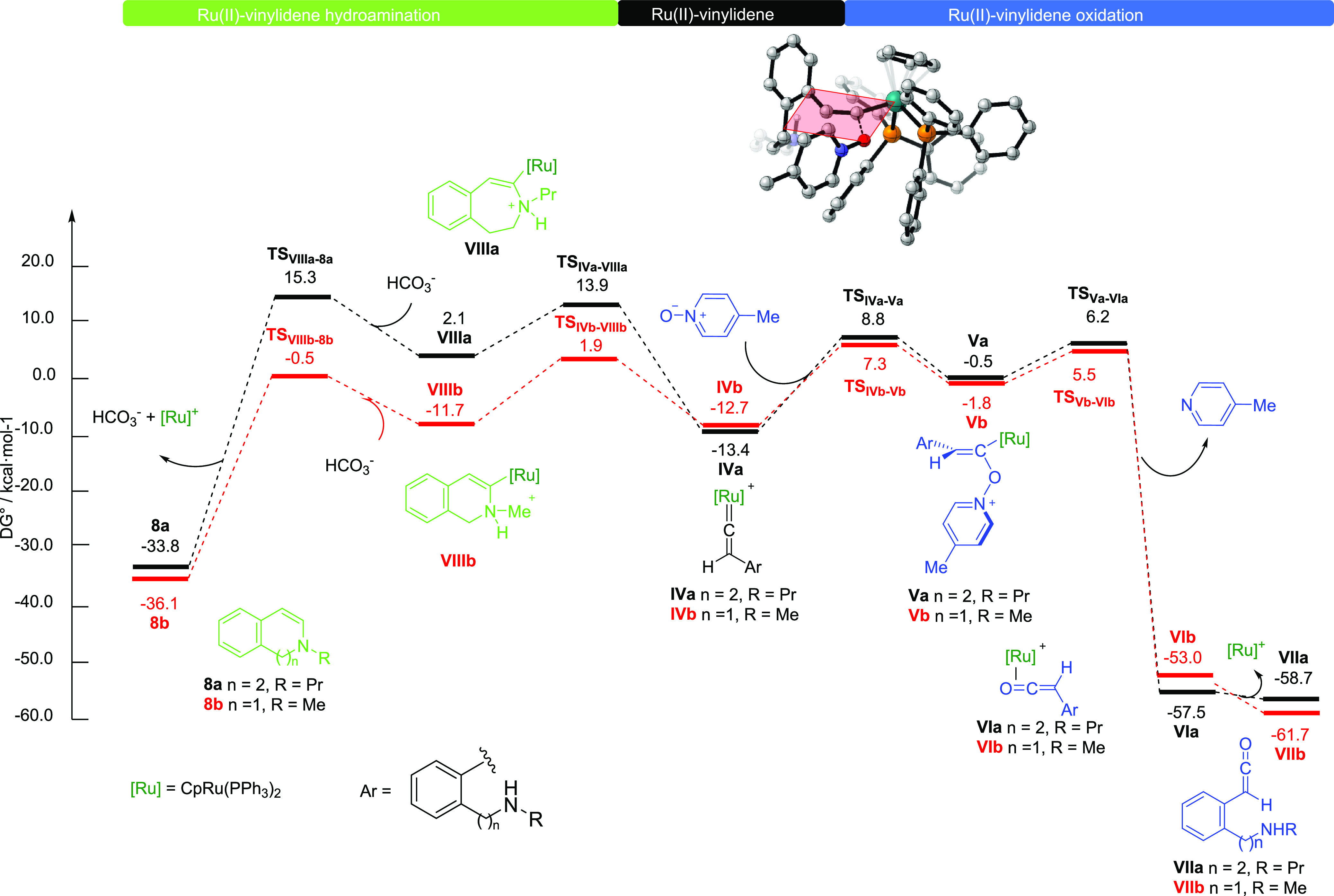
Gibbs energy profiles (DLPNO-CCSD(T)/def2-TZVP//B3LYP-D3/6-31G(d,p)-SDD(Ru)_DCE(SMD)_ 373 K) for the Ru(II)-catalyzed oxidation with picoline *N*-oxide (right) and for the intramolecular Ru(II)-catalyzed hydroamination of Ru(II)-vinylidenes **IVa** and **IVb** (left). Truncated structure of **TS_IVa–Va_** (inset draw). Energies are relative to [CpRu(PPh_3_)_2_]^+^**I** and alkynylamines **1a** and **1b**, respectively, and are mass balanced.

We next turned our attention to the Ru(II)-catalyzed vinylidene oxidation from **IVa** and **IVb**. The Gibbs energy profiles for **IVa** (black pathway) and **IVb** (red pathway) are depicted in Figure 2 (right side of the profile).

The nucleophilic attack of picoline *N*-oxide to the C_α_ of the Ru-vinylidene (Δ*G*^‡^ = 22.2 and 20.0 kcal mol^–1^ for **TS_IVa–Va_** and **TS_IVb–Vb_**, respectively) takes place in a coplanar approach to the Ru-vinylidene (Figure 2, inset drawing), affording intermediate **Va** and **Vb** at −0.5 and −1.8 kcal mol^–1^, respectively. Then, release of 4-picoline occurs (Δ*G*^‡^ = 6.7 and 7.3 kcal mol^–1^ for both **TS_Va–VIa_** and **TS_Vb–VIb_**, respectively) to afford η^2^-coordinated ruthenium ketene complexes **VIa** and **VIb**,^26^ which further evolve to the more stable free ketenes **VIIa** and **VIIb** with the recovery of the catalytic species **I** in a global exergonic process.

Competitive hydroamination pathways for both Ru(II)-vinylidenes **IVa** and **IVb** (Figure 2, left) were then analyzed. The initial step is the intramolecular nucleophilic attack of the amine to the C_α_ of the Ru(II)-vinylidenes **IV** to yield the cationic alkenylruthenium complexes **VIIIa** and **VIIIb**. The transition state of this step resulted in being significantly more favorable for vinylidene **IVb** (ΔΔ*G*^‡^ = (27.3 – 14.6) = 12.7 kcal·mol^–1^) presumably due to the less entropic penalty caused by the closer proximity of the reactive centers and the formation of a more stable six-membered ring intermediate. Similarly, from the formed cationic heterocycles **VIII**, the subsequent bicarbonate assisted deprotonation followed by protonolysis of the Ru–C bonds and Ru-decoordination displays a lower energy barrier (ΔΔ*G*^‡^ = (13.2 – 11.2) = 2.0 kcal mol^–1^, **TS_VIIIa–8a_***vs***TS_VIIIb–8b_**) for the formation of the dihydroisoquinoline **8b** rather than dihydrobenzazepine **8a**.^27^

Although from vinylidene **IVb** the intramolecular hydroamination to **8b** is more energetically favored than oxidative amidation with overall Gibbs energy barriers of 14.6 and 20.0 kcal mol^–1^, respectively, the cyclization of benzylamine **1b·HCl** gave decomposition products through other non-analyzed competitive pathways.^9a^ By contrast, from vinylidene **IVa**, oxidative lactamization to **2a** is more favorable than hydroamination with overall barriers of 22.2 and 27.3 kcal mol^–1^, respectively.^28^

**Figure 3:**
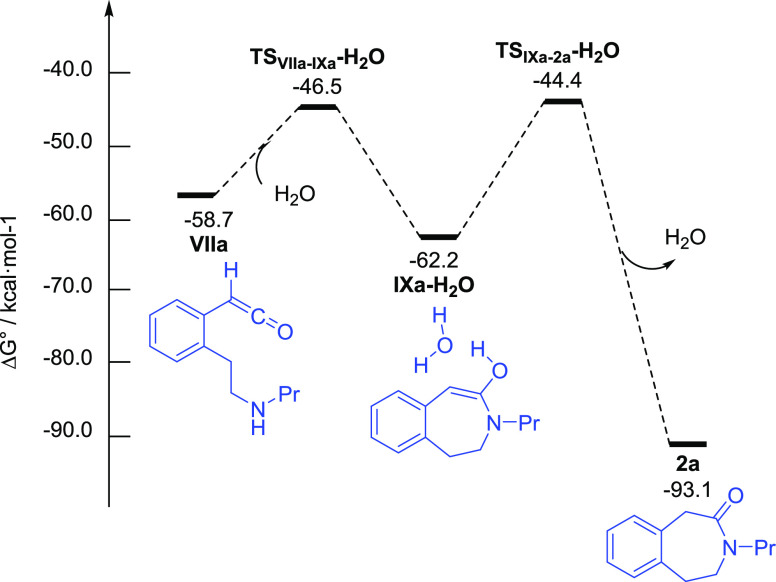
Gibbs energy profile (DLPNO-CCSD(T)/def2-TZVP//B3LYP-D3/6-31G(d,p)-SDD(Ru)_DCE(SMD)_ 373 K) for the lactamization of metal-free ketene **VIIa** with water acting as a proton carrier. Energies are relative to [CpRu(PPh_3_)_2_]^+^**I** and alkynylamine **1a** and are mass balanced.

Finally, to have a complete picture of the oxidative lactamization process, pathways involving metal-bound ketenes **VI** and metal-free ketenes **VII** as intermediates were evaluated.^29^ It was found that the most favorable pathways involve the metal-free ketenes **VII** with a water molecule acting as a proton carrier (Figure 3),^30^ whereas that of metal-bound ketene is higher in energy (Figures S2–S5, Supporting Information).^27^ Thus, intramolecular nucleophilic attack of the amine to the central carbon in ketene **VIIa** (Δ*G*^‡^ = 12.2 kcal mol^–1^) affords enol–water complex **IXa-H_2_O** (Δ*G*° = 3.5 kcal mol^–1^),^31^ which after keto-enol tautomerization (Δ*G*^‡^ = 17.8 kcal mol^–1^) and water release gives rise to the ε-lactam **2a** (Δ*G*° = −34.4 kcal mol^–1^).

**Figure 4:**
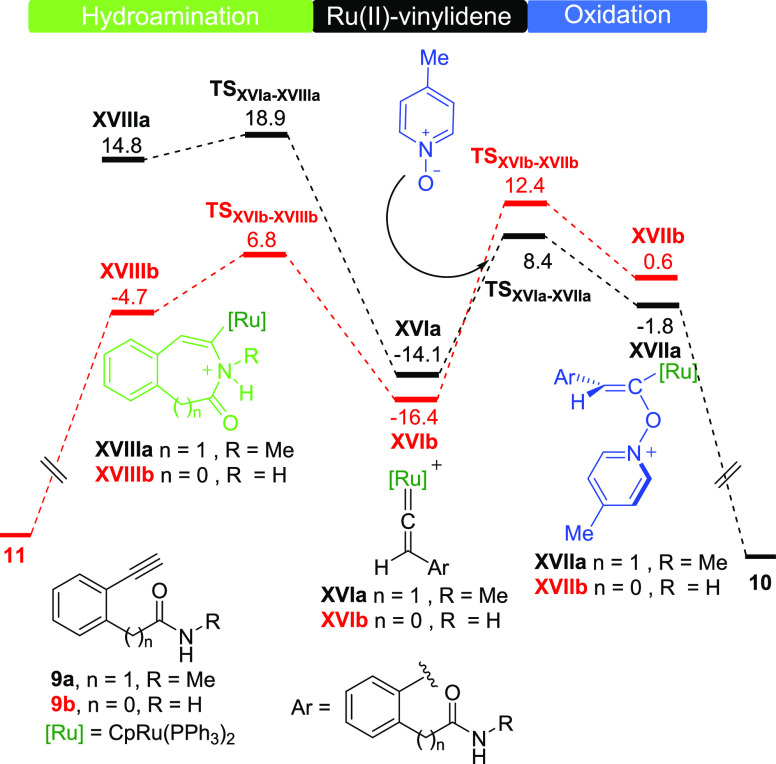
Gibbs energy profiles (DLPNO-CCSD(T)/def2-TZVP//B3LYP-D3/6-31G(d,p)-SDD(Ru)_DCE(SMD)_ 373 K) for the Ru(II)-catalyzed nucleophilic addition of picoline *N*-oxide (right) and the intramolecular nucleophilic addition of nitrogen to vinylidene (left) of Ru(II)-vinylidenes species **XVIa** and **XVIb** derived from alkynylamides **9a** and **9b**, respectively. Energies are relative to [CpRu(PPh_3_)_2_]^+^**I** and alkynylamines **9a** and **9b**, respectively, and are mass balanced.

Once the complete cyclization energetic profiles of alkynylamines were analyzed, we calculate the cyclization rate-determining steps of alkynylamides **9a** and **9b** (Figure 4). Starting from vinylidene **XVIa**, oxidative lactamization to **10** (Scheme 4) is more favorable than hydroamination with overall barriers of 22.5 and 33.0 kcal mol^–1^, respectively. Conversely, from vinylidene **XVIb**, the intramolecular hydroamination to **11** (Scheme 4) is more favorable than oxidative amidation with overall Gibbs energy barriers of 23.2 and 28.8 kcal mol^–1^, respectively. According to these results, the chain length between the alkyne and amide functionalities is crucial for the chemoselectivity found. The effects caused by the longer carbon-tethered in **9a** (1,5-alkynylamide) *vs***9b** (1,4-alkynylamide) slows down the intramolecular direct nucleophilic attack of the nitrogen to the vinylidene making the intermolecular addition of the picoline *N*-oxide a competitive process.

## Conclusions

Medium-sized benzofused seven-membered lactams (1-, 2-, 3-benzazepin-2-, -3-, and -4-ones) and eight-membered lactams (3-benzazocinone) could be efficiently assembled by chemoselective Ru-catalyzed oxidative lactamization of aromatic 1,5-alkynylamines and 1,6-alkynylamines with 4-picoline *N*-oxide as an external oxidant. DFT mechanistic studies revealed that the oxidation of the catalytic Ru-vinylidene intermediate occurs via a coplanar intermolecular nucleophilic attack of the 4-picoline *N*-oxide to the electrophilic α carbon. The chain length between the alkyne and the amine functionalities (1,5- and 1,6-alkynylamines) proved to be crucial for the chemoselective oxidation since only ε- and ζ-lactams could be obtained but not δ-lactams. In the case of 1,4-alkynylamides, the hydroamination product was obtained on being more favorable the intramolecular nucleophilic attack to the Ru-vinylidene.

## Experimental Section

All reactions were performed under an argon atmosphere, and the glassware was oven dried at 80 °C or flame dried unless otherwise stated. All dry solvents were stored under an argon atmosphere and over 4 Å molecular sieves. All chemicals were purchased from Acros Organics, TCI Chemicals, Sigma-Aldrich, Alfa Aesar, or Strem Chemicals chemical companies and used without further purification. The catalyst for Sonogashira coupling, PdCl_2_(PPh_3_)_2_, was prepared according to previously published procedure.

For the catalytic reactions, the CpRuCl(PPh_3_)_2_ catalyst used was purchased from Strem; the 4-picoline-*N*-oxide was purchased from Aldrich, and the DCE anhydrous was purchased from Acros Organics.

Analytical thin-layer chromatography was carried out on silica-coated aluminum plates (silica gel 60 F254 Merck) using UV light as a visualizing agent (254 nm) and KMnO_4_ (solution of 1.5 g of potassium permanganate, 10 g of potassium bicarbonate and 1.25 mL of 10% sodium hydroxide in 200 mL of water) or *p*-anisaldehyde (solution of 3.7 mL of *p*-anisaldehyde, 1.5 mL of glacial acetic acid, 5 mL of conc. sulfuric acid in 135 mL of absolute ethanol) with heat as developing agents. Flash column chromatography was performed on silica gel 60 (Merck, 230–400 mesh) with the indicated eluent.

^1^H and ^13^C nuclear magnetic resonance experiments were carried out using a Varian Inova 400 MHz or a Varian Mercury 300 MHz. Coupling constants J are given in Hertz (Hz). Multiplicities are reported as follows: s = singlet, bs = broad singlet, d = doublet, t = triplet, q = quartet, sxt = sextet, m = multiplet, or as a combination of them. Multiplicities of ^13^C NMR signals were determined by DEPT experiments. Yields refer to isolated compounds estimated to be >95% pure as determined by ^1^H NMR.

### Ru-Catalyzed Oxidative Lactamizations

#### General Procedure A

A suspension of the corresponding alkynylamine (1 equiv), CpRuCl(PPh_3_)_2_ (0.03 equiv), 4-Pic-*N*-oxide (1.1 equiv), and KPF_6_ (1 equiv) in DCE was heated in a screw-cap vial until complete disappearance of a starting material (TLC monitoring). The resulting mixture was washed with saturated solution of CuSO_4_ and extracted with DCM (3 × 10 mL). The combined organic layers were dried (Na_2_SO_4_) and concentrated *in vacuo*. The residue was purified by silica gel flash column chromatography to yield the corresponding lactam.

#### General Procedure B

A suspension of the corresponding alkynylamine hydrochloride (1 equiv), CpRuCl(PPh_3_)_2_ (0.03 equiv), K_2_CO_3_ (0.3 equiv), 4-Pic-*N*-oxide (1.1 equiv), and KPF_6_ (1 equiv) in DCE was heated in a screw-cap vial until complete disappearance of the starting material (TLC monitoring). The resulting mixture was washed with saturated solution of CuSO_4_ and extracted with DCM (3 × 10 mL). The combined organic layers were dried (Na_2_SO_4_) and concentrated *in vacuo*. The residue was purified by silica gel flash column chromatography to yield the corresponding lactam.

#### 3-Propyl-1,3,4,5-tetrahydro-2H-benzo[*d*]azepin-2-one (**2a**)

Procedure B. The product was purified by silica gel chromatography (EtOAc/Hex 1:1). Compound **2a**, 0.04 g (97%), yellow oil. ^1^H NMR (500 MHz, CDCl_3_): δ 7.18–7.14 (m, 1H), 7.13–7.10 (m, 1H), 7.10–7.06 (m, 2H), 3.89 (s, 2H), 3.77–3.61 (m, 2H), 3.44–3.30 (m, 2H), 3.15–3.09 (m, 2H), 1.58 (sxt, *J =* 7.4 Hz, 2H), 0.89 (t, *J =* 7.4 Hz, 3H). ^13^C{1H} NMR, DEPT (126 MHz, CDCl_3_): δ 171.8 (CO), 135.9(C), 131.8(C), 131,1 (CH), 130.3 (CH), 127.1 (CH), 126.6 (CH), 48.7 (CH_2_), 46.5 (CH_2_), 43.4 (CH_2_), 32.9 (CH_2_), 21.6 (CH_2_), 11.4 (CH_3_). MS (CI), *m*/*z* (%): 204 (M + 1, 100). HRMS (EI-TOF) *m*/*z*: [M]^+^ calcd for C_13_H_17_NO: 203.1310; found: 203.1315.

#### 2-Methyl-1,2,4,5-tetrahydro-3H-benzo[*c*]azepin-3-one (**4a**)

Known compound.^32^ Procedure B. The product was purified by silica gel chromatography (EtOAc/Hex 7:3). Compound **4a**, 0.024 g (69%), yellow oil. ^1^H NMR (500 MHz, CDCl_3_): δ 7.25–7.22 (m, 1H), 7.15–7.06 (m, 3H), 4.48 (s, 2H), 3.16 (t, *J =* 6.8 Hz, 2H), 3.04 (s, 3H), 2.91 (t, *J =* 6.8 Hz, 2H). ^13^C{1H} NMR, DEPT (126 MHz, CDCl_3_): δ 173.6 (CO), 137.7 (C), 134.2 (C), 130.5 (CH), 128.8 (CH), 128.1 (CH), 125.9 (CH), 54.4 (CH_2_), 35.2 (CH_3_), 33.5 (CH_2_), 28.7 (CH_2_).

#### 2-Benzyl-1,2,4,5-tetrahydro-3H-benzo[*c*]azepin-3-one (**4b**)

Procedure B. The product was purified by silica gel chromatography (EtOAc/Hex 3:7). Compound **4b**, 0.027 g (53%), colorless oil. ^1^H NMR (300 MHz, CDCl_3_): δ 7.63–7.53 (m, 4H), 7.21–7.13 (m, 3H), 7.06–7.04 (m, 1H), 6.85–6.82 (m, 1H), 4.66 (s, 2H), 4.40 (s, 2H), 3.22 (t, *J =* 6.8 Hz, 2H), 3.00 (t, *J =* 6.8 Hz, 2H). ^13^C{1H} NMR, DEPT (75 MHz, CDCl_3_): δ 173.1 (CO), 140.4 (C), 138.2 (C), 136.5 (C), 129.5 (CH), 129.3 (CH), 128.4 (2× CH), 127.9 (2× CH), 127.8 (2× CH), 127.7 (CH), 50.0 (CH_2_), 49.1 (CH_2_), 34.4 (CH_2_), 28.8 (CH_2_). HRMS (EI-TOF) *m*/*z*: [M]^+^ calcd for C_17_H_17_NO: 251.1310; found: 251.1316.

#### 1,3,4,5-Tetrahydro-2H-benzo[*b*]azepin-2-one (**6a**)

Known compound.^33^ Procedure A. The product was purified by silica gel chromatography (EtOAc/Hex 3:7). Compound **6a**, 0.017 (53%), yellow oil. ^1^H NMR (400 MHz, CDCl_3_): δ 7.70 (bs, 1H), 7.27–7.19 (m, 2H), 7.13 (td, *J =* 7.3, 1.3 Hz, 1H), 7.01–6.93 (m, 1H), 2.81 (t, *J =* 7.2 Hz, 2H), 2.36 (t, *J =* 7.2 Hz, 2H), 2.29–2.17 (m, 2H). ^13^C{1H} NMR, DEPT (101 MHz, CDCl_3_): δ 175.2 (CO), 137.9 (C), 134.5 (C), 130.0 (CH), 127.6 (CH), 125.9 (CH), 121.9 (CH), 32.9 (CH_2_), 30.5 (CH_2_), 28.6 (CH_2_). MS (CI), *m*/*z* (%): 162 (M + 1, 100).

#### 1-Methyl-1,3,4,5-tetrahydro-2H-benzo[*b*]azepin-2-one (**6b**)

Known compound.^34^ Procedure A. The product was purified by silica gel chromatography (EtOAc/Hex 3:7). Compound **6b**, 0.022 g (62%), yellow oil. ^1^H NMR (500 MHz, CDCl_3_): δ 7.29 (ddd, *J =* 7.9, 7.0, 1.8 Hz, 1H), 7.21–7.13 (m, 3H), 3.35 (s, 3H), 2.72 (t, *J =* 7.2 Hz, 2H), 2.30 (t, *J =* 7.2 Hz, 2H), 2.19–2.14 (m, 2H). ^13^C{1H} NMR, DEPT (126 MHz, CDCl_3_): δ 173.5 (CO), 143.9 (C), 135.3 (C), 129.4 (CH), 127.6 (CH), 126.2 (CH), 122.4 (CH), 35.3 (CH_3_), 33.3 (CH_2_), 30.2 (CH_2_), 29.0 (CH_2_). MS (CI), *m*/*z* (%): 176 (M + 1, 100). HRMS (CI-TOF) *m*/*z*: [M + H]^+^ calcd for C_11_H_14_NO: 176.1070; found: 176.1064.

#### 2,3-Dihydrobenzo[*b*][1,4]oxazepin-4(5H)-one (**6c**)

Known compound.^35^ Procedure A. The product was purified by silica gel chromatography (EtOAc/Hex 1:1 to EtOAc). Compound **6c**, 0.017 g (53%), yellow oil. ^1^H NMR (400 MHz, CDCl_3_): δ 8.28 (bs, 1H), 7.08–7.00 (m, 3H), 6.99–6.94 (m, 1H), 4.46 (t, *J =* 5.7 Hz, 2H), 2.86 (t, *J =* 5.7 Hz, 2H). ^13^C{1H} NMR, DEPT (101 MHz, CDCl_3_): δ 172.9 (CO) 148.6 (C), 128.9 (C), 125.5 (CH), 123.8 (CH), 122.2 (CH), 121.7 (CH), 68.8 (CH_2_), 36.9 (CH_2_). MS (CI), *m*/*z* (%): 164 (M + 1, 100).

#### 5-Methyl-2,3-dihydrobenzo[*b*][1,4]oxazepin-4(5H)-one (**6d**)

Procedure A. The product was purified by silica gel chromatography (EtOAc/Hex 1:1 to EtOAc). Compound **6d**, 0.021 g (58%), yellow oil. ^1^H NMR (400 MHz, CDCl_3_): δ 7.21–7.07 (m, 4H), 4.57 (t, *J =* 6.6 Hz, 2H), 3.35 (s, 3H), 2.64 (t, *J =* 6.6 Hz, 2H). ^13^C{1H} NMR, DEPT (101 MHz, CDCl_3_): δ 171.0 (CO), 149.5 (C), 138.3 (C), 127.0 (CH), 125.2 (CH), 123.1 (CH), 122.9 (CH), 74.5 (CH_2_), 35.2 (CH_2_), 35.0 (CH_3_). MS (CI), *m*/*z* (%): 178 (M + 1, 100). HRMS (CI-TOF) *m*/*z*: [M + H]^+^ calcd for C_10_H_12_NO_2_: 178.0863; found: 178.0860.

#### 5-Benzyl-2,3-dihydrobenzo[*b*][1,4]oxazepin-4(5H)-one (**6e**)

Procedure A. The product was purified by silica gel chromatography (EtOAc/Hex 3:7). Compound **6e**, 0.035 g (70%), yellow oil. ^1^H NMR (500 MHz, CDCl_3_): δ 7.30–7.07 (m, 9H), 5.07 (s, 2H), 4.62 (t, *J =* 6.6 Hz, 2H), 2.74 (t, *J =* 6.6 Hz, 2H). ^13^C{1H} NMR (126 MHz, CDCl_3_): δ 171.2 (CO), 149.9 (C), 137.5 (C), 137.3 (C), 128.7 (2× CH), 127.30 (CH), 127.26 (CH), 127.1 (2× CH), 125.2 (CH), 123.2 (CH), 123.0 (CH), 74.6 (CH_2_), 51.0 (CH_2_), 35.3 (CH_2_). MS (CI), *m*/*z* (%): 254 (M + 1, 100). HRMS (CI-TOF) *m*/*z*: [M + H]^+^ calcd for C_16_H_16_NO_2_: 254.1176; found: 254.1176.

#### 2,3-Dihydrobenzo[*b*][1,4]thiazepin-4(5H)-one (**6f**)

Known compound.^36^ Procedure A. The product was purified by silica gel chromatography (EtOAc/Hex 4:6). Compound **6f**, 0.031 g (43%), brown semisolid. ^1^H NMR (500 MHz, CDCl_3_): δ 8.41 (bs, 1H), 7.60 (dd, *J =* 7.7, 1.5 Hz, 1H), 7.35 (td, *J =* 7.7, 1.5 Hz, 1H), 7.16 (td, *J =* 7.7, 1.5 Hz, 1H), 7.11 (dd, *J =* 7.7, 1.5 Hz, 1H), 3.48–3.41 (m, 2H), 2.63 (t, *J =* 6.9 Hz, 2H). ^13^C{1H} NMR, DEPT (126 MHz, CDCl_3_): δ 174.0 (CO), 141.6 (C), 135.6 (CH), 129.9 (CH), 127.0 (C), 126.6 (CH), 123.4 (CH), 34.5 (CH_2_), 33.7 (CH_2_). MS (CI), *m*/*z* (%): 180 (M + 1, 100).

#### 3-Propyl-3,4,5,6-tetrahydrobenzo[*d*]azocin-2(1H)-one (**7**)

Known compound.^37^ Procedure B. The product was purified by silica gel chromatography (EtOAc/Hex 3:7 to 1:1). Compound **7**, 0.039 g (90%), yellow oil. ^1^H NMR (500 MHz, CDCl_3_): δ (243 K): 7.41 (dd, *J =* 6.9, 2.0 Hz, 1H), 7.21–7.08 (m, 3H), 4.07 (d, *J =* 11.7 Hz, 1H), 3.87 (dd, *J =* 15.8, 11.3 Hz, 1H), 3.46–3.24 (m, 3H), 3.07–2.83 (m, 3H), 2.16–2.08 (m, 1H), 1.64–1.53 (m, 1H), 1.53–1.33 (m, 2H), 0.75 (t, *J =* 7.3 Hz, 3H). ^13^C{1H} NMR (126 MHz, CDCl_3_): δ (243 K): 173.0 (CO), 139.9 (C), 136.0 (C), 130.0 (CH), 129.8 (CH), 127.2 (CH), 127.2 (CH), 51.4 (CH_2_), 49.5 (CH), 40.2 (CH), 36.7 (CH), 30.2 (CH), 20.9 (CH), 11.3 (CH_3_). MS (CI), *m*/*z* (%): 218 (M + 1, 100). HRMS (EI-TOF) *m*/*z*: [M]^+^ calcd for C_14_H_19_NO: 217.1467; found: 217.1469.

#### 3-Methyl-1,5-dihydro-2H-benzo[*d*]azepine-2,4(3H)-dione (**10**)

Procedure A. The product was purified by silica gel chromatography (EtOAc/Hex 3:7). Compound **10**, 0.028 g (73%), orange needles. ^1^H NMR (500 MHz, CDCl_3_): δ 7.30–7.27 (m, 4H), 4.11 (s, 4H), 3.13 (s, 3H). ^13^C{1H} NMR, DEPT (126 MHz, CDCl_3_): δ 171.0 (CO), 131.9 (2× C), 128.6 (2× CH), 128.5 (2× CH), 45.0 (CH_2_), 29.8 (CH_3_). MS (CI), *m*/*z* (%): 190 (M + 1, 100), 163 (3), 162 (28). HRMS (CI-TOF) *m*/*z*: [M + H]^+^ calcd for C_11_H_12_NO_2_: 190.0863; found: 190.0862.

#### Isoquinolin-1(2H)-one (**11**)

Procedure A in the absence of oxidant. Known compound.^9a^ The product was purified by silica gel chromatography (EtOAc/Hex 1:1). Compound **11**, 0.042 g (83%), brown solid. ^1^H NMR (300 MHz, CDCl_3_): δ 11.49 (s, 1H), 8.43 (d, *J =* 8.00 Hz, 1H), 7.68 (t, *J =* 6.9 Hz, 1H), 7.59–7.51 (m, 2H), 7.20 (d, *J =* 7.1 Hz, 1H), 6.58 (d, *J =* 7.1 Hz, 1H). ^13^C{1H} NMR, DEPT (75 MHz, CDCl_3_): δ 164.4 (C=O), 138.1(C), 132.5 (CH), 127.6 (CH), 127.3 (CH), 126.8 (CH), 126.2 (CH), 126.1(C), 106.7 (CH). MS (EI) (*m*/*z*, %): 145 (M^+^, 100), 118 (36), 90 (29). HRMS (EI-TOF) *m*/*z*: [M]^+^ calcd for C_9_H_7_NO: 145.0528; found: 145.0528.

## Supporting Information Available

General experimental procedures, NMR spectra, and DFT calculations (PDF)
