# Supplementary material for: Chemoselective Ru-Catalyzed Oxidative Lactamization vs Hydroamination of Alkynylamines: Insights from Experimental and Density Functional Theory Studies
Source: J Org Chem. 2022 Dec 29;88(2):1185–93. doi: 10.1021/acs.joc.2c02770 (PMC9872091; doi:10.1021/acs.joc.2c02770)
Supplement: Supplementary file 1 — jo2c02770_si_001.pdf [file jo2c02770_si_001.pdf]

Supporting Information

Chemoselective Ru-Catalyzed Oxidative Lactamization *vs* Hydroamination of Alkynylamines: Insights from Experimental and DFT Studies

Andrés M. Álvarez-Constantino,[a] Andrea Álvarez-Pérez,[a] Jesús A. Varela,[a] Giuseppe Sciortino,[b*] Gregori Ujaque,[b]* and Carlos Saá[a]*

[a] Centro Singular de Investigación en Química Biolóxica e Materiais Moleculares (CiQUS), Departamento de Química Orgánica, Universidade de Santiago de Compostela, 15782 Santiago de Compostela, Spain

[b] Departament de Química and Centro de Innovación en Química Avanzada (ORFEO-CINQA), Universitat Autònoma de Barcelona, 08193 Cerdanyola del Vallès, Catalonia, Spain

*Email: Giuseppe.Sciortino@uab.cat

*Email: Gregori.Ujaque@uab.cat

*Email: carlos.saa@usc.es

Table of Contents

1 General experimental procedures S3

2 Optimization of the reaction conditions S4

3 Ru-catalyzed oxidative lactamizations S8

4 Hydroamination of alkynylamines S9

5 Chemoselective lactamization *vs* hydroamination of alkynylamides S11

6 Preparation and data of starting materials S12

7 NMR spectra of starting materials S26

8 NMR spectra of cyclized products S39

9. Computational details S52

10. Complete Gibbs energy profiles for all calculated pathways S54

11. Thermal rate constants k (373 K) for steps involving the most significant transitions states for microkinetic simulations S60

12. B3LYP energy values S64

13. DLPNO-CCSTD Energy values S69

14. Cartesian coordinates in Å for all the stationary points involved throughout DFT study S76

1 General experimental procedures

All reactions were performed under argon atmosphere and the glassware was oven dried at 80 ºC or flame dried unless otherwise stated. All dry solvents were stored under argon atmosphere and over 4 Å molecular sieves. All chemicals were purchased from Acros Organics, TCI Chemicals, Sigma-Aldrich, Alfa Aesar or Strem Chemicals chemical companies and used without further purification. The catalyst for Sonogashira coupling, $PdCl_2(PPh_3)_2$, was prepared according to previously published procedure.[1]

For the catalytic reactions, the $CpRuCl(PPh_3)_2$ catalyst used was purchased from Strem; the 4-picoline-*N*-oxide was purchased from Aldrich and the DCE anhydrous was purchased from Acros Organics.

For the heated reactions, silicon baths or aluminum plates were used.

Analytical thin layer chromatography was carried out on silica-coated aluminum plates (silica gel 60 F254 Merck) using UV light as visualizing agent (254 nm) and $KMnO_4$ (solution of 1.5 g of potassium permanganate, 10 g of potassium bicarbonate and 1.25 mL of 10% sodium hydroxide in 200 mL of water) or *p*-anisaldehyde (solution of 3.7 mL of p-anisaldehyde, 1.5 mL of glacial acetic acid, 5 mL of conc. sulfuric acid in 135 mL of absolute ethanol) with heat as developing agents. Flash column chromatography was performed on silica gel 60 (Merck, 230-400 mesh) with the indicated eluent.[2]

Reactions were also followed using a GC Agilent HP-6890N with a mass spectroscopy HP5973N using DB-35MS and HP-5MS columns for the GC and a chemical ionization font for the MS. Melting points were determined using a Büchi B-540 melting point apparatus. **^{1}H** and **^{13}C** Nuclear Magnetic Resonance experiments were carried out using a Varian Inova 400 MHz or a Varian Mercury 300 MHz. Coupling constants *J* are given in Hertz (Hz). Multiplicities are reported as follows: s = singlet, bs = broad singlet, d = doublet, t = triplet, q = quartet, sxt = sextet, m = multiplet or as a combination of them. Multiplicities of ^{13}C NMR signals were determined by DEPT experiments. Yields refer to isolated compounds estimated to be > 95% pure as determined by ^{1}H NMR.

---

[1] Miyaura, N.; Suzuki, A. Palladium-catalyzed reaction of 1-alkenylboronates with vinylic halides: (1*Z*,3*E*)-1-phenyl-1,3-octadiene. *Org. Synth.* **1990**, *68*, 130.

[2] Still, W. C.; Kahn, M.; Mitra, A. Rapid chromatographic technique for preparative separations with moderate resolution. *J. Org. Chem.* **1978**, *43*, 2923-2925.

2 Optimization of the reaction conditions

Complete optimization process summarized in Table S1.

Table S1a. Temperature variation with free amine 1a.[a]

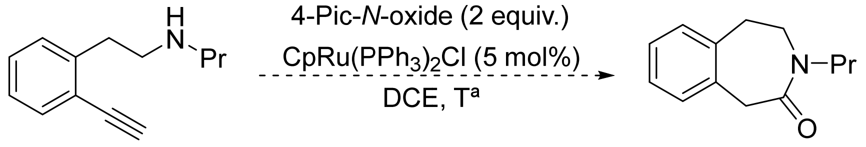

1a **2a**

Entry	T (°C)	Product **2a**[b]
1	rt	SM (96%)
2	50	SM (75%) + dec. traces
3	70	dec.
4[c]	100	dec.
5[d]	100	dec.

[a] Reaction conditions: 0.2 mmol of **1a** and 2 mL of DCE. [b] Determined by ^{1}H NMR using trimethoxybenzene as internal standard. [c] Sealed tube. [d] Sealed tube with 1 equiv. of KPF_6.

^{1}H NMR monitoring under the reaction conditions showed in Table S1a.

1H NMR spectrum of 1a

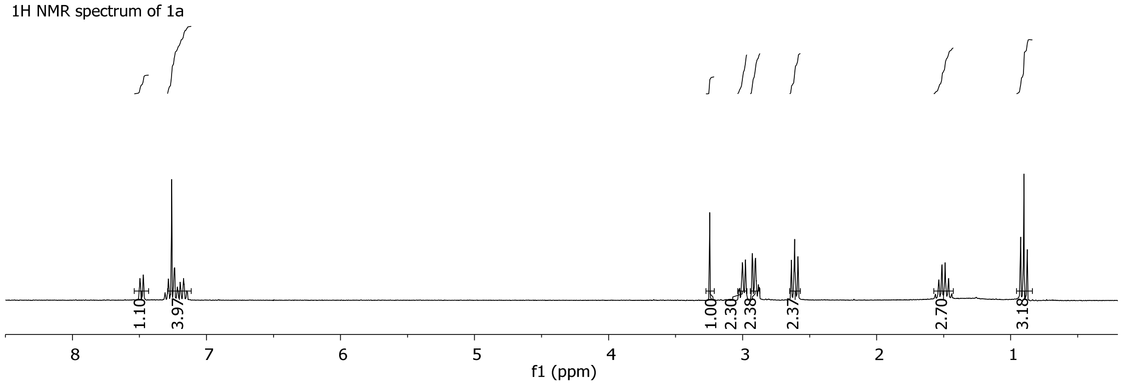

1H NMR spectrum of entry 1 (room temperature))

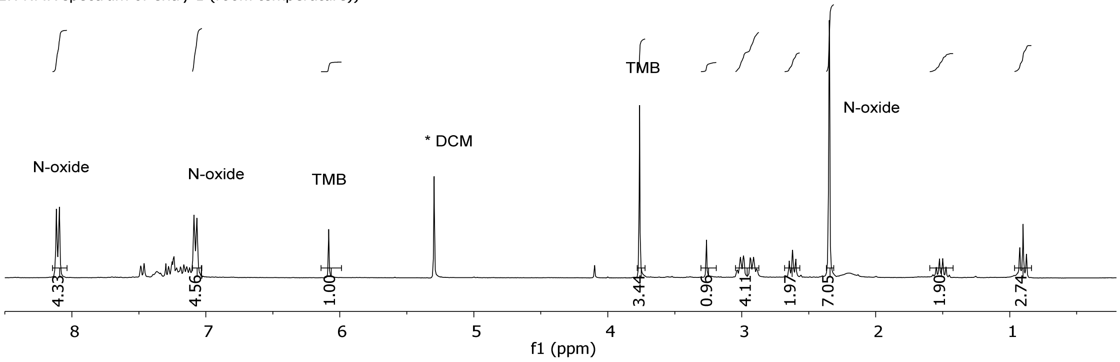

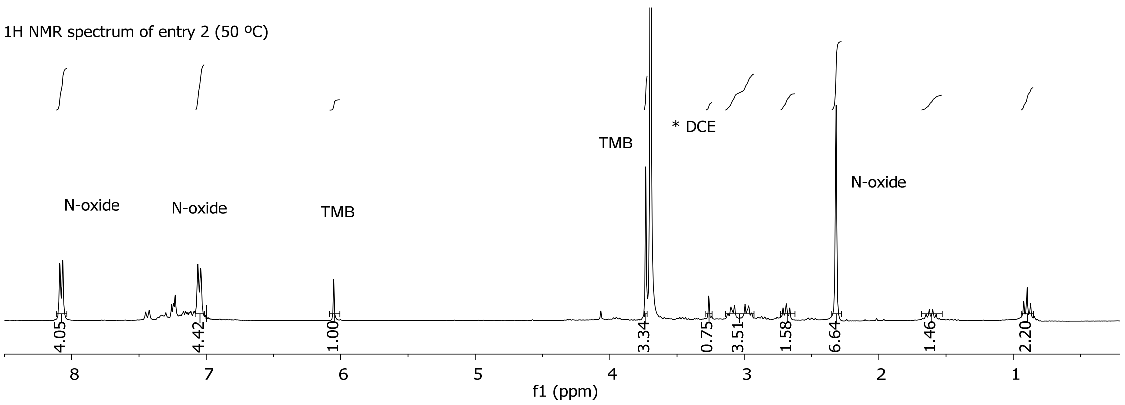

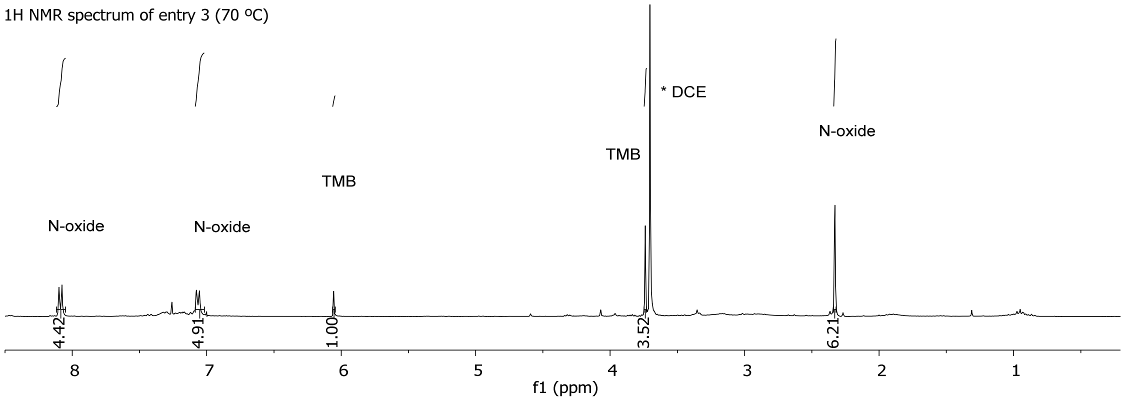

Table S1b. Additive effect with 1a·HCl.[a]

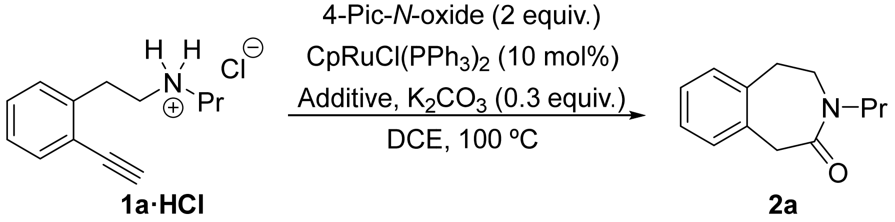

Entry	1	Additive	t (h)	Product **2a**[b]
1	**1a·HCl**	-	4	30%
2	**1a·HCl**	0.3 equiv. KPF_6	4	50%
3	**1a·HCl**	1 equiv. KPF_6	1	58%
4	**1a·HPF$_6$**	-	2	30%
5	**1a·HCl**	6 mol% $AgBF_4$	3	40%

[a] Reaction conditions: 0.2 mmol of **1a·HCl** and 2 mL of DCE in a sealed tube. [b] NMR yields are given using tetrachloroethane as internal standard.

Table S1c. Catalyst variations with 1a·HCl.[a]

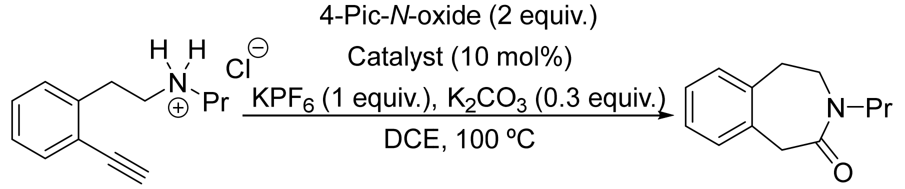

1a·HCl **2a**

Entry	Catalyst	Product **2a**[h]
1	$CpRuCl(PPh_3)_2$	58%
2	CpRuCl(dppe)	54%
3[b]	$[Ru(p\text{-cym})Cl_2]_2/P(4\text{-}FC_6H_4)_3$	SM + dec.
4[c]	CpRuCl(cod)	SM + dec.
5	$[CpRu(CH_3CN)_3]PF_6$	SM + dec.
6	$[Cp^*Ru(CH_3CN)_3]PF_6$	traces[i] + dec.
7[d]	$[Cp^*Ru(CH_3CN)_3]PF_6$ ${}^tBu_3P.BF_4/Et_4NCl$	traces
8[e]	$[RhCl(cod)]_2/P(4\text{-}FC_6H_4)_3$	traces
9[f]	$[RhCl(cod)]_2/P(4\text{-}FC_6H_4)_3$	dec.
10[g]	$RhCl(PPh_3)_3$	dec.

[a] Reaction conditions: 0.2 mmol of **1a·HCl** and 2 mL of DCE in a sealed tube. [b] 5 mol% of [Ru] catalyst and 20 mol% of $P(4\text{-}FC_6H_4)_3$. [c] 5 mol% of [Ru] catalyst. [d] 10 mol% of [Ru] catalyst, 20 mol% of ${}^tBu_3P.BF_4$ and 10 mol% Et_4NCl. [e] 2.5 mol% of [Rh] catalyst and 12 mol% of $P(4\text{-}FC_6H_4)_3$ in 2 mL of CH_3CN at 60 ºC. [f] 2.5 mol% of [Rh] catalyst and 12 mol% of $P(4\text{-}FC_6H_4)_3$. [g] 5 mol% of [Rh] catalyst at 60 ºC. [h] NMR yields are given using tetrachloroethane as internal standard. [i] Traces are considered less than 10% of yield.

Table S1d. Base optimization with 1a·HCl.[a]

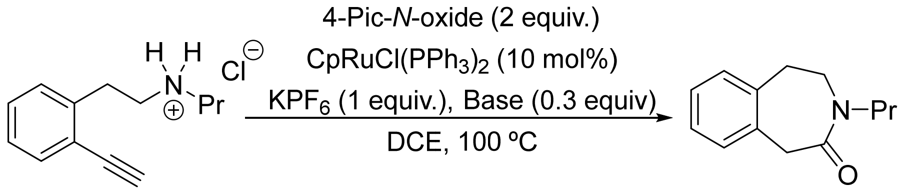

1a·HCl **2a**

Entry	Bases	Product **2a**[c]
1	K_2CO_3	58%
2	-	SM
3	Cs_2CO_3	46%
4	Et_3N	34%
5[b]	Pyridine	CM[d]

[a] Reaction conditions: 0.2 mmol of **1a·HCl** and 2 mL of DCE in a sealed tube. [b] Pyridine was used as solvent. [c] NMR yields are given using tetrachloroethane as internal standard. [d] Complex mixture.

Table S1e. Oxidants variation with 1a·HCl.[a]

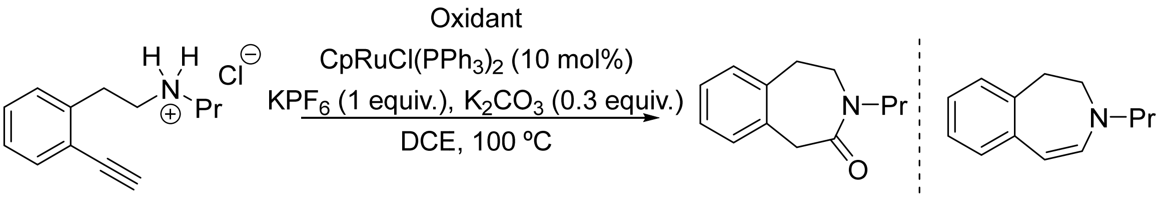

1a·HCl **2a** **8a**

Entry	Oxidant	t (h)	Product **2a**[b]
1	2 equiv. 4-Pic-*N*-oxide	1	58%
2	---	2	**8a**, 14%
3	1.1 equiv. 4-Pic-*N*-oxide	0.5	92% (90%)[c]
4	1.1 equiv. methylphenylsulfoxide	3	CM

[a] Reaction conditions: 0.2 mmol of **1a·HCl** and 2 mL of DCE in a sealed tube. [b] NMR yields area given using tetrachloroethane as internal standard. [c] Isolated yield.

Table S1f. Catalyst loading optimization.[a]

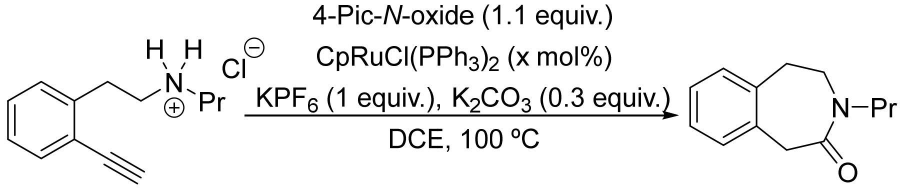

1a·HCl 2a

Entry	$CpRuCl(PPh_3)_2$	t (min)	Product **2a**[b]
1	10 mol%	30	92% (90%)[c]
2	5 mol%	30	93%
3	3 mol%	60	97% (96%)[c]

Reaction conditions: 0.2 mmol of **1a·HCl** and 2 mL of DCE in a sealed tube. [b] NMR yields using tetrachloroethane as internal standard. [c] Isolated yields.

Table S1g. Temperature optimization.[a]

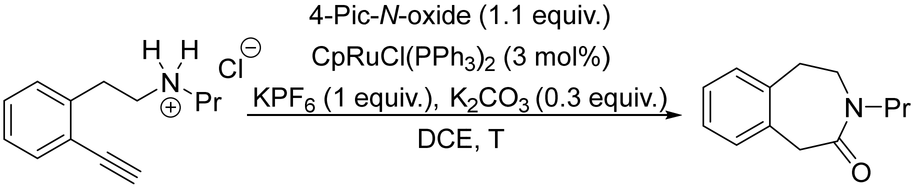

1a·HCl 2a

Entry	T (ºC)	t (h)	Product **2a**[b]
1	100	1	97%
2	60	7	99% (97%)[c]
3	ta	24	SM

[a] Reaction conditions: 0.2 mmol of **1a·HCl** and 2 mL of DCE in a sealed tube. [b] NMR yields are given using tetrachloroethane as internal standard. [c] Isolated yield.

3 Ru-catalyzed oxidative lactamizations

General procedure A: a suspension of the corresponding alkynylamine (1 equiv), $CpRuCl(PPh_3)_2$ (0.03 equiv), 4-Pic-*N*-oxide (1.1 equiv) and KPF*6* (1 equiv) in DCE was heated in a screw cap vial until complete disappearance of starting material (TLC monitoring). The resulting mixture was washed with saturated solution of $CuSO_4$ and extracted with DCM (3 x 10 mL). The combined organic layers were dried (Na_2SO_4) and concentrated *in vacuo*. The residue was purified by silica gel flash column chromatography to yield the corresponding lactam.

General procedure B: a suspension of the corresponding alkynylamine hydrochloride (1 equiv), $CpRuCl(PPh_3)_2$ (0.03 equiv), K_2CO_3 (0.3 equiv), 4-Pic-*N*-oxide (1.1 equiv) and KPF_6 (1 equiv) in DCE was heated in a screw cap vial until complete disappearance of starting material (TLC monitoring). The resulting mixture was washed with saturated solution of $CuSO_4$ and extracted with

DCM (3 x 10 mL). The combined organic layers were dried (Na_2SO_4) and concentrated in vacuo. The residue was purified by silica gel flash column chromatography to yield the corresponding lactam.

3.1 Ru-catalyzed reaction of aromatic 1,4-alkynylamine 1b·HCl.

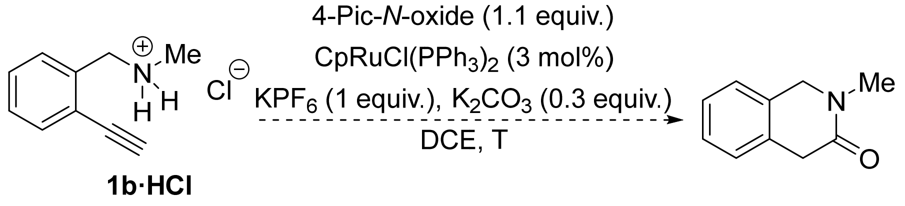

Entry	T (ºC)	T (h)	Product
1	100	4	dec.
2	37	24	CM

[a] Reaction conditions: 0.2 mmol of **1b·HCl** and 2 mL of DCE in a sealed tube.

Following *general procedure B*, decomposition of starting material at 100 ºC was observed and formation of complex mixture of products at 37ºC that could not be identified.

4 Hydroamination of alkynylamines

4.1 Alkynylamine 1a·HCl

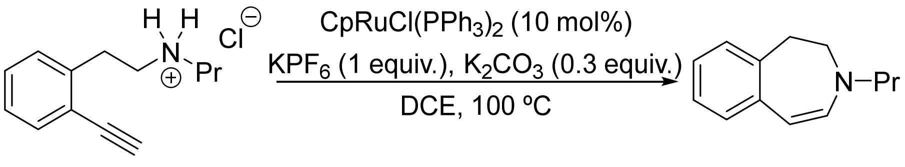

1a·HCl **8a**

Entry	T (ºC)	t (h)	Product **8a**
1	100	2	14% + CM
2	37	24	traces + SM

[a] Reaction conditions: 0.2 mmol of **1a·HCl** and 2 mL of DCE in a sealed tube.

General procedure B was followed with some modifications: $CpRuCl(PPh_3)_2$ (0.0015 g, 0.02 mmol, 0.1 equiv.), K_2CO_3 (0.008 g, 0.06 mmol, 0.3 equiv.), KPF_6 (0.037 g, 0.2 mmol, 1 equiv.) and **1a·HCl** (0.045 g, 0.2 mmol, 1 equiv.) in 2 mL of DCE were heated at 100 ºC (or 37 ºC) for 2 hours. The

residue was purified by flash column chromatography in silica gel (EtOAc/Hex 1:9) to afford **8a** (*Entry 1*) as a yellow oil (0.005 g, 14%). Spectroscopic data agree with those previously reported.[3]

^{1}H NMR (300 MHz, $CDCl_3$) δ 7.14 – 7.00 (m, 2H), 6.99 – 6.83 (m, 2H), 6.06 (d, *J* = 9.8 Hz, 1H), 5.00 (d, *J* = 9.8 Hz, 1H), 3.44 – 3.34 (m, 2H), 3.09 – 2.97 (m, 4H), 1.58 (sxt, *J* = 7.4 Hz, 2H), 0.90 (t, *J* = 7.4 Hz, 3H).

^{13}C{1H} NMR, DEPT (101 MHz, $CDCl_3$) δ 138.6 (C), 137.8 (C), 137.6 (CH), 128.8 (CH), 127.9 (CH), 126.3 (CH), 122.7 (CH), 96.5 (CH), 60.0 (CH_2), 51.4 (CH_2), 38.3 (CH_2), 22.4 (CH_2), 11.5 (CH_3).

MS (ESI), m/z (%): 188 (M^++1, 69), 161 (24), 129 (100). **HRMS** (ESI-TOF) m/z: $[M]^+$ Calcd for $C_{13}H_{18}N$ 188.1430; Found 188.1434.

4.2 Alkynylamine 1b·HCl

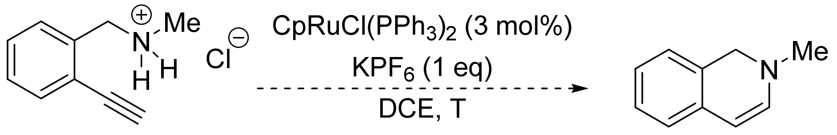

Entry	T (ºC)	t (h)	Product
1	100	2	dec.
2	37	24	CM

[a] Reaction conditions: 0.2 mmol of **1b·HCl** and 2 mL of DCE in a sealed tube.

Following *general procedure B*, in absence of oxidant, it was determined decomposition of starting material at 100 ºC and the formation of complex mixture of products that could not be identified.

[3] Álvarez-Pérez, A.; González-Rodríguez, C.; García-Yebra, C.; Varela, J. A.; Oñate, E.; Esteruelas, M. A.; Saá, C. "Catalytic Cyclization of o-Alkynyl Phenethylamines via Osmacyclopropene Intermediates: Direct Access to Dopaminergic 3-Benzazepines" *Angew. Chem. Int. Ed.* **2015**, *54*, 13357-13361.

5 Chemoselective lactamization *vs* hydroamination of alkynylamides

5.1 Ru-catalyzed oxidative lactamization of alkynyl phenylacetamide 9a: synthesis of 3-methyl-1,5-dihydro-2*H*-benzo[*d*]azepine-2,4(3*H*)-dione (10)

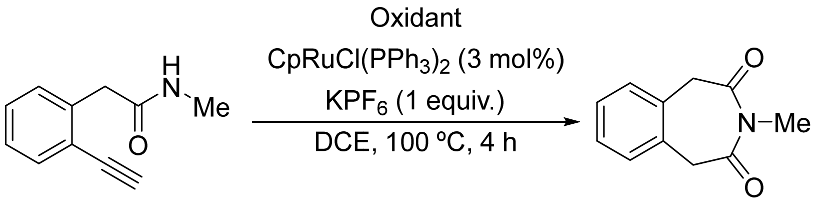

9a **10**

Entry	Oxidant	T (ºC)	t (h)	Product
1	1.1 equiv. of *N*-oxide	100	4	**10**, 73%[b]
2	---	100	24	CM

[a] Reaction conditions: 0.2 mmol of **1b·HCl** and 2 mL of DCE in a sealed tube. [b] Isolated yield.

Entry 2: following the *general procedure A*, in absence of oxidant, a complex mixture of products was obtained.

5.2 Ru-catalyzed hydroamination of alkynylbenzamide 9b. Formation of isoquinolin-1(2H)-one (11).[a]

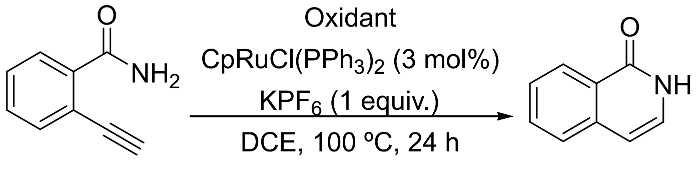

9b **11**

Entry	Oxidant	Product **11**[b]
1	1.1 equiv. of 4-Pic-*N*-oxide	48%
2	---	83%

[a] Reaction conditions: 0.4 mmol of **9b**, and 2 mL of DCE in a sealed tube. [b] Isolated yields.

Entry 2: following the *general procedure A* with some modifications in absence of oxidant: $CpRuCl(PPh_3)_2$ (0.009 g, 0.012 mmol, 0.03 equiv.), KPF_6 (0.074 g, 0.4 mmol, 1 equiv.) and amide **9b** (0.058 g, 0.4 mmol, 1 equiv.) in 2 mL of DCE was heated at 100 ºC for 24 hours. The residue was purified by flash column chromatography in silica gel (EtOAc/Hex 1:1) to afford the title compound **11** as brown solid (0.042 g, 83%).

^{1}H NMR (300 MHz, $CDCl_3$), δ 11.49 (s, 1H), 8.43 (d, *J* = 8.00 Hz, 1H), 7.68 (t, *J* = 6.9 Hz, 1H), 7.59-7.51 (m, 2H), 7.20 (d, *J* = 7.1 Hz, 1H), 6.58 (d, *J* = 7.1 Hz, 1H).

^{13}C{1H} NMR, DEPT (75 MHz, $CDCl_3$), δ 164.4 (C=O), 138.1 (C), 132.5 (CH), 127.6 (CH), 127.3 (CH), 126.8 (CH), 126.2 (CH), 126.1 (C), 106.7 (CH).

MS (EI) (m/z, %): 145 (M^+, 100), 118 (36), 90 (29). **HRMS** (EI-TOF) m/z: $[M]^+$ Calcd for C_9H_7NO 145.0528; Found 145.0528.

6 Preparation and data of starting materials

6.1 Synthesis of *N*-(2-ethynylphenethyl)propan-1-amine (1a) and its ammonium salt (1a·HCl)

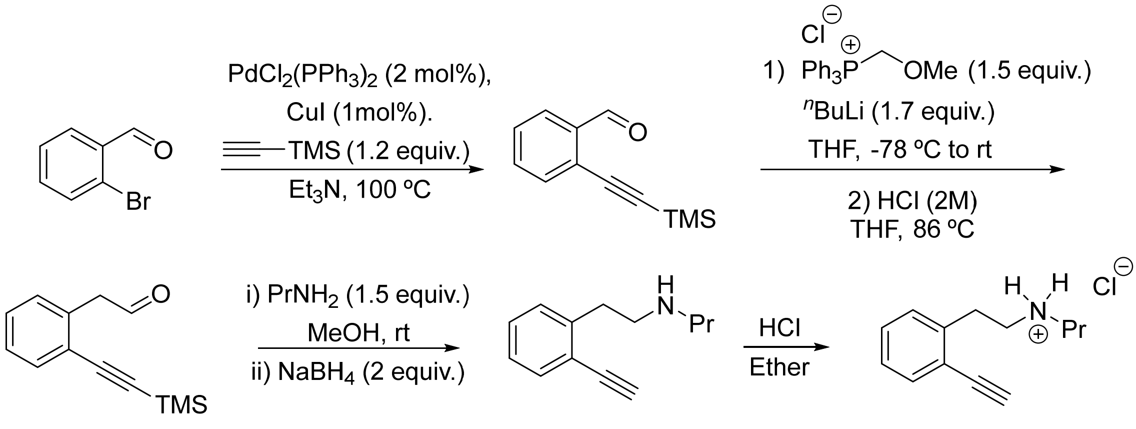

1a **1a·HCl**

The alkynylamine **1a** was prepared following previously reported synthesis.[4] Appearance: yellow oil. Overall yield: 68%.

Synthesis ammonium salt **1a·HCl**: to a solution of amine **1a** (0.720 g, 3.85 mmol) in Et_2O (3 mL) HCl (2M in Et_2O) was added until acid pH. Then, the ammonium salt formed as a white solid was filtered through a Büchner funnel to remove the solvent and the excess of acid. The solid was dried *in vacuo* (0.835 g, 98%) to afford the title compound (**1a·HCl**) pure as a white solid.

^{1}H NMR (400 MHz, $CDCl_3$) δ 9.80 (s, 2H), 7.48 (d, *J* = 7.6 Hz, 1H), 7.34 (d, *J* = 7.6 Hz, 1H), 7.33 – 7.24 (m, 1H), 7.22 (t, *J* = 7.6 Hz, 1H), 3.44 (dd, *J* = 11.0, 6.2 Hz, 2H), 3.39 (s, 1H), 3.28 – 3.19 (m, 2H), 3.01 – 2.91 (m, 2H), 1.97 (sxt, *J* = 7.5 Hz, 2H), 1.02 (t, *J* = 7.5 Hz, 3H).

^{13}C{1H} NMR, DEPT (101 MHz, $CDCl_3$) δ 139.2 (C), 133.1 (CH), 129.7 (CH), 129.5 (CH), 127.3 (CH), 121.9 (C), 82.3 (CH), 81.4 (C), 49.2 (CH_2), 47.4 (CH_2), 31.0 (CH_2), 19.6 (CH_2), 11.4 (CH_3).

---

[4] Álvarez-Pérez, A.; González-Rodríguez, C.; García-Yebra, C.; Varela, J. A.; Oñate, E.; Esteruelas, M. A.; Saá, C. "Catalytic Cyclization of o-Alkynyl Phenethylamines via Osmacyclopropene Intermediates: Direct Access to Dopaminergic 3-Benzazepines" *Angew. Chem. Int. Ed.* **2015**, *54*, 13357-13361.

MS (CI), m/z (%): 188 (M+1, 100). **HRMS** (CI-TOF) m/z: $[M + H]^+$ Calcd for $C_{13}H_{18}N$ 188.1434 Found 188.1432. **Mp:** 148-150 °C.

6.2 Synthesis of *N*-methyl-1-(2-(prop-2-yn-1-yl)phenyl)methanamine (3a)[5] and its ammonium salt (3a·HCl)

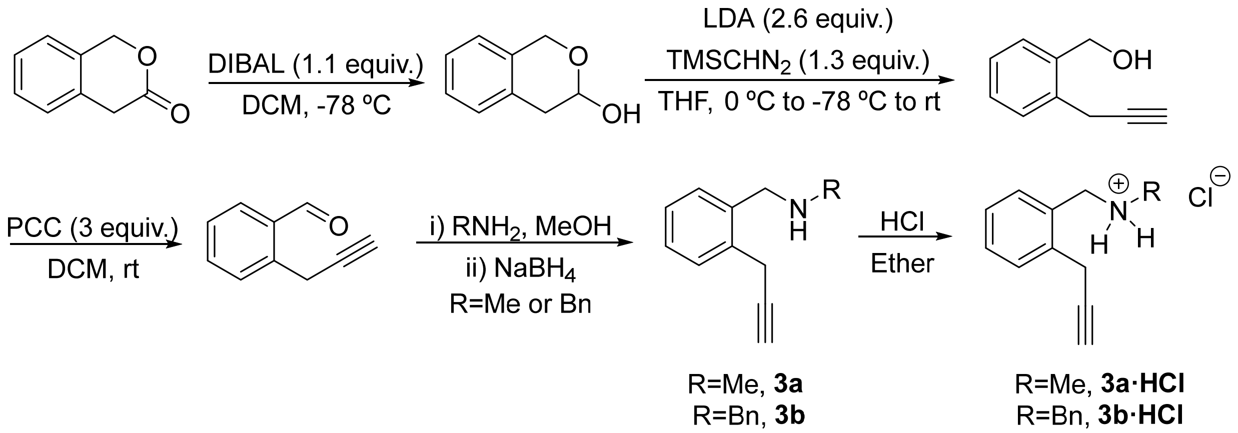

Step 1

To a solution of 3-isochromanone (1.5 g, 10.12 mmol) in DCM (30 mL) diisobutylaluminum hydride (1.0 M solution in hexanes, 11.1 mL, 11.13 mmol, 1.1 equiv.) was added at -78 °C. After stirring at -78 °C for 2.5 h, the reaction mixture was allowed to warm to room temperature and was quenched by addition of HCl (2M, 5 mL). The resulting white suspension was filtered over silica and the filtrate was extracted with ether (2 x 20 mL), dried (Na_2SO_4) and concentrated *in vacuo*. The 3-isochromanol was used in the following step without further purification. **^{1}H NMR** (300 MHz, $CDCl_3$) δ 7.22 – 7.16 (m, 2H), 7.16 – 7.10 (m, 1H), 7.05 – 6.99 (m, 1H), 5.38 (t, J = 4.5 Hz, 1H), 5.04 – 4.74 (m, 2H), 3.14 – 3.04 (m, 1H), 2.93 (bs, 1H), 2.88 – 2.78 (m, 1H).

Step 2

To a solution of diisopropylamine (3.5 mL, 24.9 mmol, 2.6 equiv.) in THF (40 mL) ^{n}BuLi (2.5 M in hexanes, 10 mL, 24.9 mmol, 2.6 equiv.) was added dropwise at 0 °C. The resulting solution was stirred for 15 min before cooling to -78 ºC. Then, a solution of trimethylsilyldiazomethane (2 M in ether, 6.3 mL, 12.47 mmol, 1.3 equiv.) was added dropwise. Upon the completion of the addition, the color of solution turned into orange and stirring was continued for additional 30 min at -78 °C. To this solution, 3-isochromanol (1.44 g, 9.6 mmol) was added in THF (6 mL) at room temperature. Once the reaction finished, saturated aqueous solution of NH_4Cl (20 mL) was added to quench the

---

[5] This reference was followed with some modifications: Chang, S.; Lee, M.; Jung, D. Y.; Yoo, E. J.; Cho, S. H.; Han, S. K. Catalytic One-Pot Synthesis of Cyclic Amidines by Virtue of Tandem Reactions Involving Intramolecular Hydroamination under Mild Conditions. *J. Am. Chem. Soc.* **2006**, *128*, 12366-12367.

reaction and diethyl ether (50 mL) was added to dilute the reaction mixture. The organic layers were separated and the resulting aqueous layer was further extracted with diethyl ether (2 x 20 mL). The combined organic layers were dried (Na_2SO_4), filtered and evaporated to provide the crude alcohol. The residue was purified by flash column chromatography in silica gel (EtOAc/Hex 4:6) to afford the title compound as a colorless oil (1.17 g, 84% in two steps). **^{1}H NMR** (300 MHz, $CDCl_3$) δ 7.53 – 7.45 (m, 1H), 7.41 – 7.36 (m, 1H), 7.30 (m, 2H), 4.72 (s, 2H), 3.65 (d, *J* = 2.7, 2H), 2.17 (t, *J* = 2.7 1H), 1.75 (bs, 1H).

Step 3

To a solution of the above obtained benzyl alcohol derivative (1.16 g, 7.98 mmol) in DCM (50 mL) a mixture of PCC (5.16 g, 23.94 mmol, 3 equiv.) and celite® (4 g) were added in one portion and the resulting brown slurry was stirred at room temperature. Once the reaction finished, the resulting slurry was filtered through a pad of silica gel. The solvent was concentrated *in vacuo* and the resulting residue was purified by flash column chromatography in silica gel (EtOAc/Hex 1:9) to afford the title compound as a yellow oil (1.13 g, 99%). **^{1}H NMR** (300 MHz, $CDCl_3$) δ 10.20 (s, 1H), 7.84 – 7.71 (m, 2H), 7.65 – 7.53 (m, 1H), 7.50 – 7.43 (m, 1H), 4.09 (d, *J* = 2.7 Hz, 2H), 2.25 (t, *J* = 2.7 Hz, 1H).

Step 4[6]

To a solution of methylamine (2 M in MeOH, 6 mL, 11 mmol, 3 equiv.), $Ti(O^iPr)_4$ (1.5 mL, 5.15 mmol, 1.3 equiv.) and the aldehyde (0.57 g, 3.96 mmol, 1 equiv.) were added at room temperature. After 4 hours $NaBH_4$ (0.18 g, 4.75 mmol, 1.2 equiv.) was added portionwise. After 2 hours, water (1 mL) and diethyl ether (20 mL) were added and the mixture was filtered over silica. The above filtrate was washed with saturated solution of NaCl (10 mL) and extracted with ether (2 x 10 mL). The combined organic layers were dried (Na_2SO_4) and concentrated *in vacuo*. The residue was purified by flash column chromatography in silica gel (DCM/MeOH 9:1) to afford the title compound (**3a**) as a yellow oil (0.360 g, 57%).

Step 5

To a solution of amine **3a** (0.21 g, 1.32 mmol) in Et_2O (3 mL) HCl (2 M in Et_2O) was added until acid pH. Then, the ammonium salt formed as a white solid, was filtered through a Büchner funnel to remove the solvent and the excess of acid. The solid was dried *in vacuo* (0.25 g, 96%) to afford the title compound (**3a·HCl)** pure.

[6] Kumpaty, H. J.; Williamson, J. S.; Bhattacharyya, S. Synthesis of N-methyl secondary amines. *Synth. Commun.* **2003**, *33*, 1411-1416.

^{1}H NMR (400 MHz, DMSO-d_6) δ 9.41 (bs, 2H), 7.56 (dd, *J* = 7.6, 1.5 Hz, 1H), 7.50 (dd, *J* = 7.6, 1.5 Hz, 1H), 7.36 – 7.32 (m, 2H), 4.11 (s, 2H), 3.85 (d, *J* = 2.7 Hz, 2H), 3.13 (t, *J* = 2.7 Hz, 1H), 2.55 (s, 3H).

^{13}C{1H} NMR, DEPT (101 MHz, DMSO-d_6) δ 135.8 (C), 130.8 (CH), 130.2 (C), 129.2 (CH), 128.7 (CH), 127.0 (CH), 81.6 (C), 74.20 (CH), 47.8 (CH_2), 32.4 (CH_3), 21.9 (CH).

Mp: 128-131 °C.

6.3 Synthesis of *N*-benzyl-1-(2-(prop-2-yn-1-yl)phenyl)methanamine (3b)[5] and its ammonium salt (3b·HCl)

Step 4

To a solution of aldehyde (0.478 g, 3.32 mmol, 1 equiv.) in MeOH (13 mL) benzylamine (0.4 mL, 3.65 mmol, 1.1 equiv.) was added the at room temperature. After 4 hours $NaBH_4$ (0.19 g, 4.98 mmol, 1.5 equiv.) was added portionwise. After 2 additional hours, the solvents were removed *in vacuo*. The residue was redissolved in DCM (10 mL) and water (10 mL) was added. The mixture was extracted with more DCM (3 x 10 mL) and the combined organic layers were dried (Na_2SO_4) and concentrated *in vacuo*. The residue was purified by flash column chromatography in silica gel (EtOAc/Hex 3:7) to afford the title compound (**3b**) as a yellow oil (0.389 g, 50%). Spectroscopic data agree with those reported previously.[5]

Step 5

To a solution of amine **3b** (0.214 g, 0.91 mmol) in Et_2O (3 mL) HCl (2 M in Et_2O) was added until acid pH. Then, the ammonium salt formed as a white solid, was filtered through a Büchner funnel to remove the solvent and the excess of acid. The solid was dried *in vacuo* (0.243 g, 98%).

^{1}H NMR (300 MHz, $CDCl_3$) δ 10.19 (bs, 1H), 7.72 – 7.62 (m, 1H), 7.51 - 7.49 (m, 2H), 7.44 – 7.27 (m, 5H), 7.30 – 7.10 (m, 2H), 3.98 – 3.82 (m, 4H), 3.42 (d, *J* = 2.7 Hz, 2H), 2.06 (t, *J* = 2.7 Hz, 1H).

^{13}C{1H} NMR, DEPT (75 MHz, $CDCl_3$) δ 136.0 (C), 131.5 (CH), 130.4 (2xCH), 130.1 (C), 129.8 (CH), 129.5 (CH), 129.3 (2xCH), 128.6 (C), 127.9 (CH), 81.1 (C), 71.5 (CH), 49.5 (CH_2), 45.0 (CH_2), 23.0 (CH_2).

Mp: 145-147 °C.

6.4 Synthesis of 2-(but-3-yn-1-yl)aniline (5a)

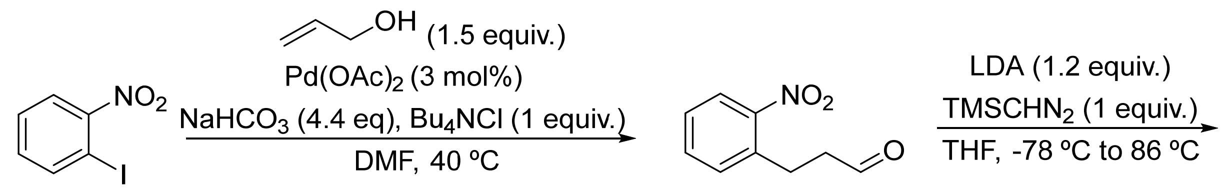

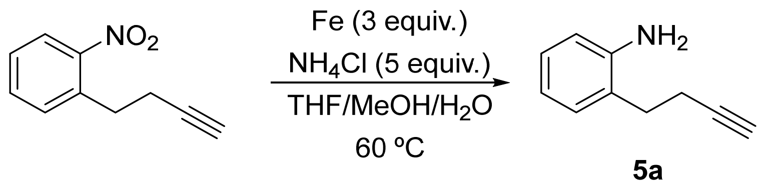

5a

Step 1[7]

To a suspension of 1-iodo-2-nitrobenzene (3.5 g, 14 mmol), Pd(OAc)2 (0.094 g, 0.42 mmol,0.03 equiv.), tetrabutylammonium chloride (3.89 g, 14 mmol, 1 equiv.) and sodium bicarbonate (5.18 g, 61.6 mmol, 4.4 equiv.) in DMF (0.5 M) allyl alcohol (1.4 mL, 21 mmol, 1.5 equiv.)was added and the mixture was heated at 40 °C. Once the reaction finished, the mixture was filtered over a pad of silica and the above filtrate was washed with water (3 x 20 mL). The organic phase was dried (Na_2SO_4) and concentrated *in vacuo*. The residue was purified by flash column chromatography in silica gel (EtOAc/Hex 3:7) to afford the title compound as a yellow oil (2.28 g, 92%). **^{1}H NMR** (300 MHz, $CDCl_3$) δ 9.83 (t, J = 1.0 Hz, 1H), 8.03 – 7.89 (m, 1H), 7.54 (ddd, J = 8.3, 6.2, 1.4 Hz, 1H), 7.45 – 7.31 (m, 2H), 3.21 (t, J = 7.4 Hz, 2H), 2.90 (t, J = 7.4 Hz, 2H).

Step 2

To a solution of diisopropylamine (2.14 mL, 15.28 mmol, 1.2 equiv.) in THF (66 mL) ^{n}BuLi (2.5 M in hexanes, 6.1 mL, 15.28 mmol, 2.6 equiv.) was added dropwise at -78 °C. The resulting solution was stirred for 15 min before warm to 0 °C and stirring was continued for other 15 minutes. Then, a solution of trimethylsilyldiazomethane (2 M in ether, 6.4 mL, 12.73 mmol, 1 equiv.) was added dropwise at -78 °C. Upon completion of the addition, the stirring was continued for additional 30 min at -78 °C. To this solution, the aldehyde (1.44 g, 9.6 mmol) was added in THF (13 mL) at room temperature. After 1 hour, the mixture was heated at 86 °C for 3 hours. Once the reaction finished, saturated aqueous solution of NH_4Cl (20 mL) was added to quench the reaction and Et_2O (50 mL) was added to dilute the reaction mixture. The organic layers were separated and the resulting aqueous layer was further extracted with Et_2O (2 x 20 mL). The combined organic layers were dried (Na_2SO_4), filtered and evaporated to provide the crude alcohol. The residue was purified by flash column

[7] Schuster, C. H.; Coombs, J. R.; Kasun, Z. A.; Morken, J. P. Enantioselective Carbocycle Formation through Intramolecular Pd-Catalyzed Allyl–Aryl Cross-Coupling. *Org. Lett.* **2014**, *16*, 4420-4423.

chromatography in silica gel (EtOAc/Hex 1:9) to afford the title compound as a colorless oil (1.97 g, 88%). ^{1}**H NMR** (300 MHz, $CDCl_3$) δ 7.95 (d, *J* = 8.2 Hz, 1H), 7.56 (t, *J* = 7.5 Hz, 1H), 7.49 – 7.35 (m, 2H), 3.13 (t, *J* = 7.1 Hz, 2H), 2.61 (td, *J* = 7.1, 2.5 Hz, 2H), 1.99 (d, *J* = 2.5 Hz, 1H).

Step 3

To a suspension of Fe (0.45 g, 8.01 mmol, 3 equiv.) and NH_4Cl (0.71 g, 13.35 mmol, 5 equiv.) in a mixture of THF/MeOH/water (14 mL, 6:3:1) nitro compound (0.47 g, 2.68 mmol) was added and the mixture was heated at 60 °C for 24 hours. Then, the mixture was filtered over a pad of silica. The above filtrate was then washed with saturated solution of NaCl (10 mL) and extracted with EtOAc (2 x 10 mL). The combined organic layers were dried (Na_2SO_4), filtered and concentrated *in vacuo*. The residue was purified by flash column chromatography in silica gel (EtOAc/Hex 1:9) to afford the title compound (**5a**) as an orange solid (1.75 g, 83%).

^{1}**H NMR** (500 MHz, $CDCl_3$) δ 7.11 – 7.05 (m, 2H), 6.78 (td, *J* = 7.4, 1.3 Hz, 1H), 6.72 – 6.69 (m, 1H), 3.69 (bs, 1H), 2.78 (t, *J* = 7.6 Hz, 2H), 2.53 (td, *J* = 7.6, 2.6 Hz, 2H), 2.04 (t, *J* = 2.6 Hz, 1H).

^{13}**C{1H} NMR, DEPT** (126 MHz, $CDCl_3$) δ 144.3 (C), 129.6 (CH), 127.7 (CH), 124.9 (CH), 119.0 (CH), 116.0 (C), 84.2 (C), 69.1 (CH), 30.6 (CH_2), 18.4 (CH_2). **MS (CI)**, m/z (%): 146 (M+1, 100).

HRMS (CI-TOF) m/z: $[M + H]^+$ Calcd for $C_{10}H_{12}N$ 146.0964 Found 146.0959.

Mp: 55-58 °C.

6.5 Synthesis 2-(but-3-yn-1-yl)-N-methylaniline (5b)

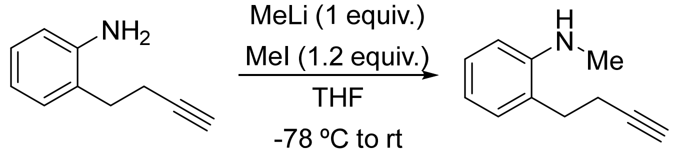

5b

Step 1[8]

To a solution of aniline **5a** (0.312 g, 2.15 mmol) in THF (7 mL) MeLi (1.6 M en Et_2O, 1.3 mL, 2.15 mmol, 1 equiv.) was slowly added at -78 °C. After stirring at room temperature for 5 min, the reaction mixture was cooled to –78 °C and MeI (0.16 mL, 2.58 mmol, 1.2 equiv.) was added dropwise. Then, the mixture was gradually warmed to room temperature for 12 hours. The reaction mixture was quenched with saturated solution of NH_4Cl (10 mL) and extracted with EtOAc (3 x 15 mL). The combined organic layers were washed with saturated solution of NaCl (10 mL), dried (Na_2SO_4), and

[8] Taguchi, M.; Tokimizu, Y.; Oishi, S.; Fujii, N.; Ohno, H. Synthesis of Fused Carbazoles by Gold-Catalyzed Tricyclization of Conjugated Diynes via Rearrangement of an N-Propargyl Group. *Org. Lett.* **2015**, *17*, 6250-6253.

concentrated *in vacuo*. The residue was purified by flash column chromatography in silica gel (EtOAc/Hex 1:9) to afford the title compound (**5b**) as a yellow oil (0.342 g, 74%).

^{1}H NMR (400 MHz, $CDCl_3$) δ 7.25 – 7.17 (m, 1H), 7.09 (dd, *J* = 7.4, 1.5 Hz, 1H), 6.77 – 6.70 (m, 1H), 6.67 (d, *J* = 8.1 Hz, 1H), 3.76 (bs, 1H), 2.89 (s, 3H), 2.75 (t, *J* = 7.7 Hz, 2H), 2.55 – 2.49 (m, 2H), 2.04 (t, *J* = 2.5 Hz, 1H).

^{13}C{1H} NMR, DEPT (101 MHz, $CDCl_3$) δ 146.9 (C), 129.1 (CH), 128.0 (CH), 124.4 (C), 117.2 (CH), 110.1 (CH), 84.2 (C), 69.1 (CH), 30.9 (CH_3), 30.5 (CH_2), 18.2 (CH).

MS (CI), m/z (%): 160 (M+1, 100), 158 (7). **HRMS** (CI-TOF) m/z: $[M + H]^+$ Calcd for $C_{11}H_{14}N$ 160.1121; Found 160.1122.

6.6 Synthesis of 2-(prop-2-yn-1-yloxy)aniline (5c)

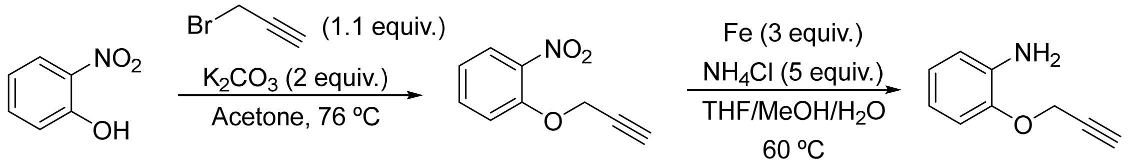

5c

Step 1

To a suspension of 2-nitrophenol (2 g, 14.38 mmol) and K_2CO_3 (3.97 g, 28.76 mmol, 2 equiv.) in acetone (20 mL) propargyl bromide (80% in toluene, 1.7 mL, 15.82 mmol, 1.1 equiv.) was added and the mixture was heated at 76 °C for 7 hours. Once finished, the mixture was allowed to cool to room temperature and saturated solution of NaCl (10 mL) was added and extracted with ether (2 x 10 mL). The combined organic layers were dried (Na_2SO_4) and concentrated *in vacuo*. The residue was used in the next step without further purifications. **^{1}H NMR** (300 MHz, $CDCl_3$) δ 7.86 (dd, *J* = 8.1, 1.8 Hz, 1H), 7.56 (td, *J* = 7.8, 1.8 Hz, 1H), 7.31 – 7.22 (m, 1H), 7.10 (dd, *J* = 8.1, 1.2 Hz, 1H), 4.85 (d, *J* = 2.4 Hz, 2H), 2.58 (t, *J* = 2.4 Hz, 1H).

Step 2

To a suspension of Fe (2.4 g, 43.14 mmol, 3 equiv.) and NH_4Cl (3.84 g, 71.9 mmol, 5 equiv.) in a mixture of THF/MeOH/water (70 mL, 6:3:1) nitro compound (14.38 mmol) was added and the mixture was heated at 60 °C for 12 hours. Then, the mixture was filtered over a pad of silica. The above filtrate was then washed with saturated solution of NaCl (30 mL) and extracted with EtOAc (2 x 20 mL). The combined organic layers were dried (Na_2SO_4), filtered and concentrated *in vacuo*. The residue was purified by flash column chromatography in silica gel (EtOAc/Hex 3:7) to afford the title

compound (**5c**) as a yellow oil (1.75 g, 83%). Spectroscopic data agree with those reported previously.[9]

^{1}H NMR (500 MHz, $CDCl_3$) δ 6.92 (dd, *J* = 7.9, 1.4 Hz, 1H), 6.84 (ddd, *J* = 7.3, 1.4 Hz, 1H), 6.76 – 6.69 (m, 2H), 4.72 (d, *J* = 2.4 Hz, 2H), 3.82 (bs, 2H), 2.52 (t, *J* = 2.4 Hz, 1H).

^{13}C{1H} NMR, DEPT (126 MHz, $CDCl_3$) δ 145.4 (C), 136.8 (C), 122.4 (CH), 118.5 (CH), 115.6 (CH), 112.8 (CH), 79.0 (C), 75.5 (CH), 56.5 (CH_2).

MS (CI), m/z (%): 148 (M+1, 100).

6.7 Synthesis of *N*-methyl-2-(prop-2-yn-1-yloxy)aniline (5d)

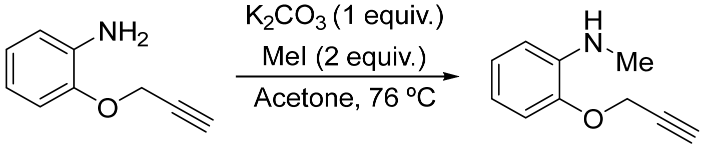

5d

Step 1[10]

To a suspension of aniline **5c** (1.75 g, 11.9 mmol) and K_2CO_3 (1.64 g, 11.9 mmol, 1 equiv.) in acetone (40 mL) MeI (1.5 mL, 23.8 mmol, 2 equiv.) was added and the mixture was heated at 76 °C for 24 hours. Once the reaction finished, was quenched with water (20 mL) and extracted with $CHCl_3$ (3 x 20 mL). The combined organic layers were washed with saturated solution of NaCl (20 mL) dried (Na_2SO_4) and concentrated *in vacuo*. The residue was purified by flash column chromatography in silica gel (EtOAc/Hex 2:8) to afford the title compound (**5d**) as a yellow oil (1.2 g, 61%). Spectroscopic data agree with those reported previously.[11]

^{1}H NMR (300 MHz, $CDCl_3$) δ 7.01 (td, *J* = 7.6, 1.4 Hz, 1H), 6.93 (dd, *J* = 8.0, 1.4 Hz, 1H), 6.77 – 6.65 (m, 2H), 4.74 (d, *J* = 2.4 Hz, 2H), 4.32 (bs, 1H), 2.90 (s, 3H) 2.57 (t, *J* = 2.4 Hz, 1H).

^{13}C{1H} NMR (75 MHz, $CDCl_3$) δ 144.9 (C), 139.8 (C), 122.5 (CH), 116.1 (CH), 111.3 (CH), 109.8 (CH), 78.8 (C), 75.5 (CH), 56.3 (CH_2), 30.3 (CH_3).

MS (CI), m/z (%): 162 (M+1, 100). **HRMS** (CI-TOF) m/z: $[M + H]^+$ Calcd for $C_{10}H_{12}NO$ 162.0913; Found 162.0913.

---

[9] Zhou, Y.-G.; Yang, P.-Y.; Han, X.-W. Synthesis and Highly Enantioselective Hydrogenation of Exocyclic Enamides: (*Z*)-3-Arylidene-4-acetyl-3,4-dihydro-2H- 1,4-benzoxazines. *J. Org. Chem.* **2005**, *70*, 1679-1683.

[10] Kundu, N. G.; Nandi, B. Depropargylation under palladium–copper catalysis: synthesis of diaryl sulfides. *Tetrahedron* **2001**, *57*, 5885-5895.

[11] Padín, D.; Varela, J. A.; Saá, C. Ruthenium-Catalyzed Tandem Carbene/Alkyne Metathesis/N–H Insertion: Synthesis of Benzofused Six-Membered Azaheterocycles. *Org. Lett.* **2020**, *22*, 2621-2625.

6.8 Synthesis of *N*-benzyl-2-(prop-2-yn-1-yloxy)aniline (5e)

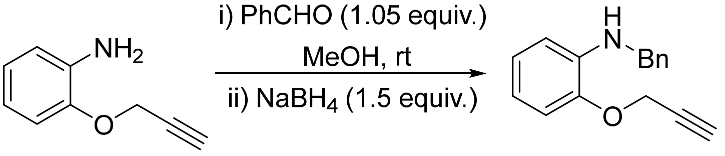

5e

To a solution of aniline **5c** (0.308 g, 2.09 mmol) in MeOH (8 mL) benzaldehyde (0.22 mL, 2.19 mmol, 1.05 equiv.)was added at room temperature. After 4 hours $NaBH_4$ (0.12 g, 3.14 mmol, 1.5 equiv.) was added portion wise. After 2 additional hours, the solvents were removed *in vacuo*. The residue was redissolved in DCM (10 mL) and water (10 mL) was added. The mixture was extracted with more DCM (3 x 10 mL) and the combined organic layers were dried (Na_2SO_4) and concentrated *in vacuo*. The residue was purified by flash column chromatography in silica gel (EtOAc/Hex 2:8) to afford the title compound (**5e**) as a yellow solid (0.343, 69%).

^{1}H NMR (400 MHz, $CDCl_3$) δ 7.42 – 7.21 (m, 5H), 6.95 – 6.85 (m, 3H), 6.67 (td, *J* = 7.7, 1.5 Hz, 1H), 6.61 (d, *J* = 8.2 Hz, 1H), 4.72 (d, *J* = 2.4 Hz, 2H), 4.67 (bs, 1H), 4.37 (s, 2H), 2.51 (t, *J* = 2.4 Hz, 1H).

^{13}C{1H} NMR, DEPT (75 MHz, $CDCl_3$) δ 144.8 (C), 139.5 (C), 138.5 (C), 128.6 (2xCH), 127.4 (2xCH), 127.1 (CH), 122.4 (CH), 116.4 (CH), 111.6 (CH), 110.6 (CH), 78.9 (C), 75.6 (CH), 56.3 (CH_2), 47.8 (CH_2).

MS (CI), m/z (%): 238 (M+1, 100), 198 (6). **HRMS** (CI-TOF) m/z: $[M + H]^+$ Calcd for $C_{16}H_{16}NO$ 238.1226; Found 238.1221. **Mp:** 56-59 °C.

6.9 Synthesis of 2-(prop-2-yn-1-ylthio)aniline (5f)[12]

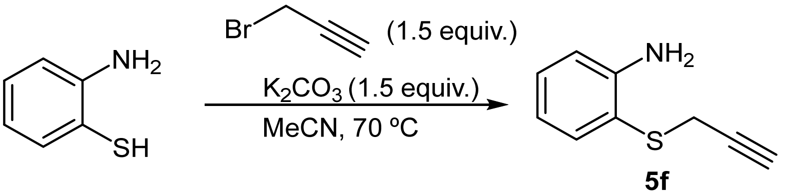

5f

To a suspension of 2-aminobenzenethiol (20 mmol) and K_2CO_3 (30 mmol, 1.5 equiv.) in acetone (80 mL) propargyl bromide (80% in toluene, 1.5 equiv.) was added and the mixture was heated at 76 °C until complete consumption of starting material. Then, the mixture was allowed to cool to room

[12] We would like to thank Damián Padín Santos, PhD, for the generous gift of this substrate for the catalytic reaction attempt. See: (a) Catalytic ruthenium vinyl carbenes. Properties, reactivity and applications in tandem reactions. PhD dissertation. Universidade de Santiago de Compostela, 2019. (b) Padín, D.; Varela, J. A.; Saá, C. Ruthenium-Catalyzed Tandem Carbene/Alkyne Metathesis/N–H Insertion: Synthesis of Benzofused Six-Membered Azaheterocycles. *Org. Lett.* **2020**, *22*, 2621-2625.

temperature and was quenched with aqueous solution of HCl (5%) and diluted with water. The aqueous layer was extracted with EtOAc (3 x 20 mL) and the combined organic layers were washed with brine, dried (Na_2SO_4) and concentrated *in vacuo*. The residue was purified by flash column chromatography in silica gel (EtOAc/Hex 2:8) to afford the title compound (**5e**) as a yellow oil (2.44, 74%).

^{1}H NMR (300 MHz, $CDCl_3$) δ 7.48 (d, *J* = 7.7 Hz, 1H), 7.17 (t, *J* = 7.7, 1H), 6.80 – 6.67 (m, 2H), 4.53 (bs, 2H), 3.45 (d, *J* = 2.7 Hz, 2H), 2.23 (t, *J* = 2.6 Hz, 1H).

^{13}C{1H} NMR, DEPT (75 MHz, $CDCl_3$) δ 148.7 (C), 136.8 (CH), 130.8 (CH), 118.6 (CH), 116.5 (C), 115.1 (CH), 80.2 (C), 71.7 (CH), 22.9 (CH_2).

MS (CI), m/z (%): 164 (M+1, 100), 124 (22), 94 (14). **HRMS** (CI-TOF) m/z: $[M + H]^+$ Calcd for $C_9H_{10}NS$ 164.0528; Found 164.0528.

6.10 Synthesis of 1-(2-ethynylphenyl)-*N*-methylmethanamine ammonium salt (1b·HCl)

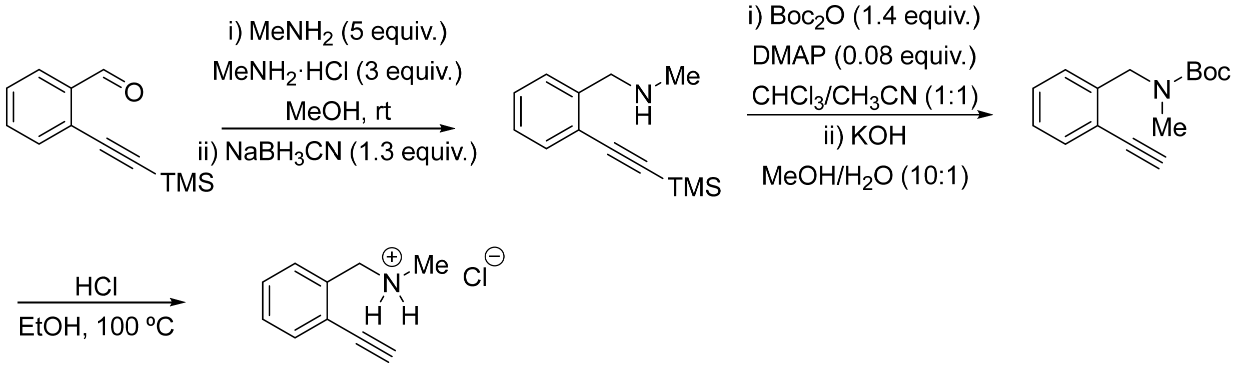

1b·HCl

The 1,5-alkynylamine ammonium salt was synthesized in accordance with the procedure reported in literature.[13] Compound **1b·HCl**, 0.350 g (40% overall yield), off-white solid.

^{1}H NMR (500 MHz, $CDCl_3$) δ 9.69 (bs, 2H), 7.78 (d, *J* = 7.7 Hz, 1H), 7.46 (d, *J* = 7.7 Hz, 1H), 7.36 (t, *J* = 7.7 Hz, 1H), 7.27 (t, *J* = 7.7 Hz, 1H), 4.31 (s, 2H), 3.42 (s, 1H), 2.53 (s, 3H).

^{13}C{1H} NMR, DEPT (126 MHz, $CDCl_3$) δ 133.0 (CH), 132.1 (C), 130.6 (CH), 129.8 (CH), 129.3 (CH), 123.2 (C), 83.7 (C), 80.6 (CH), 49.1 (CH_2), 31.3 (CH_3).

6.11 Synthesis of 3-(2-ethynylphenyl)-N-propylpropan-1-amine ammonium salt (1c·HCl)

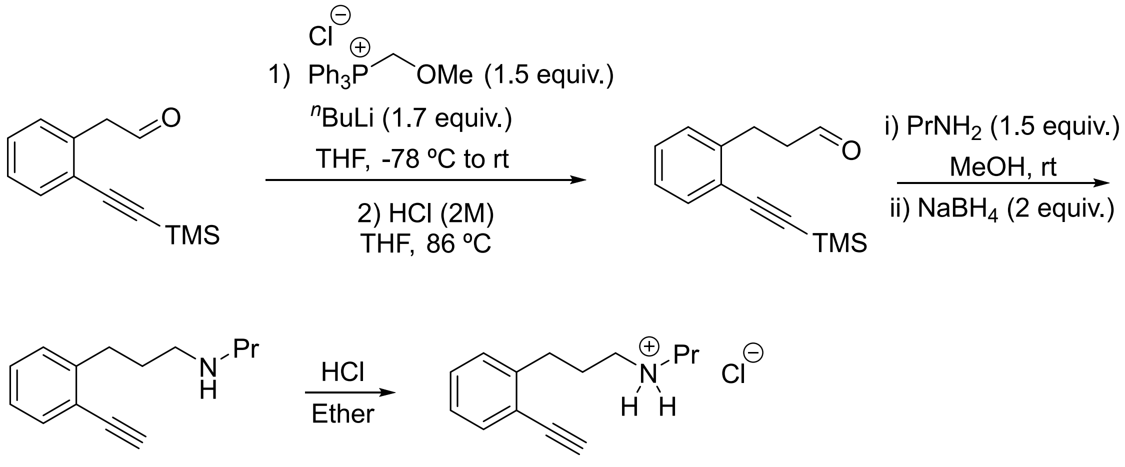

1c·HCl

Step 1

[13] Lu, X.; Masson, E. Silver-Promoted Desilylation Catalyzed by Ortho- and Allosteric Cucurbiturils. *Org. Lett.* **2010**, *12*, 2310-2313.

To a suspension of phosphonium salt (6.43 g, 18.75 mmol, 1.5 equiv.) in THF (75 mL) at -78 °C ^{n}BuLi (2.5 M in hexanes, 8.5 mL, 21.25 mmol, 1.7 equiv.) was added. The mixture was allowed to warm to room temperature over 30 min and then the aldehyde (2.7 g, 12.5 mmol, 1 equiv.) was added in THF (30 mL). Once the starting material disappear, standard workup was performed with saturated solution of NaCl (100 mL) and extraction with EtOAc (3 x 50 mL). The combined organic phases were dried (Na_2SO_4) and concentrated *in vacuo* to afford the crude product. Then, the vinyl ether was dissolved in THF (30 mL, 0.6 M) and an aqueous solution of HCl (2 M, 15 mL, 2 equiv.) was added and the resulting mixture was heated at reflux overnight. The mixture was cooled to room temperature and saturated solution of NaCl (30 mL) was added and extracted with EtOAc (3 x 20 mL). The combined organic layers were dried (Na_2SO_4) and concentrated *in vacuo*. The residue was purified by flash column chromatography in silica gel (EtOAc/Hex 0.5:9.5) to afford the homologated aldehyde as a yellow oil (1.24 g, 44%). **^{1}H NMR** (300 MHz, $CDCl_3$) δ 9.82 (s, 1H), 7.44 (d, *J* = 7.6, 1H), 7.28 – 7.07 (m, 3H), 3.10 (t, *J* = 7.6 Hz, 2H), 2.88 – 2.75 (m, 2H) 0.25 (s, 9H).

Step 2

To a solution of aldehyde (0.422 g, 1.3 mmol, 1 equiv.) in MeOH (7 mL) at room temperature propylamine (0.23 mL, 2.75 mmol, 1.5 equiv.) was added. After 4 hours, $NaBH_4$ (0.14 g, 3.67 mmol, 2 equiv.) was added portion wise. After 2 additional hours, the solvents were removed *in vacuo*. The residue was redissolved in DCM (10 mL) and water (10 mL) was added. The mixture was extracted with more DCM (3 x 10 mL) and the combined organic layers were dried (Na_2SO_4) and concentrated *in vacuo*. The residue was purified by flash column chromatography in silica gel (EtOAc) to afford the title compound as a yellow oil (0.356 g, 96%). **^{1}H NMR** (400 MHz, $CDCl_3$) δ 7.46 (d, *J* = 7.6, 1H), 7.32 - 7.05 (m, 3H), 3.23 (s, 1H), 2.83 (t, *J* = 7.4, 2H), 2.65 (t, *J* = 7.4, 2H), 2.56 (t, *J* = 7.4, 2H), 1.84 (q, *J* = 7.4, 2H), 1.49 (sxt, *J* = 7.4, 2H), 0.91 (t, *J* = 7.4 Hz, 3 H).

Step 3

To a solution of amine (0.353 g, 1.75 mmol) in Et_2O (5 mL) HCl (2 M in Et_2O) was added until acid pH. Then, the ammonium salt formed as a white solid, was filtered through a Büchner funnel to remove the solvent and the excess of acid. The solid was dried *in vacuo* (0.392 g, 90%) to afford the title compound (**1c·HCl**) pure.

^{1}H NMR (400 MHz, $CDCl_3$) δ 9.47 (bs, 2H), 7.39 (d, *J* = 7.6 Hz, 1H), 7.26 – 7.15 (m, 2H), 7.10 (ddd, *J* = 8.5, 6.5, 2.4 Hz, 1H), 3.24 (s, 1H), 2.88 (ddd, *J* = 14.6, 6.6, 3.8 Hz, 2H), 2.84 – 2.76 (m, 4H), 2.22 (p, *J* = 7.6 Hz, 2H), 1.83 (sxt, *J* = 7.6 Hz, 2H), 0.88 (t, *J* = 7.6 Hz, 3H).

^{13}C{1H} NMR, DEPT (101 MHz, $CDCl_3$) δ 142.6 (C), 133.1 (CH), 129.2 (CH), 128.9 (CH), 126.5(CH), 121.6 (C), 82.0 (CH), 81.6 (C), 49.4 (CH_2), 47.2 (CH_2), 31.3 (CH_2), 26.5 (CH_2) ,19.5 (CH_2), 11.3 (CH_3).

MS (CI), m/z (%): 202 (M+1, 100). **HRMS** (CI-TOF) m/z: $[M + H]^+$ Calcd for $C_{14}H_{20}N$ 202.1590 Found 202.1588. **Mp:** 90-93 °C.

6.12 Synthesis of 2-(2-ethynylphenyl)-*N*-methylacetamide (9a)

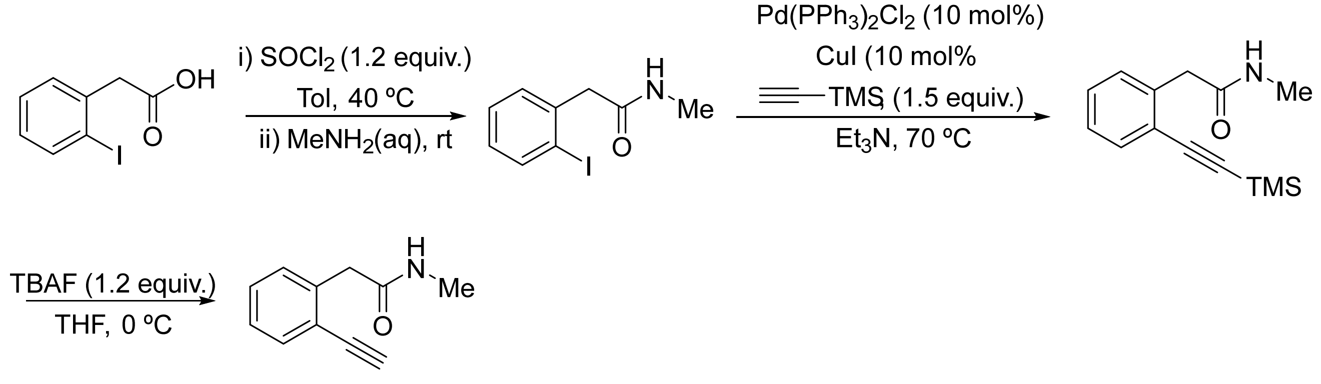

9a

The amide **9a** was prepared following previously reported synthesis.[14] Compound **9a**, 0.322g (66% overall yield), white solid.

^{1}H NMR (300 MHz, $CDCl_3$) δ 7.50 (dt, *J* = 7.5, 1.1 Hz, 1H), 7.34 – 7.30 (m, 2H), 7.23 (dt, *J* = 7.5, 4.3 Hz, 1H), 5.62 (bs, 1H), 3.73 (s, 2H), 3.31 (s, 1H), 2.73 (d, *J* = 4.8 Hz, 3H).

^{13}C{1H} NMR, DEPT (75 MHz, $CDCl_3$) δ 170.9 (CO), 137.7 (C), 133.2 (CH), 130.1 (CH), 129.6 (CH), 127.4 (CH), 122.2 (CH), 82.05 (CH), 81.8 (C), 42.3 (CH_2), 26.6 (CH_3).

MS (CI), m/z (%): 174 (M+1, 100). **Pf:** 120-123 ºC.

6.13 Synthesis of 2-ethynylbenzamide (9b)

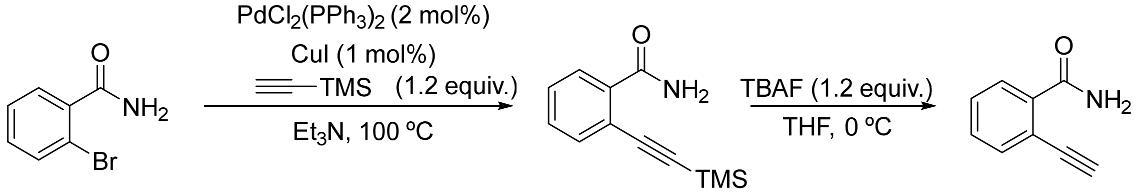

9b

The amide **9b** was prepared following previously reported synthesis.[15] Compound **9b**, 0.254g (28% overall yield), white solid.

^{1}H NMR (400 MHz, $CDCl_3$) δ 8.09-8.06 (m, 1H), 7.62-7.58 (m, 1H), 7.48-7. 44 (m, 2H), 6.44 (s, 2H), 3.53 (s, 1H).

---

[14] Wang, S.; Zhang, L.; Ding, X.; Zhou, Y.; Wang, J.; Jiang, H.; Liu, H. Synthesis of 2-Phenylnaphthalenes through Gold-Catalyzed Dimerization via a Highly Selective Carbon Nucleophile Pathway. *J. Org. Chem.* **2011**, *76*, 4514-4521.

[15] Varela-Fernández, A.; Varela, J. A.; Saá, C. Ruthenium-Catalyzed Cycloisomerization of Aromatic Homo- and Bis-Homopropargylic Amines/Amides: Formation of Indoles, Dihydroisoquinolines and Dihydroquinolines. *Adv. Synth. Catal.* **2011**, *353*, 1933-1937.

^{13}C{1H} NMR, DEPT (100 MHz, $CDCl_3$) δ 168.0 (CO), 135.4 (C), 134.2 (CH), 130.9 (CH), 130.1 (CH), 129.4 (CH), 118.9 (C), 83.9 (CH), 82.3 (C).

MS (CI), m/z (%): 146 (M+1, 100).

7 NMR spectra of starting materials

Ammonium salt of *N*-(2-ethynylphenethyl)propan-1-amine (**1a·HCl**)

^{1}H NMR (400 MHz, $CDCl_3$)

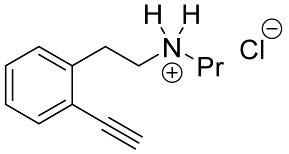

1a·HCl

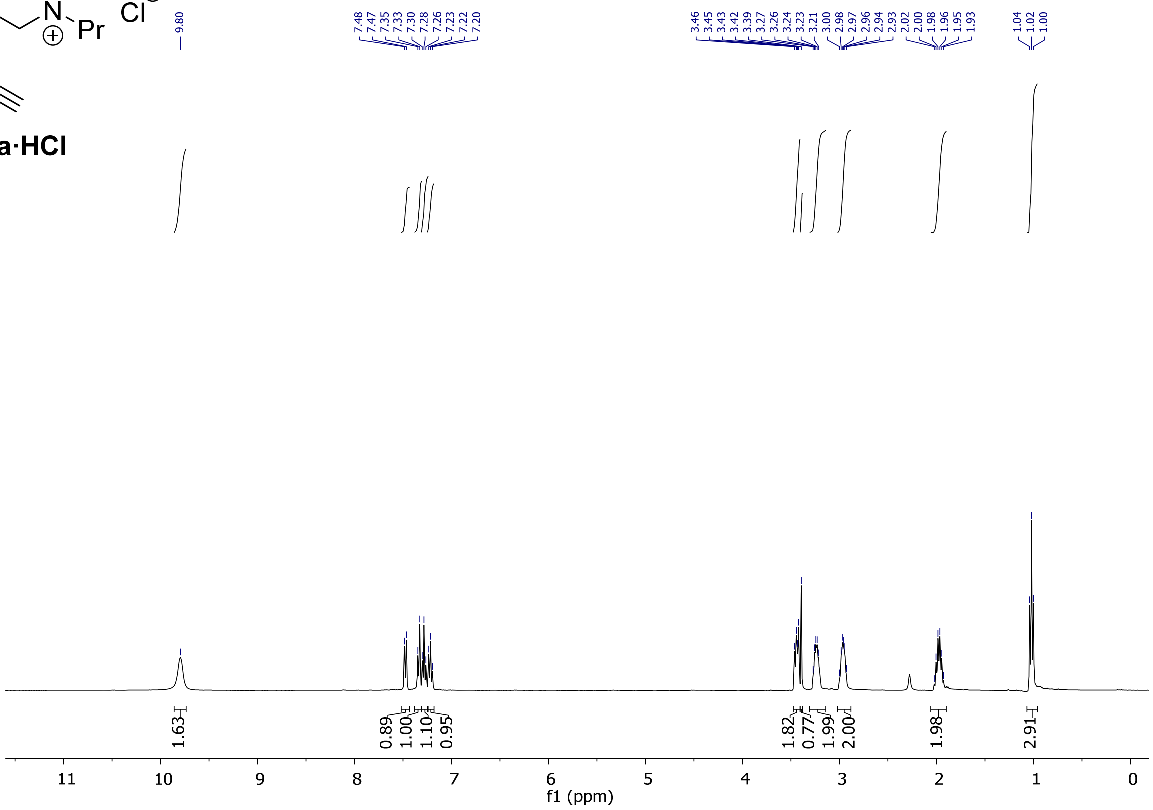

^{13}C{1H} NMR, DEPT (101 MHz, $CDCl_3$)

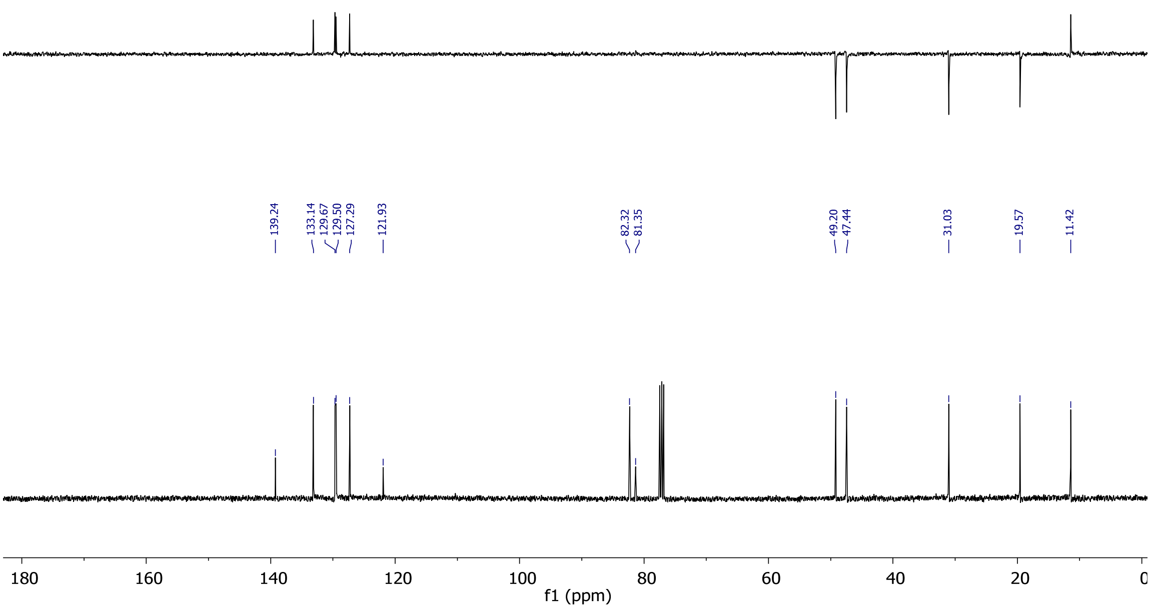

Ammonium salt of *N*-methyl-1-(2-(prop-2-yn-1-yl)phenyl)methanamine (**3a·HCl**)

^{1}H NMR (400 MHz, DMSO-d_6)

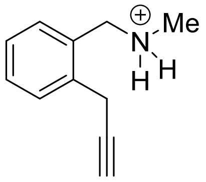

3a·HCl

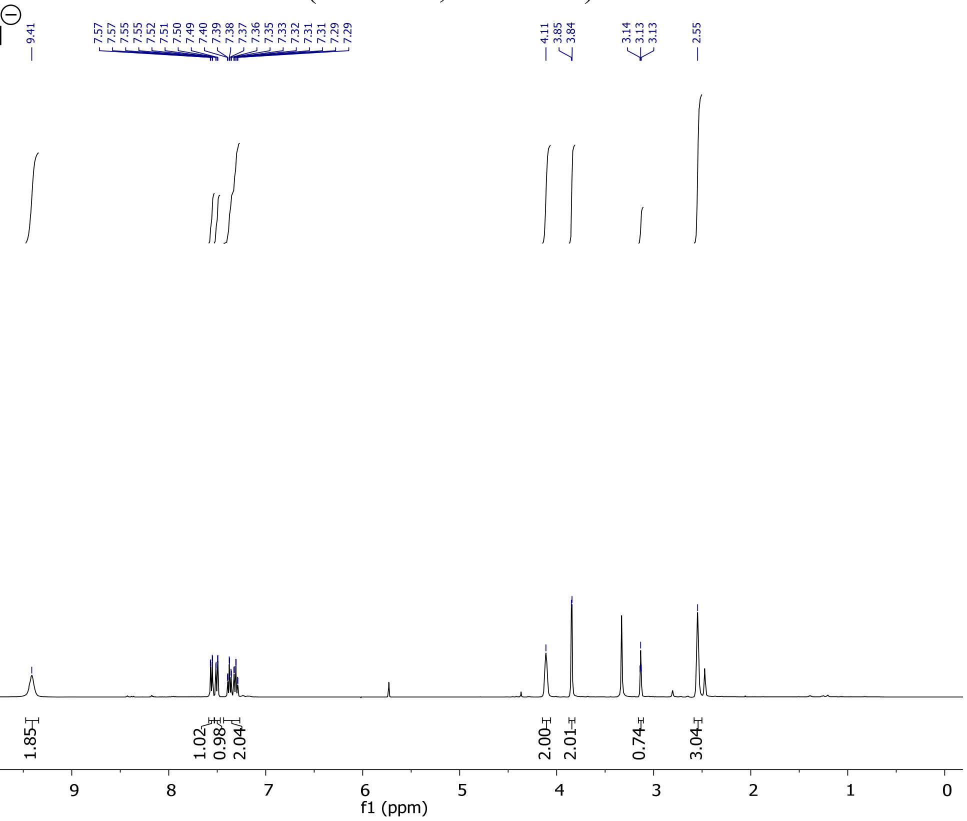

^{13}C{1H} NMR, DEPT (101 MHz, DMSO-d_6)

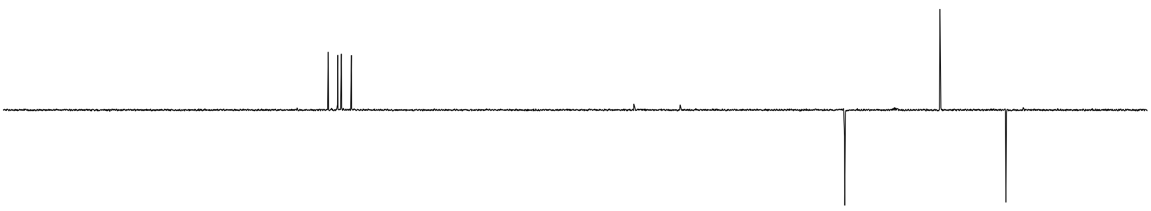

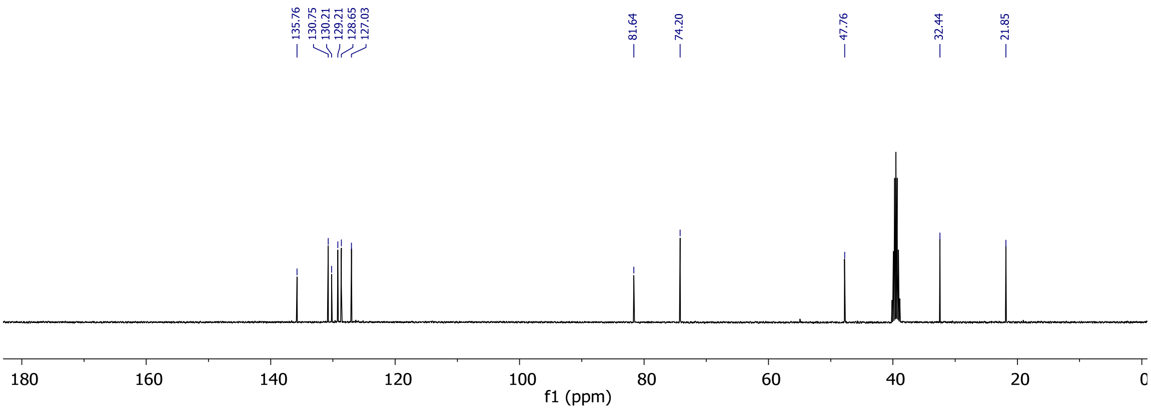

Ammonium salt of *N*-benzyl-1-(2-(prop-2-yn-1-yl)phenyl)methanamine (**3b·HCl**)

^{1}H NMR (300 MHz, $CDCl_3$)

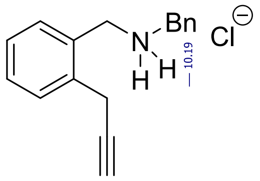

3b·HCl

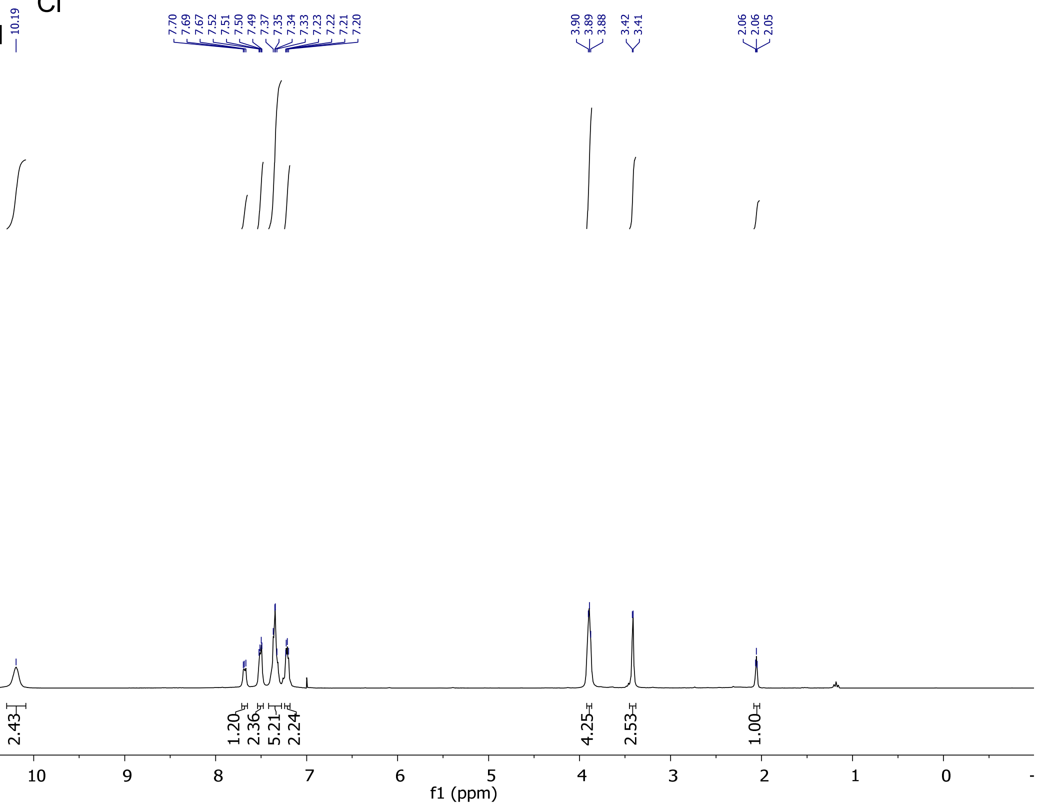

^{13}C{1H} NMR, DEPT (75 MHz, $CDCl_3$)

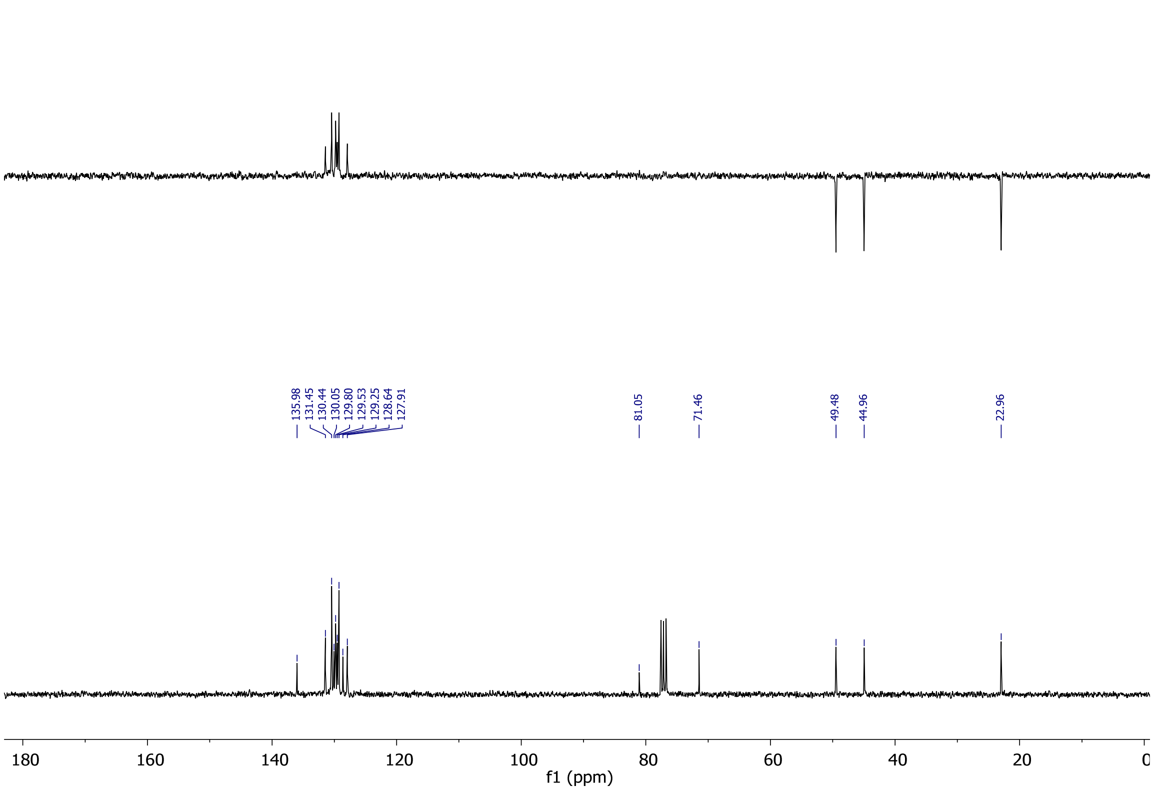

2-(But-3-yn-1-yl)aniline (**5a**)

¹H NMR (500 MHz, $CDCl_3$)

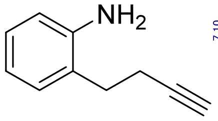

5a

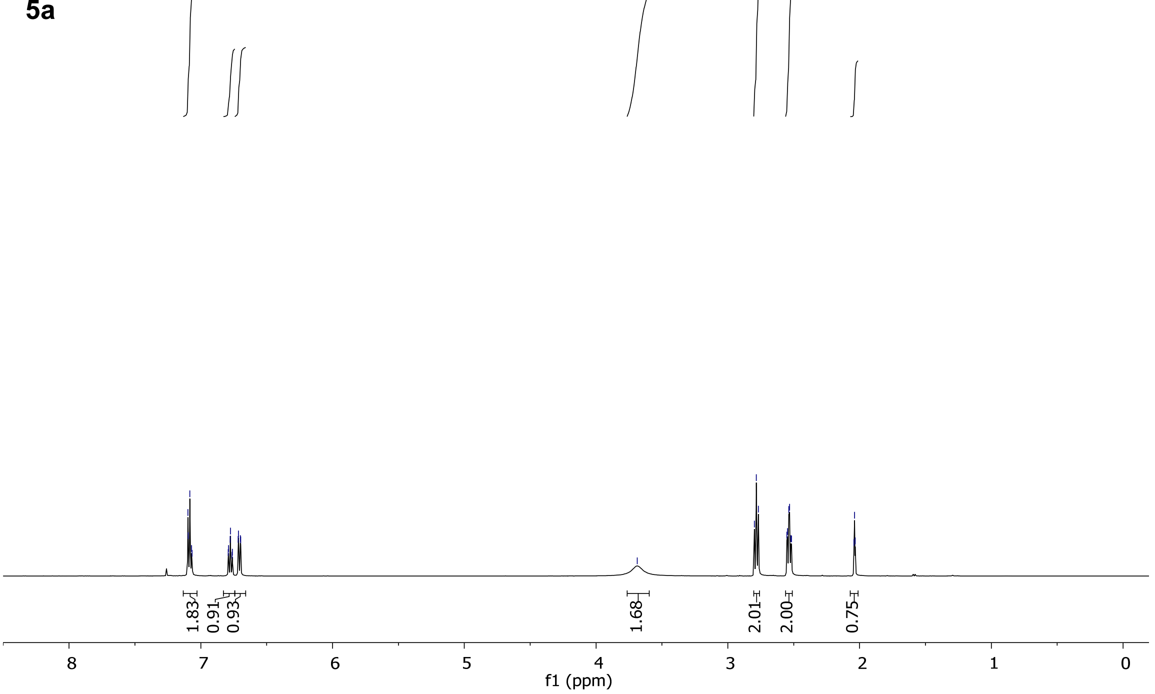

¹³C{1H} NMR, DEPT (126 MHz, $CDCl_3$)

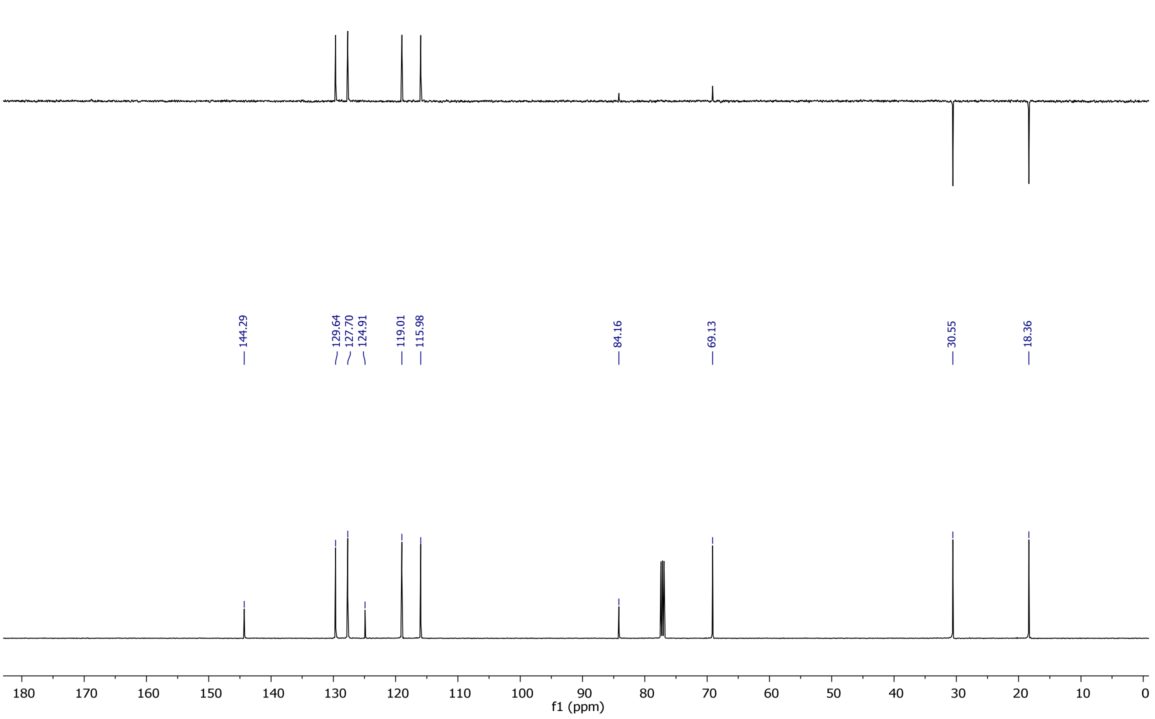

2-(But-3-yn-1-yl)-N-methylaniline (**5b**)

^{1}H NMR (400 MHz, $CDCl_3$)

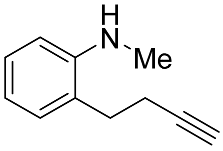

5b

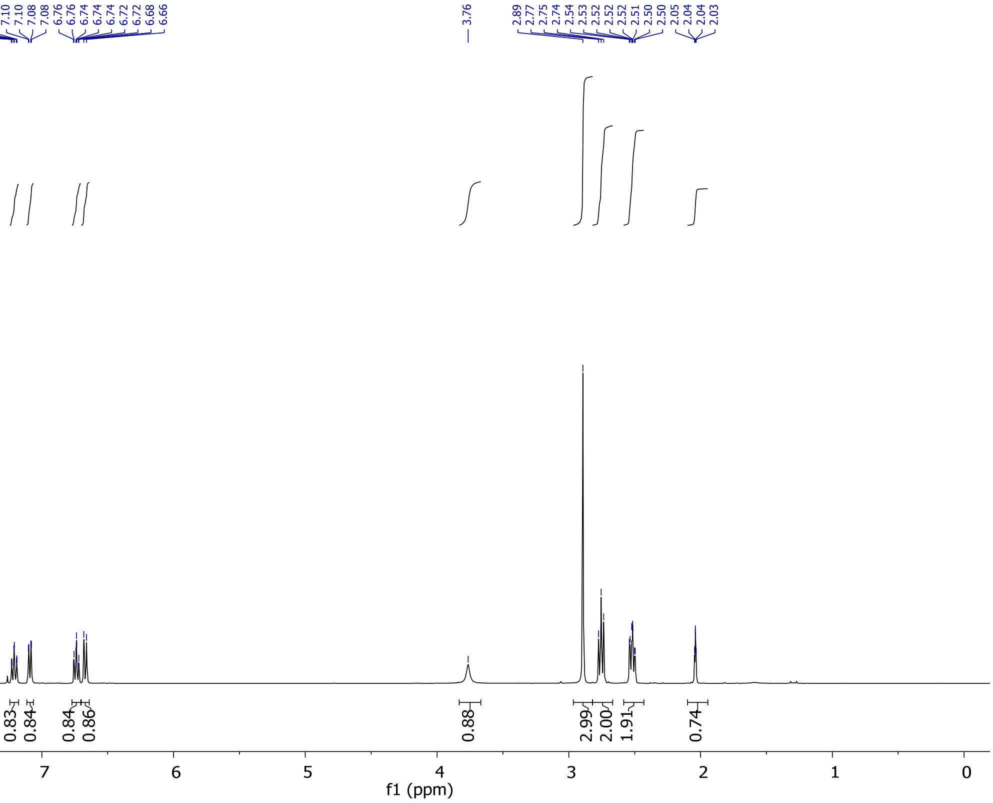

^{13}C{1H} NMR, DEPT (101 MHz, $CDCl_3$)

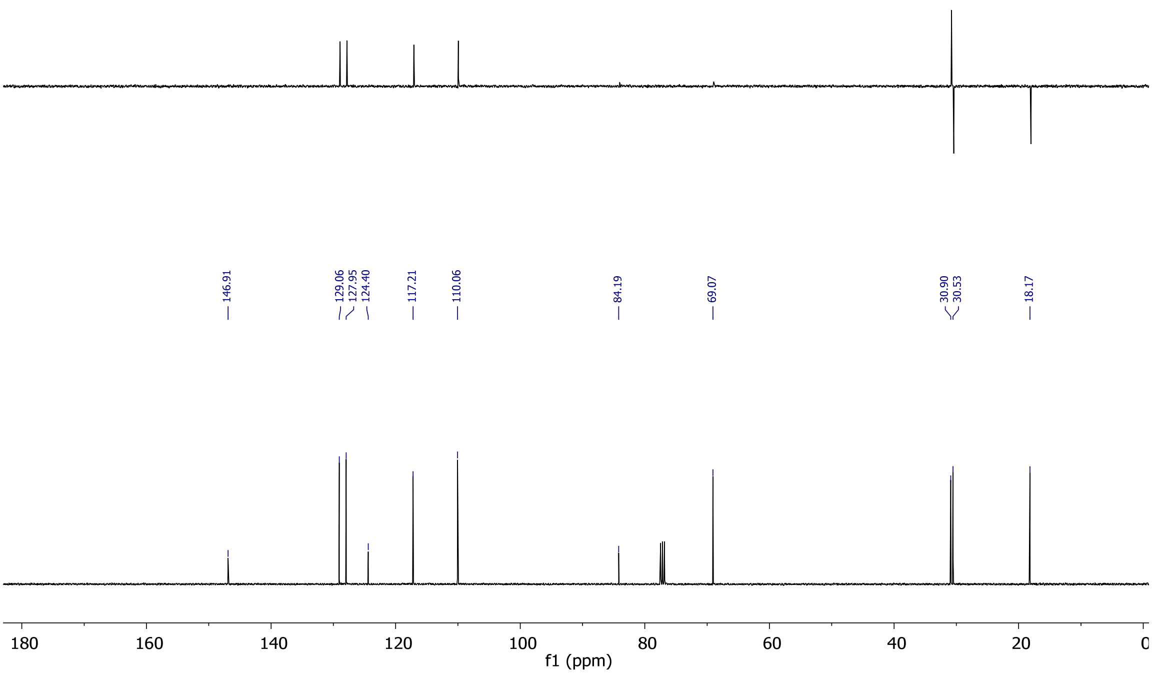

2-(Prop-2-yn-1-yloxy)aniline (**5c**)

[1]**H NMR** (500 MHz, $CDCl_3$)

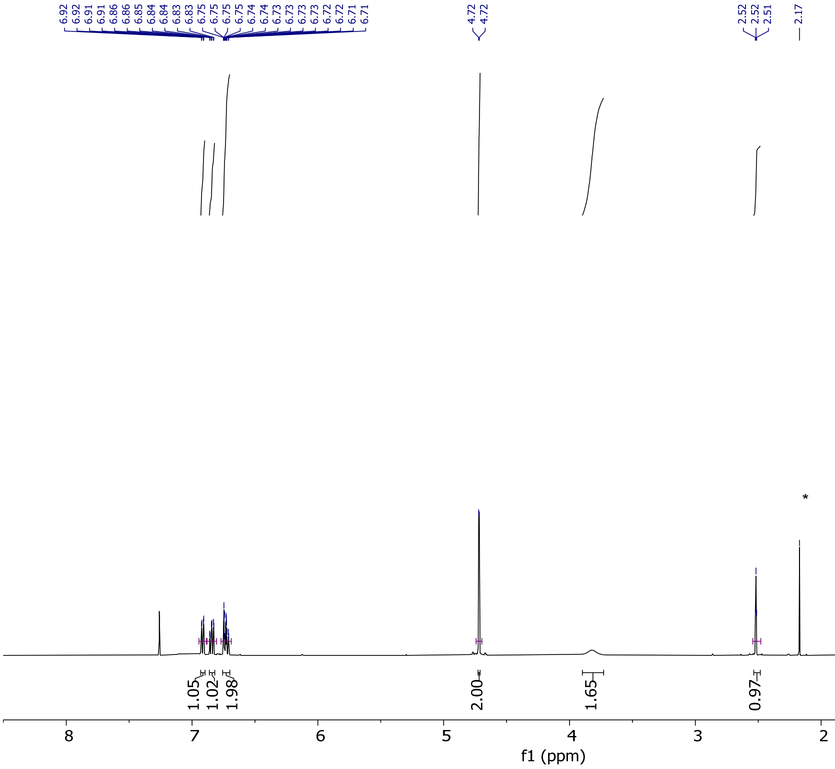

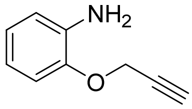

5c

[13]**C{1H} NMR, DEPT** (126 MHz, $CDCl_3$)

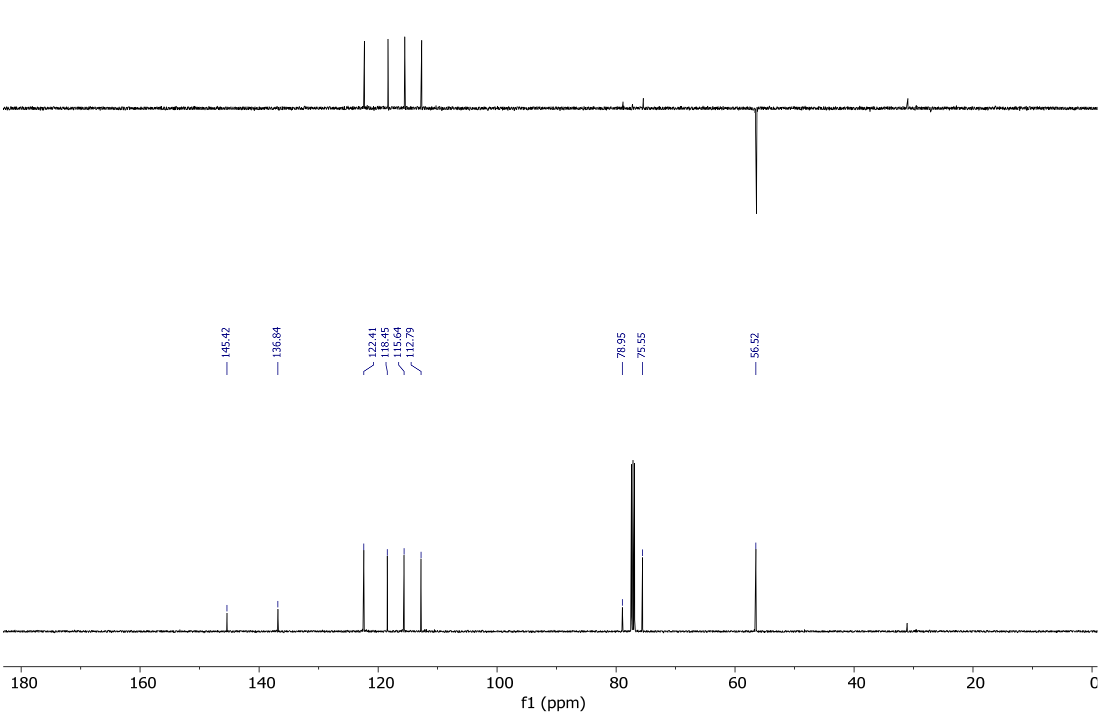

N-Methyl-2-(prop-2-yn-1-yloxy)aniline (**5d**)

[1]**H NMR** (300 MHz, $CDCl_3$)

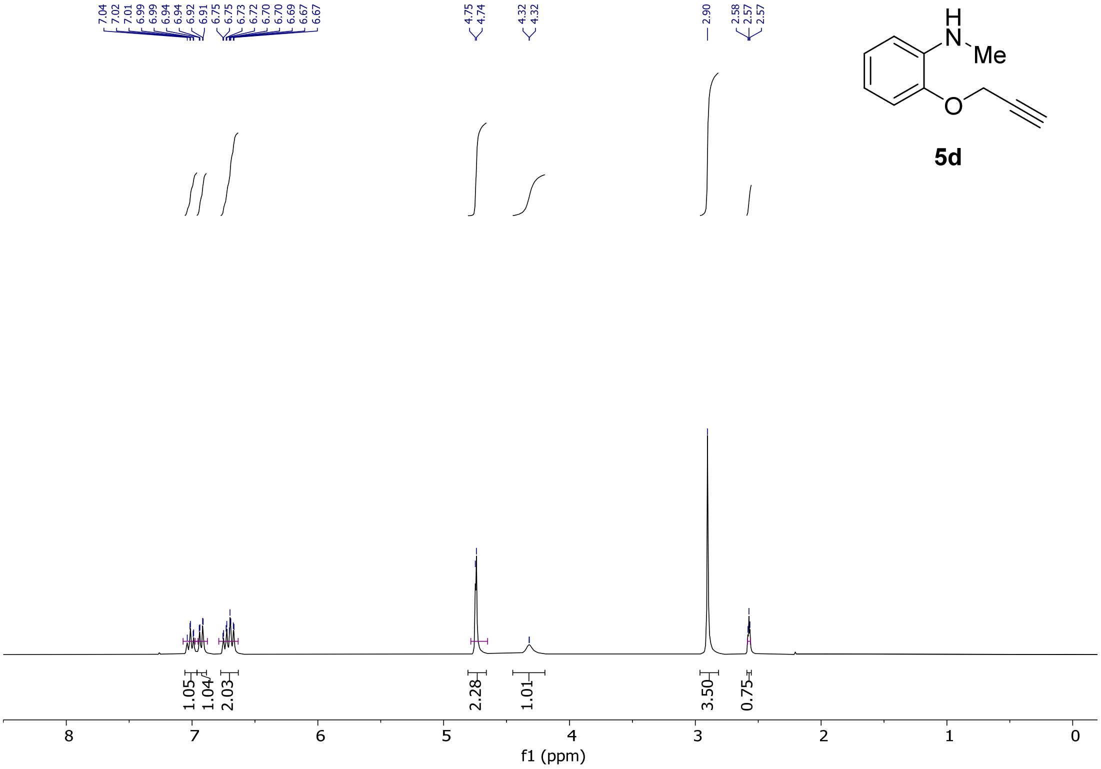

5d

[13]**C{1H} NMR** (75 MHz, $CDCl_3$)

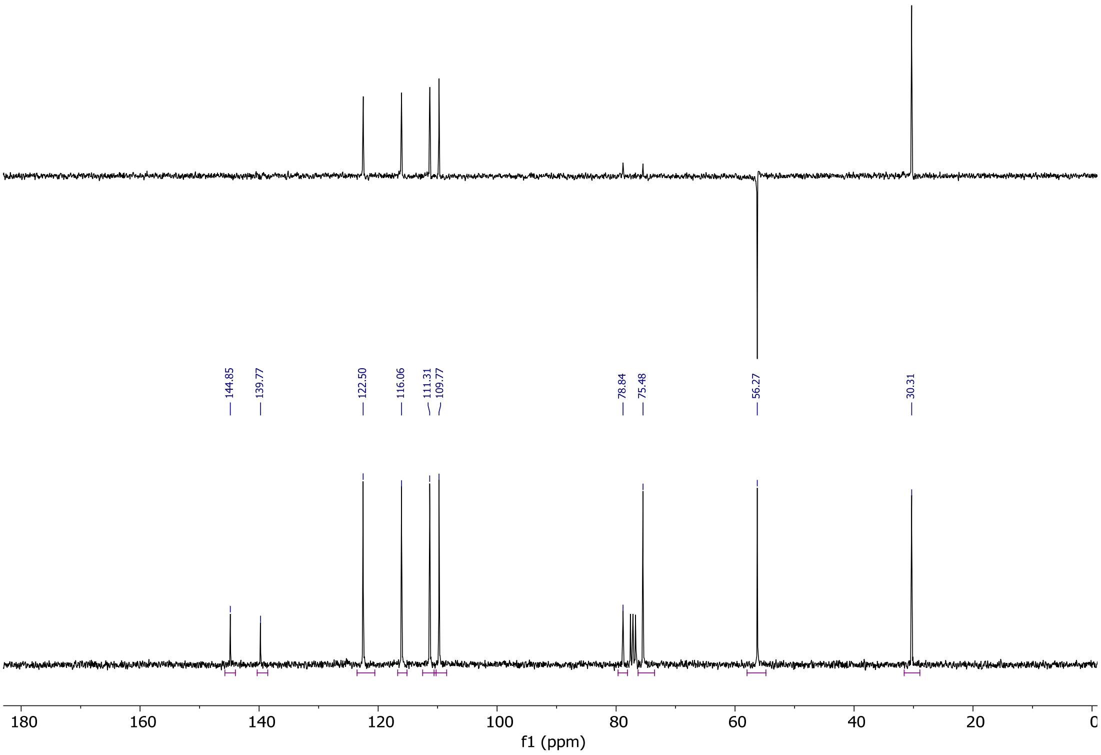

N-Benzyl-2-(prop-2-yn-1-yloxy)aniline (**5e**)

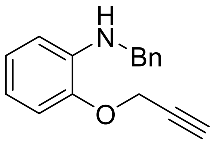

5e

^{1}H NMR (400 MHz, $CDCl_3$)

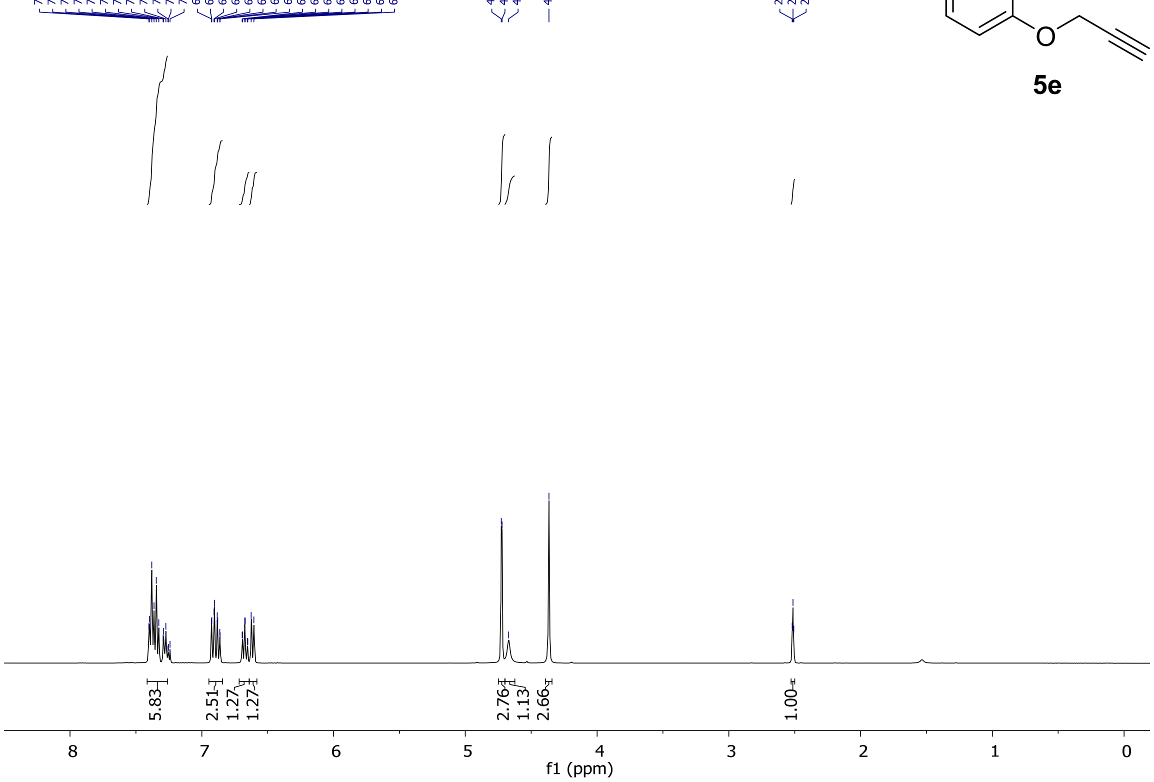

^{13}C{1H} NMR, DEPT (75 MHz, $CDCl_3$)

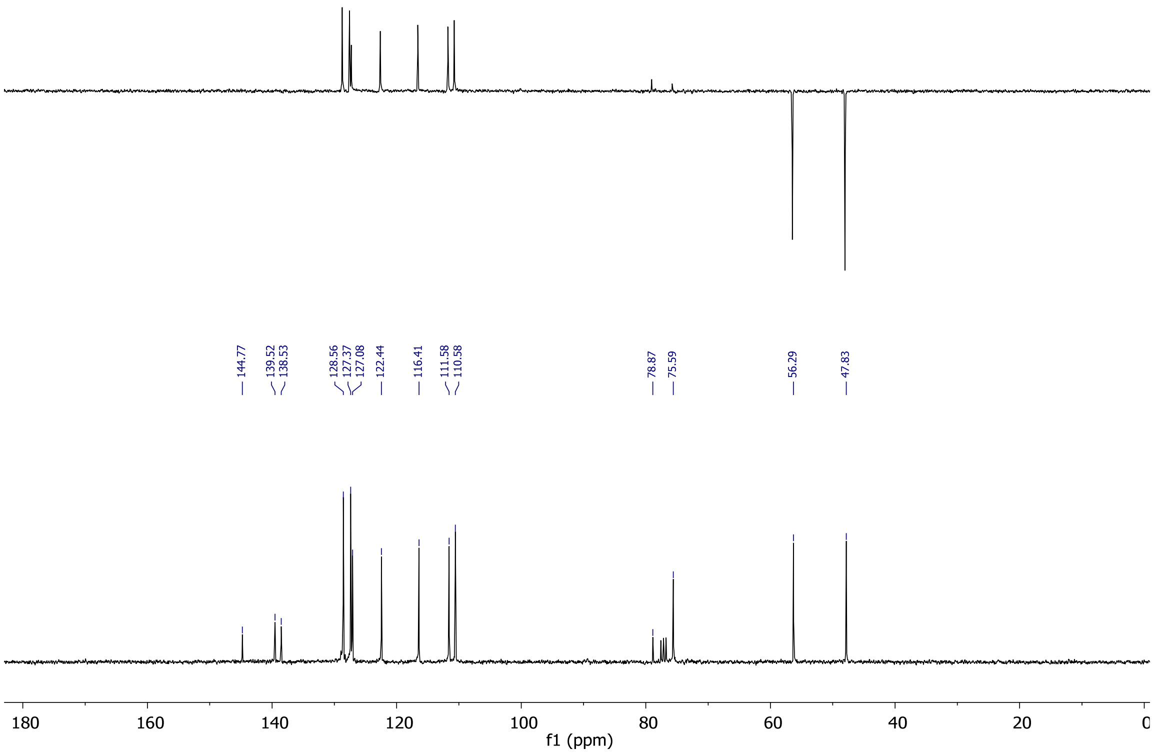

2-(Prop-2-yn-1-ylthio)aniline (**5f**)

^{1}H NMR (300 MHz, $CDCl_3$)

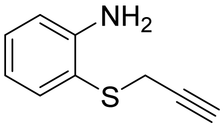

5f

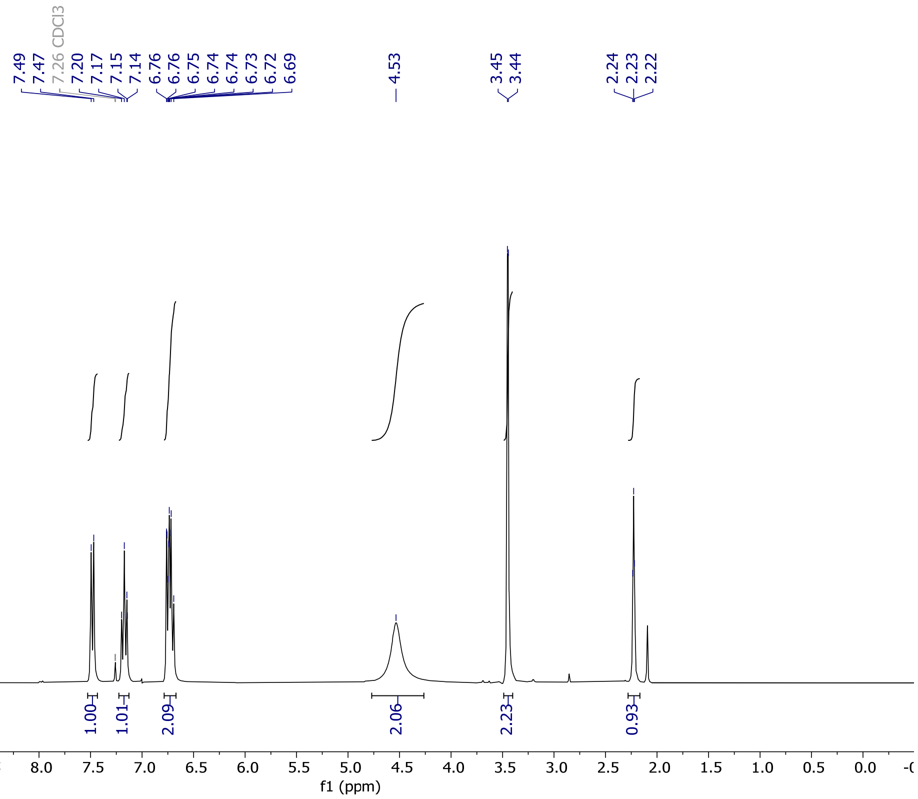

^{13}C{1H} NMR, DEPT (75 MHz, $CDCl_3$)

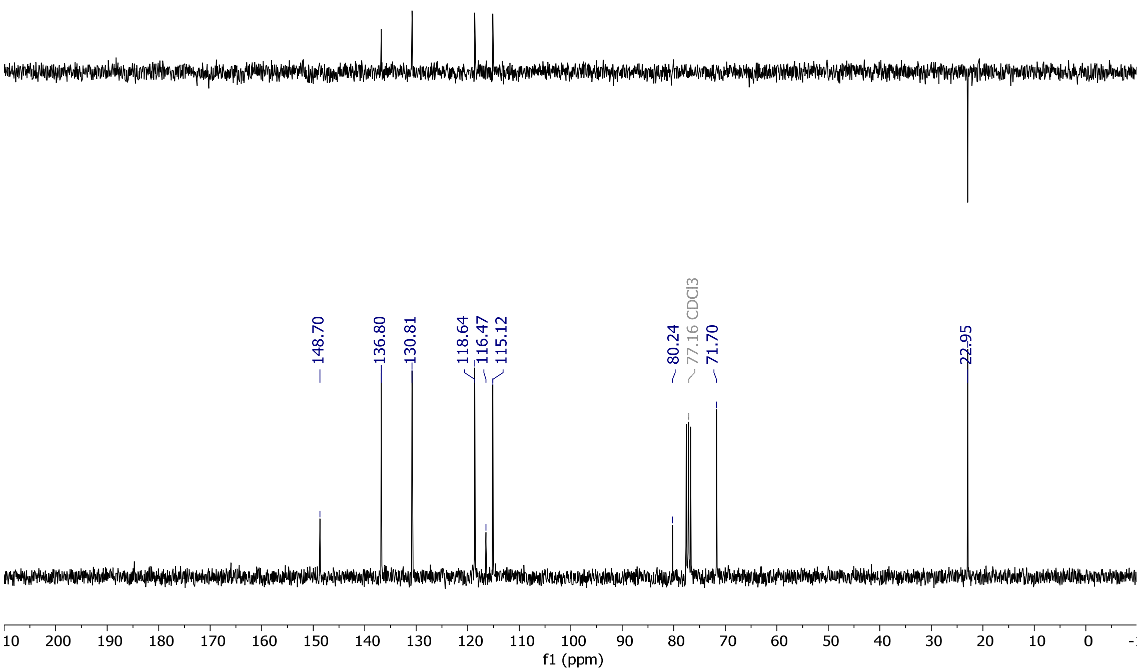

Ammonium salt of 1-(2-ethynylphenyl)-*N*-methylmethanamine (**1b·HCl**)

^{1}H NMR (500 MHz, $CDCl_3$)

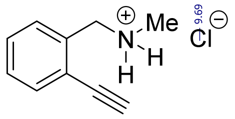

1b·HCl

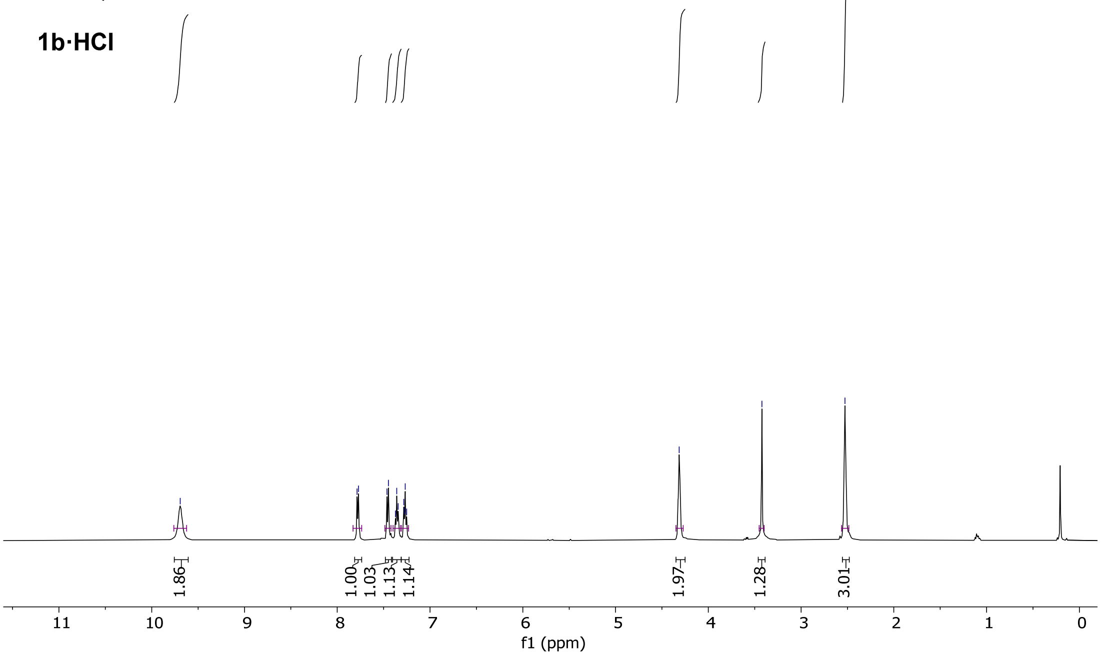

^{13}C{1H} NMR, DEPT (126 MHz, $CDCl_3$)

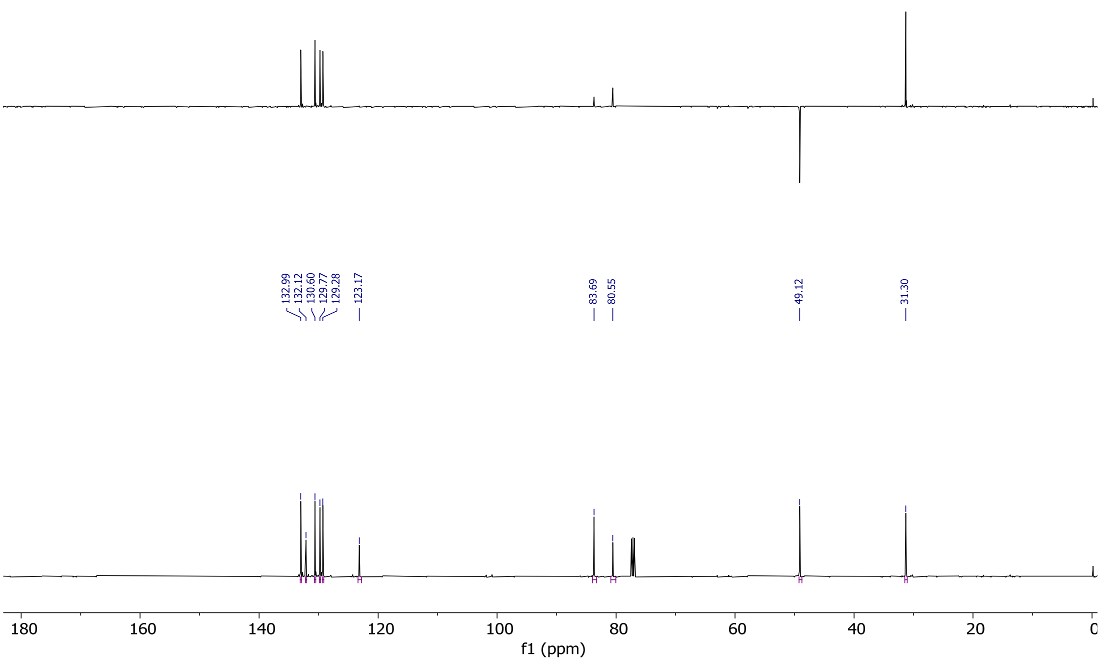

Ammonium salt 3-(2-ethynylphenyl)-N-propylpropan-1-amine (**1c·HCl**)

^{1}H NMR (400 MHz, $CDCl_3$)

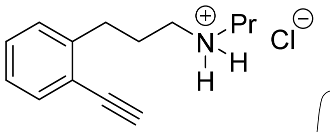

1c·HCl

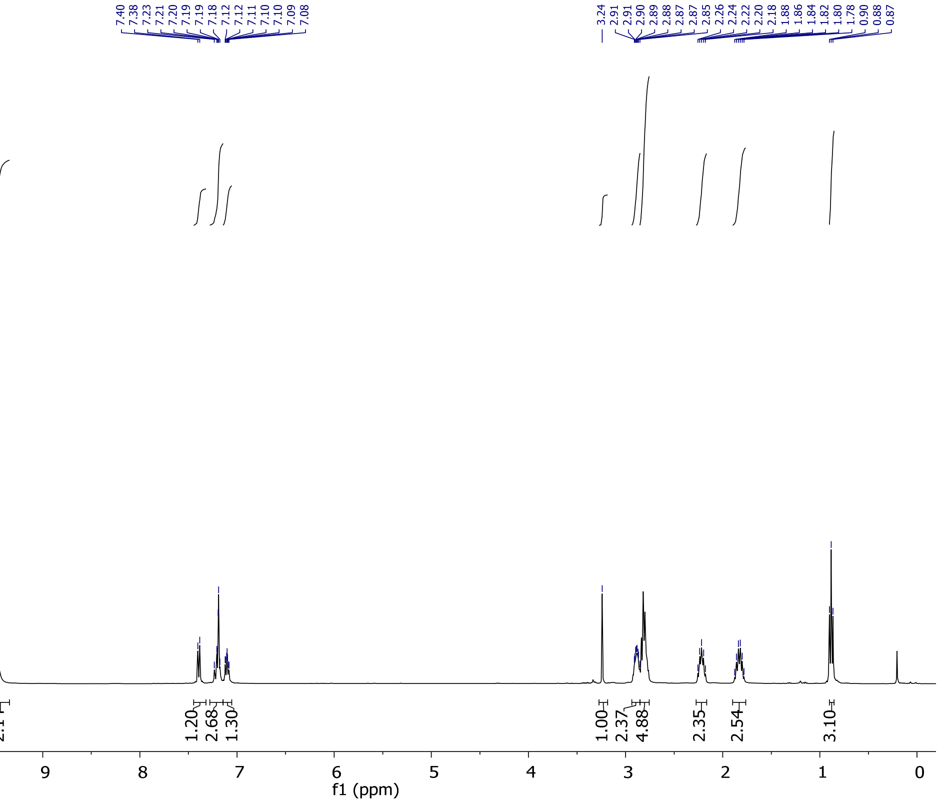

^{13}C{1H} NMR, DEPT (101 MHz, $CDCl_3$)

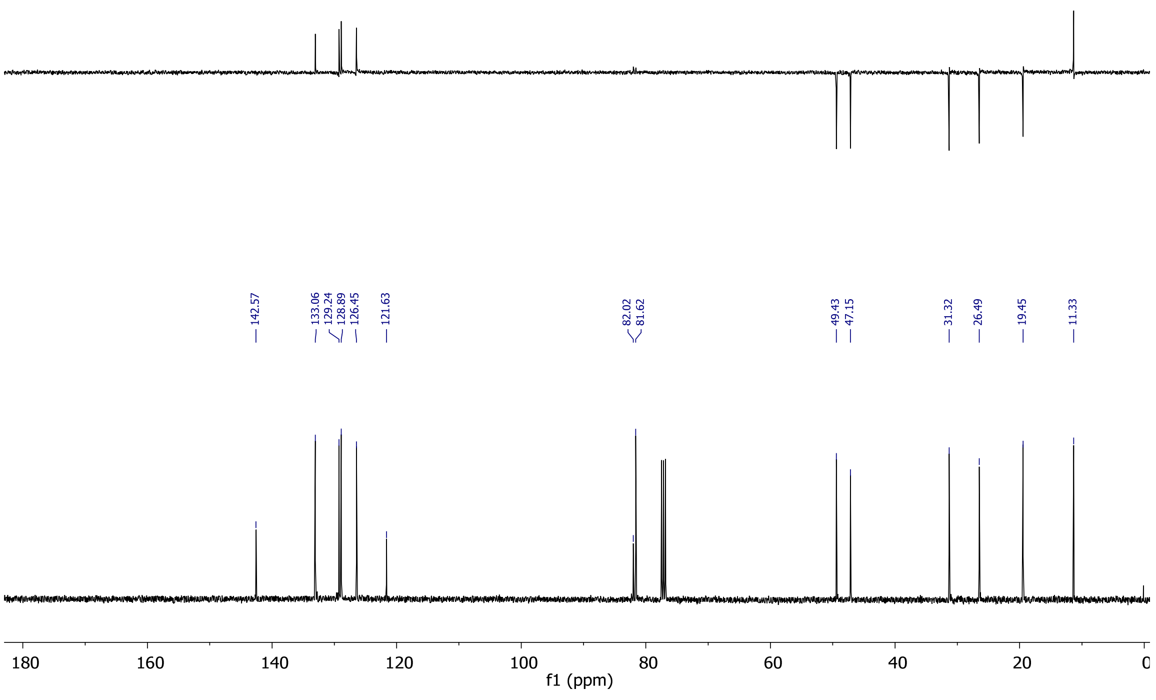

2-(2-Ethynylphenyl)-*N*-methylacetamide (**9a**)

^{1}H NMR (300 MHz, $CDCl_3$)

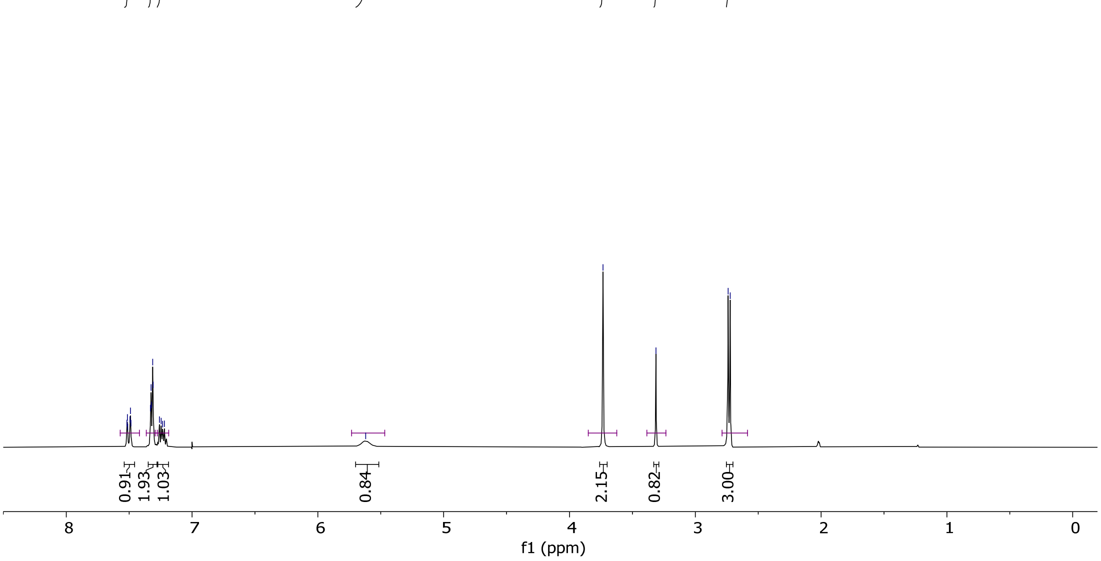

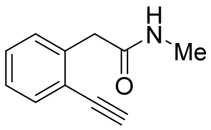

9a

^{13}C{1H} NMR, DEPT (75 MHz, $CDCl_3$)

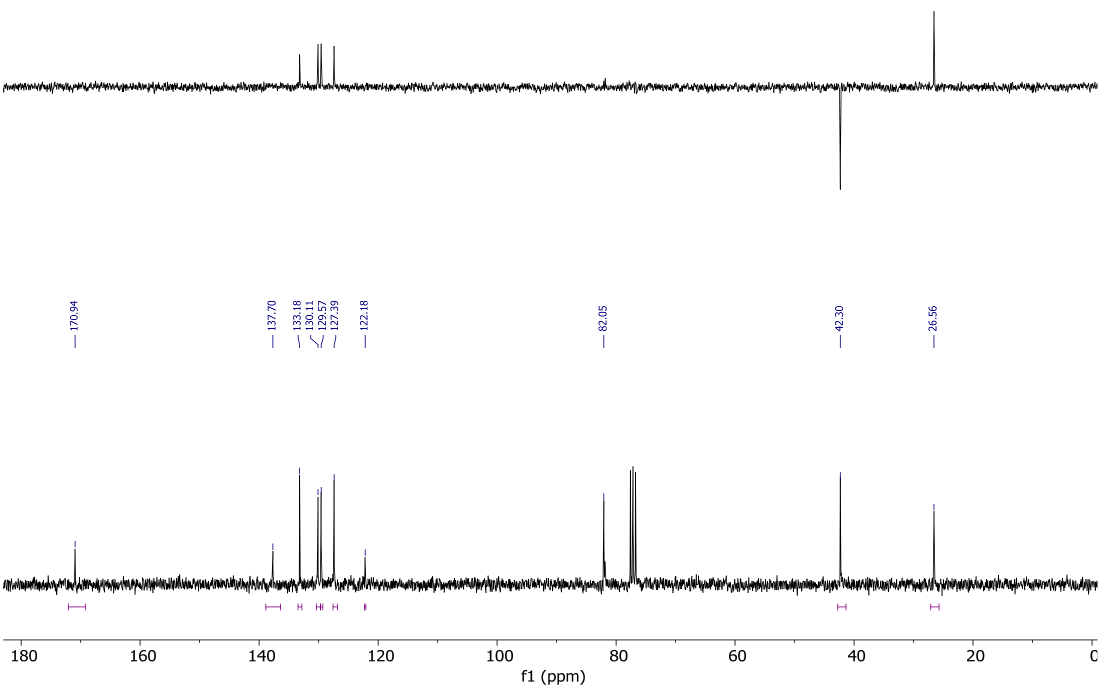

2-Ethynylbenzamide (**9b**)

^{1}H NMR (400 MHz, $CDCl_3$)

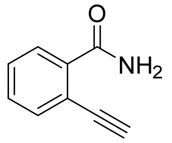

9b

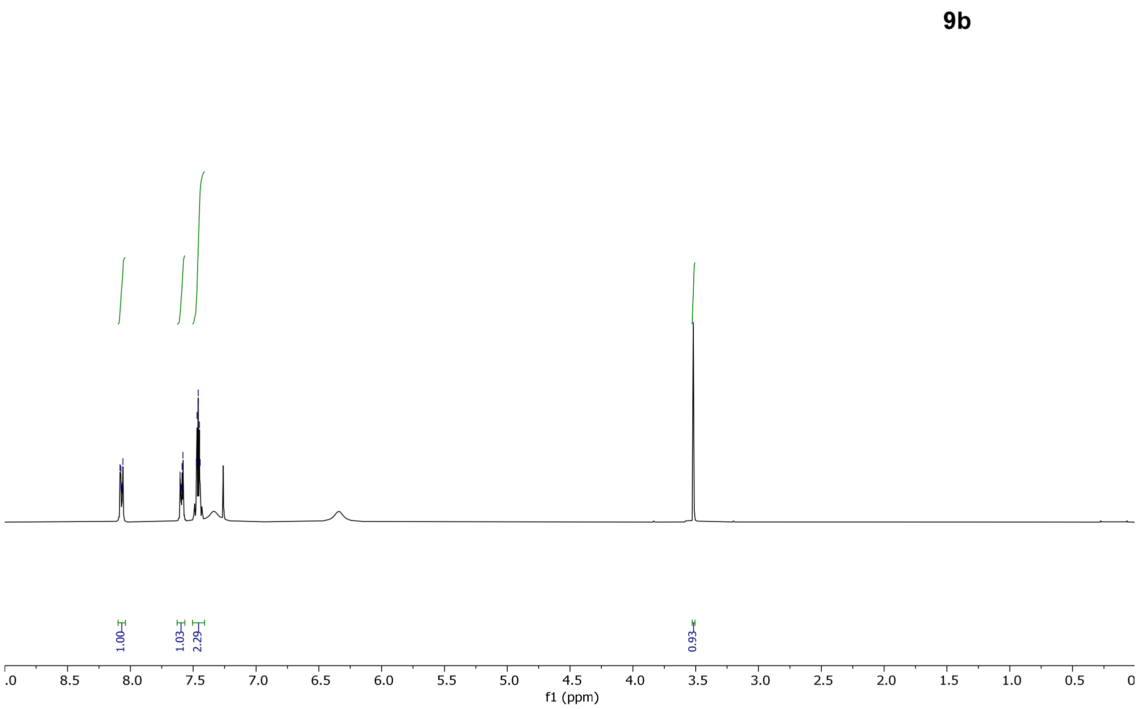

^{13}C{1H} NMR, DEPT (100 MHz, $CDCl_3$)

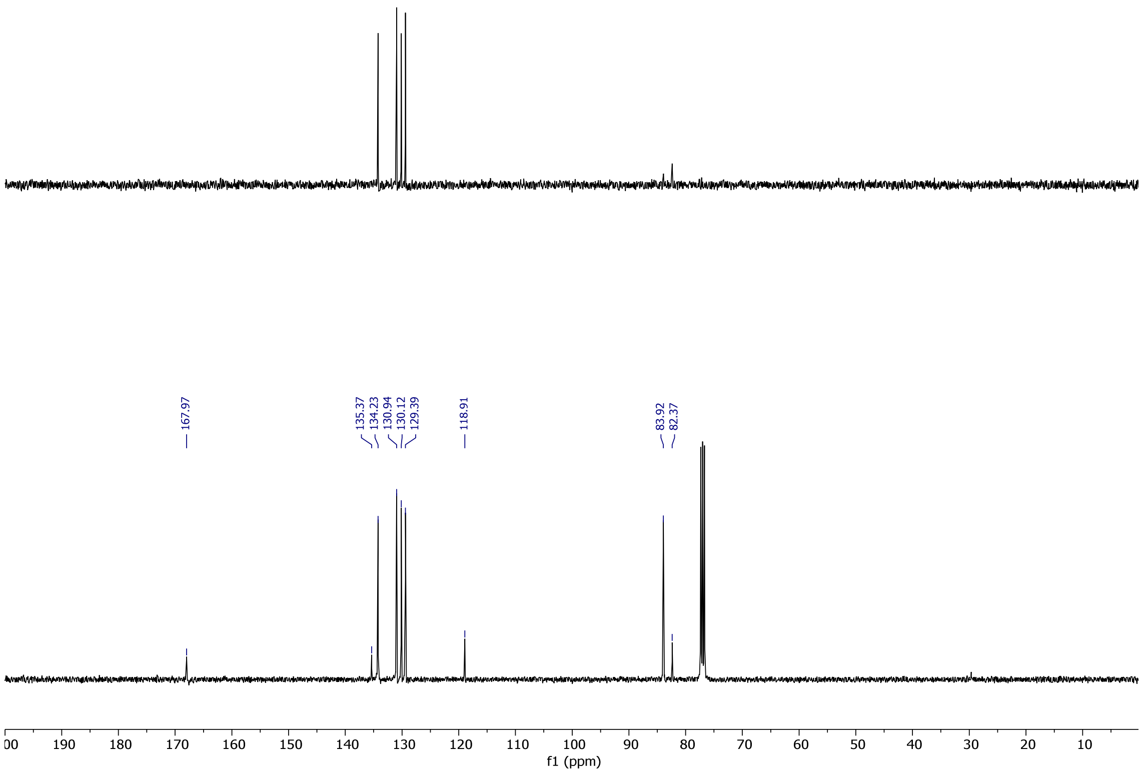

8 NMR spectra of cyclized products

3-Propyl-1,3,4,5-tetrahydro-2*H*-benzo[*d*]azepin-2-one (**2a**)

^{1}H NMR (500 MHz, $CDCl_3$)

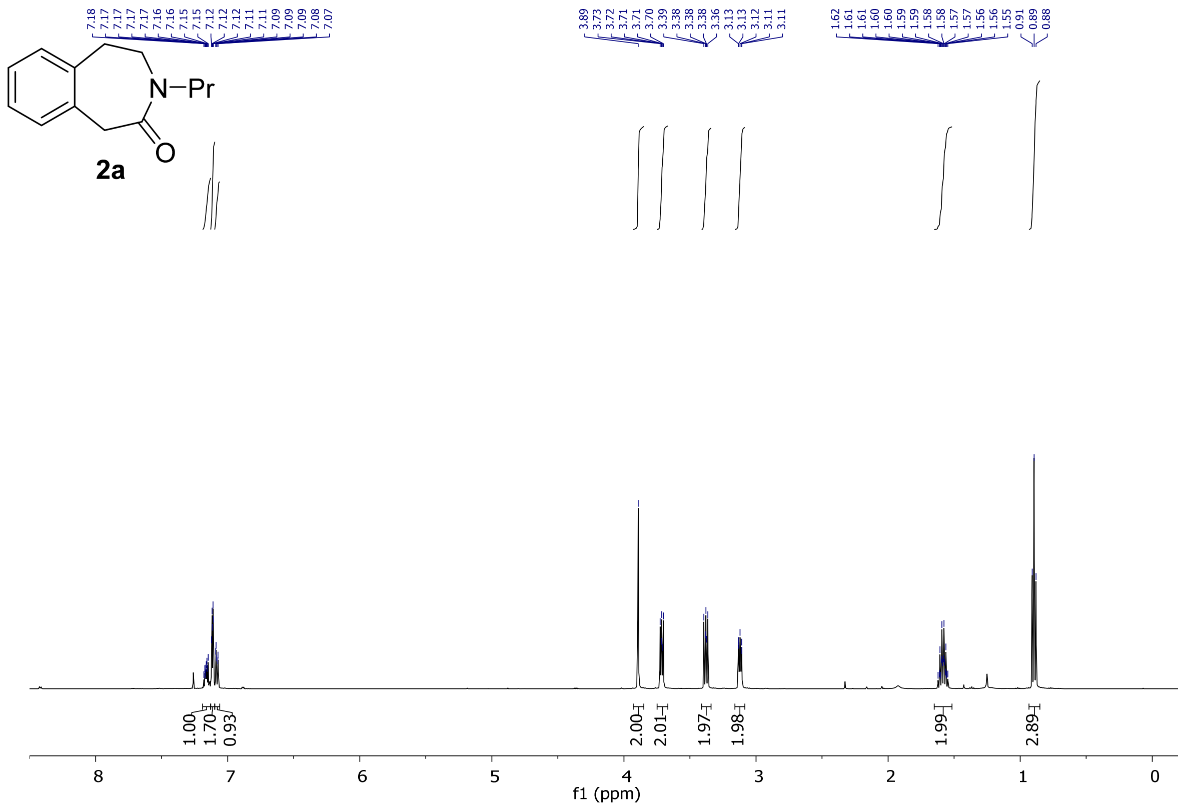

^{13}C{1H} NMR, DEPT (126 MHz, $CDCl_3$)

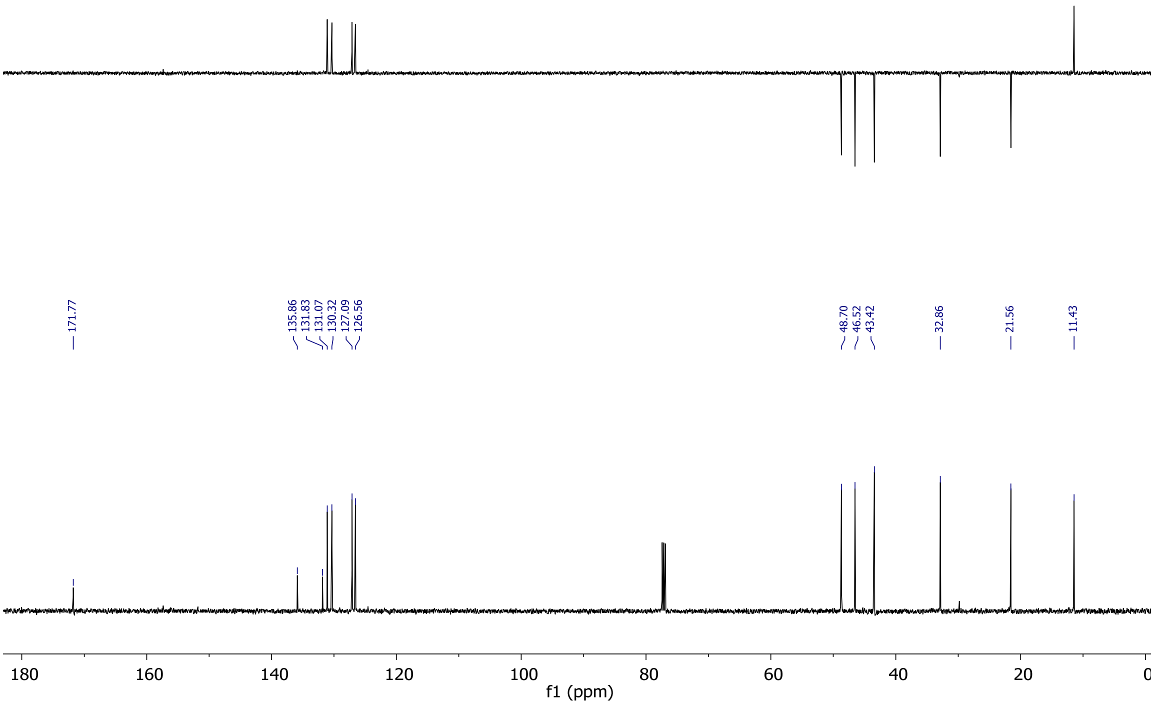

2-Methyl-1,2,4,5-tetrahydro-3*H*-benzo[*c*]azepin-3-one (**4a**)

^{1}**H NMR** (500 MHz, $CDCl_3$)

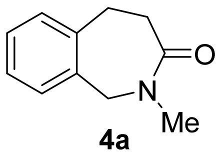

4a

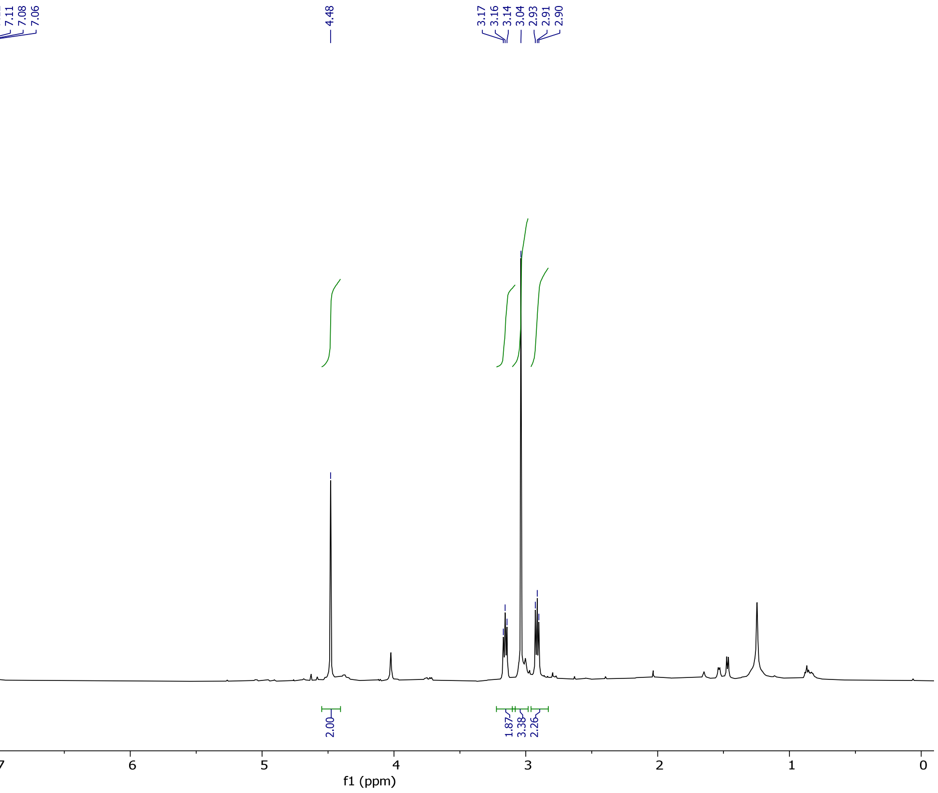

^{13}**C{1H} NMR, DEPT** (126 MHz, $CDCl_3$)

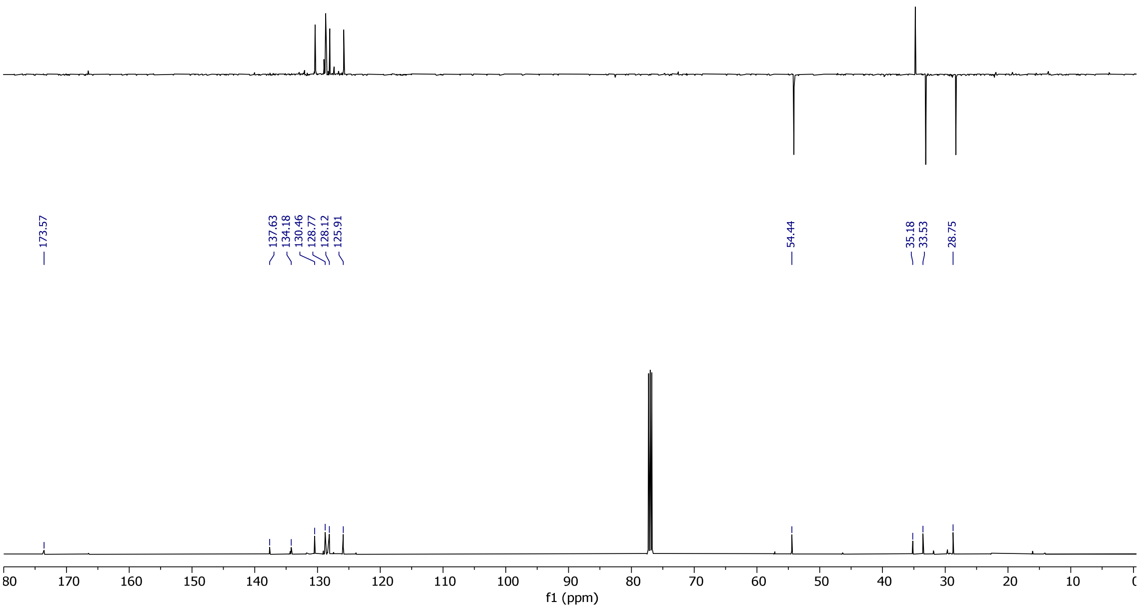

2-Benzyl-1,2,4,5-tetrahydro-3*H*-benzo[*c*]azepin-3-one (**4b**)

^{1}H NMR (300 MHz, $CDCl_3$)

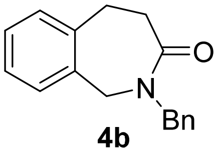

4b

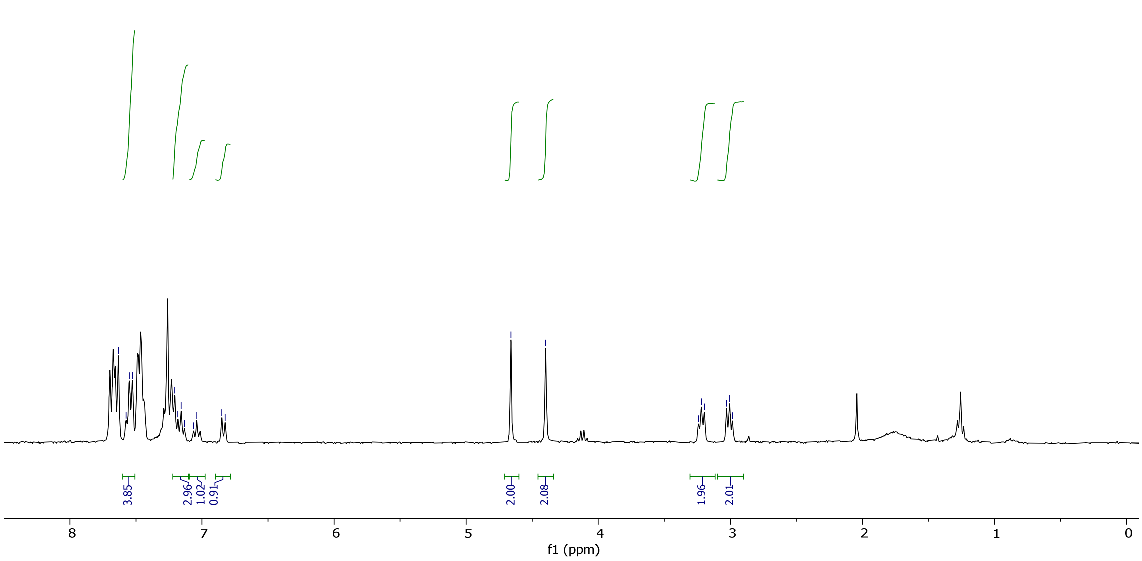

^{13}C{1H} NMR (75 MHz, $CDCl_3$)

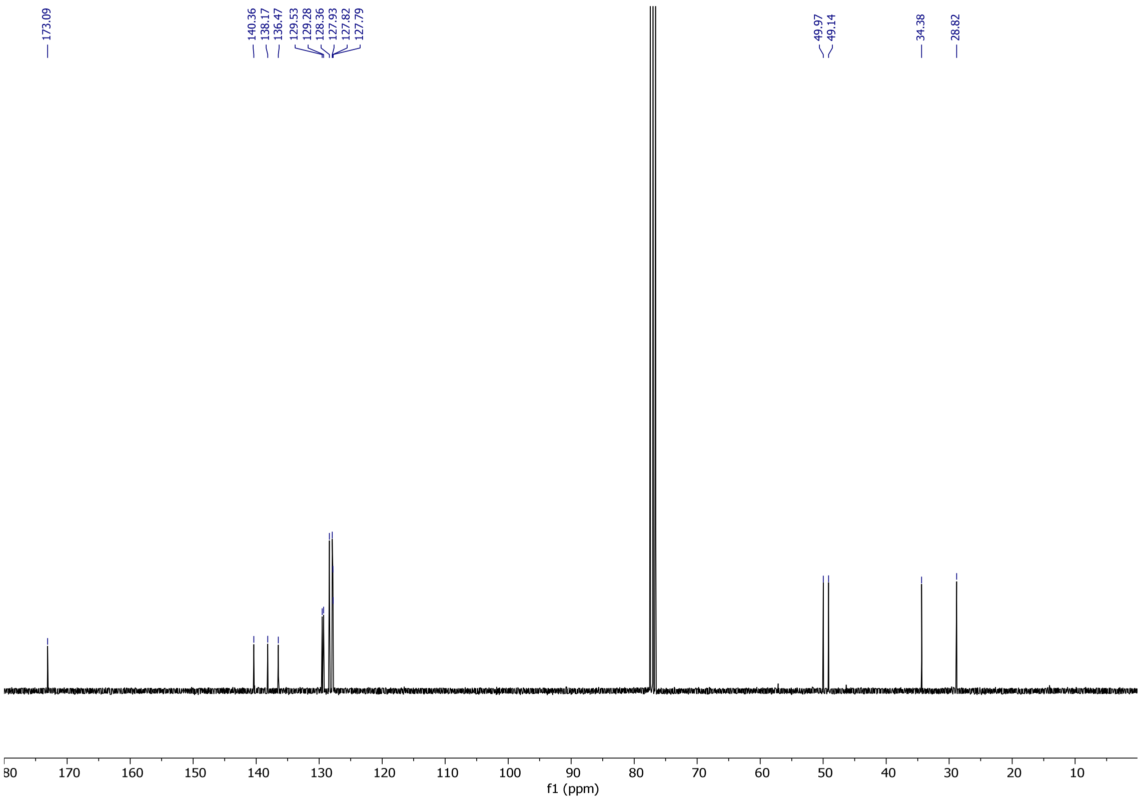

1,3,4,5-Tetrahydro-2*H*-benzo[*b*]azepin-2-one (**6a**)

^{1}H NMR (400 MHz, $CDCl_3$)

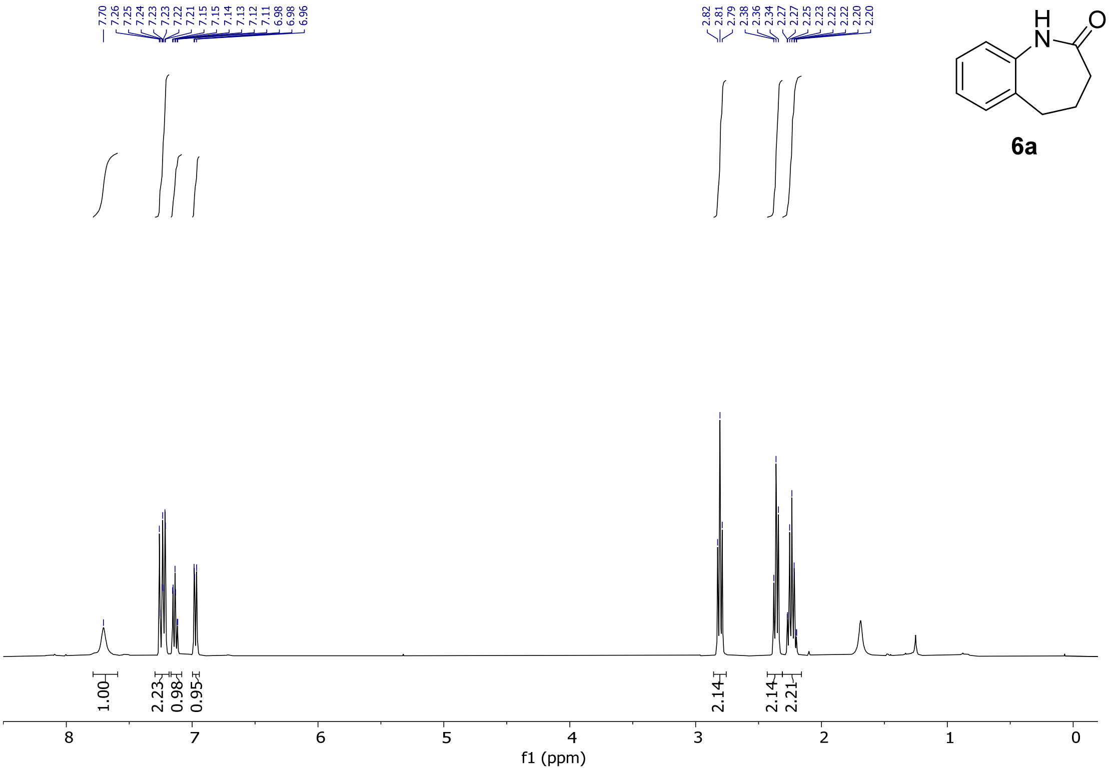

6a

^{13}C{1H} NMR, DEPT (101 MHz, $CDCl_3$)

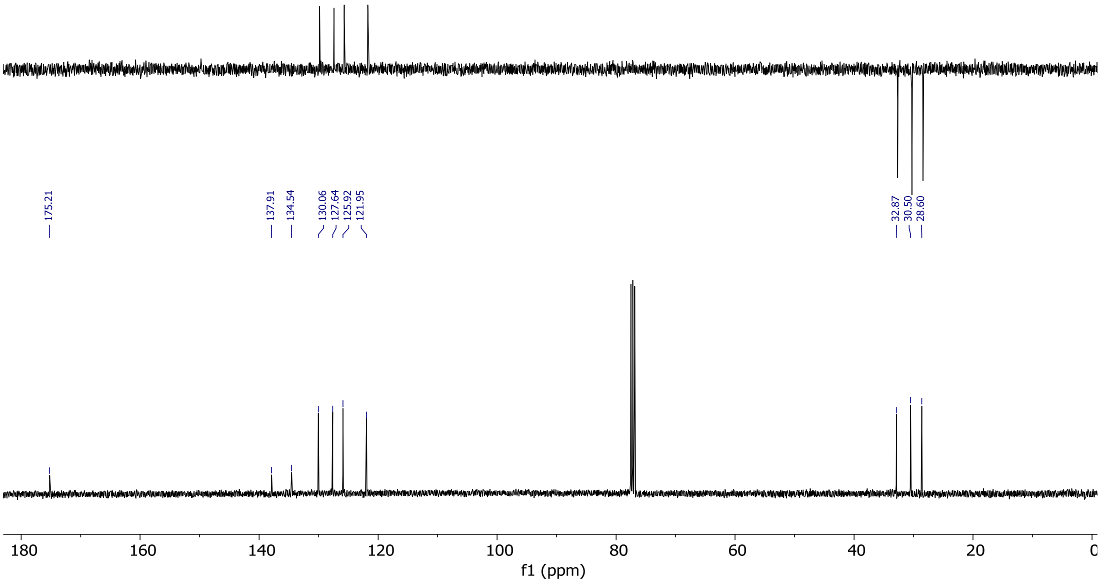

1-Methyl-1,3,4,5-tetrahydro-2*H*-benzo[*b*]azepin-2-one (**6b**)

^{1}H NMR (500 MHz, CDCl$_3$)

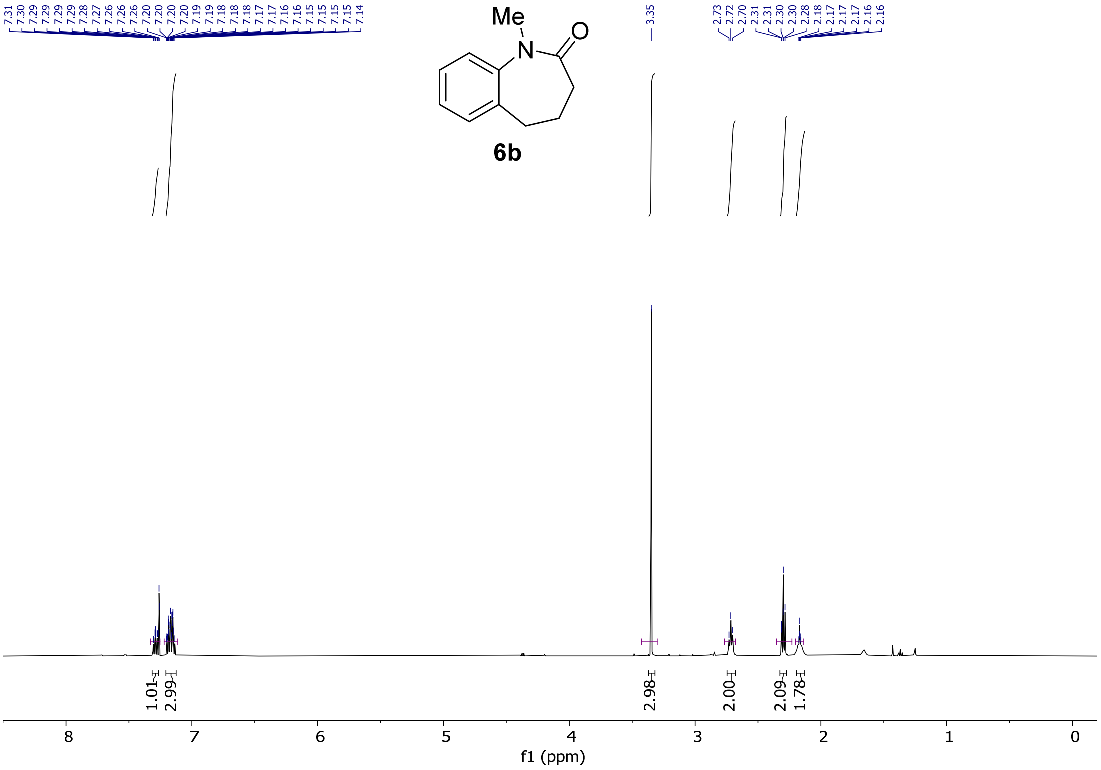

^{13}C{1H} NMR, DEPT (126 MHz, CDCl$_3$)

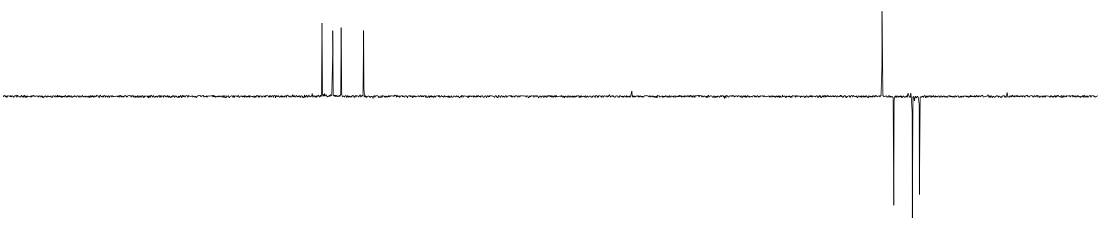

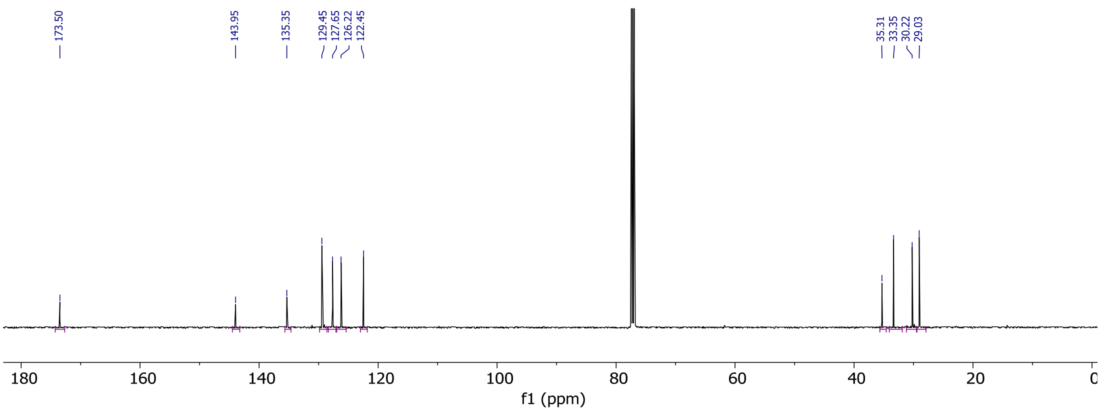

2,3-Dihydrobenzo[*b*][1,4]oxazepin-4(5*H*)-one (**6c**)

^{1}H NMR (400 MHz, $CDCl_3$)

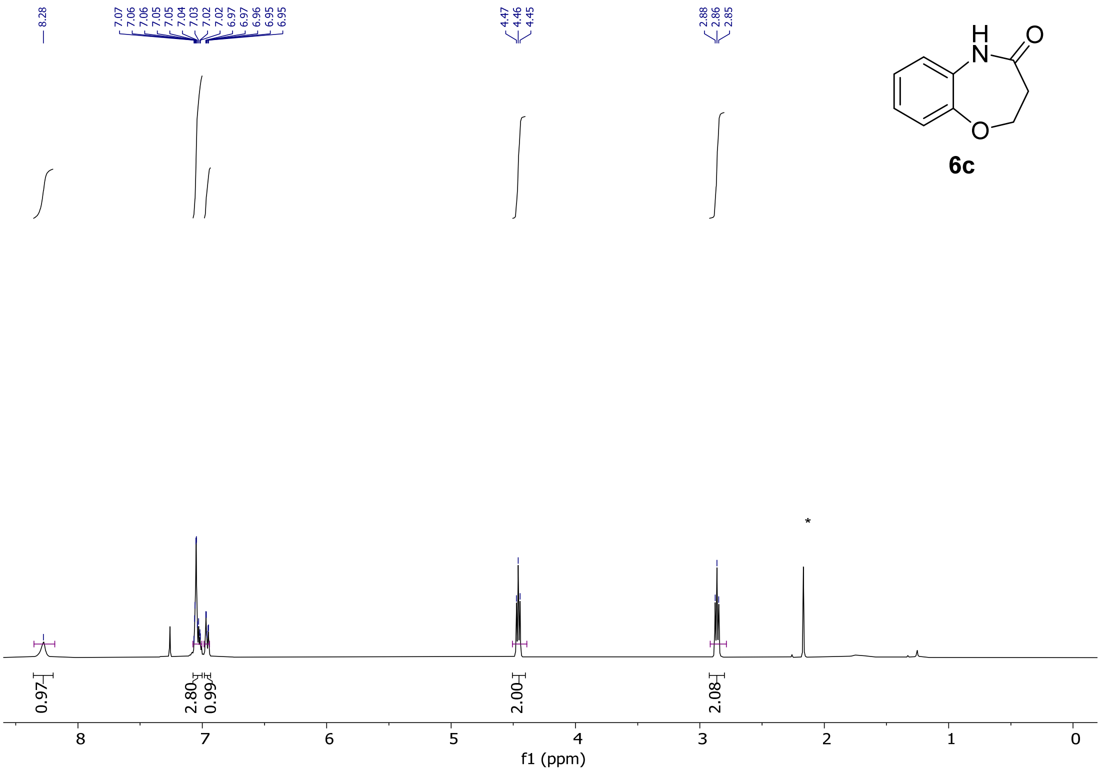

6c

^{13}C{1H} NMR, DEPT (101 MHz, $CDCl_3$)

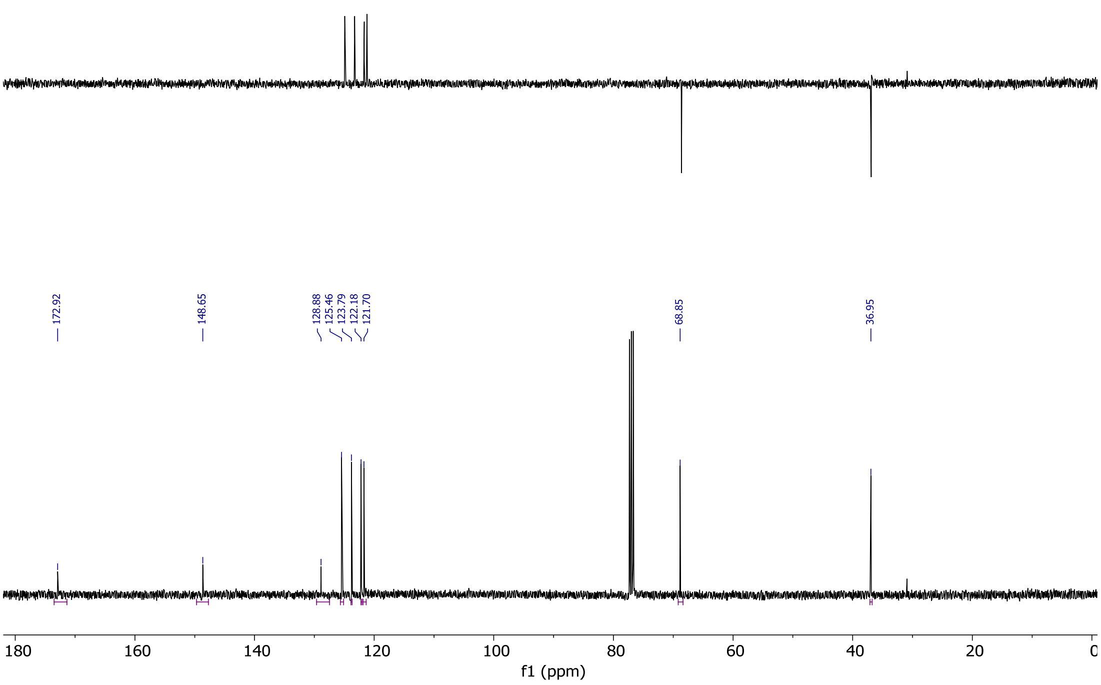

5-Methyl-2,3-dihydrobenzo[*b*][1,4]oxazepin-4(5*H*)-one (**6d**)

^{1}H NMR (400 MHz, $CDCl_3$)

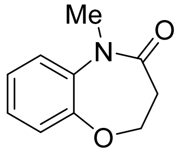

6d

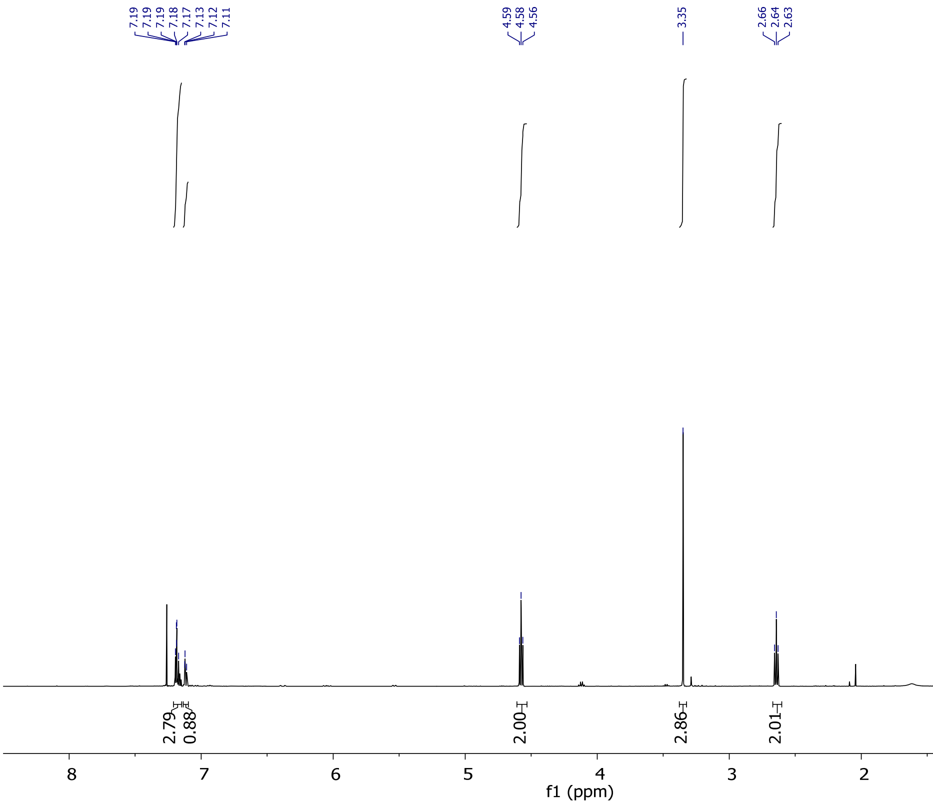

^{13}C{1H} NMR, DEPT (101 MHz, $CDCl_3$)

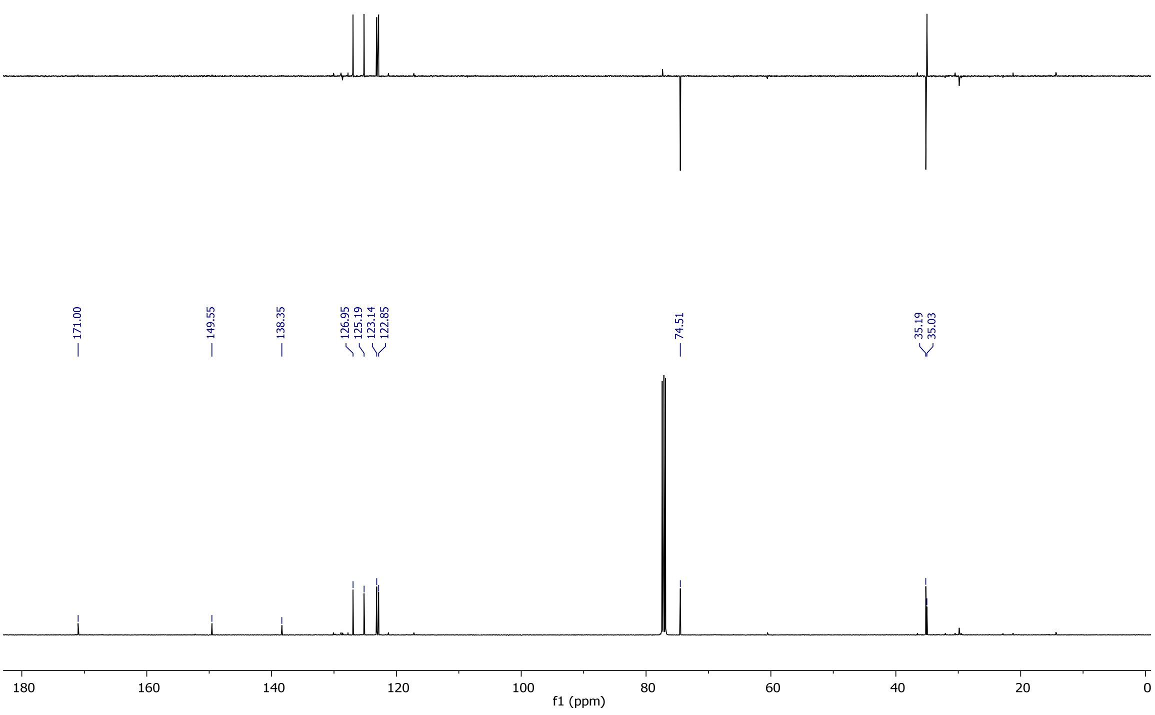

5-Benzyl-2,3-dihydrobenzo[*b*][1,4]oxazepin-4(5*H*)-one (**6e**)

^{1}H NMR (500 MHz, CDCl$_3$)

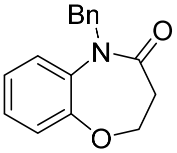

6e

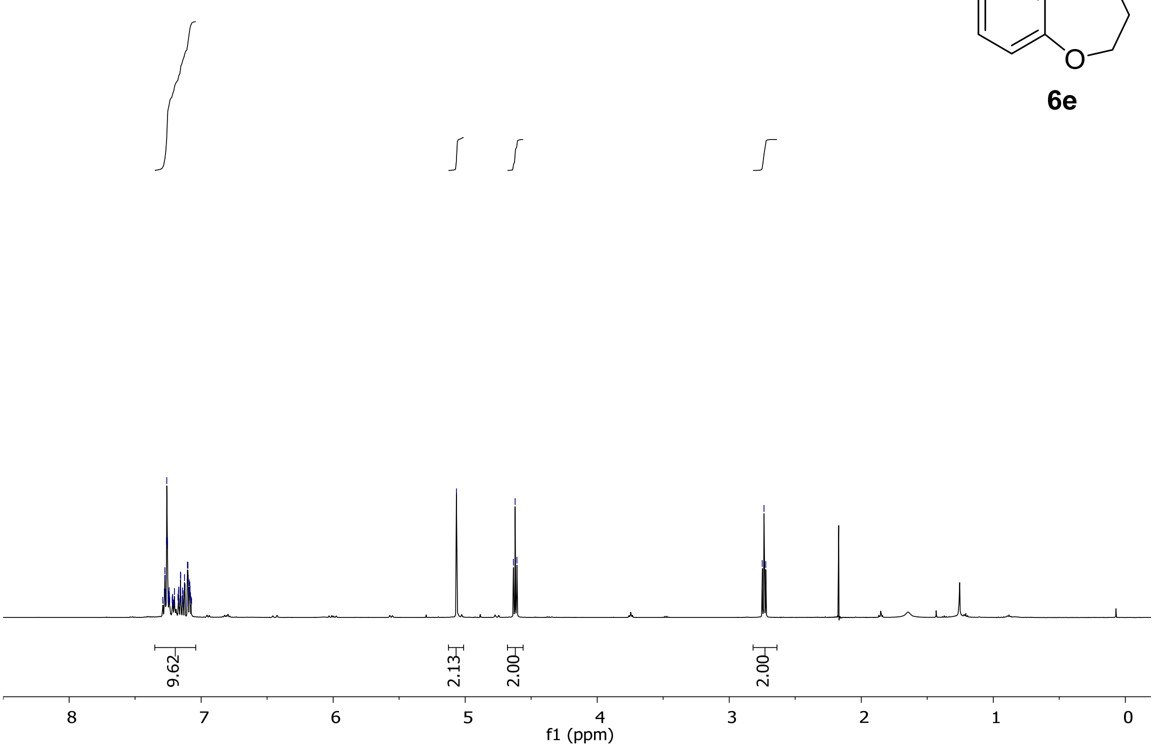

^{13}C{1H} NMR (126 MHz, CDCl$_3$)

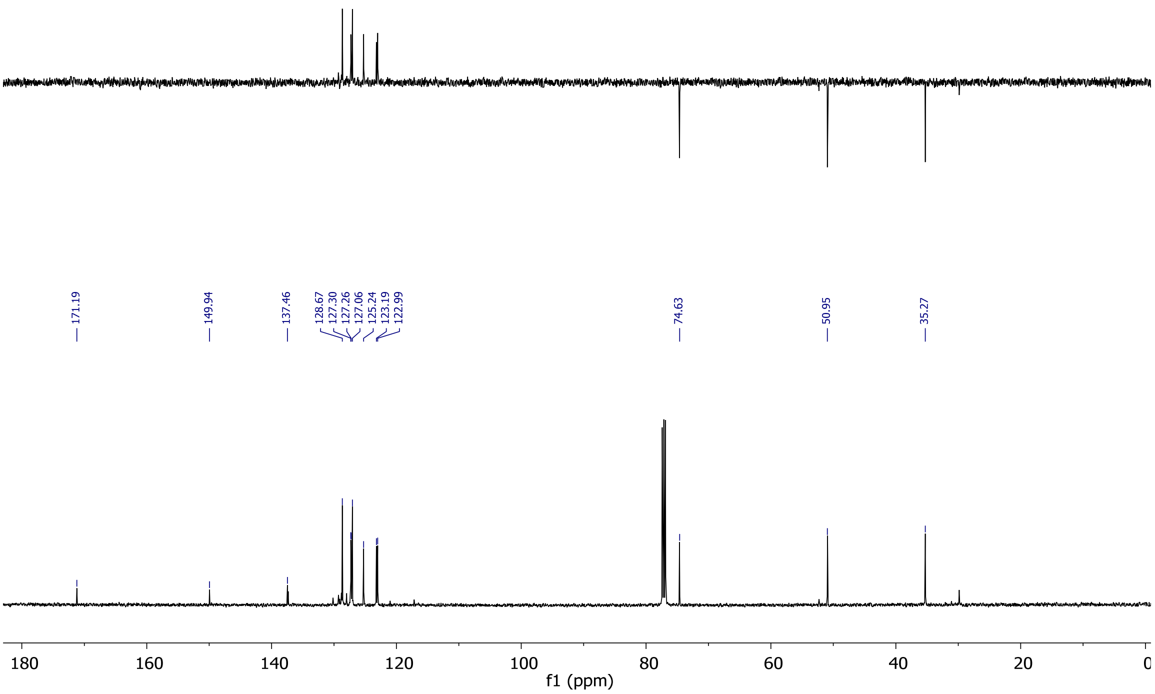

2,3-Dihydrobenzo[*b*][1,4]thiazepin-4(5*H*)-one (**6f**)

^{1}H NMR (500 MHz, CDCl$_3$)

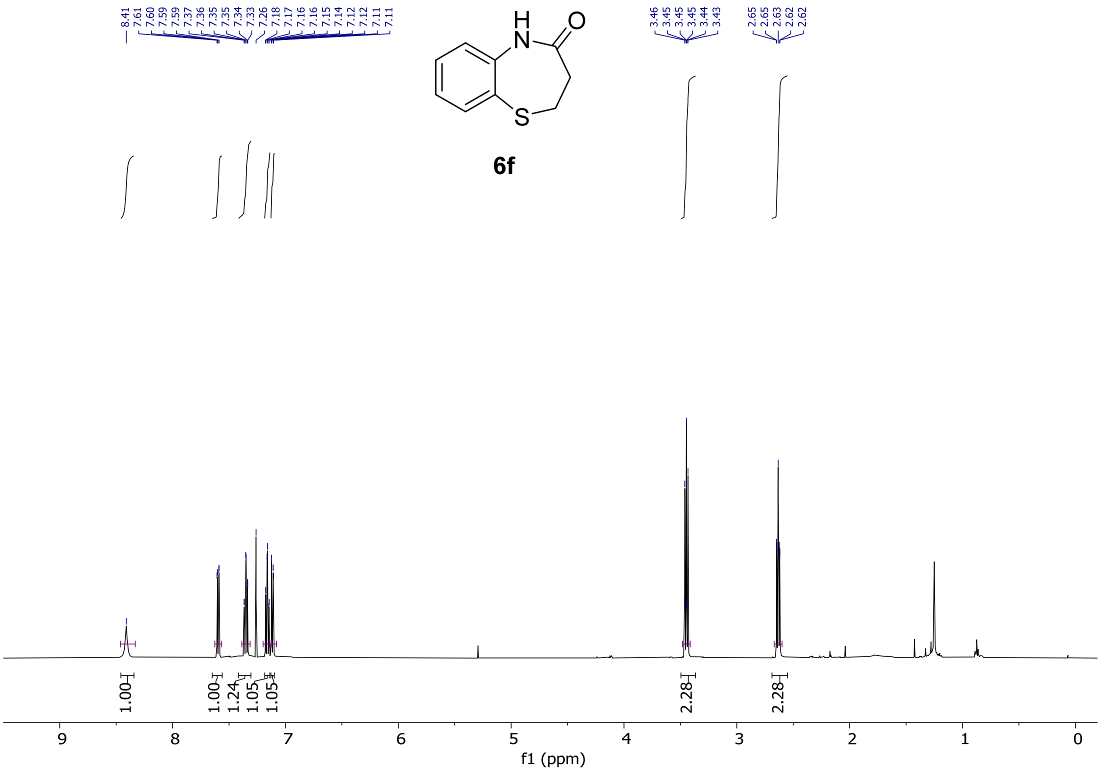

^{13}C{1H} NMR, DEPT (126 MHz, CDCl$_3$)

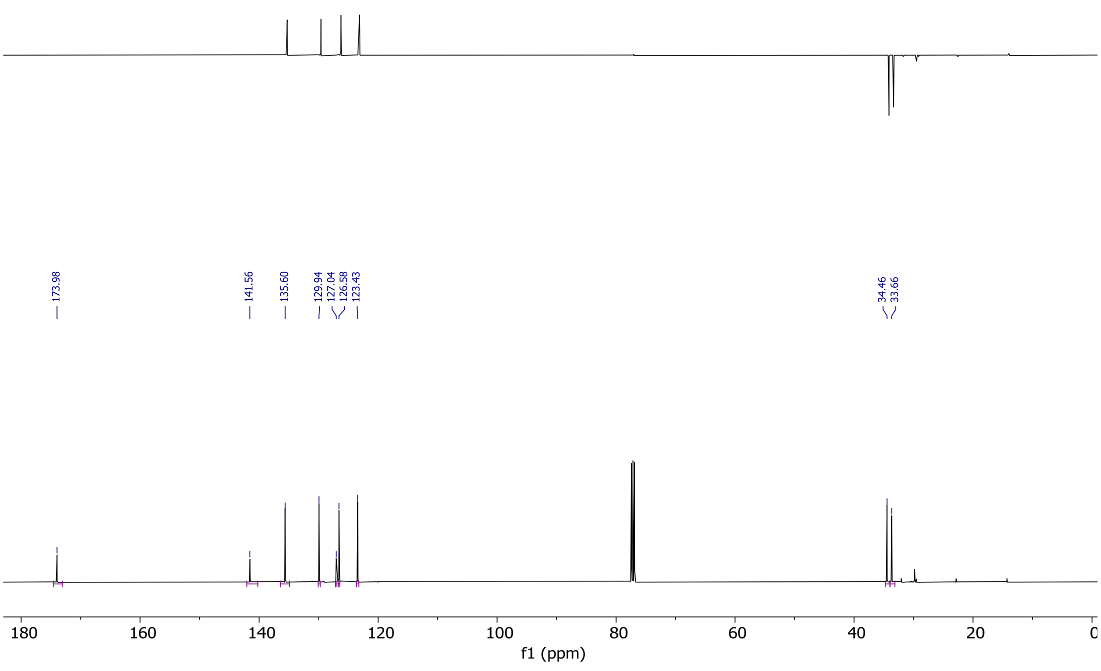

3-Propyl-3,4,5,6-tetrahydrobenzo[*d*]azocin-2(1*H*)-one (**7**)

^{1}H NMR (500 MHz, $CDCl_3$)

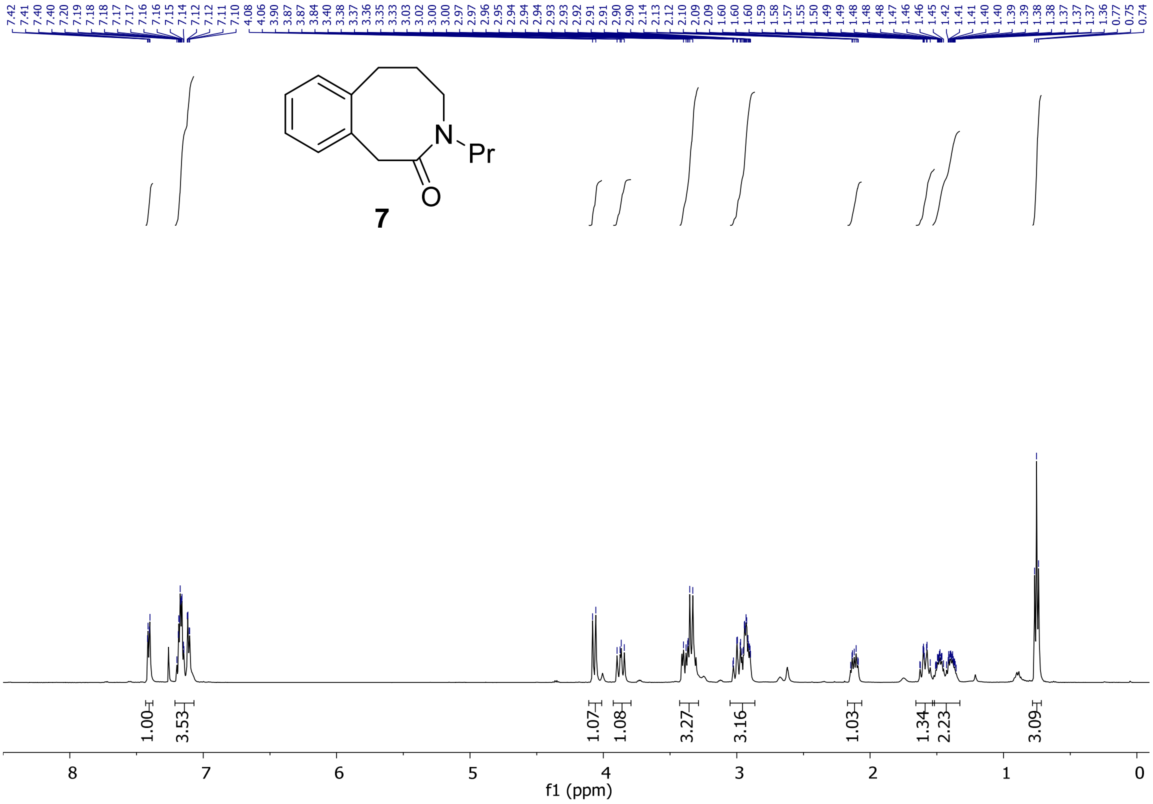

^{13}C{1H} NMR (126 MHz, $CDCl_3$)

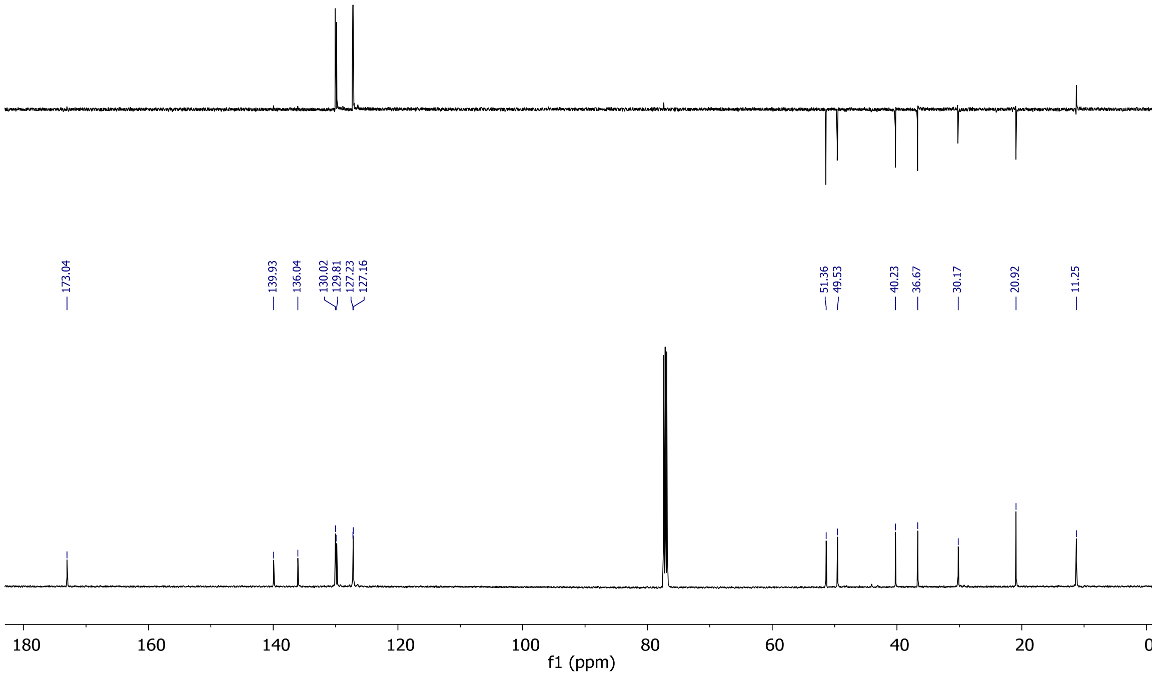

3-Propyl-2,3-dihydro-1*H*-benzo[*d*]azepine (**8a**)

^{1}H NMR (300 MHz, $CDCl_3$)

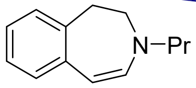

8a

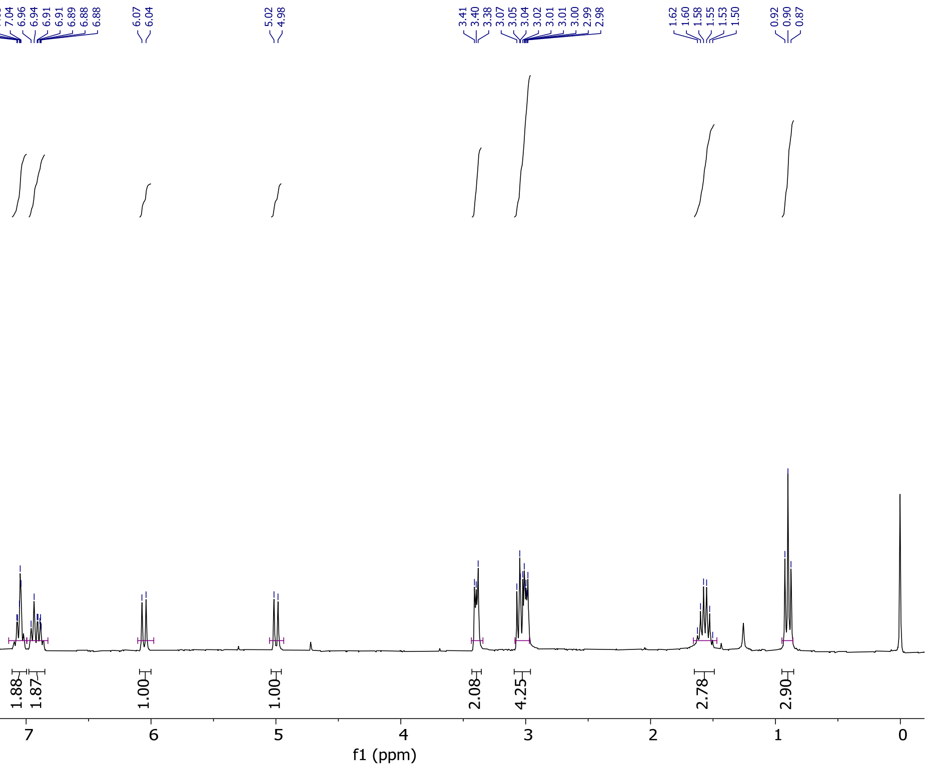

^{13}C{1H} NMR, DEPT (101 MHz, $CDCl_3$)

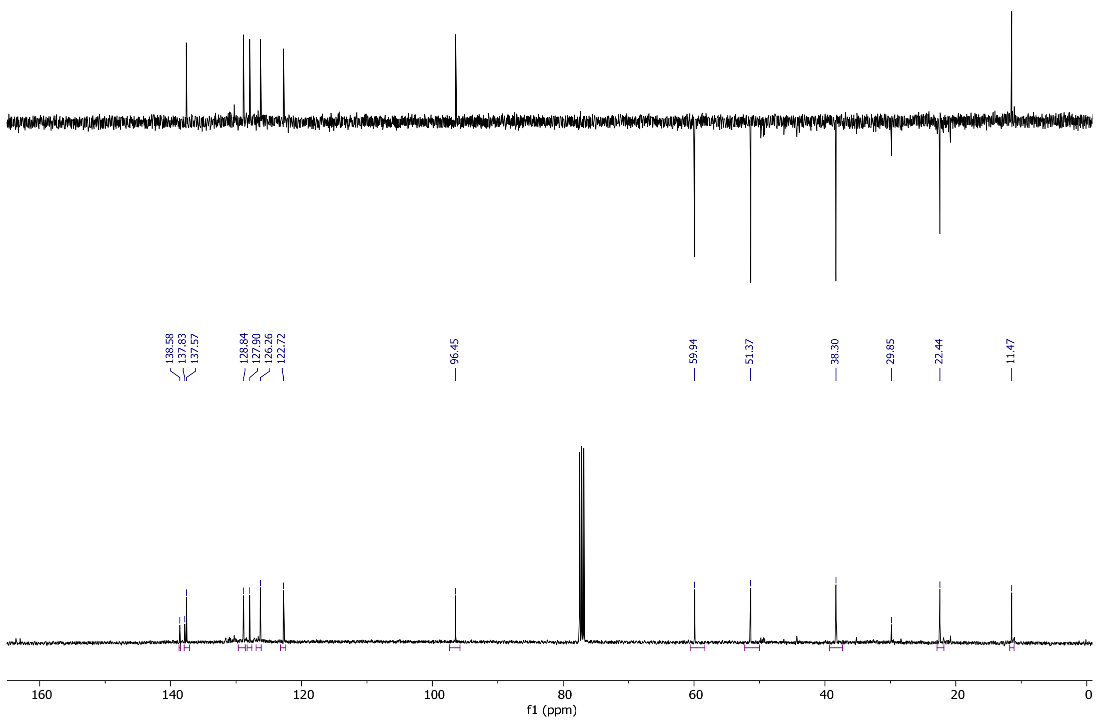

3-Methyl-1,5-dihydro-2*H*-benzo[*d*]azepine-2,4(3*H*)-dione (**10**)

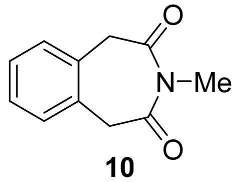

10

^{1}H NMR (500 MHz, $CDCl_3$)

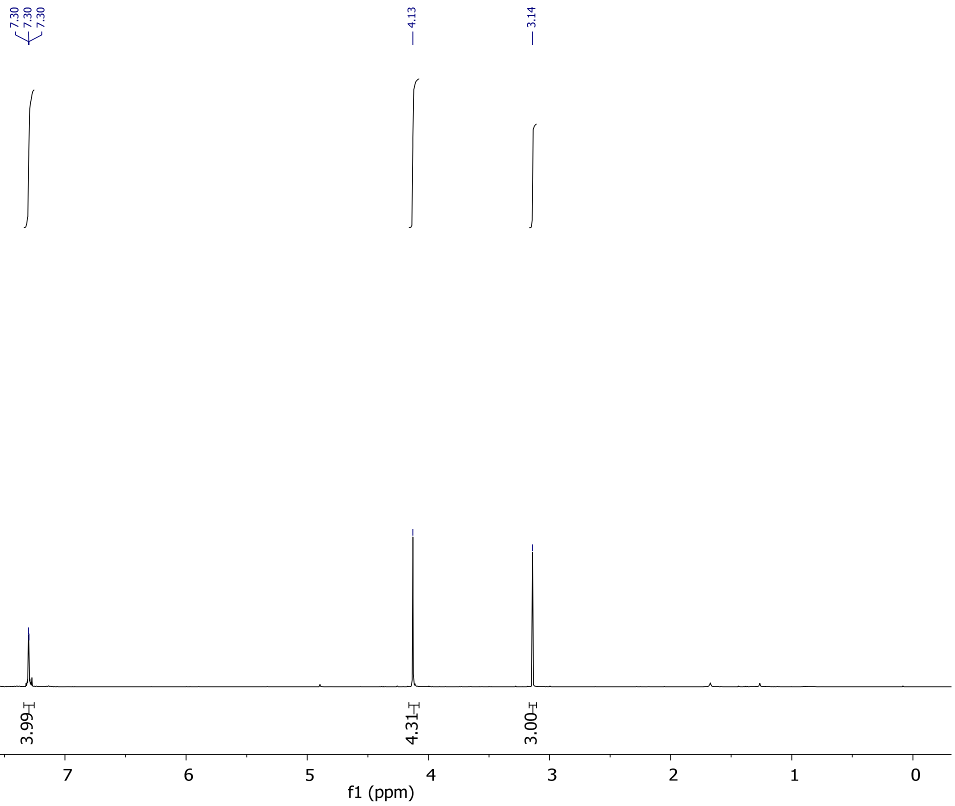

^{13}C{1H} NMR, DEPT (126 MHz, $CDCl_3$)

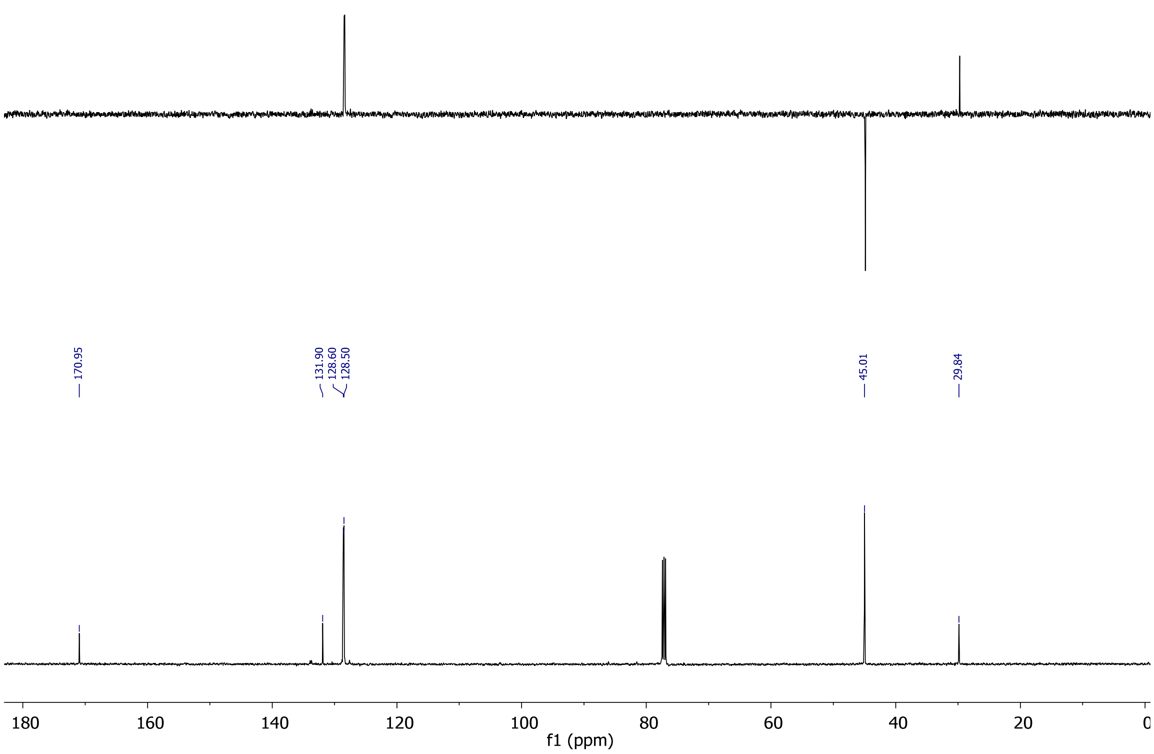

Isoquinolin-1(2H)-one (**11**)

^{1}H NMR (300 MHz, $CDCl_3$)

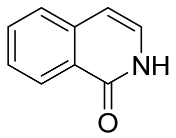

11

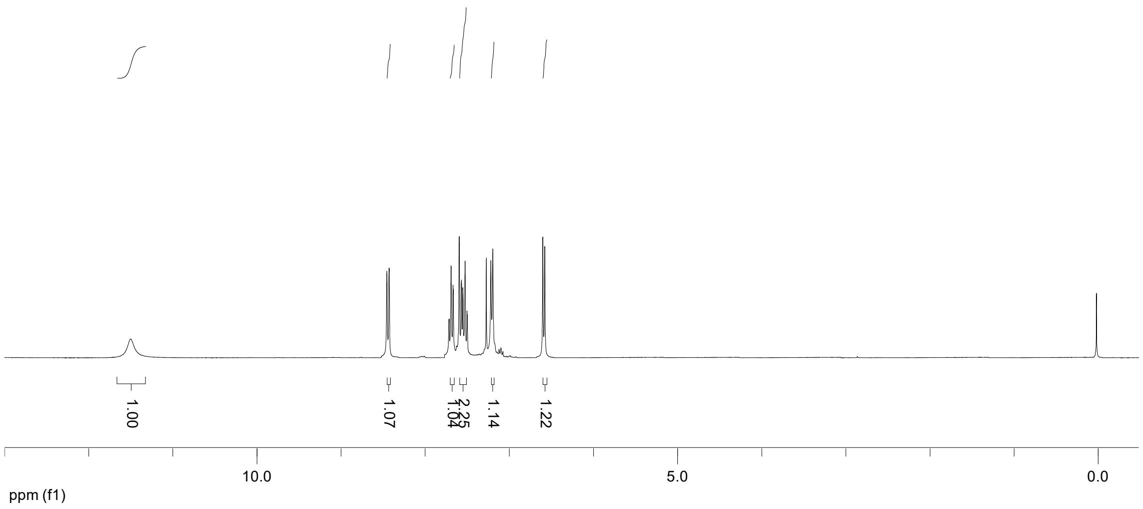

^{13}C{1H} NMR, DEPT (75 MHz, $CDCl_3$)

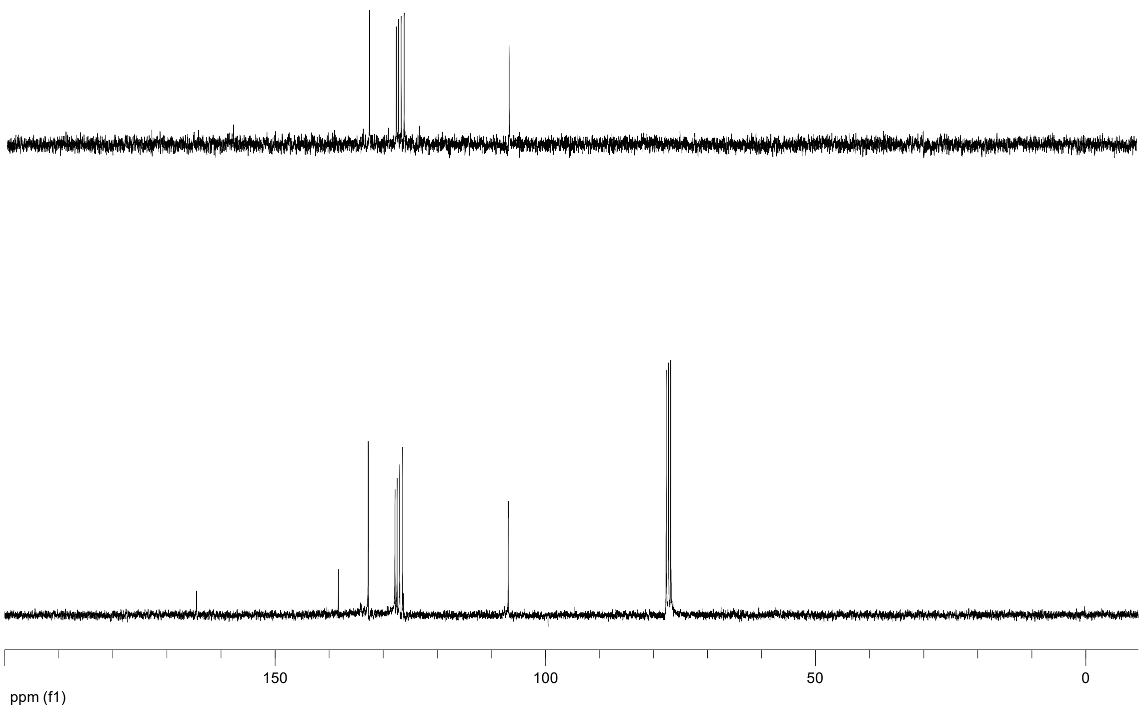

9. Computational details

Theoretical calculations were performed at DFT level of theory using Gaussian16 software.[16] The structures of all the intermediates and transition states were optimized in dichlorethane solvent with the SMD continuum model[17] using the B3LYP functional[18] combined with the Grimme's D3 correction for dispersion.[19] Basis set BS1 was used for the optimizations. BS1 includes the 6-31G(d,p) basis set for the main group elements,[20] and the scalar relativistic Stuttgart-Dresden SDD pseudopotential and its associated double-ζ basis set,[21] complemented with a set of *f* polarization functions,[22] for the ruthenium atom. Frequency calculations were carried out for all the optimized geometries to characterize the stationary points as either minima or transition states. It was confirmed that transition states connect with the corresponding intermediates by usual intrinsic reaction coordinate (IRC) calculations and subsequent optimization to minima.

The final qh-Gibbs (quasi-rigid-rotor-harmonic-oscillator) energies in DCE at 373K and 1 M are obtained using GoodVibes software,[23] with a frequency cut-off of 100 cm^{-1},

---

[16] Gaussian 016 (Revision B.01): Frisch, M. J.; Trucks, G. W.; Schlegel, H. B.; Scuseria, G. E.; Robb, M. A.; Cheeseman, J. R.; Scalmani, G.; Barone, V.; Mennucci, B.; Petersson, G. A.; Nakatsuji, H.; Caricato, M.; Li, X.; Hratchian, H. P.; Izmaylov, A. F.; Bloino, J.; Zheng, G.; Sonnenberg, J. L.; Hada, M.; Ehara, M.; Toyota, K.; Fukuda, R.; Hasegawa, J.; Ishida, M.; Nakajima, T.; Honda, Y.; Kitao, O.; Nakai, H.; Vreven, T.; Montgomery, Jr., J. A.; Peralta, J. E.;Ogliaro, F.; Bearpark, M.; Heyd, J. J.; Brothers, E.; Kudin, K. N.; Staroverov, V. N.; Kobayashi, R.; Normand, J.; Raghavachari, K.; Rendell, A.; Burant, J. C.; Iyengar, S. S.; Tomasi, J.; Cossi, M.; Rega, N.; Millam, J. M.; Klene, M.; Knox, J. E.; Cross, J. B.; Bakken, V.; Adamo, C.; Jaramillo, J.; Gomperts, R.; Stratmann, R. E.; Yazyev, O.; Austin, A. J.; Cammi, R.; Pomelli, C.; Ochterski, J.W.; Martin, R. L.; Morokuma, K.; Zakrzewski, V. G.; Voth, G. A.; Salvador, P.; Dannenberg, J. J.; Dapprich, S.; Daniels, A. D.; Farkas, Ö.; Foresman, J. B.; Ortiz, J. V.; Cioslowski, J.; Fox, D. J., Gaussian, Inc., Wallingford CT, 2011.

[17] Marenich, A. V.; Cramer, C. J.; Truhlar, D. G. Universal Solvation Model Based on Solute Electron Density and on a Continuum Model of the Solvent Defined by the Bulk Dielectric Constant and Atomic Surface Tensions. *J. Phys. Chem. B* **2009**, *113*, 6378-6396.

[18] (a) Lee, C.; Yang, W.; Parr, R. G. Development of the Colle-Salvetti correlation-energy formula into a functional of the electron density. *Physical Review B* **1988**, *37*, 785-789. (b) Becke, A. D. Density-functional thermochemistry. III. The role of exact exchange. *J. Chem. Phys.* **1993**, *98*, 5648-5652.

[19] Grimme, S.; Antony, J.; Ehrlich, S.; Krieg, H. A consistent and accurate ab initio parametrization of density functional dispersion correction (DFT-D) for the 94 elements H-Pu. *J. Chem. Phys.* **2010**, *132*, 154104.

[20] (a) Hehre, W. J.; Ditchfield, R.; Pople, J. A. Self—Consistent Molecular Orbital Methods. XII. Further Extensions of Gaussian—Type Basis Sets for Use in Molecular Orbital Studies of Organic Molecules. *J. Chem. Phys.* **1972**, *56*, 2257-2261. (b) Francl, M. M.; Pietro, W. J.; Hehre, W. J.; Binkley, J. S.; Gordon, M. S.; DeFrees, D. J.; Pople, J. A. Self-consistent molecular orbital methods. XXIII. A polarization-type basis set for second-row elements. *J. Chem. Phys.* **1982**, *77*, 3654-3665.

[21] Andrae, D.; Häußermann, U.; Dolg, M.; Stoll, H.; Preuß, H. Energy-adjustedab initio pseudopotentials for the second and third row transition elements. *Theor. Chim. Acta* **1990**, *77*, 123-141.

[22] Ehlers, A. W.; Böhme, M.; Dapprich, S.; Gobbi, A.; Höllwarth, A.; Jonas, V.; Köhler, K. F.; Stegmann, R.; Veldkamp, A.; Frenking, G. A set of f-polarization functions for pseudo-potential basis sets of the transition metals Sc-Cu, Y-Ag and La-Au. *Chem. Phys. Lett.* **1993**, *208*, 111-114.

[23] Luchini, G.; Alegre-Requena, J. V.; Funes-Ardoiz, I.; Rodríguez-Guerra, J.; Chen, J.; Paton, R. S. (2019, July 23). bobbypaton/GoodVibes: GoodVibes v3.0.0 (Version v3.0.0). Zenodo.

adding the thermal and entropic corrections computed at the BS1 to the electronic energy computed at BS2 level. BS2 consists in the *def2*-TZVP basis set for the main group elements and the quadruple-ζ *def2*-QZVP basis set for Ru.[24] A correction of 2.37 kcal/mol was applied to all Gibbs energy values to change the standard state from the gas phase (1 atm) to solution (1 M) at 373.15 K.[25]

The final values were obtained as single point energies over the DFT optimized structures with coupled-cluster singles and doubles with perturbative triples method employing the domain based local pair natural orbitals approach [DLPNO-CCSD(T)].[26] combined with triple-ζ split valence basis set, def2-TZVP using ORCA package.[27] Entropic and enthalpic correction as well as solvation energies, used to compute the final Gibbs energy values, were considered employing the DFT values at B3LYP-D3/BS1 theory level.

All 3D chemical structures were prepared using CYLview20.[28]

[24] Weigend, F.; Furche, F.; Ahlrichs, R. Gaussian basis sets of quadruple zeta valence quality for atoms H–Kr. *J. Chem. Phys.* **2003**, *119*, 12753-12762.

[25] Bryantsev, V. S.; Diallo, M. S.; Goddard Iii, W. A. Calculation of Solvation Free Energies of Charged Solutes Using Mixed Cluster/Continuum Models. *J. Phys. Chem. B* **2008**, *112*, 9709-9719.

[26] (a) Riplinger, C.; Sandhoefer, B.; Hansen, A.; Neese, F. Natural triple excitations in local coupled cluster calculations with pair natural orbitals. *J. Chem. Phys.* **2013**, *139*, 134101. (b) Guo, Y.; Riplinger, C.; Becker, U.; Liakos, D. G.; Minenkov, Y.; Cavallo, L.; Neese, F. Communication: An improved linear scaling perturbative triples correction for the domain based local pair-natural orbital based singles and doubles coupled cluster method [DLPNO-CCSD(T)]. *J. Chem. Phys.* **2018**, *148*, 011101.

[27] (a) Neese, F. The ORCA program system. *Wiley Interdiscip. Rev.: Comput. Mol. Sci.* **2012**, *2*, 73-78. (b) Neese, F. Software update: the ORCA program system, version 4.0. *Wiley Interdiscip. Rev.: Comput. Mol. Sci.* **2018**, *8*, e1327.

[28] C. Y. Legault, CYLview20, **2020** (http://www.cylview.org).

10.Complete Gibbs energy profiles for all calculated pathways

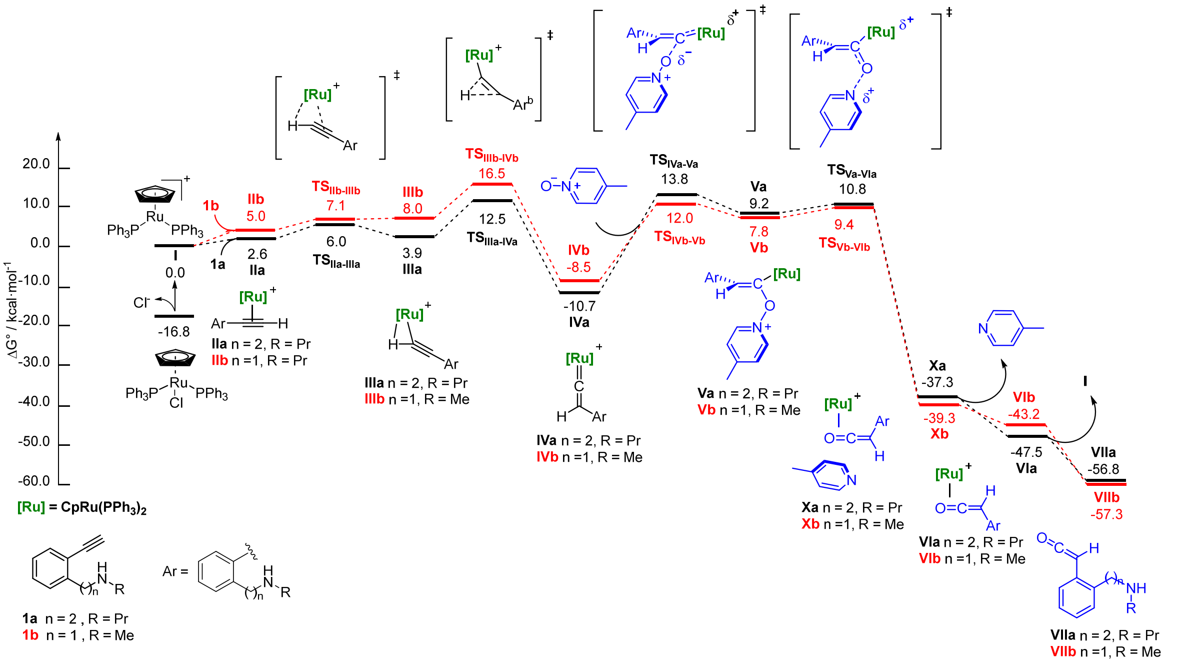

Figure S1. Complete Gibbs energy profile (B3LYP-D3/def2-TZVP- def2-QZVP(Ru)DCE(SMD)//B3LYP-D3/6-31G(d,p)-SDD(Ru) DCE(SMD) 373 K) computed for the Ru(II)-catalyzed transformation of **1a** (black) and **1b** (red) to aminoketenes **VIIa** and **VIIb**, respectively. Energies are relative to $[CpRu(PPh_3)_2]^+$ **I** and alkynylamines **1a** and **1b**, respectively, and are mass balanced.

Three different pathways for the metal-free lactamization of ketenes **VIIa** and **VIIb** to 3-benzazepinone **2a** and dihydroisoquinolinone **2b** (Figures S2 and S3, respectively) were computed: i) N-H addition to the C=C bond of the ketene (green), ii) N-H addition to the C=O ketene bond (purple) and iii) nucleophilic addition of the amine to the ketene favored by water acting as proton shuttle (black).

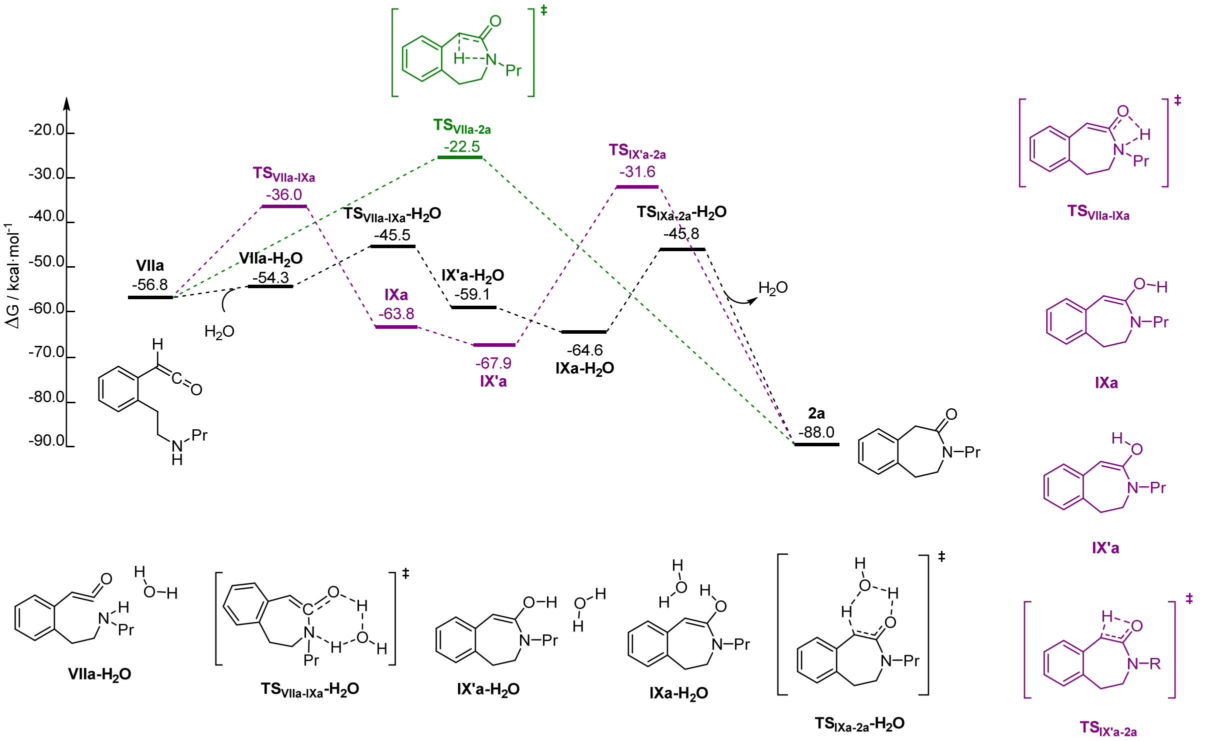

Figure S2. Complete Gibbs energy profiles (B3LYP-D3/def2-TZVP- def2-QZVP(Ru)DCE(SMD)//B3LYP-D3/6-31G(d,p)-SDD(Ru) DCE(SMD) 373 K) computed for the lactamization of free ketene **VIIa** to 3-benzazepinone **2a**. Energies are relative to $[CpRu(PPh_3)_2]^+$ **I** and alkynylamine **1a** and are mass balanced.

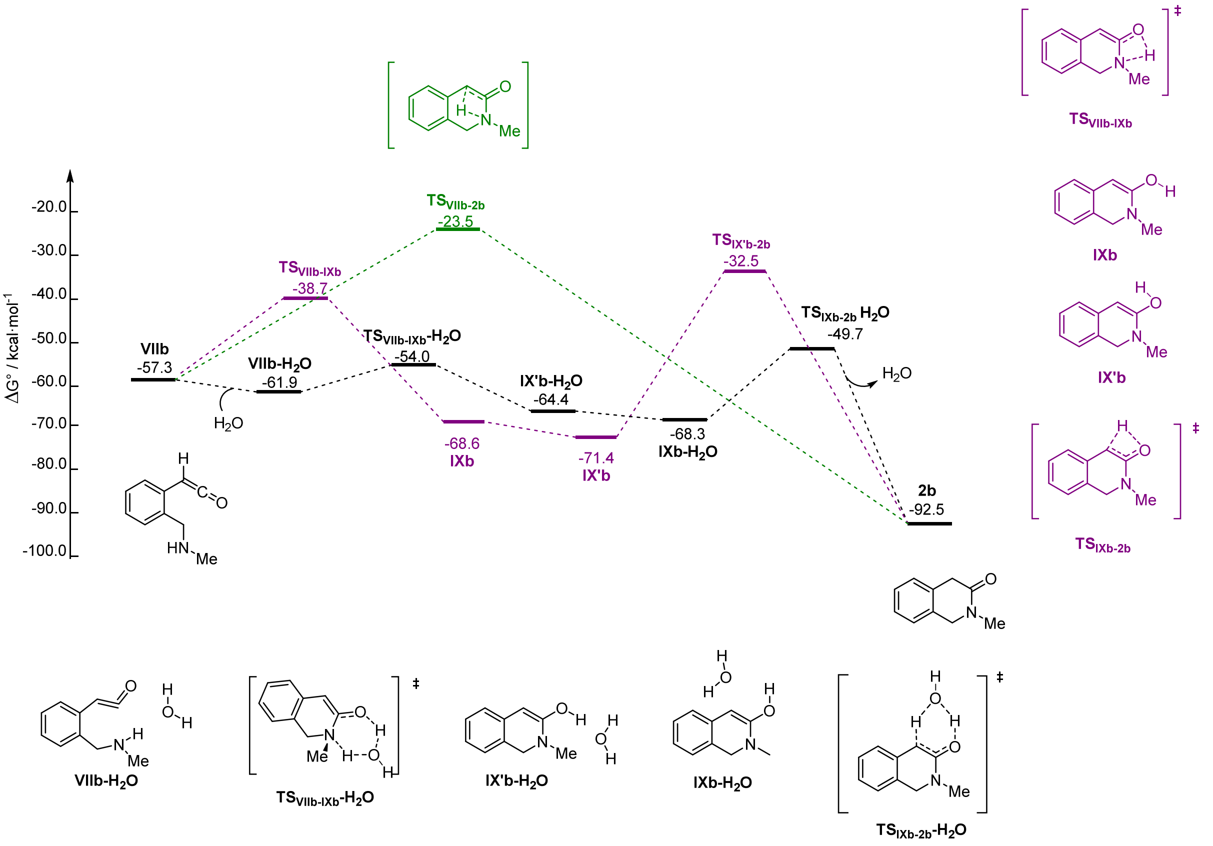

Figure S3. Complete Gibbs energy profiles (B3LYP-D3/def2-TZVP- def2-QZVP(Ru)DCE(SMD)//B3LYP-D3/6-31G(d,p)-SDD(Ru) DCE(SMD) 373 K) computed for the lactamization of free ketene **VIIb** to dihydroisoquinolinone **2b**. Energies are relative to $[CpRu(PPh_3)_2]^+$ **I** and alkynylamine **1b** and are mass balanced.

Metal-bound lactamization pathway of ruthenium-ketene complexes **VIa** and **VIb** was computed and compared with the more stable metal-free lactamization pathway (green and black pathways, respectively in Figures S4 and S5). The Metal-bound lactamization pathway proceeds in a stepwise manner beginning with a η^2/η^1 coordination rearrangement of complexes **VIa** and **VIb** ($\Delta G^{\ddagger}_{\mathbf{VIa\text{-}XIVa}}$= 8.6 kcal·mol^{-1}; $\Delta G^{\ddagger}_{\mathbf{VIb\text{-}XIVb}}$ = 5.6 kcal·mol-1) to complexes **XIVa** and **XIVb**, followed by the formation of the C-N bond giving rise to intermediates **XV**.

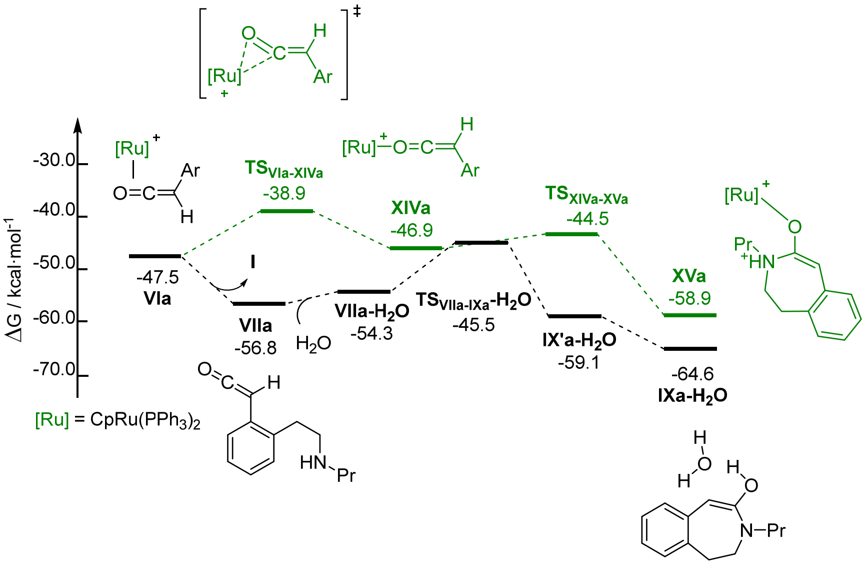

Figure S4. Gibbs energy profiles (B3LYP-D3/def2-TZVP- def2-QZVP(Ru)DCE(SMD)//B3LYP-D3/6-31G(d,p)-SDD(Ru) DCE(SMD) 373 K) for metal-free (black) and metal-bound (green) lactamization processes from intermediate **VIa**. Energies are relative to $[CpRu(PPh_3)_2]^+$ **I** and alkynylamine **1a** and are mass balanced.

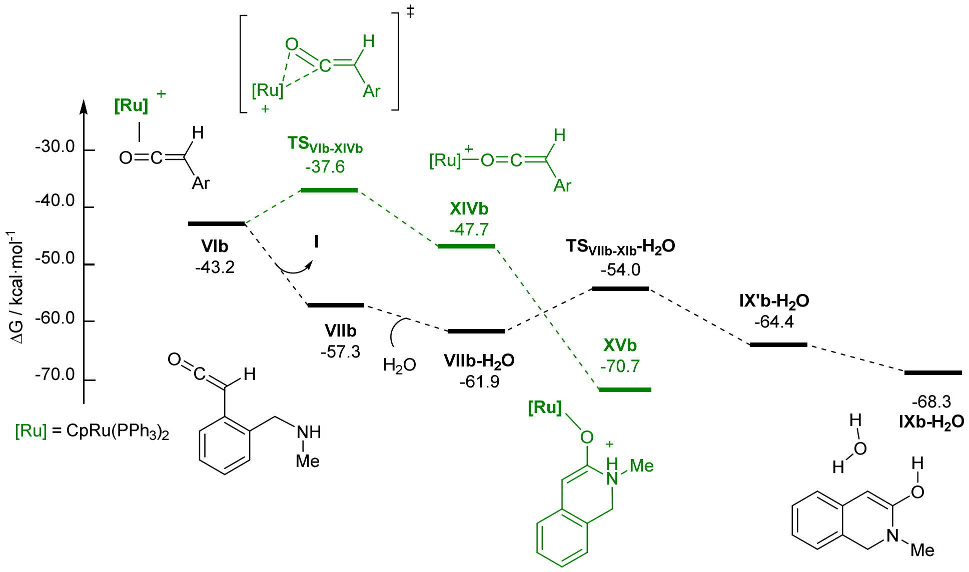

Figure S5. Gibbs energy profiles (B3LYP-D3/def2-TZVP- def2-QZVP(Ru)DCE(SMD)//B3LYP-D3/6-31G(d,p)-SDD(Ru) DCE(SMD) 373 K) for metal-free (black) and metal-bound (green) lactamization processes from intermediate **VIb**. Energies are relative to $[CpRu(PPh_3)_2]^+$ **I** and alkynylamine **1b** and are mass balanced.

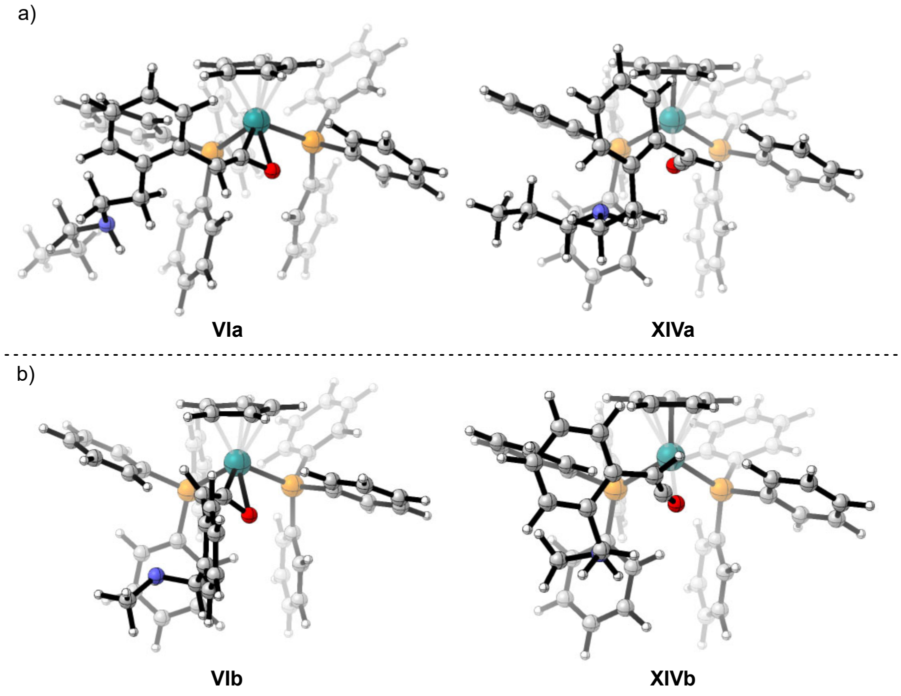

Figure S6. 3D models for η^2- **VI** and η^1- **XIV** modes of coordination of ruthenium to the ketenes obtained from a) alkynylamine **1a** and b) alkynylamine **1b**.

To confirm which pathway is the prevalent, metal-free lactamization from **VI** to **2** and metal-bound lactamization from **VI** to **XV**, a microkinetic analysis using COPASI[29] was performed (Figure S7). The analysis was done considering the most unfavourable situation with no free amine **1** present to trap the catalyst **I** released in the first step of the metal-free lactamization pathway (step **VI** to **VII** + **I** reversible). According to this analysis, the metal-bound pathway can be ruled out since all the final product is formed through the metal-free pathway (only for **VIa** a negligible 6% formation of **XVa** was observed.

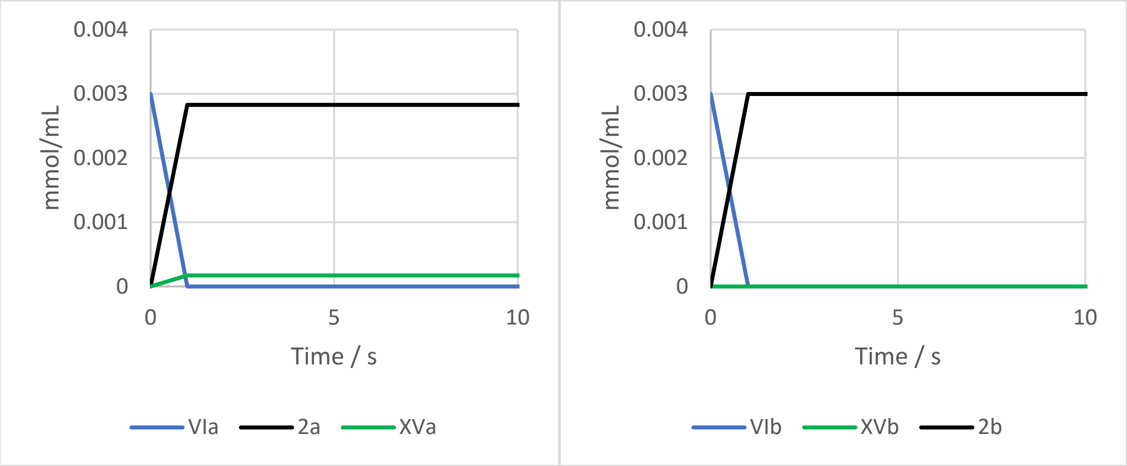

Figure S7. Microkinetic simulation for the evolution of intermediates **VIa** (left) and **VIb** (right) through metal-free and metal-bound pathways as a function of concentration and time (B3LYP-D3/def2-TZVP- def2-QZVP(Ru)DCE(SMD)//B3LYP-D3/6-31G(d,p)-SDD(Ru) DCE(SMD) 373 K) .

For the intramolecular Ru(II)-catalyzed hydroamination of Ru(II)-vinylidene species **IVa** and **IVb** (Figure S8), intramolecular protonolysis of vinyl-ruthenium species **VIIIa** and **VIIIb** (Figure S8, grey and pink pathways, respectively) were also computed, finding activation barriers too high, around 40 kcal mol^{-1}, compare with intermolecular pathways using HCO_3^- as base (Figure S8, black and red pathways, respectively).

[29] (a) COPASI 4.33, (2020) Build 246. http://copasi.org/. (b) Hoops, S.; Sahle, S.; Gauges, R.; Lee, C.; Pahle, J.; Simus, N.; Singhal, M.; Xu, L.; Mendes, P.; Kummer, U. COPASI—a COmplex PAthway SImulator. *Bioinformatics* **2006**, *22*, 3067-3074.

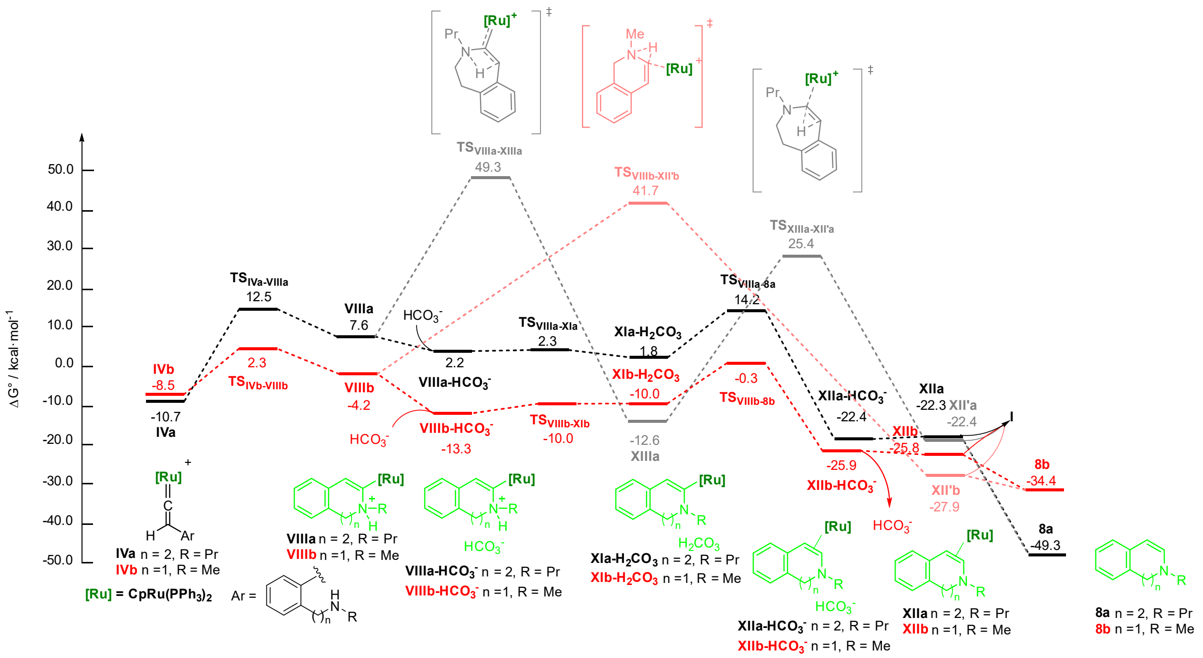

Figure S8. Complete Gibbs energy profile (B3LYP-D3/def2-TZVP- def2-QZVP(Ru)DCE(SMD)//B3LYP-D3/6-31G(d,p)-SDD(Ru) DCE(SMD) 373 K) computed for the intramolecular Ru(II)-catalyzed hydroamination of Ru(II)-vinylidene species **IVa** (intermolecular protonolysis: black; intramolecular protonolysis: grey) and **IVb** (intermolecular protonolysis: red; intramolecular protonolysis: pink) to dihydrobenzazepine **8a** and dihydroisoquinoline **8b**, respectively. Energies are relative to $[CpRu(PPh_3)_2]^+$ **I** and alkynylamines **1a** and **1b**, respectively, and are mass balanced.

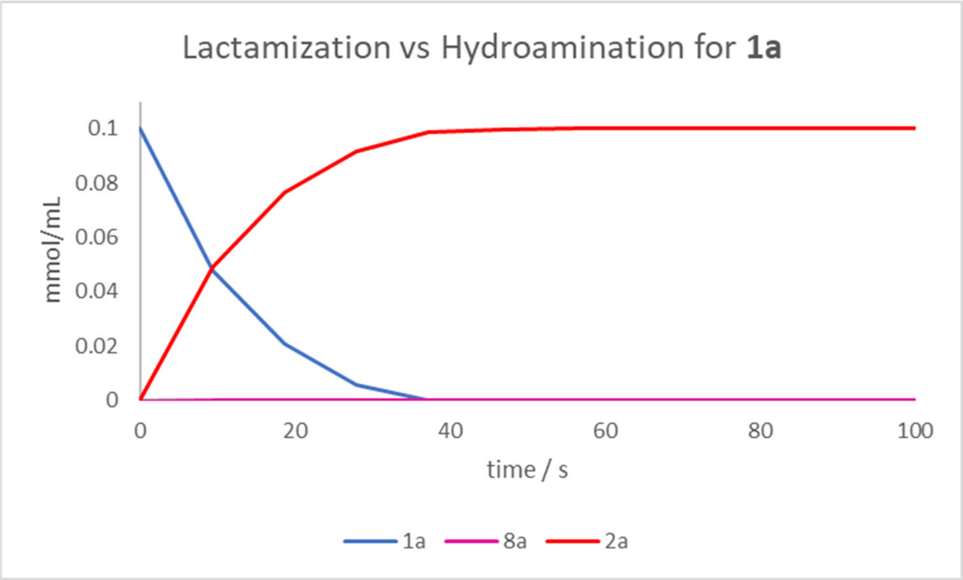

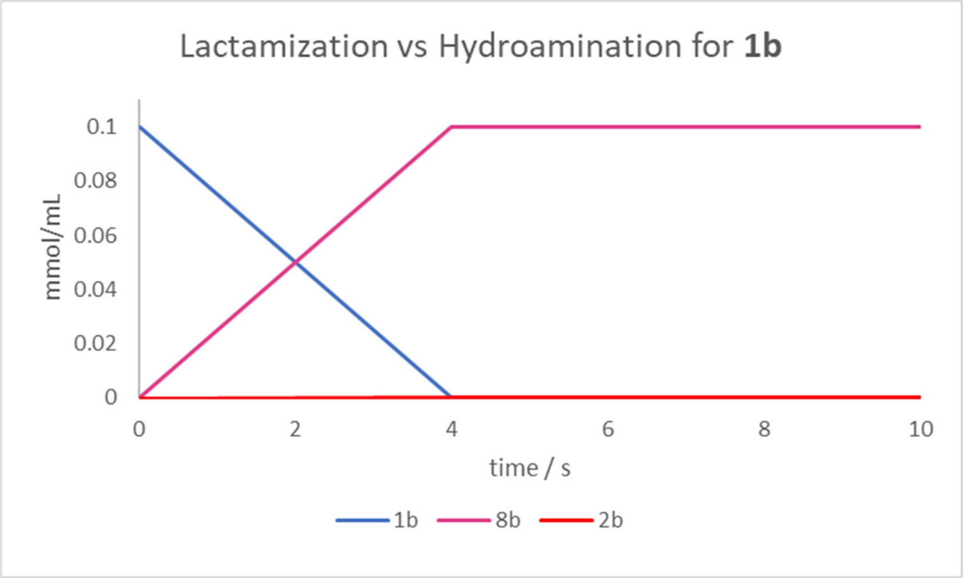

Figure S9. Microkinetic simulation for the evolution of 1,5-alkynylamine **1a** (up) and 1,4-alkynylamine **1b** (down) as a function of concentration and time (DLPNO-CCSD(T)/def2-TZVP//B3LYP-D3/6-31G(d,p)-SDD(Ru)$_{DCE(SMD)}$ 373 K).

11. Thermal rate constants k (373 K) for steps involving the most significant transitions states for microkinetic simulations

The thermal rate coefficient, $k_i(T)$, for each process i presenting a barrier is calculated according to transition state theory:

$$k_i(T) = \sigma \frac{k_B T}{h} \left(\frac{RT}{P_0}\right)^{\Delta n} e^{-\frac{\Delta G_i^{\ddagger}}{RT}}$$

Where σ is the reaction path degeneracy, T is the temperature (373 K), h is Planck's constant, $\Delta G^{\ddagger}_i$ is the free energy of activation, p_0 is 1 bar and Δn = 1 (0) for bimolecular (unimolecular) reactions.

Following the approach of Harvey and co-workers,[30] the barrierless associative reactions are assumed to be diffusion controlled, and their thermal rate coefficients, $k_{diff}(T)$, are calculated by the following equation:

$$k_{diff}(T) = \frac{8k_B T}{3\eta}$$

[30] Rush, L. E.; Pringle, P. G.; Harvey, J. N. Computational Kinetics of Cobalt-Catalyzed Alkene Hydroformylation. *Angew. Chem. Int. Ed.* **2014**, *53*, 8672-8676.

where η is the viscosity of the solvent (dichloroethane). The rate for the corresponding reverse dissociative process is calculated using detailed balance.

Table S1. Thermal rate constants k (373 K) used for microkinetic simulations of evolution of 1a

Step	k_{direct} (373 K)	$k_{reverse}$ (373 K)
**I** + **1a** ⇆ **IIa**	1.03E+10 M^{-1} s^{-1}	4.01E+08 s^{-1}
**IIa** ⇆ **IVa**	6.82E+04 s^{-1}	2.32E-00 s^{-1}
**IVa** + **4-pic *N*-oxide** → **VIIa** + **I** + **4-picoline**	2.13E+01 M^{-1} s^{-1}	-
**VIIa** + **H_2O** ⇆ **IXa-H_2O**	1.61E+07 M^{-1} s^{-1}	3.98E+03 s^{-1}
**IXa-H_2O** → **2a** + **H_2O**	1.35 s^{-1}	-
**IVa** ⇆ **VIIIa**	7.87E-04 s^{-1}	8.94E+5 s^{-1}
**VIIIa** + **HCO_3^-** ⇆ **8a** + **I** + **HCO_3^-**	4.17E+06 M^{-1} s^{-1}	-

The initial amounts used in COPASI for the microkinetic simulations were:

1a: 0.1 M

I: 0.003 M

4-Pic N oxide: 0.11 M

H_2O: 0.0003 M

HCO_3^-: 0.03 M

Total volume: 2 mL

Table S2. Thermal rate constants k (373 K) used for microkinetic simulations of evolution of 1b

Step	K_{direct} (373 K)	$k_{reverse}$ (373 K)
**I** + **1b** ⇆ **IIb**	1.03E+10 M^{-1} s^{-1}	7.89E+09 s^{-1}
**IIb** ⇆ **IVb**	1.17E+05 s^{-1}	1.77E-02 s^{-1}
**IVb** + **4-pic *N*-oxide** ⇆ **VIIb** + **I** + **4-picoline**	3.65E+02 M^{-1} s^{-1}	-
**VIIb** + **H_2O** ⇆ **IXb-H_2O**	1.84E+10 M^{-1} s^{-1}	1.03E+04 s^{-1}
**IXb-H_2O** → **2b** + **H_2O**	1.18 s^{-1}	-

**IVb ⇆ VIIIb**	2.02E+04 s^{-1}	2.202E+05 s^{-1}
**VIIIb + HCO_3^- ⇆ 8b** + **I** + **HCO_3^-**	1.34E+06 M^{-1} s^{-1}	-

The initial amounts used in COPASI for the mirokinetic simulations were:

1b: 0.1 M

I: 0.003 M

4-Pic *N* oxide: 0.11 M

H_2O: 0.0003 M

HCO_3^-: 0.03 M

Total volume: 2 mL

Table S3. Thermal rate constants k (373 K) used for microkinetic simulations of evolution of VIa

Step	K_{direct} (373 K)	$k_{reverse}$ (373 K)
**VIa ⇆ VIIa + I**	3.04E+15 s^{-1}	1.03E+10 M^{-1} s^{-1}
**VIa ⇆ XIVa**	1.02E+9 s^{-1}	2.30E+9 s^{-1}
**VIIa + H_2O ⇆ VIIa-H_2O**	1.03E+10 M^{-1} s^{-1}	3.05E+11 s^{-1}
**VIIa-H_2O ⇆ IXa-H_2O**	5.20E+07 s^{-1}	4.56E+01 s^{-1}
**IXa-H_2O → 2a** + **H_2O**	6.84E+01 s^{-1}	-
**XIVa →XVa**	3.01E+11	-

The initial amounts used in COPASI for the mirokinetic simulations were:

VIa: 0.003 M

H_2O: 0.0003 M

Total volume: 2 mL

Table S4. Thermal rate constants k (373 K) used for microkinetic simulations of evolution of VIb

Step	K_{direct} (373 K)	$k_{reverse}$ (373 K)
**VIb** ⇆ **VIIb + I**	2.02E+18 s^{-1}	1.03E+10 M^{-1} s^{-1}
**VIb** → **XVb**	3.96E+9 s^{-1}	-
**VIIb** + **H_2O** ⇆ **VIIb-H_2O**	1.03E+10 M^{-1} s^{-1}	2.04E+07 s^{-1}
**VIIb-H_2O** ⇆ **IXb-H_2O**	1.76E+08 s^{-1}	3.03E+04 s^{-1}
**IXb-H_2O** → **2b** + **H_2O**	8.97E+01 s^{-1}	-

The initial amounts used in COPASI for the mirokinetic simulations were:

VIb: 0.003 M

H_2O: 0.0003 M

Total volume: 2 mL

12. B3LYP energy values

Common species										
Structure	BS1						BS2		ΔG (kcal/mol) corrected with BS2	ΔG (kcal/mol) correction 1 atm to 1 M
	E (a.u.)	Negative frequencies / cm^{-1}	ZPE (a.u.)	H_therm (a.u.)	G_therm (a.u.)	G (a.u.)	E (a.u.)	G (a.u.) corrected		
**CpRu(PPh_3)$_2$Cl**	-2821.564123	-	0.639408	0.702302	0.534600	-2821.029523	-2822.231762	-2821.697162	**-14.5**	-16.8
**I**	-2361.167324	-	0.638064	0.698603	0.534654	-2360.63267	-2361.799849	-2361.265195	**0.0**	0.0
**4-pic *N*-oxide**	-362.791043	-	0.120515	0.132073	0.078155	-362.712888	-362.934007	-362.855852	-	-
**4-picoline**	-287.625740	-	0.116348	0.126607	0.075776	-287.549964	-287.729897	-287.654121	-	-
**HCO_3^-**	-264.513904	-	0.026324	0.032421	-0.007239	-264.521143	-264.662056	-264.669295	-	-
**Cl^-**	-460.349807	-	0	0.002954	-0.019465	-460.369272	-460.389465	-460.408930	-	-
**H_2CO_3**	-265.010764	-	0.039395	0.045882	0.005579	-265.005185	-265.137514	-265.131935	-	-
**H_2O**	-76.425073	-	0.02113	0.025870	-0.002040	-76.427113	-76.470540	-76.472580	-	-

ε-lactam (2a)										
Structure	BS1						BS2		ΔG (kcal/mol) corrected with BS2	ΔG (kcal/mol) correction 1 atm to 1 M
	E (a.u.)	Negative frequencies / cm^{-1}	ZPE (a.u.)	H_therm (a.u.)	G_therm (a.u.)	G (a.u.)	E (a.u.)	G (a.u.) corrected		
**1a**	-560.357100	-	0.268943	0.291909	0.210582	-560.146518	-560.556124	-560.345542	**-**	**-**
**IIa**	-2921.573051	-	0.911840	0.994833	0.786070	-2920.786981	-2922.388937	-2921.602867	**4.9**	**2.6**
**TS$_{IIa-IIIa}$**	-2921.559920	-219.7	0.909026	0.992174	0.780173	-2920.779747	-2922.377567	-2921.597394	**8.4**	**6.0**
**IIIa**	-2921.562372	-	0.909733	0.993171	0.780358	-2920.782014	-2922.381199	-2921.600841	**6.2**	**3.9**
**TS$_{IIIa-IVa}$**	-2921.547830	-688.2	0.906476	0.989813	0.777732	-2920.770098	-2922.364797	-2921.587065	**14.9**	**12.5**
**IVa**	-2921.590432	-	0.911336	0.994468	0.781192	-2920.809240	-2922.405186	-2921.623994	**-8.3**	**-10.7**
**TS$_{IVa-Va}$**	-3284.387123	-131.4	1.035693	1.129441	0.893544	-3283.493579	-3285.330679	-3284.437135	**18.5**	**13.8**
**Va**	-3284.395270	-	1.036290	1.130626	0.894632	-3283.500638	-3285.339095	-3284.444463	**13.9**	**9.2**
**TS$_{Va_VIa}$**	-3284.392808	-461.7	1.033968	1.128315	0.892492	-3283.500316	-3285.334367	-3284.441875	**15.5**	**10.8**
**Xa**	-3284.464818	-	1.034746	1.130095	0.892302	-3283.572516	-3285.410845	-3284.518543	**-32.6**	**-37.3**
**VIa**	-2996.813292	-	0.916672	1.000645	0.781944	-2996.031348	-2997.666360	-2996.884416	**-45.1**	**-47.5**
**VIIa**	-635.618510	-	0.274017	0.298142	0.213488	-635.405022	-635.851277	-635.637789	**-56.8**	**-56.8**
**Metal-free Lactamization. VIa to 2a**										
**TS$_{VIIa-IXa}$**	-635.596645	-1728.9	0.273229	0.294863	0.218662	-635.377983	-635.823366	-635.604704	**-36.0**	**-36.0**
**IXa**	-635.643253	-	0.277393	0.299640	0.221919	-635.421334	-635.871211	-635.649292	**-63.8**	**-63.8**
**IX'a**	-635.648588	-	0.277282	0.299726	0.220929	-635.427659	-635.876449	-635.655520	**-67.9**	**-67.9**

**$TS_{IX'a\text{-}2a}$**	-635.588085	-1950.8	0.272219	0.294024	0.217220	-635.370865	-635.814826	-635.597606	**-31.6**	-31.6
**TS_{VIIa_2a}**	-635.573496	-1883.7	0.271708	0.293533	0.216979	-635.356517	-635.800148	-635.583169	**-22.5**	-22.5
**VIIa-H_2O**	-712.079481	-	0.303917	0.329602	0.243815	-711.835666	-712.346485	-712.102670	**-52.0**	-54.3
**$TS_{VIIaIXa}$-H_2O**	-712.063466	-1307.5	0.297355	0.321532	0.239218	-711.824248	-712.327910	-712.088692	**-43.2**	-45.5
**IX'a-H_2O**	-712.087069	-	0.302570	0.328654	0.241884	-711.845185	-712.352181	-712.110297	**-56.8**	-59.1
**IXa-H_2O**	-712.091572	-	0.301901	0.328547	0.238940	-711.852632	-712.358077	-712.119137	**-62.3**	-64.6
**TS_{IXa_2a}-H_2O**	-712.062939	-1748.8	0.295813	0.320231	0.237047	-711.825892	-712.326079	-712.089032	**-43.4**	-45.8
**2a**	-635.68	-	0.278329	0.300293	0.221410	-635.459975	-635.908896	-635.687486	**-88.0**	-88.0
**Metal-bound Lactamization. VIa to XVa**										
**$TS_{VIa\text{-}XIVa}$**	-2996.808287	-168.90	0.915181	0.999106	0.787150	-2996.021137	-2997.657899	-2996.870749	**-38.9**	-38.9
**XIVa**	-2996.813725	-	0.915218	1.000296	0.781682	-2996.032043	-2997.665139	-2996.883457	**-46.9**	-46.9
**$TS_{XIVa\text{-}XVa}$**	-2996.813470	-14.6	0.915144	0.998927	0.785048	-2996.028422	-2997.664718	-2996.879670	**-44.5**	-44.5
**XVa**	-2996.849983	-	0.920968	1.003656	0.794179	-2996.055804	-2997.696762	-2996.902583	**-58.9**	-58.9
**Hydroamination. IVa to 2a**										
**$TS_{IVa\text{-}VIIIa}$**	-2921.567683	-70.3	0.913862	0.994898	0.792129	-2920.775554	-2922.379290	-2921.587161	**14.8**	12.5
**VIIIa**	-2921.585292	-	0.920483	1.000687	0.801366	-2920.783926	-2922.396199	-2921.594833	**10.0**	7.6
**VIIIa-HCO_3^-**	-3186.146984		0.945774	1.033322	0.816802	-3185.330182	-3187.085831	-3186.269029	**6.9**	2.2
**$TS_{VIIIa\text{-}XIa}$**	-3186.136235	-518.2	0.940002	1.027471	0.808553	-3185.327682	-3187.077294	-3186.268741	**7.1**	2.3
**XIa-H_2CO_3**	-3186.139839	-	0.942252	1.030287	0.810194	-3185.329645	-3187.079776	-3186.269582	**6.6**	1.8
**$TS_{VIIIa\text{-}8a}$**	-3186.120447	-1343.2	0.938124	1.026021	0.806660	-3185.313787	-3187.056617	-3186.249957	**18.9**	14.2
**XIIa-HCO_3^-**	-3186.168298	-	0.941398	1.031164	0.799056	-3185.369242	-3187.111004	-3186.311948	**-20.0**	-22.4

**XIIa**	-2921.617195	-	0.914991	0.996859	0.789273	-2920.827922	-2922.431841	-2921.642568	**-20.0**	-22.3
**TS$_{VIIa\text{-}XIIIa}$**	-2921.50539	-1935.3	0.90914	0.990333	0.786264	-2920.719126	-2922.314735	-2921.528471	**51.6**	49.3
**XIIIa**	-2921.611455	-	0.916641	0.997583	0.794086	-2920.817369	-2922.421171	-2921.627085	**-10.3**	-12.6
**TS$_{XIIIa\text{-}XII'a}$**	-2921.542055	-1151.2	0.911823	0.992785	0.789893	-2920.752162	-2922.356431	-2921.566538	**27.7**	25.4
**XII'a**	-2921.618235	-	0.915717	0.997441	0.790104	-2920.828131	-2922.432722	-2921.642618	**-20.0**	-22.4
**2a**	-560.423409	-	0.272884	0.293663	0.218645	-560.204764	-560.6159	-560.397255	**-46.9**	-49.3

**δ-lactam (2b)**										
**Structure**	**BS1**						**BS2**		**ΔG (kcal/mol) corrected with BS2**	**ΔG (kcal/mol) correction 1 atm to 1 M**
	**E (a.u.)**	**Negative frequencies / cm^{-1}**	**ZPE (a.u.)**	**H_therm (a.u.)**	**G_therm (a.u.)**	**G (a.u.)**	**E (a.u.)**	**G (a.u.) corrected**		
**1b**	-442.397283	-	0.18359	0.200487	0.134812	-442.26	-442.558899	-442.424087	**-**	-
**IIb**	-2803.603772	-	0.82623	0.903538	0.706656	-2802.897116	-2804.384272	-2803.677616	**7.3**	5.0
**TS$_{IIb\text{-}IIIb}$**	-2803.592678	-108.2	0.822876	0.900260	0.700320	-2802.892358	-2804.374529	-2803.674209	**9.5**	7.1
**IIIb**	-2803.594816	-	0.825252	0.902263	0.704936	-2802.889880	-2804.377727	-2803.672791	**10.3**	8.0
**TS$_{IIIb\text{-}IVb}$**	-2803.582504	-593.4	0.823854	0.900485	0.703992	-2802.878512	-2804.363320	-2803.659328	**18.8**	16.5
**IVb**	-2803.627176	-	0.826246	0.903092	0.706942	-2802.920234	-2804.406053	-2803.699111	**-6.2**	-8.5
**TS$_{IVb\text{-}Vb}$**	-3166.425221	-141.3	0.949147	1.037391	0.816021	-3165.609200	-3167.334524	-3166.518503	**16.7**	12.0
**Vb**	-3166.437300	-	0.951263	1.039331	0.820388	-3165.616912	-3167.345537	-3166.525149	**12.5**	7.8
**TS$_{Vb\text{-}VIb}$**	-3166.429160	-464.6	0.94782	1.036458	0.813778	-3165.615382	-3167.336369	-3166.522591	**14.1**	9.4

**Xb**	-3166.503347	-	0.949335	1.038873	0.813224	-3165.690123	-3167.413402	-3166.600178	**-34.5**	**-39.3**
**VIb**	-2878.856944	-	0.949335	1.038873	0.713005	-2878.143939	-2879.669099	-2878.956094	**-40.8**	**-43.2**
**VIIb**	-517.658964	-	0.188521	0.206659	0.137190	-517.521774	-517.854422	-517.717232	**-57.4**	**-57.3**
**Metal-free Lactamization. VIIb to 2b**										
**TS$_{VIIb-IXb}$**	-517.638487	-1756.2	0.187395	0.203083	0.141320	-517.497167	-517.828917	-517.687597	**-38.7**	**-38.7**
**IXb**	-517.687766	-	0.191345	0.207792	0.144167	-517.543599	-517.879333	-517.735166	**-68.6**	**-68.6**
**IX'b**	-517.690646	-	0.190919	0.207637	0.143393	-517.547253	-517.88	-517.74	**-71.4**	**-71.4**
**TS$_{IX'b-2b}$**	-517.624447	-1939.6	0.185449	0.201705	0.138242	-517.486205	-517.815914	-517.677672	**-32.5**	**-32.5**
**TS$_{VIIb-2b}$**	-517.610704	-1927.4	0.185603	0.201913	0.138248	-517.472456	-517.801684	-517.663436	**-23.5**	**-23.5**
**VIIb-H_2O**	-594.125531	-	0.217149	0.237631	0.164191	-593.961340	-594.357562	-594.193371	**-59.6**	**-61.9**
**TS$_{VIIb-IXb}$-H_2O**	-594.114697	-1469.3	0.21105	0.229356	0.161872	-593.952825	-594.342681	-594.180809	**-51.7**	**-54.0**
**IX'b-H_2O**	-594.133514	-	0.216752	0.236754	0.164892	-593.968622	-594.362294	-594.197402	**-62.1**	**-64.4**
**IXb-H_2O**	-594.134818	-	0.215691	0.236604	0.161843	-593.972975	-594.365367	-594.203524	**-65.9**	**-68.3**
**TS$_{IXb-2b}$-H_2O**	-594.105566	-1682.9	0.2096	0.228486	0.158689	-593.946877	-594.332602	-594.173913	**-47.4**	**-49.7**
**2b**	-517.724680	-	0.191461	0.208037	0.143143	-517.581537	-517.916459	-517.773316	**-92.5**	**-92.5**
**Metal-bound Lactamization. VIb to XVb**										
**TS$_{VIb-XIVb}$**	-2878.843395	-209.2	0.829986	0.907792	0.70993	-2878.133468	-2879.657142	-2878.947215	**-37.6**	**-37.6**
**XIVb**	-2878.853976	-	0.829308	0.908445	0.70514	-2878.1488350	-2879.668413	-2878.963272	**-47.7**	**-47.7**
**XVb**	-2878.897257	-	0.833923	0.91129	0.70998	-2878.187277	-2879.709853	-2878.999873	**-70.7**	**-70.7**
**Hydroamination. IVb to 8b**										
**TS$_{IVb-VIIIb}$**	-2803.610867	-65.3	0.827163	0.902820	0.7065510	-2802.904316	-2804.388398	-2803.681847	**4.7**	**2.3**

**VIIIb**	-2803.62992	-	0.831178	0.906583	0.714166	-2802.915754	-2804.406425	-2803.692259	**-1.9**	-4.2
**VIIIb-HCO_3^-**	-3068.200547	-	0.857855	0.940062	0.732072	-3067.468475	-3069.104221	-3068.372149	**-8.5**	-13.3
**TS$_{VIIIb-XIb}$**	-3068.195831	-457.0	0.853539	0.935214	0.729477	-3067.466354	-3069.096493	-3068.367016	**-5.3**	-10.0
**XIb-H_2CO_3**	-3068.196086	-	0.855818	0.938173	0.730093	-3067.465993	-3069.097094	-3068.367001	**-5.3**	-10.0
**TS$_{VIIIb-8b}$**	-3068.168532	-1311.1	0.850644	0.933268	0.721141	-3067.447391	-3069.072661	-3068.35152	**4.4**	-0.3
**XIIb-HCO_3^-**	-3068.215165	-	0.858066	0.940758	0.730043	-3067.485122	-3069.122273	-3068.39223	**-21.1**	-25.9
**XIIb**	-2803.662479	-	0.829717	0.905403	0.712564	-2802.949915	-2804.439106	-2803.726542	**-23.4**	-25.8
**TS-$_{VIIIb-XII'b}$**	-2803.549119	-1570.9	0.82301	0.898759	0.706232	-2802.842887	-2804.32522	-2803.618988	**44.1**	41.7
**XII'b**	-2803.663393	-	0.828679	0.904599	0.710355	-2802.953038	-2804.440291	-2803.729936	**-25.5**	-27.9
**8b**	-442.463308	-	0.186626	0.201634	0.141183	-442.322125	-442.62008	-442.478897	**-31.6**	-34.4

13. DLPNO-CCSTD Energy values

**Common species**								
**Structure**	**BS1**				**DLPNO-CCSTD**		**kcal/mol) corr with BS2**	**cal/mol) correc atm to 1 M**
	**E (a.u.)**	**E gas (a.u.)**	**ΔE solv (a.u.)**	**G_therm (a.u.)**	**E (a.u.)**	**G (a.u.) correctec**		
**I**	-2361.167324	-2361.073751	-0.093573	0.534654	-2356.68584	-2356.24476	**0.0**	0.0

**4-pic *N*-oxide**	-362.791043	-362.7772	-0.013856	0.078155	-362.124677	-362.06038	-	-
**4-picoline**	-287.625740	-287.615607	-0.010133	0.075776	-287.05890	-286.99326	-	-
**HCO_3^-**	-264.513904	-264.4261	-0.087789	-0.007239	-264.09850	-264.1935	-	-
**H_2O**	-76.425073	-76.41815	-0.006923	-0.002040	-76.32676	-76.33572	-	-

**ε-lactam (2a)**								
**Structure**	**BS1**				**DLPNO-CCSTD**		**kcal/mol) corr with BS2**	**kcal/mol) correct atm to 1 M**
	**E (a.u.)**	**E gas (a.u.)**	**ΔE solv (a.u.)**	**G_therm (a.u.)**	**E (a.u.)**	**G (a.u.) corrected**		
**1a**	-560.3571	-560.34017	-0.016927	0.210582	-559.2253	-559.03164	-	-
**IIa**	-2921.573051	-2921.4801	-0.092982	0.786070	-2915.96955	-2915.27646	**0.0**	**-2.4**
**TS$_{IIa-IIIa}$**	-2921.559920	-2921.4671	-0.092793	0.780173	-2915.949436	-2915.26206	**9.0**	**6.6**
**IIIa**	-2921.562372	-2921.4677	-0.09467	0.780358	-2915.95365	26796	**5.3**	**2.9**
**TS$_{IIIa-IVa}$**	-2921.547830	-2921.4540	-0.093858	0.777732	-2915.93844	-2915.2546	**13.7**	**11.3**

**IVa**	-2921.590432	-2921.4949	-0.09549	0.781192	-2915.979639	-2915.2939	**-11.0**	-13.4
**TS$_{IVa-Va}$**	-3284.387123	-3284.289	-0.098576	0.893544	-3278.110155	-3277.3152	**13.5**	8.8
**Va**	-3284.395270	-3284.292	-0.103161	0.894632	-3278.121451	-3277.3300	**4.3**	-0.5
**TS$_{Va_VIa}$**	-3284.392808	-3284.2982	-0.094608	0.892492	-3278.117265	-3277.31938	**10.9**	6.2
**VIa**	-2996.813292	-2996.7248	-0.088492	0.781944	-2991.121006	-2990.42755	**-52.7**	-57.5
**VIIa**	-635.618510	-635.60099	-0.017515	0.213488	-634.3882219	-634.19225	**-58.7**	-58.7
**Metal-free Lactamization. VIa to 2a**								
**VIIa-H_2O**	-712.079481	-712.052258	-0.027223	0.243815	-710.737803	-710.792249	**-224.5**	-226.9
**TS$_{VIIaIXa}$-H_2O**	-712.063466	-712.040643	-0.022823	0.239218	-710.7249207	-710.770567	**-210.9**	-213.3
**IX'a-H_2O**	-712.087069	-712.06595	-0.021119	0.241884	-710.754431	-710.796669	**-227.3**	-229.6
**IXa-H_2O**	-712.091572	-712.067855	-0.023717	0.238940	-710.755061	-710.802495	**-230.9**	-233.3
**TS$_{IXa_2a}$-H_2O**	-712.062939	-712.042156	-0.020783	0.237047	-710.721487	-710.763053	**-206.2**	-208.5
**2a**	-635.68	-635.661346	-0.018654	0.221410	-634.449862	-634.487170	**-243.7**	-243.7
**Hydroamination. IVa to 8a**								
**TS$_{IVa-VIIIa}$**	-2921.567683	-2921.478	-0.089663	0.792129	-2915.956771	-2915.25431	**13.9**	13.9
**VIIIa**	-2921.585292	-2921.493	-0.092738	0.801366	-2915.981712	-2915.27308	**2.1**	2.1
**TS$_{VIIIa-8a}$**	-3186.120447	-3186.067	-0.053317	0.80666	-3180.195169	-3179.44183	**17.6**	15.3

**8a**	-560.423409	-560.406	-0.017435	0.218645	-559.2829997	-559.08179	**-31.5**	**-33.8**

δ-lactam (2b)								
**Structure**	**BS1**				**DLPNO-CCSTD**		**kcal/mol) corr with BS2**	**kcal/mol) correct atm to 1 M**
	**E (a.u.)**	**E gas (a.u.)**	**ΔE solv (a.u.)**	**G_therm (a.u.**	**E (a.u.)**	**G (a.u.) corrected**		
**1b**	-442.397283	-442.383194	-0.014089	0.134812	-441.5205826	-441.400	-	-
**IIb**	-2803.603772	-2803.513356	-0.090416	0.706656	-2798.2574314	-2797.64119	**2.1**	**-0.2**
**TS$_{IIb-IIIb}$**	-2803.592678	-2803.503758	-0.08892	0.700320	-2798.241513	-2797.630113	**9.1**	**6.7**
**IIIb**	-2803.594816	-2803.50479	-0.090026	0.704936	-2798.24730	-2797.632391	**7.7**	**5.3**
**TS$_{IIIb-IVb}$**	-2803.582504	-2803.492978	-0.089526	0.703992	-2798.23437	-2797.619904	**15.5**	**13.1**
**IVb**	-2803.627176	-2803.534405	-0.092771	0.706942	-2798.273827	-2797.660	**-9.4**	**-12.7**
**TS$_{IVb-Vb}$**	-3166.425221	-3166.425221	-3166.328	0.816021	-3160.404269	-3159.686	**12.1**	**7.3**
**Vb**	-3166.437300	-3166.4373	-3166.338	0.820388	-3160.421206	-3159.700	**2.9**	**-1.8**
**TS$_{Vb-VIb}$**	-3166.429160	-3166.3340	-0.094994	0.813778	-3160.4074813	-3159.688697	**10.2**	**5.5**
**VIb**	-2878.856944	-2878.7580	-0.099268	0.713005	-2873.406093	-2872.792	**-50.6**	**-53.0**

**VIIb**	-517.658964	-517.64460	-0.014368	0.137190	-516.6842997	-516.561	**-59.3**	**-61.7**
**Metal-free Lactamization. VIb to 2b**								
**VIIb-H_2O**	-594.125531	-594.100957	-0.024574	0.164191	-593.040243	-592.90063	**-61.4**	**-63.8**
**$TS_{VIIb\text{-}IXb}$-H_2O**	-594.114697	-594.094234	-0.020463	0.161872	-593.0313162	-592.88991	**-54.7**	**-57.1**
**IX'b-H_2O**	-594.133514	-594.115438	-0.018076	0.164892	-593.056673	-592.90986	**-67.2**	**-69.6**
**IXb-H_2O**	-594.134818	-594.111963	-0.022855	0.161843	-593.053015	-592.91403	**-69.9**	**-72.2**
**$TS_{IXb\text{-}2b}$-H_2O**	-594.105566	-594.087536	-0.01803	0.158689	-593.020025	-592.87937	**-48.1**	**-50.5**
**2b**	-517.724680	-517.707293	-0.017387	0.143143	-516.748106	-516.62235	**-97.5**	**-97.5**
**Hydroamination. IVb to 8b**								
**$TS_{IVb\text{-}VIIIb}$**	-2803.610867	-2803.521	-0.089639	0.7065510	-2798.254669	-2797.638	**4.3**	**1.9**
**VIIIb**	-2803.62992	-2803.535	-0.095112	0.714166	-2798.277374	-2797.658	**-8.6**	**-11.0**
**$TS_{VIIIb\text{-}8b}$**	-3068.168532	-3068.11	-0.058975	0.721141	-3062.493615	-3061.831	**4.2**	**-0.5**
**8b**	-442.463308	-442.4486	-0.014668	0.141183	-441.5800629	-441.454	**-33.7**	**-36.1**

imide (9a)								
Structure	BS1				DLPNO-CCSTD		kcal/mol) corr with BS2	kcal/mol) correct atm to 1 M
	E (a.u.)	E gas (a.u.)	\E solv (a.u.	G_therm (a.u.)	E (a.u.)	G (a.u.) corrected		
**9a**	-555.764708	-555.7474	-0.01735	0.139548	-554.7197959	-554.59759	-	-
**XVIa**	-2916.996088	-2916.905	-0.09144	0.708396	-2911.477971	-2910.86102	**-11.7**	**-14.1**
**TS$_{XVIa-XVIIa}$**	-3279.796911	-3279.704	-0.09318	0.82142	-3273.610011	-3272.88177	**13.2**	**8.4**
**XVIIa**	-3279.805654	-3279.708	-0.09734	0.824265	-3273.625029	-3272.89811	**2.9**	**-1.8**
**Hydroamination. IVa to 11a**								
**TS$_{XVIa-XVIIIa}$**	-2916.959217	-2916.867	-0.09264	0.718712	-2911.438336	-2910.81227	**18.9**	**18.9**
**XIIIa**	-2916.962773	-2916.868	-0.09477	0.722086	-2911.44614	-2910.81882	**14.8**	**14.8**
**11a**	-555.819449	-555.8024	-0.01706	0.19962	-554.7698737	-554.58731	**6.5**	**6.5**

imide (9a)								
Structure	BS1				DLPNO-CCSTD		kcal/mol) corre with BS2	kcal/mol) correct atm to 1 M
	E (a.u.)	E gas (a.u.)	\E solv (a.u.	G_therm (a.u.)	E (a.u.)	G (a.u.) corrected		
**9a**	-555.764708	-555.7474	-0.01735	0.139548	-554.7197959	-554.59759	-	-
**XVIa**	-2916.996088	-2916.905	-0.09144	0.708396	-2911.477971	-2910.86102	**-11.7**	**-14.1**
**TS$_{XVIa-XVIIa}$**	-3279.796911	-3279.704	-0.09318	0.82142	-3273.610011	-3272.88177	**13.2**	**8.4**
**XVIIa**	-3279.805654	-3279.708	-0.09734	0.824265	-3273.625029	-3272.89811	**2.9**	**-1.8**
**Hydroamination. IVa to 11a**								
**TS$_{XVIa-XVIIIa}$**	-2916.959217	-2916.867	-0.09264	0.718712	-2911.438336	-2910.81227	**18.9**	**18.9**
**XIIIa**	-2916.962773	-2916.868	-0.09477	0.722086	-2911.44614	-2910.81882	**14.8**	**14.8**
**11a**	-555.819449	-555.8024	-0.01706	0.19962	-554.7698737	-554.58731	**6.5**	**6.5**

14. Cartesian coordinates in Å for all the stationary points involved throughout DFT study

a. Coordinates for common species

$CpRu(PPh_3)_2Cl$

C	-0.701878	-3.581468	-0.501880
C	-0.924954	-3.045248	0.797789
C	0.335642	-2.694748	1.380189
C	1.356478	-3.063184	0.431902
C	0.721064	-3.585200	-0.722912
H	-1.456104	-3.937697	-1.188975
H	-1.892137	-2.918719	1.263660
H	0.495807	-2.298550	2.372656
H	2.422164	-2.940161	0.571761
H	1.216280	-3.911151	-1.627817
Ru	0.050376	-1.458392	-0.436052
Cl	0.089512	-1.118656	-2.921811
P	1.785182	0.090155	-0.135739
P	-1.768761	-0.027769	0.022797
C	3.195636	-0.058354	-1.328202
C	3.408986	-1.266391	-2.006816
C	4.103630	0.996341	-1.518061
C	4.510485	-1.421816	-2.851317
H	2.701833	-2.077033	-1.886727
C	5.201543	0.842235	-2.366350
H	3.959400	1.939520	-1.000516
C	5.408680	-0.368034	-3.033875
H	4.659603	-2.363135	-3.373174
H	5.894789	1.667417	-2.504153
H	6.262364	-0.486553	-3.695392
C	2.637259	-0.141250	1.493321
C	3.978852	-0.535426	1.599822
C	1.890507	0.038048	2.670338
C	4.557808	-0.747194	2.855488
H	4.579588	-0.680706	0.708734
C	2.473325	-0.157858	3.921656
H	0.850990	0.331402	2.607739
C	3.810366	-0.556236	4.018469
H	5.597106	-1.057462	2.919555
H	1.878960	-0.003145	4.817783
H	4.264169	-0.716316	4.992469
C	1.465902	1.906337	-0.196786
C	1.874229	2.814766	0.790096
C	0.765580	2.382445	-1.315525
C	1.557737	4.170784	0.672107
H	2.426559	2.471552	1.657695
C	0.447079	3.735175	-1.430493
H	0.460598	1.685387	-2.088401
C	0.835745	4.632404	-0.431229
H	1.872037	4.864024	1.447461
H	-0.117654	4.081736	-2.290947
H	0.580827	5.685201	-0.514479
C	-1.609840	0.681187	1.723164
C	-1.151383	1.993014	1.924095
C	-1.787978	-0.147651	2.845899
C	-0.889160	2.465988	3.212266
H	-0.985059	2.649589	1.078684
C	-1.530907	0.328565	4.132007
H	-2.130328	-1.169348	2.721092

C	-1.079257	1.638644	4.320290
H	-0.527165	3.481768	3.342134
H	-1.680538	-0.325905	4.986061
H	-0.874982	2.007779	5.321186
C	-3.381497	-0.954276	0.060156
C	-3.624212	-1.872724	-0.974125
C	-4.395511	-0.705401	0.996584
C	-4.845292	-2.543431	-1.057581
H	-2.852587	-2.061028	-1.714097
C	-5.613499	-1.386111	0.918119
H	-4.243470	0.017477	1.790887
C	-5.841196	-2.308345	-0.105573
H	-5.015925	-3.250976	-1.864327
H	-6.385578	-1.189836	1.657061
H	-6.789066	-2.835786	-0.164575
C	-2.393213	1.402170	-0.991383
C	-2.160840	1.417485	-2.373779
C	-3.217870	2.394435	-0.433725
C	-2.704778	2.425253	-3.174486
H	-1.542373	0.646622	-2.819143
C	-3.752582	3.404915	-1.233626
H	-3.447749	2.386461	0.626272
C	-3.492946	3.427935	-2.606869
H	-2.505709	2.425046	-4.242769
H	-4.377722	4.170872	-0.782895
H	-3.909912	4.215608	-3.228303

I

C	-0.942544	-2.625141	-2.517370
C	-1.127647	-2.935288	-1.143457
C	0.167605	-3.020102	-0.519658
C	1.160804	-2.841184	-1.555453
C	0.479211	-2.566042	-2.761650
H	-1.720521	-2.487767	-3.255538
H	-2.078211	-3.058057	-0.642075
H	0.363794	-3.273703	0.512612
H	2.232746	-2.870888	-1.415969
H	0.943963	-2.333192	-3.711828
Ru	-0.082003	-1.015524	-1.225900
P	1.758467	0.057092	-0.126235
P	-1.825130	0.046982	-0.049979
C	3.036833	0.612721	-1.335096
C	2.893585	0.304532	-2.695835
C	4.150880	1.360313	-0.915048
C	3.847084	0.733289	-3.623533
H	2.034382	-0.272529	-3.026419
C	5.105171	1.779510	-1.841103
H	4.270822	1.614654	0.133816
C	4.953932	1.468872	-3.196894
H	3.722180	0.493642	-4.675683
H	5.964865	2.352782	-1.506248
H	5.695697	1.803444	-3.916368
C	2.603182	-1.163214	0.963046
C	3.888127	-1.654508	0.697028
C	1.890631	-1.665240	2.066633
C	4.451564	-2.631696	1.523717
H	4.453665	-1.283378	-0.151120
C	2.461718	-2.629574	2.895891
H	0.891676	-1.300237	2.279897
C	3.743308	-3.119115	2.623374
H	5.447186	-3.007508	1.305768
H	1.902306	-2.999964	3.750241
H	4.185805	-3.875585	3.265039

C	1.614378	1.567042	0.930590
C	2.001964	1.620501	2.276437
C	1.095584	2.724581	0.328739
C	1.839354	2.798236	3.011260
H	2.423252	0.749887	2.764667
C	0.925401	3.896426	1.063920
H	0.819993	2.714319	-0.718916
C	1.289116	3.933227	2.413737
H	2.140301	2.821952	4.054742
H	0.506014	4.775215	0.583051
H	1.153409	4.843327	2.990940
C	-1.634618	-0.168464	1.762731
C	-1.183020	0.848372	2.613793
C	-1.816702	-1.465682	2.280001
C	-0.929304	0.573861	3.961152
H	-1.007016	1.848046	2.234338
C	-1.566754	-1.732827	3.624484
H	-2.153062	-2.266556	1.628234
C	-1.119599	-0.711064	4.469731
H	-0.571186	1.370252	4.606472
H	-1.714800	-2.737225	4.010546
H	-0.918797	-0.919042	5.516673
C	-3.526369	-0.586535	-0.408299
C	-3.862257	-0.857885	-1.744587
C	-4.511713	-0.717440	0.581423
C	-5.152753	-1.265630	-2.083350
H	-3.111799	-0.750713	-2.521706
C	-5.801512	-1.133005	0.240510
H	-4.280880	-0.500656	1.618798
C	-6.124689	-1.410063	-1.089333
H	-5.396951	-1.473576	-3.121256
H	-6.553477	-1.236712	1.017648
H	-7.128061	-1.733727	-1.350700
C	-2.066304	1.831435	-0.409018
C	-1.532210	2.319247	-1.611083
C	-2.831373	2.683602	0.400772
C	-1.735497	3.647778	-1.988625
H	-0.940443	1.661474	-2.245618
C	-3.021104	4.015431	0.029983
H	-3.275137	2.312171	1.319154
C	-2.472500	4.499642	-1.161861
H	-1.312377	4.017741	-2.917980
H	-3.602539	4.673990	0.668693
H	-2.624524	5.536532	-1.447284

4-Pic *N*-oxide

C	-0.773850	1.175830	-0.002061
C	0.609460	1.189563	-0.010181
C	1.350551	0.000046	-0.012271
C	0.609490	-1.189515	-0.010173
C	-0.773800	-1.175830	-0.002057
H	-1.393025	2.063203	-0.002465
H	1.110266	2.152973	-0.016445
H	1.110337	-2.152906	-0.016455
H	-1.392953	-2.063218	-0.002504
C	2.855559	-0.000009	0.012240
H	3.261878	0.888720	-0.479973
H	3.228927	-0.002110	1.044433
H	3.261845	-0.886816	-0.483484
O	-2.764872	-0.000042	0.009865
N	-1.476107	-0.000004	0.003571

4-Picoline

C	-1.221340	1.141507	0.002483
C	0.171944	1.193065	-0.008784
C	0.906158	-0.000000	-0.012404
C	0.171943	-1.193065	-0.008785
C	-1.221340	-1.141506	0.002483
H	-1.798629	2.064799	0.003905
H	0.679163	2.153833	-0.016873
H	0.679163	-2.153833	-0.016875
H	-1.798630	-2.064798	0.003909
C	2.411816	-0.000000	0.007361
H	2.818129	0.889029	-0.484130
H	2.779161	0.000019	1.041355
H	2.818128	-0.889048	-0.484096
N	-1.927367	0.000000	0.008382

HCO_3^-

C	0.146771	-0.063807	0.000012
O	1.207099	0.589611	-0.000031
O	-1.031724	0.746098	0.000062
H	-1.751473	0.097777	-0.000458
O	-0.066518	-1.300076	0.000017

Cl^-

Cl	0.000000	0.000000	0.000000

H_2CO_3

C	-0.000000	0.100975	-0.000012
O	0.000072	1.314467	0.000002
O	1.085529	-0.682731	0.000000
H	1.864143	-0.099409	0.000024
O	-1.085594	-0.682632	0.000001
H	-1.864194	-0.099270	0.000022

H_2O

O	0.000000	0.000000	0.119956
H	0.000000	-0.759138	-0.479824
H	0.000000	0.759138	-0.479824

b. Coordinates for aminoketene VIIa formation. 1a to VIIa.

1a

C	-3.792068	-0.440511	-0.149775
C	-2.659452	0.391052	-0.074804
C	-1.364199	-0.169987	0.080689
C	-1.261685	-1.563399	0.157229
C	-2.390442	-2.384839	0.081823
C	-3.659267	-1.824811	-0.072391
H	-4.771652	0.012451	-0.268752
H	-0.288349	-2.026559	0.277344
H	-2.273688	-3.463133	0.144375
H	-4.538871	-2.458914	-0.131271
C	-2.827410	1.809831	-0.154793
C	-2.964318	3.012200	-0.220886
H	-3.091537	4.071602	-0.278651
C	-0.163986	0.749732	0.167747
C	1.204593	0.071113	0.242720
H	-0.276994	1.407063	1.039723
H	-0.176791	1.422234	-0.701458
H	1.261223	-0.561910	1.137791

H	1.327407	-0.599774	-0.624416
N	2.253617	1.087157	0.350542
C	3.605549	0.556057	0.547041
C	4.186227	-0.298136	-0.592945
H	3.604468	-0.032508	1.475251
H	4.274202	1.409800	0.722194
C	5.624441	-0.739798	-0.308167
H	3.556923	-1.181925	-0.754085
H	4.151240	0.285289	-1.523776
H	6.030077	-1.342496	-1.128048
H	6.285122	0.125012	-0.171619
H	5.678276	-1.343272	0.606083
H	2.245727	1.655924	-0.495399

IIa

C	-0.066420	2.365854	-2.468674
C	0.161852	1.064584	-2.988929
C	1.542610	0.747853	-2.822548
C	2.181657	1.903875	-2.243530
C	1.196072	2.891086	-2.017747
H	-1.015470	2.880992	-2.445396
H	-0.591034	0.422478	-3.422810
H	2.031406	-0.159492	-3.143185
H	3.235301	1.995705	-2.019640
H	1.355838	3.862540	-1.570527
Ru	0.675377	1.043867	-0.775242
P	2.604287	0.187165	0.383163
P	-0.701337	-0.872880	-0.488366
C	3.571299	1.498279	1.270594
C	3.438811	2.851001	0.924344
C	4.520019	1.141329	2.245005
C	4.227973	3.825360	1.539837
H	2.710947	3.151224	0.181852
C	5.307088	2.115579	2.859635
H	4.653128	0.101345	2.522923
C	5.162699	3.460823	2.510175
H	4.105973	4.868380	1.261962
H	6.033883	1.821158	3.611102
H	5.773993	4.218546	2.991792
C	3.872784	-0.484105	-0.789534
C	5.136366	0.108557	-0.938209
C	3.549847	-1.592492	-1.589719
C	6.052724	-0.402151	-1.862194
H	5.415732	0.970180	-0.343400
C	4.471192	-2.109170	-2.500062
H	2.574993	-2.051458	-1.506631
C	5.726930	-1.512802	-2.641906
H	7.024167	0.072480	-1.966971
H	4.199455	-2.972981	-3.099619
H	6.443493	-1.909093	-3.355517
C	2.496686	-1.107833	1.702061
C	2.985749	-2.411718	1.536861
C	1.945474	-0.748901	2.944243
C	2.896232	-3.340719	2.576692
H	3.441845	-2.717147	0.604404
C	1.862062	-1.675318	3.981992
H	1.596619	0.262374	3.117146
C	2.329317	-2.979167	3.798909
H	3.277732	-4.346567	2.426341
H	1.430608	-1.377093	4.932719
H	2.261572	-3.702181	4.606603
C	0.085625	-2.401412	-1.169045

C	0.591812	-3.407676	-0.334758
C	0.251894	-2.528763	-2.560247
C	1.262827	-4.507583	-0.877074
H	0.475153	-3.338474	0.739312
C	0.914299	-3.630402	-3.099817
H	-0.147904	-1.773626	-3.227468
C	1.428858	-4.621984	-2.257535
H	1.656586	-5.271629	-0.213111
H	1.030066	-3.713435	-4.176539
H	1.950111	-5.477468	-2.677005
C	-2.267846	-0.788383	-1.488800
C	-2.887795	0.452333	-1.687764
C	-2.872085	-1.935484	-2.032884
C	-4.079699	0.552705	-2.408335
H	-2.437068	1.347999	-1.285281
C	-4.061226	-1.835371	-2.757134
H	-2.413500	-2.910285	-1.910367
C	-4.669525	-0.591766	-2.948275
H	-4.541940	1.526445	-2.542283
H	-4.511115	-2.732347	-3.172910
H	-5.594138	-0.517474	-3.513160
C	-1.352882	-1.452506	1.146775
C	-2.118124	-2.631645	1.232548
C	-1.129898	-0.711849	2.312341
C	-2.636133	-3.054326	2.455854
H	-2.308352	-3.227934	0.347621
C	-1.654913	-1.131967	3.537807
H	-0.563026	0.206121	2.262437
C	-2.405594	-2.303571	3.614222
H	-3.223616	-3.966506	2.502751
H	-1.475734	-0.537304	4.428515
H	-2.816331	-2.629507	4.565192
C	-1.575809	4.533828	-0.463578
C	-1.809444	3.332084	0.238897
C	-3.126305	3.002108	0.652125
C	-4.164347	3.877367	0.309338
C	-3.926691	5.052699	-0.404924
C	-2.625178	5.388876	-0.784315
H	-0.561467	4.786886	-0.753415
H	-5.179421	3.641581	0.611747
H	-4.755943	5.707610	-0.656350
H	-2.427728	6.309101	-1.325864
C	-0.672544	2.500379	0.555853
C	0.363506	2.085452	1.123986
H	0.971927	2.159656	2.008641
C	-3.374580	1.756735	1.466930
C	-4.750555	1.106503	1.302043
H	-3.205657	1.974841	2.530641
H	-2.619803	1.015630	1.198233
H	-5.542458	1.763811	1.683441
H	-4.943497	0.969714	0.226271
N	-4.785937	-0.138866	2.071670
C	-5.952731	-0.987829	1.813983
C	-6.002068	-1.647290	0.425834
H	-6.858197	-0.385834	1.971839
H	-5.967165	-1.776908	2.578658
C	-7.204118	-2.578805	0.256625
H	-6.020712	-0.877963	-0.354143
H	-5.070543	-2.209733	0.276026
H	-7.211080	-3.044773	-0.735021
H	-7.192419	-3.382799	1.002725
H	-8.148633	-2.033847	0.374392

H	-3.952370	-0.678850	1.850503

$TS_{IIa-IIIa}$

C	-0.197393	1.722576	-2.906030
C	0.253670	0.420285	-3.227667
C	1.669421	0.358266	-3.008448
C	2.098438	1.673631	-2.604717
C	0.953761	2.503394	-2.522286
H	-1.218189	2.074999	-2.946085
H	-0.373378	-0.398137	-3.552178
H	2.307758	-0.494651	-3.189983
H	3.116633	1.968113	-2.389807
H	0.939342	3.543552	-2.225371
Ru	0.731837	0.807665	-1.053841
P	2.603629	0.568717	0.382889
P	-0.416617	-1.170800	-0.460772
C	3.207020	2.166496	1.105000
C	2.780904	3.393549	0.576101
C	4.133615	2.170710	2.163635
C	3.261507	4.598899	1.095988
H	2.070986	3.411536	-0.242097
C	4.614117	3.373798	2.679396
H	4.483498	1.233864	2.584918
C	4.176140	4.591702	2.149563
H	2.916111	5.540070	0.677798
H	5.329344	3.360120	3.496882
H	4.547735	5.527697	2.556475
C	4.067056	-0.045634	-0.567328
C	5.241716	0.705726	-0.717239
C	3.971879	-1.292912	-1.208489
C	6.297259	0.217293	-1.493661
H	5.342133	1.673097	-0.237802
C	5.032847	-1.784645	-1.968136
H	3.067650	-1.880643	-1.115617
C	6.198411	-1.027460	-2.117569
H	7.198454	0.813422	-1.605910
H	4.941471	-2.755325	-2.446962
H	7.021891	-1.404745	-2.717108
C	2.591865	-0.489466	1.899811
C	3.309138	-1.687823	2.018537
C	1.828165	-0.047549	2.992543
C	3.239500	-2.440262	3.194335
H	3.924713	-2.045475	1.202493
C	1.762659	-0.796436	4.165973
H	1.292547	0.894824	2.931999
C	2.462302	-2.002118	4.267354
H	3.797680	-3.369324	3.266837
H	1.162495	-0.440803	4.998014
H	2.408395	-2.588975	5.179713
C	0.605442	-2.667826	-0.812796
C	1.225079	-3.393780	0.214890
C	0.887078	-3.009872	-2.148477
C	2.110877	-4.432353	-0.085632
H	1.033722	-3.148283	1.252219
C	1.763935	-4.052549	-2.445180
H	0.418291	-2.466684	-2.961558
C	2.384237	-4.764612	-1.413015
H	2.588426	-4.976224	0.724152
H	1.965134	-4.306245	-3.482006
H	3.072543	-5.572185	-1.644531
C	-1.934415	-1.428168	-1.496717
C	-2.729699	-0.315376	-1.812188

C	-2.340139	-2.696856	-1.939199
C	-3.892368	-0.462405	-2.570372
H	-2.436628	0.669650	-1.469590
C	-3.501540	-2.842204	-2.701985
H	-1.750500	-3.575452	-1.701582
C	-4.278047	-1.726421	-3.024043
H	-4.495152	0.411238	-2.801291
H	-3.797734	-3.829978	-3.043391
H	-5.179545	-1.842615	-3.618370
C	-1.114493	-1.504870	1.220990
C	-1.638359	-2.773335	1.533943
C	-1.181650	-0.493243	2.184389
C	-2.187543	-3.023358	2.790820
H	-1.613519	-3.571458	0.798915
C	-1.732661	-0.742567	3.445325
H	-0.806779	0.493838	1.956618
C	-2.230053	-2.009128	3.754142
H	-2.584261	-4.008918	3.017196
H	-1.774517	0.056364	4.179673
H	-2.659556	-2.203968	4.732295
C	-2.889217	4.438368	-1.093420
C	-2.872114	3.393438	-0.133851
C	-4.059923	3.054218	0.574197
C	-5.222672	3.775479	0.282038
C	-5.232703	4.792163	-0.673393
C	-4.062490	5.124980	-1.367663
H	-1.968539	4.688703	-1.611193
H	-6.136031	3.534223	0.819056
H	-6.154019	5.331293	-0.874585
H	-4.069754	5.919091	-2.107805
C	-1.652126	2.722852	0.096446
C	-0.565968	2.128424	0.176811
H	0.291523	2.134618	0.844724
C	-4.107022	1.944355	1.595287
C	-4.874538	0.712489	1.080627
H	-4.597839	2.303586	2.507209
H	-3.093641	1.638509	1.871671
H	-5.911307	0.994345	0.853825
H	-4.422410	0.389512	0.130818
N	-4.892342	-0.329587	2.105789
C	-5.688144	-1.513915	1.767871
C	-5.291748	-2.260467	0.483164
H	-6.743862	-1.212911	1.703022
H	-5.612873	-2.204835	2.618530
C	-6.033199	-3.589334	0.324154
H	-5.483265	-1.627979	-0.390420
H	-4.209596	-2.441106	0.500604
H	-5.746865	-4.095443	-0.604576
H	-5.811147	-4.268995	1.156321
H	-7.119931	-3.440875	0.300814
H	-3.934156	-0.629808	2.270785

IIIa

C	-0.179043	1.371854	-3.145394
C	0.363544	0.075737	-3.303959
C	1.760408	0.128141	-2.991008
C	2.087350	1.505266	-2.713642
C	0.895807	2.263672	-2.788222
H	-1.216289	1.649193	-3.269159
H	-0.192066	-0.810422	-3.575331
H	2.457227	-0.694814	-3.049978
H	3.071221	1.886393	-2.477636

H	0.799425	3.325288	-2.604091
Ru	0.683925	0.706142	-1.138333
P	2.513985	0.727043	0.383522
P	-0.296615	-1.323290	-0.420177
C	2.873609	2.422029	1.033339
C	2.362491	3.557750	0.387518
C	3.693344	2.592532	2.163130
C	2.658829	4.837843	0.862873
H	1.729108	3.446104	-0.484107
C	3.988261	3.871489	2.634634
H	4.103660	1.728111	2.675065
C	3.469743	4.997195	1.987547
H	2.250767	5.706962	0.354932
H	4.621567	3.988080	3.509294
H	3.696265	5.991940	2.360259
C	4.083172	0.235002	-0.456038
C	5.175701	1.106689	-0.570284
C	4.169493	-1.046135	-1.027110
C	6.331345	0.702015	-1.245089
H	5.134140	2.101181	-0.140188
C	5.329395	-1.451106	-1.686508
H	3.332970	-1.729103	-0.953029
C	6.412975	-0.575308	-1.801827
H	7.168065	1.389424	-1.330304
H	5.380907	-2.449135	-2.111827
H	7.313747	-0.887096	-2.322520
C	2.517364	-0.254706	1.942702
C	3.461407	-1.251015	2.229143
C	1.535118	0.048885	2.897825
C	3.398912	-1.952292	3.435946
H	4.242600	-1.491906	1.518394
C	1.473475	-0.652380	4.100604
H	0.820079	0.841347	2.703778
C	2.401276	-1.662785	4.368740
H	4.132253	-2.726542	3.642278
H	0.696555	-0.415917	4.820776
H	2.351114	-2.214426	5.303015
C	0.885857	-2.719900	-0.646304
C	1.612190	-3.248191	0.431568
C	1.177816	-3.171633	-1.945987
C	2.605601	-4.206309	0.215297
H	1.415665	-2.912980	1.441699
C	2.169859	-4.128687	-2.158599
H	0.625827	-2.784702	-2.795705
C	2.889727	-4.647833	-1.077717
H	3.159992	-4.598484	1.062787
H	2.378977	-4.468729	-3.168721
H	3.664029	-5.390965	-1.244123
C	-1.756583	-1.791092	-1.462386
C	-2.605772	-0.782229	-1.942862
C	-2.073987	-3.132002	-1.730118
C	-3.736117	-1.105699	-2.696560
H	-2.385962	0.256003	-1.726154
C	-3.199271	-3.451964	-2.492583
H	-1.442789	-3.931151	-1.356377
C	-4.031226	-2.441037	-2.980881
H	-4.385028	-0.312298	-3.056201
H	-3.425955	-4.493392	-2.701406
H	-4.906944	-2.693770	-3.571425
C	-1.014752	-1.589403	1.263476
C	-1.230886	-2.889165	1.755041
C	-1.436497	-0.499785	2.034261

C	-1.808391	-3.085491	3.009599
H	-0.947858	-3.753587	1.164442
C	-2.023077	-0.697275	3.287112
H	-1.310682	0.508785	1.665447
C	-2.201158	-1.989664	3.783408
H	-1.956119	-4.096135	3.378683
H	-2.333573	0.162746	3.873395
H	-2.649875	-2.143348	4.760349
C	-3.082607	4.383752	-1.257252
C	-3.079683	3.333342	-0.308465
C	-4.237845	3.085663	0.473415
C	-5.360784	3.895022	0.261783
C	-5.359488	4.921999	-0.682412
C	-4.213290	5.168629	-1.445686
H	-2.183463	4.566039	-1.838447
H	-6.252895	3.712483	0.855371
H	-6.247973	5.531668	-0.819146
H	-4.202191	5.970009	-2.178406
C	-1.898807	2.551560	-0.187843
C	-0.866476	1.882684	-0.235846
H	0.131292	1.781127	0.302764
C	-4.310501	1.967574	1.484989
C	-4.863803	0.661926	0.882994
H	-4.971194	2.264923	2.306434
H	-3.322607	1.773362	1.916388
H	-5.791238	0.883309	0.338618
H	-4.145496	0.281733	0.138388
N	-5.183466	-0.289259	1.947878
C	-5.793458	-1.542518	1.491154
C	-4.885365	-2.488340	0.690097
H	-6.679247	-1.287835	0.892472
H	-6.162830	-2.075964	2.378253
C	-5.604289	-3.771888	0.269359
H	-4.506160	-1.975508	-0.198632
H	-4.010365	-2.735970	1.303270
H	-4.930223	-4.446287	-0.268838
H	-5.996364	-4.313466	1.139274
H	-6.451044	-3.553117	-0.392804
H	-4.325498	-0.515391	2.448232

$TS_{IIIa\text{-}IVa}$

C	-0.030836	0.639185	-3.453286
C	0.553172	-0.652572	-3.363036
C	1.936779	-0.510952	-3.028865
C	2.206792	0.893556	-2.931667
C	1.001942	1.603096	-3.190394
H	-1.061782	0.857092	-3.694933
H	0.027864	-1.584135	-3.518679
H	2.652913	-1.311362	-2.916182
H	3.165747	1.337075	-2.699083
H	0.883098	2.677888	-3.205550
Ru	0.703594	0.346014	-1.318730
P	2.321072	0.804050	0.322388
P	-0.271603	-1.552512	-0.337791
C	2.597074	2.614871	0.620100
C	2.127421	3.562419	-0.300998
C	3.310768	3.064968	1.745805
C	2.354744	4.927033	-0.101638
H	1.579487	3.233869	-1.174971
C	3.539269	4.426740	1.942996
H	3.692342	2.352597	2.469767
C	3.058809	5.362393	1.021701

H	1.978111	5.645666	-0.824100
H	4.092252	4.756671	2.817792
H	3.233726	6.422639	1.180381
C	3.989629	0.197416	-0.206459
C	5.116092	1.030391	-0.272934
C	4.121205	-1.151314	-0.580721
C	6.346297	0.522597	-0.701733
H	5.045430	2.076388	0.003025
C	5.353516	-1.658932	-0.991491
H	3.260022	-1.806963	-0.551342
C	6.470556	-0.821246	-1.057589
H	7.207476	1.182838	-0.753266
H	5.434561	-2.707164	-1.264377
H	7.428872	-1.213007	-1.386230
C	2.186158	0.198641	2.065862
C	2.964672	-0.853856	2.568309
C	1.267748	0.827751	2.922556
C	2.811485	-1.280058	3.890365
H	3.692791	-1.349055	1.937380
C	1.123568	0.408796	4.243929
H	0.669169	1.658028	2.560732
C	1.890195	-0.653274	4.730587
H	3.420142	-2.100090	4.260808
H	0.406964	0.906375	4.890409
H	1.774225	-0.983931	5.758668
C	0.919265	-2.963034	-0.278608
C	1.566389	-3.315569	0.915586
C	1.317529	-3.591557	-1.471849
C	2.594111	-4.261690	0.915342
H	1.284347	-2.845980	1.849371
C	2.340401	-4.539846	-1.469195

H	0.828547	-3.346017	-2.407884
C	2.987909	-4.873732	-0.275413
H	3.087698	-4.512794	1.849869
H	2.631350	-5.017379	-2.400453
H	3.788662	-5.607531	-0.275245
C	-1.684658	-2.183628	-1.360873
C	-2.518236	-1.254306	-2.003076
C	-1.988317	-3.550018	-1.460020
C	-3.620603	-1.682449	-2.745606
H	-2.304896	-0.194528	-1.924084
C	-3.087874	-3.976639	-2.208387
H	-1.365200	-4.287061	-0.964446
C	-3.904515	-3.045750	-2.855652
H	-4.257332	-0.949659	-3.233116
H	-3.305687	-5.038183	-2.283630
H	-4.759157	-3.380816	-3.436003
C	-1.073087	-1.590615	1.330379
C	-1.404845	-2.816092	1.937873
C	-1.439020	-0.402958	1.969340
C	-2.053054	-2.843304	3.171544
H	-1.154405	-3.752338	1.450385
C	-2.096451	-0.429633	3.203152
H	-1.209268	0.546135	1.507568
C	-2.398020	-1.647999	3.811305
H	-2.292799	-3.797662	3.631265
H	-2.361749	0.504647	3.689173
H	-2.902769	-1.669217	4.772686
C	-2.491231	4.459105	-1.515816
C	-2.646800	3.421364	-0.567353
C	-3.790937	3.374369	0.270075
C	-4.754029	4.377462	0.108733

C	-4.598188	5.396381	-0.831901
C	-3.462415	5.442388	-1.647892
H	-1.602242	4.470158	-2.138896
H	-5.640037	4.358660	0.737282
H	-5.364729	6.160130	-0.927079
H	-3.339462	6.236809	-2.377217
C	-1.638499	2.413915	-0.520697
C	-0.665159	1.613514	-0.611274
H	-0.413499	2.469074	0.202702
C	-4.013637	2.279020	1.282075
C	-4.657357	1.014460	0.675883
H	-4.683629	2.648736	2.064681
H	-3.070231	2.004434	1.765573
H	-5.502120	1.310146	0.040499
H	-3.923104	0.519097	0.017236
N	-5.176066	0.165581	1.747479
C	-5.878762	-1.042369	1.301427
C	-5.000323	-2.161449	0.721753
H	-6.631834	-0.740625	0.560337
H	-6.434906	-1.442336	2.161035
C	-5.817424	-3.383862	0.297159
H	-4.434510	-1.783987	-0.134944
H	-4.261042	-2.452843	1.478008
H	-5.169775	-4.185846	-0.070879
H	-6.403026	-3.782042	1.135268
H	-6.520238	-3.131631	-0.506330
H	-4.399371	-0.116471	2.343353

IVa

C	0.085962	1.642021	-3.087371
C	0.393756	0.272237	-3.341723
C	1.783682	0.065540	-3.103414
C	2.331771	1.320912	-2.689312
C	1.292957	2.296022	-2.687663
H	-0.887163	2.101132	-3.187614
H	-0.318665	-0.470674	-3.672238
H	2.321953	-0.864116	-3.214656
H	3.366068	1.502044	-2.428995
H	1.398672	3.344052	-2.445014
Ru	0.759724	0.692938	-1.104030
P	2.563690	0.454156	0.428807
P	-0.548016	-1.179948	-0.498188
C	3.269567	2.070877	0.968781
C	2.807389	3.283901	0.442643
C	4.295066	2.092313	1.933454
C	3.360964	4.496204	0.865127
H	2.005080	3.286450	-0.282854
C	4.847875	3.301955	2.349517
H	4.660504	1.163748	2.360389
C	4.381467	4.507856	1.815958
H	2.987343	5.428638	0.452052
H	5.639495	3.302983	3.093068
H	4.809825	5.450037	2.145276
C	3.974439	-0.422778	-0.389175
C	5.247261	0.153763	-0.519808
C	3.744536	-1.689690	-0.952596
C	6.268147	-0.532689	-1.183389
H	5.454129	1.137892	-0.116979
C	4.769943	-2.377820	-1.600457
H	2.761793	-2.139230	-0.899615
C	6.036551	-1.800225	-1.719485
H	7.245961	-0.069576	-1.279238

H	4.569534	-3.359493	-2.019255
H	6.833845	-2.330777	-2.231757
C	2.351969	-0.400194	2.050429
C	2.619277	-1.766568	2.211093
C	1.898695	0.340412	3.155484
C	2.420144	-2.383513	3.448558
H	2.989234	-2.357940	1.383289
C	1.715308	-0.275793	4.392087
H	1.691657	1.401041	3.054607
C	1.968686	-1.641912	4.541024
H	2.629384	-3.444031	3.555478
H	1.369416	0.311331	5.237633
H	1.819836	-2.122767	5.503336
C	0.294490	-2.776804	-0.877672
C	0.795277	-3.606043	0.137449
C	0.553363	-3.116340	-2.217493
C	1.554562	-4.735227	-0.180154
H	0.610248	-3.371688	1.178134
C	1.305948	-4.246830	-2.532047
H	0.162619	-2.502275	-3.020106
C	1.817074	-5.056337	-1.512601
H	1.940559	-5.360512	0.619662
H	1.493029	-4.494311	-3.572877
H	2.408913	-5.933356	-1.757608
C	-2.101008	-1.238102	-1.506229
C	-2.754364	-0.034682	-1.816105
C	-2.682074	-2.450214	-1.912958
C	-3.961939	-0.040812	-2.516188
H	-2.325724	0.912067	-1.511018
C	-3.885490	-2.453206	-2.621905
H	-2.198003	-3.394461	-1.687865

C	-4.529047	-1.250275	-2.924626
H	-4.456080	0.901581	-2.734465
H	-4.319552	-3.398172	-2.935493
H	-5.466015	-1.256639	-3.473732
C	-1.214415	-1.443548	1.203352
C	-1.927863	-2.622386	1.492461
C	-1.052411	-0.488202	2.210607
C	-2.450034	-2.839537	2.766070
H	-2.069325	-3.377945	0.727063
C	-1.581289	-0.704350	3.486943
H	-0.508494	0.421537	2.008657
C	-2.277209	-1.880007	3.769173
H	-2.995006	-3.755660	2.973551
H	-1.439759	0.046565	4.257740
H	-2.686641	-2.047683	4.760910
C	-1.947806	4.038257	-1.287188
C	-2.168054	3.335794	-0.088118
C	-3.468390	3.289202	0.461439
C	-4.497727	3.974355	-0.201758
C	-4.273211	4.665151	-1.392905
C	-2.990654	4.688866	-1.945363
H	-0.940232	4.082885	-1.688368
H	-5.498714	3.947534	0.222340
H	-5.092949	5.181696	-1.884213
H	-2.797913	5.225408	-2.870151
C	-1.012251	2.668403	0.576590
C	-0.241757	1.792142	-0.038947
H	-0.776026	2.909207	1.614742
C	-3.815600	2.433836	1.660473
C	-4.580868	1.177426	1.204699
H	-4.436128	2.992953	2.370178

H	-2.911689	2.122297	2.193193
H	-5.546009	1.482235	0.779716
H	-4.019086	0.708353	0.383662
N	-4.837256	0.266570	2.319418
C	-5.769935	-0.820584	1.995637
C	-5.447465	-1.657119	0.745710
H	-6.774656	-0.387183	1.883880
H	-5.812198	-1.483158	2.870271
C	-6.380937	-2.859127	0.589361
H	-5.511108	-1.028350	-0.149222
H	-4.408102	-1.999343	0.806028
H	-6.147035	-3.427463	-0.317754
H	-6.295748	-3.542789	1.443065
H	-7.429599	-2.544109	0.522714
H	-3.949616	-0.147546	2.597730

$TS_{IVa\text{-}Va}$

C	-0.404959	-0.802992	-3.320926
C	0.057933	-2.091346	-2.907212
C	1.473725	-2.076209	-2.864419
C	1.902263	-0.762768	-3.262639
C	0.753115	0.011030	-3.558182
H	-1.433128	-0.521573	-3.485544
H	-0.576973	-2.930510	-2.657509
H	2.117440	-2.899487	-2.591130
H	2.924430	-0.424019	-3.348548
H	0.758028	1.038007	-3.895246
Ru	0.663755	-0.506282	-1.341830
P	2.691990	-0.306078	-0.065956
P	-0.671116	-1.584017	0.265049
C	3.921460	0.834696	-0.863174
C	3.507010	1.794764	-1.796073
C	5.283777	0.762023	-0.527190
C	4.431044	2.662857	-2.382053
H	2.458753	1.861017	-2.057518
C	6.207125	1.631039	-1.110636
H	5.627283	0.027783	0.194815
C	5.783113	2.583702	-2.040948
H	4.092500	3.399309	-3.105633
H	7.256504	1.561720	-0.838698
H	6.502250	3.258175	-2.497062
C	3.685769	-1.885736	-0.052684
C	4.350489	-2.268129	-1.233499
C	3.782461	-2.730641	1.061523
C	5.068480	-3.460989	-1.301562
H	4.329254	-1.622274	-2.103552
C	4.505504	-3.925104	0.994425
H	3.301809	-2.474206	1.996177
C	5.145624	-4.299821	-0.185812
H	5.575340	-3.728997	-2.224413
H	4.559252	-4.561943	1.872583
H	5.705945	-5.228996	-0.235904
C	2.746562	0.227846	1.711210
C	2.059754	-0.510266	2.684577
C	3.424270	1.390449	2.113657
C	2.067675	-0.120034	4.025371
H	1.498873	-1.390834	2.404989
C	3.425554	1.786858	3.451708
H	3.938124	2.004835	1.384798
C	2.751191	1.031565	4.415625
H	1.524960	-0.713110	4.755892
H	3.952809	2.692511	3.738481

H	2.754897	1.341971	5.456522
C	0.072898	-3.107291	1.019917
C	-0.014428	-3.412483	2.387163
C	0.724631	-4.015728	0.169345
C	0.543795	-4.591630	2.888804
H	-0.513425	-2.733956	3.069744
C	1.259780	-5.202995	0.666910
H	0.818119	-3.796936	-0.886997
C	1.175912	-5.493109	2.031027
H	0.475093	-4.805144	3.951714
H	1.759350	-5.890628	-0.008964
H	1.604142	-6.411571	2.421865
C	-2.206303	-2.276114	-0.520016
C	-2.996432	-1.409993	-1.291395
C	-2.620786	-3.606204	-0.358579
C	-4.166732	-1.863884	-1.899486
H	-2.699471	-0.375952	-1.417008
C	-3.795951	-4.059742	-0.967018
H	-2.036716	-4.295119	0.241408
C	-4.570663	-3.192898	-1.739661
H	-4.760712	-1.174732	-2.493420
H	-4.102896	-5.093183	-0.832404
H	-5.481067	-3.548610	-2.213608
C	-1.405011	-0.689407	1.706732
C	-2.516898	-1.221332	2.382698
C	-0.861683	0.524663	2.146194
C	-3.057067	-0.563370	3.487301
H	-2.964739	-2.151141	2.047864
C	-1.415562	1.191054	3.242449
H	-0.013349	0.950709	1.629483
C	-2.510207	0.648553	3.917646
H	-3.913838	-0.990499	4.000347
H	-0.991379	2.136852	3.566866
H	-2.938717	1.167332	4.770648
C	-2.199881	1.973758	-3.197944
C	-2.283000	2.074460	-1.799956
C	-3.559217	2.166827	-1.189732
C	-4.696264	2.132784	-2.009747
C	-4.599787	1.999768	-3.397174
C	-3.343027	1.920298	-3.996341
H	-1.221107	1.954788	-3.662336
H	-5.681946	2.201472	-1.559724
H	-5.501950	1.969235	-4.001814
H	-3.248703	1.832457	-5.075074
C	-1.042783	2.171547	-0.982700
C	-0.082705	1.244137	-0.902987
C	-3.678852	2.290578	0.320206
C	-4.977045	1.745958	0.928436
H	-3.577754	3.348724	0.603163
H	-2.838968	1.767018	0.783678
H	-4.930220	1.888012	2.015358
H	-5.836229	2.344340	0.578101
N	-5.107025	0.314449	0.669450
C	-6.286364	-0.334193	1.237114
C	-7.644770	0.078640	0.644278
H	-6.288799	-0.149174	2.320543
H	-6.161399	-1.418124	1.107127
C	-8.809120	-0.695904	1.267179
H	-7.801115	1.155277	0.785992
H	-7.618763	-0.094467	-0.440873
H	-9.769535	-0.401562	0.830012
H	-8.691069	-1.775953	1.116183

H	-8.867573	-0.518584	2.348000
H	-5.077712	0.138801	-0.330390
H	-0.966646	3.083109	-0.394883
C	1.333795	4.387192	-0.484568
C	1.260587	5.726852	-0.153741
C	1.013407	6.127062	1.168641
C	0.857904	5.113255	2.125286
C	0.932475	3.781375	1.762494
H	1.522042	4.010815	-1.479890
H	1.400617	6.461889	-0.938907
H	0.679894	5.355319	3.167484
H	0.851145	2.955122	2.452643
C	0.897555	7.577832	1.536983
H	1.551605	8.197619	0.917480
H	-0.131494	7.922870	1.376084
H	1.141380	7.742277	2.589695
O	1.257038	2.146327	0.145883
N	1.166178	3.438474	0.471593

Va

C	-0.470055	-0.776632	-3.246942
C	0.028375	-2.059986	-2.845858
C	1.444675	-2.025954	-2.852959
C	1.839720	-0.705229	-3.266094
C	0.668673	0.050494	-3.519594
H	-1.505418	-0.509177	-3.384899
H	-0.581379	-2.912230	-2.577992
H	2.110932	-2.841394	-2.610678
H	2.852582	-0.355053	-3.398975
H	0.652731	1.074562	-3.864403
Ru	0.672133	-0.495505	-1.310831
P	2.707579	-0.362013	-0.094948
P	-0.703636	-1.543391	0.274888
C	3.893987	0.911633	-0.757673
C	3.496392	1.824329	-1.741455
C	5.220356	0.955445	-0.294395
C	4.395463	2.767223	-2.248086
H	2.479978	1.790532	-2.115264
C	6.118857	1.897044	-0.796287
H	5.552404	0.252517	0.463930
C	5.708240	2.807072	-1.775544
H	4.067862	3.466530	-3.012444
H	7.139659	1.918011	-0.425359
H	6.409195	3.537979	-2.168743
C	3.805486	-1.865227	-0.280186
C	4.489012	-2.033123	-1.500232
C	3.964996	-2.853654	0.701020
C	5.278605	-3.157005	-1.738138
H	4.423571	-1.272203	-2.269094
C	4.761329	-3.978782	0.465433
H	3.481721	-2.764871	1.663564
C	5.414839	-4.141429	-0.755079
H	5.794185	-3.257381	-2.689186
H	4.864111	-4.727555	1.245841
H	6.030979	-5.017285	-0.937035
C	2.756008	-0.007524	1.729032
C	2.222302	-0.933035	2.639883
C	3.221337	1.219336	2.231151
C	2.187348	-0.662732	4.008458
H	1.796077	-1.861300	2.286798
C	3.172478	1.497857	3.598803
H	3.613781	1.972624	1.558618
C	2.663482	0.555171	4.495656

H	1.768327	-1.401159	4.686010
H	3.534010	2.456515	3.960569
H	2.630079	0.772225	5.559439
C	-0.006172	-3.069072	1.074431
C	-0.299623	-3.444426	2.395857
C	0.817744	-3.904460	0.305236
C	0.231662	-4.618426	2.934219
H	-0.934182	-2.819771	3.014055
C	1.330602	-5.088852	0.836903
H	1.070631	-3.621207	-0.709163
C	1.044924	-5.446426	2.156078
H	0.004662	-4.886579	3.962300
H	1.970492	-5.717839	0.225188
H	1.455359	-6.360257	2.576113
C	-2.235038	-2.249329	-0.513455
C	-3.054019	-1.387616	-1.258648
C	-2.616952	-3.592616	-0.381404
C	-4.222563	-1.853470	-1.861313
H	-2.778749	-0.347033	-1.369005
C	-3.787555	-4.060468	-0.988101
H	-2.011854	-4.282065	0.196387
C	-4.594176	-3.194668	-1.728211
H	-4.838823	-1.164231	-2.432012
H	-4.066861	-5.104406	-0.875468
H	-5.502765	-3.560508	-2.198006
C	-1.442604	-0.648248	1.717054
C	-2.685844	-1.021819	2.252273
C	-0.730928	0.392794	2.330207
C	-3.195853	-0.379588	3.382252
H	-3.265749	-1.810524	1.788113
C	-1.241725	1.034936	3.459215

H	0.224338	0.694813	1.927246
C	-2.476047	0.650739	3.989257
H	-4.161564	-0.680068	3.777587
H	-0.672911	1.830973	3.931348
H	-2.874093	1.151885	4.867202
C	-2.110442	2.020714	-3.042142
C	-2.228491	2.068348	-1.643903
C	-3.527612	2.101761	-1.071482
C	-4.640216	2.057138	-1.923480
C	-4.502123	1.968657	-3.311401
C	-3.227390	1.951095	-3.875400
H	-1.120359	2.059012	-3.478322
H	-5.640677	2.081504	-1.503524
H	-5.386181	1.927067	-3.941698
H	-3.099057	1.902188	-4.953222
C	-1.024587	2.212227	-0.794947
C	0.053175	1.399252	-0.691788
C	-3.685138	2.169765	0.439024
C	-5.051858	1.747517	0.988954
H	-3.479950	3.197023	0.773723
H	-2.915427	1.543447	0.899194
H	-5.041472	1.889516	2.077275
H	-5.831771	2.425358	0.599469
N	-5.312489	0.333850	0.724953
C	-6.633220	-0.154567	1.113755
C	-7.822007	0.359997	0.283004
H	-6.791715	0.094933	2.172425
H	-6.613936	-1.251700	1.052601
C	-9.154009	-0.239170	0.741819
H	-7.871356	1.454363	0.341151
H	-7.643517	0.109398	-0.772082

H	-9.989977	0.122588	0.133315
H	-9.140084	-1.333723	0.671990
H	-9.365380	0.021645	1.785936
H	-5.152111	0.124017	-0.255406
H	-1.054198	3.112341	-0.181353
C	1.392295	4.224191	-0.524329
C	1.483368	5.565289	-0.206669
C	1.303533	5.998799	1.117107
C	1.028404	5.023591	2.089061
C	0.939479	3.689873	1.738432
H	1.512503	3.822500	-1.519428
H	1.699248	6.272328	-0.999432
H	0.880954	5.297855	3.127674
H	0.748482	2.887663	2.433955
C	1.423200	7.445488	1.491711
H	0.627019	7.735327	2.183899
H	2.377711	7.615902	2.004772
H	1.388777	8.092757	0.612818
O	1.134129	1.971585	0.149723
N	1.120854	3.334264	0.452308

$TS_{Va\text{-}VIa}$

C	-0.431543	-1.515215	-3.094312
C	0.025998	-2.642338	-2.356631
C	1.448462	-2.625744	-2.326934
C	1.874502	-1.468546	-3.064898
C	0.723221	-0.787071	-3.537012
H	-1.457777	-1.288651	-3.328860
H	-0.613652	-3.386228	-1.902622
H	2.090264	-3.359503	-1.863651
H	2.900700	-1.175085	-3.240617
H	0.723706	0.110418	-4.139637
Ru	0.689368	-0.756030	-1.253691
P	2.798837	-0.102314	-0.365197
P	-0.428894	-1.425190	0.700774
C	3.543651	1.333740	-1.276236
C	2.894000	1.873668	-2.394305
C	4.761581	1.902867	-0.864271
C	3.446517	2.953428	-3.089264
H	1.941696	1.462859	-2.706462
C	5.312855	2.982216	-1.555730
H	5.281504	1.504713	0.001338
C	4.656390	3.511286	-2.670845
H	2.924555	3.361491	-3.950294
H	6.254427	3.409234	-1.222524
H	5.085064	4.353006	-3.207245
C	4.061322	-1.446405	-0.605845
C	5.221683	-1.288200	-1.378068
C	3.814885	-2.699895	-0.019631
C	6.117137	-2.350336	-1.541066
H	5.437801	-0.344172	-1.863992
C	4.715830	-3.753376	-0.168396
H	2.901602	-2.864267	0.537904
C	5.873816	-3.582028	-0.932048
H	7.007564	-2.207738	-2.146993
H	4.502454	-4.708299	0.303771
H	6.574506	-4.402563	-1.057181
C	3.120552	0.405528	1.394984
C	3.433670	-0.530702	2.393142
C	2.985337	1.754294	1.767705
C	3.585911	-0.132302	3.723965
H	3.554353	-1.578255	2.152908

C	3.140488	2.152145	3.094733
H	2.766008	2.503664	1.020292
C	3.435253	1.208140	4.082232
H	3.819714	-0.877851	4.478455
H	3.027206	3.200926	3.354605
H	3.551382	1.515374	5.117510
C	0.428224	-2.777186	1.649778
C	0.833523	-2.681512	2.987868
C	0.700059	-3.971140	0.954738
C	1.524351	-3.732488	3.600947
H	0.615925	-1.795361	3.569497
C	1.374102	-5.024114	1.570386
H	0.385725	-4.082933	-0.075794
C	1.802758	-4.902968	2.896149
H	1.838127	-3.629901	4.635970
H	1.570612	-5.935087	1.012201
H	2.338885	-5.717194	3.374912
C	-2.054565	-2.251826	0.339948
C	-2.813180	-1.851084	-0.767773
C	-2.575575	-3.239089	1.192629
C	-4.045930	-2.447858	-1.043937
H	-2.450920	-1.060230	-1.409858
C	-3.813596	-3.826294	0.924993
H	-2.018269	-3.552817	2.069276
C	-4.549425	-3.439083	-0.198813
H	-4.612089	-2.123772	-1.912584
H	-4.201233	-4.588862	1.594589
H	-5.508590	-3.903137	-0.410156
C	-0.951831	-0.207271	2.001585
C	-2.307814	0.105044	2.192994
C	-0.001145	0.426624	2.818352
C	-2.696917	1.005520	3.189968
H	-3.085807	-0.328531	1.575958
C	-0.393221	1.304054	3.830903
H	1.049143	0.229309	2.676536
C	-1.745820	1.595607	4.024583
H	-3.752950	1.230411	3.310534
H	0.366200	1.764761	4.455097
H	-2.052479	2.281629	4.809095
C	-2.327485	0.962765	-3.671690
C	-2.486176	1.381533	-2.341925
C	-3.800763	1.466200	-1.806906
C	-4.878982	1.045553	-2.596452
C	-4.694229	0.572479	-3.898886
C	-3.411833	0.546121	-4.446059
H	-1.333190	0.976182	-4.103142
H	-5.886220	1.082812	-2.193198
H	-5.550771	0.249418	-4.484177
H	-3.253974	0.214424	-5.468681
C	-1.337158	1.815536	-1.519597
C	-0.256181	1.128865	-1.083455
C	-4.013577	2.041515	-0.414470
C	-5.312431	1.651858	0.296460
H	-3.970953	3.138226	-0.483098
H	-3.173294	1.749388	0.221199
H	-5.380171	2.223768	1.230933
H	-6.180234	1.959304	-0.311115
N	-5.304907	0.228726	0.636783
C	-6.552914	-0.303482	1.184835
C	-7.729182	-0.415069	0.200409
H	-6.844948	0.322955	2.039014
H	-6.338802	-1.301946	1.589223

C	-8.976069	-1.018184	0.852476
H	-7.969170	0.573944	-0.209013
H	-7.414255	-1.037812	-0.648438
H	-9.801161	-1.107461	0.137539
H	-8.769123	-2.019431	1.249636
H	-9.324634	-0.397964	1.687096
H	-5.044719	-0.315043	-0.181473
H	-1.435569	2.827354	-1.129066
C	1.130840	4.206004	-0.196537
C	1.071423	5.472244	0.367920
C	0.173273	5.732376	1.415529
C	-0.647798	4.680491	1.854838
C	-0.551984	3.431336	1.261564
H	1.806356	3.926387	-0.996661
H	1.730548	6.248288	-0.005961
H	-1.358565	4.825770	2.661259
H	-1.158284	2.580168	1.538221
C	0.072760	7.097720	2.031149
H	-0.739356	7.658830	1.552466
H	-0.157340	7.034495	3.098255
H	0.995998	7.666275	1.895440
O	0.639819	1.712907	-0.238798
N	0.321232	3.251738	0.266969

Xa

C	0.203056	-0.379758	-3.560946
C	0.077174	1.014064	-3.310742
C	-1.299707	1.304657	-3.094932
C	-2.039475	0.076658	-3.279235
C	-1.113500	-0.956336	-3.544907
H	1.121888	-0.911006	-3.755699
H	0.889560	1.725026	-3.275554
H	-1.717721	2.279982	-2.891378
H	-3.113180	-0.031112	-3.217240
H	-1.344656	-2.002865	-3.694170
Ru	-0.667864	-0.066070	-1.470677
P	-2.689213	0.345387	-0.175547
P	0.810393	1.224698	-0.090569
C	-3.758633	-1.159074	-0.145279
C	-3.633323	-2.176915	-1.101279
C	-4.790899	-1.238596	0.804657
C	-4.535767	-3.242099	-1.121684
H	-2.827213	-2.150580	-1.821355
C	-5.689371	-2.306181	0.784069
H	-4.903654	-0.464340	1.556311
C	-5.568495	-3.307214	-0.183514
H	-4.423575	-4.024696	-1.866456
H	-6.483155	-2.352941	1.523752
H	-6.269073	-4.136987	-0.200100
C	-3.784636	1.621652	-0.920671
C	-4.967338	1.273743	-1.591341
C	-3.394597	2.969986	-0.876929
C	-5.741379	2.260870	-2.207013
H	-5.290315	0.239620	-1.637818
C	-4.175275	3.952501	-1.484229
H	-2.487834	3.253729	-0.362042
C	-5.349713	3.599873	-2.155121
H	-6.653679	1.978470	-2.724317
H	-3.860653	4.990622	-1.432481
H	-5.955940	4.364287	-2.632350

C	-2.564305	0.771568	1.604787
C	-3.283619	1.818150	2.201125
C	-1.730381	-0.030849	2.397077
C	-3.138596	2.075824	3.565577
H	-3.947501	2.439356	1.611883
C	-1.580209	0.237673	3.756874
H	-1.204915	-0.865181	1.951532
C	-2.277787	1.296764	4.343038
H	-3.694994	2.891752	4.017564
H	-0.914815	-0.380336	4.351379
H	-2.158163	1.508500	5.401637
C	0.098722	2.906659	0.186876
C	-0.471683	3.289894	1.409134
C	0.073688	3.814273	-0.888481
C	-1.038115	4.559107	1.557623
H	-0.479829	2.607310	2.249758
C	-0.488775	5.080994	-0.735535
H	0.503697	3.541566	-1.846233
C	-1.045570	5.458603	0.490802
H	-1.477968	4.835638	2.511059
H	-0.492861	5.771157	-1.574092
H	-1.483751	6.445056	0.610637
C	2.419683	1.632965	-0.919881
C	2.981781	0.746921	-1.845127
C	3.127715	2.800569	-0.584866
C	4.215094	1.021567	-2.440123
H	2.467103	-0.169151	-2.097403
C	4.362724	3.071254	-1.173549
H	2.718361	3.509540	0.125889
C	4.908834	2.185517	-2.107326
H	4.631635	0.313176	-3.149340
H	4.896045	3.978502	-0.904600
H	5.868357	2.401495	-2.568235
C	1.425422	0.695854	1.575780
C	2.019141	1.642645	2.429508
C	1.407548	-0.648322	1.974207
C	2.552328	1.259799	3.658745
H	2.064012	2.687374	2.146045
C	1.956023	-1.031329	3.200950
H	0.964003	-1.405190	1.344784
C	2.523403	-0.081023	4.049731
H	2.997305	2.009807	4.305602
H	1.931062	-2.075881	3.491389
H	2.942762	-0.381144	5.005710
C	2.276943	-3.139503	-3.376226
C	2.456802	-2.746401	-2.040962
C	3.764024	-2.462999	-1.572730
C	4.839543	-2.593212	-2.461587
C	4.644147	-2.974438	-3.792165
C	3.356219	-3.240114	-4.257143
H	1.273402	-3.376609	-3.719395
H	5.848762	-2.387061	-2.118747
H	5.497319	-3.062992	-4.459236
H	3.192229	-3.539003	-5.288699
C	1.304605	-2.740703	-1.107644
C	0.315438	-1.856793	-0.932762
C	3.943870	-1.966403	-0.153533
H	3.809985	-2.796827	0.554951
H	3.127146	-1.273944	0.061208
H	1.222368	-3.585584	-0.418551
C	-1.417200	-4.592187	0.949316
C	-2.443615	-4.081214	1.738549

C	-2.145833	-3.547761	2.999056
C	-0.802748	-3.572053	3.394334
C	0.153666	-4.119208	2.537824
H	-1.638396	-4.992200	-0.038203
H	-3.463110	-4.085418	1.369175
H	-0.501712	-3.175307	4.359538
H	1.198564	-4.155670	2.839640
C	-3.226923	-2.954322	3.861924
H	-4.103330	-3.609921	3.898299
H	-2.878527	-2.778689	4.883312
H	-3.556180	-1.995022	3.448249
O	-0.730354	-1.782645	-0.220269
N	-0.129520	-4.625439	1.328794
C	5.252122	-1.229252	0.148393
H	5.524140	-0.601796	-0.707260
H	6.080276	-1.950122	0.285493
N	5.075111	-0.337445	1.294185
H	4.745627	-0.872874	2.095889
C	6.297512	0.375285	1.658967
H	7.125743	-0.307937	1.933127
H	6.638462	0.928041	0.772515
C	6.053930	1.363233	2.800081
H	5.258728	2.054464	2.496412
H	5.671419	0.817959	3.673739
C	7.315726	2.138864	3.184790
H	7.118101	2.842345	4.000953
H	8.114329	1.463251	3.513936
H	7.699796	2.715192	2.334428

VIa

C	0.060215	2.882659	-1.805538
C	0.295879	1.714021	-2.580262
C	1.679069	1.393215	-2.489802
C	2.316384	2.418745	-1.693981
C	1.317345	3.318908	-1.261397
H	-0.890908	3.374100	-1.670164
H	-0.451476	1.157786	-3.126891
H	2.170388	0.565097	-2.979824
H	3.371258	2.483391	-1.466874
H	1.462492	4.177499	-0.619238
Ru	0.842394	1.227571	-0.439696
P	2.833517	0.067483	0.374433
P	-0.586588	-0.708574	-0.482757
C	3.765556	1.140992	1.551884
C	3.564923	2.527273	1.615594
C	4.748496	0.555010	2.368582
C	4.335262	3.313360	2.475220
H	2.800122	2.996850	1.012466
C	5.517127	1.343983	3.224109
H	4.920196	-0.516012	2.336962
C	5.312487	2.725190	3.279824
H	4.163415	4.384999	2.518223
H	6.274008	0.877480	3.847848
H	5.908992	3.337901	3.949515
C	4.058128	-0.300264	-0.946496
C	5.243883	0.439041	-1.074008
C	3.773563	-1.305772	-1.884478
C	6.126680	0.173556	-2.124066
H	5.484424	1.220404	-0.361381
C	4.661828	-1.573111	-2.925206
H	2.864558	-1.883932	-1.797364
C	5.840049	-0.831356	-3.049917

H	7.041110	0.752864	-2.211935
H	4.428656	-2.360450	-3.635833
H	6.530798	-1.037100	-3.862347
C	2.659766	-1.489043	1.332239
C	3.426687	-2.637286	1.086178
C	1.735992	-1.490359	2.387753
C	3.239737	-3.781164	1.864759
H	4.160921	-2.652638	0.289867
C	1.544904	-2.638064	3.155608
H	1.164774	-0.595770	2.601236
C	2.290676	-3.789576	2.889635
H	3.832962	-4.668042	1.662033
H	0.806314	-2.632345	3.951362
C	0.245706	-2.087005	-1.383843
C	0.783990	-3.202769	-0.728073
C	0.390984	-1.982495	-2.779989
C	1.438557	-4.201090	-1.455344
H	0.700618	-3.302470	0.347023
C	1.040827	-2.982033	-3.502404
H	-0.015088	-1.127046	-3.308993
C	1.565875	-4.097369	-2.840972
H	1.852695	-5.055764	-0.929121
H	1.138637	-2.888695	-4.579972
H	2.072633	-4.876343	-3.402920
C	-2.112956	-0.468388	-1.510640
C	-2.696959	0.797550	-1.624247
C	-2.735622	-1.560087	-2.140893
C	-3.866266	0.981634	-2.365612
H	-2.248999	1.644666	-1.125683
C	-3.908200	-1.378030	-2.873598
H	-2.307148	-2.553578	-2.073912
C	-4.474707	-0.105459	-2.993832
H	-4.302137	1.973509	-2.432167
H	-4.376063	-2.231872	-3.355110
H	-5.384829	0.034517	-3.569856
C	-1.313734	-1.494574	1.028718
C	-1.869735	-2.783851	0.957101
C	-1.417216	-0.786501	2.233890
C	-2.484594	-3.355477	2.070157
H	-1.823839	-3.355617	0.037834
C	-2.042813	-1.356221	3.345099
H	-1.007076	0.207353	2.320933
C	-2.572899	-2.644611	3.269893
H	-2.899802	-4.355860	1.995317
H	-2.109085	-0.788062	4.268409
H	-3.054185	-3.091347	4.135098
C	-2.300655	4.662115	0.027621
C	-2.498544	3.443802	0.695977
C	-3.793528	2.868464	0.717411
C	-4.839942	3.543832	0.074716
C	-4.625804	4.750406	-0.597159
C	-3.348263	5.309664	-0.630849
H	-1.309183	5.107035	0.036118
H	-5.839641	3.120531	0.086746
H	-5.455974	5.248576	-1.090449
H	-3.169633	6.247928	-1.148610
C	-1.387085	2.830410	1.464740
C	-0.325741	2.114674	1.070127
C	-3.990977	1.518219	1.373635
H	-3.996759	1.629125	2.467841
H	-3.112865	0.909018	1.151169
H	-1.426203	2.958712	2.549413

O	0.689644	1.612352	1.645470
H	2.138289	-4.686315	3.483207
C	-5.219525	0.720070	0.924804
H	-5.372680	0.861234	-0.150319
H	-6.131693	1.097469	1.424592
N	-4.999326	-0.709767	1.141661
H	-4.795212	-0.878446	2.125523
C	-6.140357	-1.529029	0.737615
H	-6.344632	-1.315817	-0.320852
H	-7.067378	-1.273017	1.288155
C	-5.849891	-3.021280	0.898842
H	-5.606986	-3.230423	1.949596
H	-4.951060	-3.265410	0.320764
C	-7.022369	-3.897845	0.452838
H	-6.794370	-4.962158	0.576380
H	-7.926349	-3.681587	1.034681
H	-7.261302	-3.729221	-0.604179

VIIa

C	-3.688405	-0.840467	-0.117031
C	-2.602404	0.054406	-0.066067
C	-1.284890	-0.462279	0.054585
C	-1.119675	-1.851236	0.122382
C	-2.205161	-2.729413	0.071332
C	-3.497094	-2.218231	-0.049951
H	-4.695240	-0.441565	-0.209669
H	-0.121774	-2.265888	0.216204
H	-2.037667	-3.801260	0.126016
H	-4.353966	-2.884895	-0.091596
C	-2.922418	1.485532	-0.143947
C	-2.138023	2.550035	-0.101159
C	-0.109213	0.488385	0.112242
C	1.282678	-0.139941	0.218526
H	-0.231498	1.170856	0.965103
H	-0.123644	1.122461	-0.787827
H	1.346344	-0.752427	1.126985
H	1.440864	-0.821470	-0.633777
N	2.292677	0.915167	0.320602
C	3.659488	0.436493	0.546955
C	4.287421	-0.416993	-0.568140
H	3.664733	-0.134650	1.485939
H	4.294353	1.316804	0.715980
C	5.735722	-0.801887	-0.253674
H	3.692254	-1.325394	-0.722120
H	4.246396	0.147955	-1.510066
H	6.175257	-1.404423	-1.056003
H	6.363054	0.088345	-0.123374
H	5.796599	-1.386423	0.672363
H	2.277274	1.467324	-0.536196
O	-1.525087	3.550442	-0.073683
H	-3.969534	1.768530	-0.249015

c. Coordinates for Metal-free lactamization of VIIa. VIIa to 2a.

$TS_{VIIa\text{-}IXa}$

C	-2.388171	-0.075206	-1.444283
C	-1.452979	-0.590864	-0.526616
C	-1.456949	-0.083480	0.800715
C	-2.335897	0.952068	1.136860

C	-3.234640	1.476384	0.204479
C	-3.268588	0.945240	-1.087816
H	-2.406704	-0.477449	-2.454396
H	-2.331167	1.335614	2.154792
H	-3.911544	2.276879	0.489486
H	-3.974873	1.329743	-1.819013
C	-0.516084	-1.641637	-0.936878
C	0.760346	-1.702956	-0.492785
C	-0.582471	-0.727368	1.855821
C	0.921579	-0.456371	1.728696
H	-0.736581	-1.812905	1.815009
H	-0.890738	-0.396180	2.853053
H	1.147397	0.575005	2.007654
H	1.475721	-1.114555	2.403931
N	1.438355	-0.671177	0.339161
C	1.711359	0.592475	-0.412496
C	3.002812	1.262527	0.053154
H	0.853490	1.265205	-0.308048
H	1.797196	0.322986	-1.468713
C	3.274888	2.548571	-0.731919
H	2.947854	1.491206	1.123991
H	3.831414	0.555443	-0.077496
H	4.207367	3.018291	-0.402858
H	3.361644	2.346904	-1.805821
H	2.466205	3.275391	-0.593125
H	2.275856	-1.629989	0.064403
O	1.755675	-2.534201	-0.697342
H	-0.837658	-2.386044	-1.659832

IXa

C	1.820411	-0.759219	-0.305558
C	1.802080	0.638702	-0.075497
C	0.618068	1.464426	-0.272603
C	0.569485	-1.451516	-0.781256
C	-0.712131	1.163742	-0.199588
C	-0.626611	-1.297873	0.169256
H	-0.290743	-1.432418	1.208195
H	0.767922	-2.521254	-0.900191
H	-1.350754	-2.092422	-0.037929
C	3.013143	1.263824	0.295905
H	3.019178	2.340388	0.450863
C	3.001243	-1.480263	-0.102797
H	2.994670	-2.554793	-0.273719
C	4.186598	0.535478	0.476535
H	5.099692	1.047935	0.768747
C	4.185467	-0.849616	0.287791
H	5.093501	-1.428621	0.430716
O	-1.557999	2.236744	-0.358250
H	-2.345898	1.954522	-0.850068
N	-1.353400	-0.034494	0.028941
H	0.802779	2.510989	-0.496589
H	0.275901	-1.069176	-1.768179
C	-2.685939	-0.050318	0.660712
C	-3.799001	-0.495748	-0.295296
H	-2.909818	0.941097	1.061877
H	-2.646267	-0.730275	1.521495
C	-5.151806	-0.597744	0.412603
H	-3.864283	0.214811	-1.130305
H	-3.530684	-1.464543	-0.734947
H	-5.940262	-0.908481	-0.280602
H	-5.116364	-1.329517	1.228339
H	-5.446815	0.366021	0.844461

IX’a

C	-2.936265	1.206433	-0.310242
C	-1.667268	0.607796	-0.109536
C	-1.622514	-0.810515	-0.021820
C	-2.811630	-1.546060	-0.094118
C	-4.051510	-0.935324	-0.287793
C	-4.104211	0.457328	-0.401852
H	-2.992058	2.289879	-0.390465
H	-2.755768	-2.629288	-0.006575
H	-4.955975	-1.533696	-0.350208
H	-5.055568	0.960551	-0.555499
C	-0.518004	1.498508	-0.039119
C	0.805748	1.295724	0.264666
C	-0.312045	-1.527573	0.163864
C	0.569378	-0.903442	1.273337
H	0.268057	-1.519837	-0.767535
H	-0.503767	-2.578299	0.406848
H	-0.071758	-0.559023	2.093251
H	1.239603	-1.662973	1.680110
N	1.405453	0.203861	0.828092
C	2.834009	-0.058990	0.593147
C	3.140442	-0.681964	-0.775497
H	3.174189	-0.724921	1.392700
H	3.385272	0.876512	0.703106
C	4.641729	-0.903873	-0.973524
H	2.606046	-1.634972	-0.871327
H	2.750007	-0.018990	-1.557657
H	4.852382	-1.347120	-1.952519
H	5.192954	0.041830	-0.908882
H	5.047263	-1.577153	-0.208744
H	1.290688	2.984350	-0.488384
O	1.704311	2.310230	0.071094
H	-0.751390	2.535989	-0.272704

TS$_{IX'a-2a}$

C	-3.043281	1.116547	-0.179309
C	-1.732798	0.606193	-0.028293
C	-1.572747	-0.803826	-0.017800
C	-2.701801	-1.622386	-0.174502
C	-3.983427	-1.099339	-0.339852
C	-4.149798	0.289142	-0.334467
H	-3.178076	2.196064	-0.181974
H	-2.565321	-2.701849	-0.156164
H	-4.835982	-1.761900	-0.459147
H	-5.138742	0.725523	-0.450136
C	-0.645009	1.591338	0.090088
C	0.786736	1.345560	0.201603
C	-0.227674	-1.475199	0.185967
C	0.680724	-0.822410	1.245068
H	0.334782	-1.514469	-0.756118
H	-0.400213	-2.515256	0.482605
H	0.093402	-0.487370	2.105728
H	1.411068	-1.549413	1.603780
N	1.444362	0.314529	0.709236
C	2.896999	0.168381	0.520713
C	3.255316	-0.774745	-0.632709
H	3.315882	-0.200062	1.463362
H	3.308521	1.162707	0.341103
C	4.771154	-0.916163	-0.789199
H	2.805234	-1.759149	-0.457071

H	2.812325	-0.381860	-1.555849
H	5.019822	-1.585433	-1.619149
H	5.240630	0.054083	-0.988730
H	5.226662	-1.326648	0.119678
H	0.202301	2.517890	-0.733689
O	1.391967	2.357893	-0.366525
H	-0.904924	2.443732	0.725474

$TS_{VIIa\text{-}2a}$

C	-2.694455	0.756485	-1.239388
C	-1.617894	0.624097	-0.338975
C	-1.665234	-0.425946	0.614869
C	-2.751281	-1.310717	0.605205
C	-3.800830	-1.177756	-0.304831
C	-3.771625	-0.125777	-1.224622
H	-2.673464	1.568445	-1.962426
H	-2.776966	-2.111528	1.341063
H	-4.632998	-1.875657	-0.287534
H	-4.585409	0.006718	-1.932892
C	-0.476245	1.565868	-0.475665
C	0.673005	1.552865	0.412419
C	-0.595863	-0.560228	1.680318
C	0.807582	-0.875675	1.154969
H	-0.872910	-1.348157	2.387102
H	-0.547671	0.372072	2.261829
H	1.527989	-0.892652	1.985582
H	0.821296	-1.855302	0.670222
N	1.232126	0.136823	0.153628
C	2.682954	0.161020	-0.127988
C	3.198392	-1.159558	-0.699751
H	3.221170	0.427390	0.791427
H	2.854299	0.966029	-0.850697
C	4.671100	-1.050140	-1.105137
H	3.082523	-1.960918	0.038465
H	2.586579	-1.434359	-1.568211
H	5.038981	-2.000424	-1.505430
H	4.815073	-0.283238	-1.874972
H	5.298942	-0.783457	-0.246971
H	0.444777	0.518015	-0.882122
O	1.320355	2.416415	0.963496
H	-0.747578	2.550689	-0.856343

VIIa-H2O

C	2.680301	0.625767	1.399761
C	1.636226	-0.030774	0.705607
C	1.917796	-0.498509	-0.603879
C	3.173973	-0.271606	-1.174312
C	4.185857	0.392537	-0.477316
C	3.929468	0.837215	0.823256
H	2.491802	0.975202	2.412284
H	3.362676	-0.634776	-2.182408
H	5.156248	0.553109	-0.938279
H	4.702923	1.350887	1.388742
C	0.385491	-0.265029	1.412806
C	-0.903233	-0.547799	1.034589
C	0.849941	-1.224534	-1.377664
C	-0.389863	-0.356165	-1.591907
H	0.552013	-2.146867	-0.857734
H	1.233551	-1.520893	-2.358120
H	-0.106639	0.690028	-1.707274
H	-0.943736	-0.662421	-2.483301
N	-1.368251	-0.462162	-0.460828

C	-2.467760	0.574499	-0.578178
C	-2.031463	1.983069	-0.188892
H	-3.270492	0.231672	0.074587
H	-2.817607	0.535599	-1.613614
C	-3.198966	2.963495	-0.338289
H	-1.677904	1.978783	0.847145
H	-1.192706	2.312603	-0.811397
H	-2.894763	3.974347	-0.049218
H	-3.556079	3.003984	-1.373850
H	-4.043700	2.672986	0.296629
H	-1.861098	-1.372531	-0.563461
O	-1.885966	-0.841707	1.768483
H	0.468706	-0.255153	2.497877
O	-3.026307	-2.650937	0.131268
H	-2.541688	-3.482087	0.243440
H	-2.792174	-2.121165	0.932252

$TS_{VIIa\text{-}IX'a}$-H_2O

C	2.419900	0.130178	1.478050
C	1.362568	-0.475805	0.771128
C	1.579569	-0.848246	-0.576581
C	2.805267	-0.559938	-1.185475
C	3.834622	0.068719	-0.480680
C	3.639662	0.404406	0.862174
H	2.268276	0.397169	2.521143
H	2.958909	-0.847322	-2.223177
H	4.780641	0.281615	-0.970500
H	4.435489	0.881764	1.427791
C	0.101228	-0.755734	1.455590
C	-1.160160	-0.727013	0.957394
C	0.489905	-1.571228	-1.326729
C	-0.719578	-0.692457	-1.627000
H	0.161448	-2.450661	-0.757529
H	0.869617	-1.931684	-2.288358
H	-0.391654	0.210556	-2.145211
H	-1.412693	-1.222920	-2.288785
N	-1.523311	-0.277410	-0.422259
C	-1.878149	1.187831	-0.423191
C	-0.717228	2.144859	-0.164849
H	-2.651696	1.310853	0.340082
H	-2.339857	1.385726	-1.395985
C	-1.183972	3.600726	-0.257810
H	-0.299351	1.947003	0.826636
H	0.087187	1.965666	-0.886755
H	-0.355903	4.288055	-0.057172
H	-1.576587	3.831270	-1.255067
H	-1.976734	3.810666	0.469602
H	-2.610847	-0.799171	-0.519761
O	-2.235235	-0.982075	1.672673
H	0.155642	-0.997210	2.514509
O	-3.761348	-1.285093	-0.145969
H	-3.794940	-2.221292	-0.393628
H	-3.112916	-1.198651	0.932215

IX’a-H2O

C	2.804679	0.235351	1.509127
C	1.754546	-0.172864	0.659940
C	2.002833	-0.222165	-0.732621
C	3.247715	0.176603	-1.228530
C	4.271589	0.598042	-0.375879
C	4.044926	0.618740	1.002828
H	2.632149	0.254182	2.582746

H	3.420645	0.141751	-2.302039
H	5.233806	0.896653	-0.782334
H	4.831621	0.934726	1.682988
C	0.494430	-0.602827	1.259909
C	-0.774787	-0.644679	0.773666
C	0.917695	-0.700270	-1.662194
C	-0.371192	0.127956	-1.564680
H	0.673566	-1.751410	-1.455855
H	1.275133	-0.653125	-2.695372
H	-0.108068	1.188828	-1.456030
H	-0.937953	0.031259	-2.496512
N	-1.281167	-0.305889	-0.492687
C	-2.541602	0.484254	-0.477826
C	-2.419343	1.860750	0.186710
H	-3.321811	-0.091890	0.024051
H	-2.853604	0.593259	-1.521526
C	-3.742093	2.629117	0.135058
H	-2.108949	1.720550	1.228973
H	-1.630587	2.442893	-0.304148
H	-3.652882	3.605011	0.623866
H	-4.061177	2.802181	-0.899749
H	-4.541662	2.074623	0.640697
H	-2.324320	-2.149328	-0.788389
O	-1.743997	-1.075751	1.636444
H	0.563237	-0.962124	2.283307
O	-2.931476	-2.677633	-0.234726
H	-2.516426	-3.552058	-0.179191
H	-2.337141	-1.685730	1.136523

IXa-H2O

C	3.035230	0.896618	-0.486008
C	1.790938	0.270105	-0.212450
C	1.835041	-1.067502	0.276511
C	3.073926	-1.702122	0.438480
C	4.283677	-1.063091	0.166410
C	4.253679	0.255258	-0.299485
H	3.026653	1.919405	-0.856760
H	3.082743	-2.729755	0.796608
H	5.226528	-1.582409	0.312546
H	5.178589	0.781222	-0.523288
C	0.597234	1.070023	-0.444178
C	-0.746907	0.793444	-0.317570
C	0.578179	-1.836720	0.601925
C	-0.547013	-1.630714	-0.436229
H	0.177481	-1.544617	1.582785
H	0.818715	-2.903571	0.670608
H	-0.117905	-1.586780	-1.443515
H	-1.235101	-2.478496	-0.412555
N	-1.332876	-0.431737	-0.195256
C	-2.635126	-0.587634	0.461805
C	-3.773939	-0.852810	-0.528046
H	-2.849837	0.315593	1.036756
H	-2.556581	-1.418109	1.176279
C	-5.113170	-1.067835	0.180347
H	-3.837120	-0.000540	-1.214905
H	-3.525231	-1.732229	-1.136068
H	-5.918856	-1.249553	-0.538725
H	-5.070337	-1.929206	0.857887
H	-5.390707	-0.190589	0.777077
H	0.342028	3.485576	1.076992
O	-1.661253	1.795086	-0.370849
H	0.799876	2.084166	-0.778736

O	-0.396874	3.950998	0.652943
H	-0.911802	4.315961	1.389579
H	-1.234491	2.635300	-0.071599

$TS_{IXa\text{-}2a}$-H_2O

C	2.979095	0.849392	-0.716172
C	1.763434	0.291429	-0.256035
C	1.819978	-0.993275	0.337590
C	3.056186	-1.647050	0.453703
C	4.244716	-1.072621	0.007219
C	4.197154	0.190387	-0.590078
H	2.954057	1.835220	-1.175519
H	3.076696	-2.638571	0.901146
H	5.186380	-1.603680	0.113542
H	5.105453	0.660363	-0.958430
C	0.567193	1.156506	-0.390099
C	-0.838145	0.854448	-0.178301
C	0.573892	-1.714685	0.797200
C	-0.598039	-1.578980	-0.190843
H	0.234639	-1.348957	1.775816
H	0.805045	-2.777297	0.926149
H	-0.231268	-1.606587	-1.221729
H	-1.289858	-2.413742	-0.069393
N	-1.374304	-0.355168	0.024593
C	-2.768325	-0.493318	0.473013
C	-3.741155	-0.711413	-0.689880
H	-3.040358	0.407894	1.024776
H	-2.806816	-1.339922	1.167704
C	-5.180051	-0.888307	-0.200571
H	-3.671509	0.149979	-1.364361
H	-3.427426	-1.592987	-1.263238
H	-5.866968	-1.039157	-1.039754
H	-5.269433	-1.755511	0.464570
H	-5.519772	-0.006344	0.355055
H	0.544258	2.306588	0.451365
O	-1.646936	1.880604	-0.107725
H	0.648747	1.834905	-1.244407
O	0.000152	3.358059	0.824390
H	-0.098073	3.302389	1.788356
H	-0.994978	2.747214	0.322411

2a

C	-2.833990	1.020900	-0.517593
C	-1.666927	0.618584	0.154800
C	-1.458200	-0.755986	0.392970
C	-2.430687	-1.668636	-0.050197
C	-3.585998	-1.257363	-0.709888
C	-3.790382	0.103706	-0.946469
H	-2.988363	2.080645	-0.704147
H	-2.267430	-2.728930	0.131101
H	-4.317097	-1.991216	-1.037627
H	-4.682267	0.448041	-1.462481
C	-0.697337	1.708090	0.588956
C	0.739927	1.498342	0.112609
C	-0.237963	-1.353985	1.080311
C	0.786798	-0.396259	1.693736
H	0.279505	-1.997916	0.358416
H	-0.584842	-2.020092	1.881052
H	0.328242	0.193496	2.494141
H	1.576025	-0.989378	2.161398
N	1.432051	0.494317	0.724338
C	2.801804	0.205049	0.291610

C	2.889156	-0.889340	-0.779681
H	3.383534	-0.084206	1.174492
H	3.222843	1.134408	-0.098366
C	4.330272	-1.119831	-1.240158
H	2.470743	-1.822655	-0.383507
H	2.261250	-0.594028	-1.628915
H	4.382776	-1.902769	-2.004047
H	4.759099	-0.205863	-1.667850
H	4.969731	-1.426725	-0.403669
H	-1.013555	2.661356	0.163087
O	1.228763	2.210524	-0.768631
H	-0.713480	1.821801	1.679558

d. Coordinates for Metal-bound lactamization of VIa. VIa to XVa.

$TS_{VIa-XIVa}$

C	0.100402	-2.250960	-2.480353
C	-0.243708	-0.950292	-2.942785
C	-1.659525	-0.776990	-2.818020
C	-2.202332	-2.019485	-2.319413
C	-1.120403	-2.900688	-2.083772
H	1.089090	-2.681736	-2.470281
H	0.448817	-0.215947	-3.327852
H	-2.224796	0.089626	-3.128265
H	-3.247610	-2.227401	-2.137493
H	-1.193918	-3.893290	-1.656519
Ru	-0.864857	-1.043886	-0.813190
P	-2.854172	-0.307791	0.314362
P	0.468337	0.887272	-0.372482
C	-3.708661	-1.676446	1.222772
C	-3.521322	-3.016563	0.851019
C	-4.619668	-1.380152	2.251260
C	-4.225648	-4.037048	1.493457
H	-2.820331	-3.270005	0.067002
C	-5.321360	-2.401507	2.892496
H	-4.788513	-0.351544	2.551864
C	-5.125722	-3.732837	2.516387
H	-4.065016	-5.069353	1.195754
H	-6.021220	-2.154989	3.685731
H	-5.670388	-4.527307	3.018366
C	-4.139970	0.263622	-0.877682
C	-5.305425	-0.475734	-1.125057
C	-3.918238	1.448964	-1.598621
C	-6.227337	-0.038218	-2.080753
H	-5.501742	-1.391709	-0.578524
C	-4.845425	1.888470	-2.542511
H	-3.024967	2.032020	-1.419009
C	-6.001709	1.142462	-2.790229
H	-7.124592	-0.622237	-2.264509
H	-4.659530	2.811782	-3.083556
H	-6.721982	1.481536	-3.529080
C	-2.813958	1.007476	1.603204
C	-3.631892	2.145839	1.590303
C	-1.921691	0.821686	2.669179
C	-3.525260	3.098357	2.606654
H	-4.346432	2.303556	0.791428
C	-1.808920	1.776864	3.677859
H	-1.315819	-0.075141	2.710026
C	-2.605843	2.924854	3.643230
H	-4.159515	3.979827	2.581624
H	-1.094979	1.627011	4.482160

C	-0.422822	2.415568	-0.900763
C	-1.009126	3.292982	0.021687
C	-0.593344	2.660987	-2.275904
C	-1.744789	4.395784	-0.421923
H	-0.903961	3.121659	1.086477
C	-1.322140	3.765401	-2.715070
H	-0.144082	1.997212	-3.006640
C	-1.902526	4.636836	-1.787152
H	-2.197935	5.060231	0.307597
H	-1.438123	3.943940	-3.780165
H	-2.473158	5.495715	-2.128122
C	2.017314	0.965250	-1.395003
C	2.713043	-0.217029	-1.677528
C	2.549728	2.186612	-1.843356
C	3.897213	-0.190975	-2.416018
H	2.336596	-1.163070	-1.312849
C	3.736890	2.213230	-2.576966
H	2.038197	3.119883	-1.635427
C	4.409879	1.024030	-2.872686
H	4.419031	-1.120956	-2.616084
H	4.132526	3.164758	-2.920611
H	5.330303	1.047508	-3.448878
C	1.173058	1.332351	1.283520
C	1.765005	2.591374	1.492096
C	1.227326	0.389110	2.316410
C	2.378491	2.894997	2.707091
H	1.749877	3.340308	0.708196
C	1.849331	0.688407	3.531603
H	0.784723	-0.585512	2.181896
C	2.425510	1.943483	3.730792
H	2.828126	3.873124	2.849914

H	1.880938	-0.061558	4.316352
H	2.911034	2.179565	4.673035
C	2.881902	-3.829909	-0.861471
C	2.981673	-3.129649	0.353279
C	4.233738	-2.626722	0.777168
C	5.342849	-2.841356	-0.053400
C	5.233201	-3.512354	-1.270996
C	3.993225	-4.008197	-1.682162
H	1.922111	-4.252605	-1.142516
H	6.313724	-2.465397	0.255563
H	6.113427	-3.653905	-1.891688
H	3.895917	-4.546356	-2.620580
C	1.785131	-2.972906	1.204392
C	0.560934	-2.578107	0.923350
C	4.401233	-1.896481	2.099712
H	4.859909	-2.575277	2.831520
H	3.419329	-1.629324	2.500702
H	1.858886	-3.198325	2.271853
O	-0.611655	-2.333517	1.047342
H	-2.516615	3.674061	4.424617
C	5.243757	-0.613639	2.037932
H	6.260227	-0.845426	1.702612
H	5.343430	-0.216760	3.066665
N	4.650314	0.347567	1.116840
H	3.694053	0.526302	1.407247
C	5.354764	1.624367	1.005580
H	5.603834	2.058336	1.994315
H	4.668343	2.327042	0.519484
C	6.631487	1.525524	0.164141
H	6.376530	1.041233	-0.785635
H	7.361868	0.876995	0.664129

C	7.258387	2.897643	-0.094143
H	8.179257	2.813888	-0.681441
H	6.568222	3.546651	-0.647006
H	7.508943	3.405594	0.845140

XIVa

C	-0.758969	1.018539	-2.877165
C	0.082530	-0.084207	-3.168536
C	1.451457	0.330017	-3.033671
C	1.439713	1.739730	-2.724764
C	0.089846	2.152872	-2.604304
H	-1.840021	1.014598	-2.868813
H	-0.257883	-1.078928	-3.419772
H	2.325606	-0.274839	-3.226313
H	2.312990	2.361450	-2.577966
H	-0.254422	3.146748	-2.349364
Ru	0.509755	0.553071	-1.076011
P	2.397389	1.013828	0.278330
P	0.011770	-1.636016	-0.332397
C	2.505158	2.797282	0.768214
C	1.576583	3.726371	0.279815
C	3.512764	3.245771	1.642557
C	1.647020	5.071883	0.652587
H	0.796488	3.400083	-0.395630
C	3.588310	4.589089	2.007573
H	4.239385	2.543990	2.039623
C	2.653387	5.505934	1.515546
H	0.914627	5.775535	0.266860
H	4.375319	4.920002	2.679041
H	2.710975	6.550878	1.806406
C	3.954383	0.712786	-0.676968
C	4.894143	1.720046	-0.941534
C	4.169699	-0.569283	-1.212126
C	6.029042	1.444062	-1.710426
H	4.751226	2.723645	-0.558118
C	5.310207	-0.845593	-1.965214
H	3.439633	-1.352423	-1.053628
C	6.244635	0.162317	-2.218806
H	6.744293	2.237251	-1.908588
H	5.457866	-1.846555	-2.360150
H	7.129513	-0.049117	-2.812218
C	2.705810	0.202670	1.917120
C	3.470918	-0.965483	2.041831
C	2.112803	0.745285	3.071339
C	3.627142	-1.583579	3.285336
H	3.955453	-1.402615	1.178178
C	2.277527	0.132035	4.312449
H	1.521048	1.652866	3.006709
C	3.031227	-1.039421	4.423489
H	4.222602	-2.489231	3.358508
H	1.814051	0.569481	5.192012
C	1.352850	-2.854270	-0.675895
C	2.090031	-3.442315	0.362468
C	1.738045	-3.105130	-2.005801
C	3.195547	-4.248792	0.078199
H	1.819190	-3.262357	1.395821
C	2.832995	-3.921329	-2.286799
H	1.185572	-2.663838	-2.827279
C	3.571584	-4.489489	-1.243739
H	3.760673	-4.686802	0.895919
H	3.112267	-4.107975	-3.319693
H	4.430840	-5.116656	-1.462618

C	-1.464287	-2.281244	-1.249826
C	-2.577685	-1.430733	-1.360172
C	-1.530750	-3.563988	-1.811719
C	-3.720777	-1.844066	-2.043636
H	-2.557835	-0.443232	-0.911344
C	-2.681371	-3.978559	-2.489939
H	-0.689777	-4.244015	-1.729213
C	-3.774707	-3.119447	-2.614195
H	-4.566729	-1.168003	-2.125994
H	-2.717903	-4.974677	-2.921887
H	-4.664205	-3.441281	-3.147965
C	-0.495638	-2.029532	1.398870
C	-0.940480	-3.326163	1.717942
C	-0.501032	-1.046542	2.393465
C	-1.374819	-3.625943	3.007463
H	-0.950408	-4.101418	0.957979
C	-0.937729	-1.348420	3.686956
H	-0.166041	-0.046779	2.166013
C	-1.375899	-2.635366	3.996131
H	-1.716477	-4.630270	3.240044
H	-0.930990	-0.574410	4.448664
H	-1.719211	-2.869153	4.999704
C	-3.700769	3.071653	-1.896434
C	-3.729161	3.022579	-0.494111
C	-4.924532	2.714383	0.182889
C	-6.075857	2.482065	-0.584346
C	-6.047139	2.527872	-1.977934
C	-4.852835	2.821393	-2.641117
H	-2.766074	3.305875	-2.397479
H	-7.006207	2.247238	-0.073061
H	-6.954065	2.335739	-2.544366
H	-4.818372	2.859692	-3.725956
C	-2.455695	3.285888	0.259764
C	-1.606111	2.305642	0.397633
C	-4.993592	2.567973	1.687349
H	-5.865196	3.112166	2.069608
H	-4.108219	3.009008	2.156502
H	-2.212285	4.252179	0.695939
O	-0.873242	1.377429	0.504471
H	3.156598	-1.519397	5.389658
C	-5.121550	1.097608	2.122601
H	-6.033684	0.684395	1.679755
H	-5.256561	1.056450	3.219227
N	-3.984913	0.300419	1.661989
H	-3.141494	0.651919	2.111470
C	-4.091010	-1.133461	1.963721
H	-4.215250	-1.328418	3.045443
H	-3.141593	-1.588269	1.671394
C	-5.222877	-1.832432	1.201713
H	-5.249510	-1.439731	0.179449
H	-6.193118	-1.597060	1.655943
C	-5.025720	-3.350599	1.169531
H	-5.858794	-3.852122	0.665052
H	-4.104622	-3.612883	0.637092
H	-4.951769	-3.763556	2.182954

$TS_{XIVa\text{-}XVa}$

C	-0.591418	0.444564	-3.119768
C	0.383512	-0.581662	-3.198386
C	1.686373	0.006152	-3.035992
C	1.499565	1.433811	-2.936399
C	0.109232	1.696794	-2.957407

H	-1.663183	0.315076	-3.177720
H	0.175287	-1.634877	-3.328561
H	2.633041	-0.512431	-3.081589
H	2.286617	2.167711	-2.824275
H	-0.355578	2.669901	-2.863227
Ru	0.576100	0.371152	-1.201802
P	2.314672	1.229930	0.168637
P	0.249789	-1.736274	-0.191737
C	2.230808	3.067168	0.385201
C	1.241602	3.823251	-0.257278
C	3.159786	3.729782	1.209443
C	1.180502	5.209846	-0.088076
H	0.511033	3.330572	-0.885002
C	3.102005	5.112814	1.373628
H	3.929330	3.163561	1.724803
C	2.110556	5.857076	0.725494
H	0.403123	5.778615	-0.590502
H	3.828444	5.609125	2.010659
H	2.063639	6.934054	0.859149
C	3.951759	0.951458	-0.652223
C	4.778675	2.004157	-1.072512
C	4.345145	-0.368705	-0.934181
C	5.977184	1.739185	-1.742254
H	4.497920	3.034693	-0.888474
C	5.548750	-0.630596	-1.588060
H	3.706385	-1.196775	-0.655441
C	6.369566	0.424220	-1.996623
H	6.602778	2.567496	-2.062907
H	5.834030	-1.659863	-1.785404
H	7.303253	0.222427	-2.513790
C	2.586021	0.711392	1.928906
C	3.441627	-0.341753	2.282337
C	1.860490	1.359464	2.945165
C	3.556848	-0.747075	3.614880
H	4.028998	-0.853117	1.530843
C	1.983939	0.959178	4.274885
H	1.195321	2.182250	2.701900
C	2.828663	-0.101252	4.614621
H	4.224051	-1.566990	3.866314
H	1.417250	1.475067	5.044772
C	1.739326	-2.819467	-0.265860
C	2.445157	-3.174739	0.892817
C	2.255781	-3.198691	-1.519022
C	3.649447	-3.878307	0.799687
H	2.071552	-2.891758	1.869867
C	3.449381	-3.913614	-1.607953
H	1.727397	-2.935678	-2.429111
C	4.155552	-4.247931	-0.447285
H	4.189213	-4.135105	1.706974
H	3.831418	-4.202892	-2.582832
H	5.091034	-4.795402	-0.516814
C	-1.061656	-2.691189	-1.087166
C	-2.258472	-2.021017	-1.396479
C	-0.932646	-4.046164	-1.427247
C	-3.296336	-2.689585	-2.046178
H	-2.383168	-0.975907	-1.128427
C	-1.974065	-4.712966	-2.079719
H	-0.023753	-4.588607	-1.188606
C	-3.155198	-4.037414	-2.393605
H	-4.214641	-2.157927	-2.278941
H	-1.858024	-5.761709	-2.340444
H	-3.962408	-4.556067	-2.902559

C	-0.341462	-1.927540	1.544117
C	-0.699066	-3.196430	2.036227
C	-0.477961	-0.812494	2.375684
C	-1.183012	-3.336394	3.335001
H	-0.603093	-4.073135	1.403124
C	-0.966036	-0.953200	3.678695
H	-0.206633	0.167260	2.009929
C	-1.321577	-2.213121	4.158988
H	-1.457493	-4.320486	3.704034
H	-1.066122	-0.075837	4.310690
H	-1.704925	-2.323646	5.169155
C	-5.129195	2.248300	-2.175764
C	-4.428813	2.342013	-0.958724
C	-5.144700	2.442910	0.254090
C	-6.546569	2.402364	0.198991
C	-7.235584	2.284312	-1.006988
C	-6.520298	2.213957	-2.205527
H	-4.565533	2.196744	-3.103877
H	-7.103348	2.472587	1.130579
H	-8.321530	2.259514	-1.012776
H	-7.040750	2.135245	-3.155766
C	-2.956049	2.406676	-1.043738
C	-2.013022	1.830118	-0.337939
C	-4.478309	2.555197	1.606158
H	-5.058100	3.234818	2.242057
H	-3.474048	2.984191	1.514396
H	-2.509730	2.993425	-1.851665
O	-1.003104	1.446414	0.162279
H	2.921222	-0.416743	5.649821
C	-4.388369	1.190021	2.307444
H	-5.399389	0.778273	2.388693
H	-4.018615	1.322777	3.336177
N	-3.551662	0.250844	1.545689
H	-2.599810	0.327553	1.888924
C	-3.957240	-1.158371	1.680513
H	-4.167928	-1.421363	2.732646
H	-3.108487	-1.775168	1.370292
C	-5.168960	-1.515736	0.817435
H	-4.943663	-1.254932	-0.222447
H	-6.035474	-0.907891	1.107499
C	-5.521551	-3.002063	0.927168
H	-6.379553	-3.257344	0.295548
H	-4.678582	-3.630821	0.615794
H	-5.775732	-3.275440	1.958908

XVa

C	-0.645502	-0.272166	-3.158141
C	0.615081	-0.930995	-3.164255
C	1.655381	0.049751	-3.065859
C	1.015278	1.337523	-3.043828
C	-0.388839	1.144965	-3.089219
H	-1.613090	-0.749052	-3.225033
H	0.759169	-2.001156	-3.222185
H	2.719541	-0.136426	-3.080548
H	1.520943	2.292421	-2.986622
H	-1.130248	1.931964	-3.090853
Ru	0.437566	0.155997	-1.225650
P	1.726426	1.675748	0.046460
P	0.787523	-1.874122	-0.087099
C	0.951396	3.356359	0.107914
C	-0.325787	3.555949	-0.434219
C	1.589989	4.433816	0.749936

C	-0.945980	4.806690	-0.355516
H	-0.843378	2.727597	-0.900624
C	0.975049	5.683500	0.819814
H	2.565623	4.293462	1.205165
C	-0.295359	5.873450	0.265818
H	-1.938256	4.940231	-0.777579
H	1.484140	6.506685	1.312883
H	-0.775365	6.846057	0.326423
C	3.380693	1.933173	-0.750206
C	3.820578	3.169146	-1.246938
C	4.200177	0.805253	-0.935825
C	5.059622	3.276634	-1.886517
H	3.203343	4.054443	-1.150994
C	5.442612	0.917432	-1.558633
H	3.858913	-0.172051	-0.616923
C	5.878386	2.156531	-2.036368
H	5.380811	4.242167	-2.267109
H	6.059365	0.031566	-1.680944
H	6.841993	2.245473	-2.529739
C	2.139597	1.447980	1.843931
C	3.198254	0.622272	2.252418
C	1.310001	2.009968	2.832163
C	3.419471	0.362387	3.606277
H	3.858952	0.169693	1.524689
C	1.534566	1.750473	4.185185
H	0.480101	2.648802	2.550939
C	2.588362	0.922173	4.578136
H	4.244865	-0.281294	3.897127
H	0.883552	2.197728	4.931222
C	2.536682	-2.453558	-0.013762
C	3.209882	-2.601468	1.207879
C	3.253997	-2.637855	-1.210067
C	4.572731	-2.911193	1.232494
H	2.680046	-2.459747	2.143143
C	4.609819	-2.963138	-1.182350
H	2.757211	-2.523250	-2.167059
C	5.276233	-3.091658	0.040642
H	5.081037	-3.011751	2.187294
H	5.146675	-3.107919	-2.115493
H	6.335011	-3.332834	0.062019
C	-0.129884	-3.218883	-0.973893
C	-1.494328	-2.989019	-1.228256
C	0.437051	-4.438921	-1.362203
C	-2.271631	-3.959602	-1.858232
H	-1.948576	-2.046761	-0.937182
C	-0.342411	-5.406696	-2.006590
H	1.482990	-4.646141	-1.163285
C	-1.694969	-5.171421	-2.255062
H	-3.325397	-3.769157	-2.041329
H	0.112174	-6.346497	-2.307227
H	-2.298087	-5.925652	-2.752244
C	0.166611	-2.183425	1.631307
C	-0.220928	-3.470748	2.041988
C	0.059806	-1.126531	2.543264
C	-0.719563	-3.684196	3.328332
H	-0.146998	-4.310148	1.359065
C	-0.437393	-1.339480	3.830579
H	0.341244	-0.132357	2.239775
C	-0.834583	-2.618804	4.225425
H	-1.020349	-4.684376	3.627044
H	-0.510489	-0.502646	4.519149
H	-1.229017	-2.786200	5.223654

C	-5.325999	0.815756	-2.092396
C	-4.603272	1.187858	-0.941383
C	-5.292417	1.849286	0.102061
C	-6.669059	2.070822	-0.008233
C	-7.377540	1.671939	-1.143796
C	-6.695417	1.050399	-2.194134
H	-4.798392	0.325324	-2.907135
H	-7.187932	2.577668	0.802138
H	-8.445789	1.855881	-1.212934
H	-7.230816	0.746133	-3.089476
C	-3.164998	0.935688	-0.886340
C	-2.380077	0.633322	0.181445
C	-4.523790	2.321702	1.310176
H	-5.157443	2.956372	1.937687
H	-3.661154	2.926887	1.002885
H	-2.629347	0.979661	-1.824802
O	-1.110648	0.471375	0.270305
H	2.760790	0.717110	5.630664
C	-4.043477	1.181918	2.197534
H	-4.878356	0.534179	2.464972
H	-3.595294	1.565674	3.115978
N	-2.973384	0.297997	1.556852
H	-2.136928	0.408486	2.138329
C	-3.288249	-1.190599	1.664360
H	-3.484152	-1.368361	2.725113
H	-2.364088	-1.703265	1.400511
C	-4.437503	-1.681829	0.797031
H	-4.205998	-1.500371	-0.255406
H	-5.357157	-1.131729	1.021537
C	-4.648237	-3.181827	1.032808
H	-5.455842	-3.560134	0.398728
H	-3.741244	-3.748813	0.796499
H	-4.913222	-3.389417	2.075682

e. Coordinates for Ru(II)-vinylidene hydroamination. IVa to 2a.

$TS_{IVa\text{-}VIIIa}$

C	-0.809994	-0.115893	-3.437567
C	-0.941036	-1.477179	-3.016902
C	0.349662	-2.022419	-2.798160
C	1.298208	-0.987423	-3.085000
C	0.585444	0.183813	-3.479067
H	-1.610779	0.552071	-3.721429
H	-1.870774	-2.012407	-2.893648
H	0.580631	-3.031235	-2.487109
H	2.371977	-1.105346	-3.057371
H	1.022486	1.127601	-3.776918
Ru	0.084875	-0.267633	-1.303172
P	2.043269	-0.729547	-0.026754
P	-1.673833	-1.032746	0.176738
C	3.564623	0.251811	-0.474326
C	3.702771	0.924495	-1.696570
C	4.662273	0.237518	0.405080
C	4.891499	1.581471	-2.025969
H	2.880402	0.958966	-2.397332
C	5.850726	0.888806	0.075528
H	4.596730	-0.289113	1.351050
C	5.969026	1.567001	-1.140376
H	4.967708	2.104783	-2.974715
H	6.684428	0.864661	0.771330
H	6.893063	2.078349	-1.393974
C	2.680200	-2.445612	-0.332416

C	3.831296	-2.664503	-1.109536
C	1.966246	-3.561008	0.131435
C	4.254585	-3.962789	-1.403254
H	4.405385	-1.830034	-1.493794
C	2.399731	-4.857117	-0.149401
H	1.064621	-3.424428	0.707641
C	3.544752	-5.063966	-0.921205
H	5.145202	-4.108348	-2.008033
H	1.830340	-5.699580	0.230914
H	3.879113	-6.072611	-1.146271
C	2.055270	-0.474032	1.802447
C	1.981024	-1.513041	2.739252
C	2.105488	0.856231	2.264655
C	1.928243	-1.227744	4.107128
H	1.968611	-2.546940	2.421451
C	2.062783	1.135104	3.629284
H	2.172661	1.674839	1.557695
C	1.964371	0.092547	4.556280
H	1.865002	-2.045656	4.819106
H	2.099917	2.167052	3.965750
H	1.924183	0.309242	5.619770
C	-1.328401	-2.710829	0.880570
C	-1.046448	-2.941529	2.232320
C	-1.353051	-3.802581	-0.007714
C	-0.780713	-4.238597	2.685544
H	-1.030742	-2.122191	2.940802
C	-1.108696	-5.094965	0.450420
H	-1.568999	-3.641526	-1.059486
C	-0.814314	-5.316580	1.800440
H	-0.555466	-4.400470	3.735800
H	-1.136646	-5.927184	-0.246875

H	-0.615122	-6.322821	2.157214
C	-3.324198	-1.407058	-0.613608
C	-3.780875	-0.735516	-1.754000
C	-4.184218	-2.339493	-0.002288
C	-5.050013	-0.988805	-2.280782
H	-3.148375	-0.008985	-2.239494
C	-5.451677	-2.593337	-0.526055
H	-3.873150	-2.865073	0.893627
C	-5.889434	-1.920590	-1.669954
H	-5.376498	-0.454182	-3.167937
H	-6.096893	-3.317123	-0.036491
H	-6.875552	-2.120798	-2.078855
C	-2.218192	-0.047544	1.645969
C	-3.568292	0.253338	1.897968
C	-1.252238	0.382044	2.565236
C	-3.933001	0.956378	3.048047
H	-4.342523	-0.046808	1.203309
C	-1.619192	1.074242	3.720084
H	-0.208762	0.178429	2.380342
C	-2.962094	1.365090	3.965607
H	-4.980421	1.185218	3.222526
H	-0.848055	1.391829	4.414879
H	-3.250165	1.909605	4.860201
C	1.888721	4.931695	-0.996758
C	1.041340	4.053887	-0.289804
C	0.302211	4.558653	0.807002
C	0.407019	5.919155	1.126564
C	1.229703	6.781898	0.402459
C	1.979555	6.280545	-0.665017
H	2.481316	4.537548	-1.818765
H	-0.165350	6.301058	1.968696

H	1.293811	7.831418	0.675393
H	2.635815	6.934939	-1.231897
C	1.070446	2.634610	-0.691718
C	0.266847	1.573256	-0.771420
H	2.064556	2.337543	-1.029446
C	-0.605535	3.659567	1.604345
C	-1.943722	3.435010	0.894497
H	-0.797290	4.090558	2.592318
H	-0.127992	2.687173	1.752438
H	-2.535561	2.701181	1.443111
H	-2.513181	4.377044	0.879904
N	-1.717465	2.925477	-0.463593
C	-2.909271	2.431846	-1.148759
C	-3.990068	3.481238	-1.445986
H	-3.337724	1.631295	-0.540833
H	-2.572966	1.983639	-2.090011
C	-5.149749	2.879738	-2.244970
H	-4.368648	3.899403	-0.505795
H	-3.539325	4.313459	-2.003926
H	-5.925937	3.625861	-2.445156
H	-4.804586	2.490931	-3.210695
H	-5.615071	2.049684	-1.701125
H	-1.307053	3.664339	-1.030392

VIIIa

C	0.782289	-0.455578	-3.413466
C	1.674896	0.572885	-2.976452
C	0.936916	1.765229	-2.755335
C	-0.432015	1.474884	-3.068098
C	-0.532721	0.113026	-3.463341
H	1.059424	-1.444546	-3.749134
H	2.743249	0.471705	-2.854676
H	1.331455	2.720274	-2.439992
H	-1.238376	2.193328	-3.042763
H	-1.435267	-0.395943	-3.774707
Ru	0.171835	0.170745	-1.296440
P	-1.154064	1.718732	-0.037625
P	1.989326	-0.228746	0.178811
C	-2.966133	1.906628	-0.479992
C	-3.492835	1.459877	-1.700689
C	-3.814710	2.617776	0.387043
C	-4.826807	1.699397	-2.040952
H	-2.873446	0.899125	-2.387933
C	-5.146557	2.858828	0.049020
H	-3.436541	2.995500	1.330876
C	-5.659230	2.398558	-1.166398
H	-5.210709	1.333919	-2.989167
H	-5.782218	3.408974	0.737007
H	-6.697070	2.583715	-1.427922
C	-0.621718	3.475240	-0.360752
C	-1.394374	4.338669	-1.157134
C	0.616954	3.939394	0.107385
C	-0.943580	5.625514	-1.461599
H	-2.350946	4.017374	-1.550612
C	1.058550	5.231327	-0.180765
H	1.249394	3.288397	0.690689
C	0.280598	6.080917	-0.969666
H	-1.558143	6.271426	-2.082479
H	2.017663	5.561241	0.206064
H	0.625877	7.084470	-1.201268
C	-1.330021	1.586425	1.802095
C	-0.619131	2.372316	2.719088

C	-2.191244	0.586855	2.297127
C	-0.741422	2.144153	4.093782
H	0.029582	3.169906	2.383973
C	-2.317613	0.367911	3.667521
H	-2.764658	-0.024131	1.609805
C	-1.583523	1.140813	4.573054
H	-0.176584	2.760948	4.787091
H	-2.986741	-0.409078	4.025968
H	-1.675182	0.966453	5.641177
C	2.739353	1.322846	0.877271
C	2.647212	1.702838	2.222865
C	3.429060	2.162474	-0.017732
C	3.219313	2.903028	2.660193
H	2.134371	1.074659	2.940478
C	4.015043	3.346350	0.424018
H	3.512231	1.887778	-1.064689
C	3.905863	3.725331	1.766586
H	3.130618	3.185668	3.705392
H	4.547277	3.978762	-0.280651
H	4.354897	4.652465	2.110654
C	3.557574	-0.941033	-0.562651
C	3.563126	-1.708660	-1.733916
C	4.780326	-0.757420	0.111600
C	4.745092	-2.271003	-2.225567
H	2.647041	-1.868033	-2.281115
C	5.960908	-1.317419	-0.375330
H	4.813488	-0.180571	1.029473
C	5.948866	-2.076648	-1.548809
H	4.717401	-2.858069	-3.138987
H	6.890341	-1.160085	0.164547
H	6.868780	-2.510546	-1.929976

C	1.833652	-1.315607	1.678100
C	2.659206	-2.430416	1.907929
C	0.818353	-1.036794	2.605515
C	2.470904	-3.234325	3.035233
H	3.444088	-2.690417	1.208716
C	0.641429	-1.829757	3.739965
H	0.152196	-0.204625	2.436838
C	1.465348	-2.936021	3.957449
H	3.113994	-4.096309	3.188666
H	-0.151935	-1.585059	4.439244
H	1.322982	-3.561762	4.833769
C	-4.582243	-2.617286	-0.935289
C	-3.360701	-2.525543	-0.243777
C	-3.117069	-3.405991	0.836378
C	-4.069161	-4.377263	1.163700
C	-5.264150	-4.477841	0.448609
C	-5.523560	-3.588524	-0.599369
H	-4.785247	-1.916894	-1.741804
H	-3.875207	-5.050762	1.994988
H	-5.994118	-5.236835	0.715192
H	-6.458028	-3.651135	-1.149983
C	-2.396922	-1.457682	-0.588343
C	-1.045244	-1.495185	-0.691882
H	-2.870185	-0.506415	-0.768606
C	-1.843409	-3.230894	1.625013
C	-0.600238	-3.600511	0.824378
H	-1.847904	-3.857397	2.521908
H	-1.754581	-2.191332	1.954578
H	0.307251	-3.273033	1.320910
H	-0.557203	-4.681690	0.679810
N	-0.603648	-3.001553	-0.569011

C	0.654725	-3.351450	-1.299816
C	0.816863	-4.845103	-1.576654
H	1.484187	-2.973156	-0.707886
H	0.616338	-2.800265	-2.235413
C	2.024722	-5.080339	-2.490123
H	0.956878	-5.400025	-0.643370
H	-0.095202	-5.231623	-2.051849
H	2.159618	-6.148386	-2.687346
H	1.898766	-4.573309	-3.454164
H	2.944704	-4.702711	-2.030341
H	-1.347594	-3.504814	-1.064445

VIIIa-HCO_3^-

C	0.035927	-1.109933	-3.123170
C	1.407353	-0.724246	-2.977639
C	1.488317	0.691206	-2.950581
C	0.148576	1.190251	-3.086166
C	-0.745480	0.089904	-3.188600
H	-0.345691	-2.110484	-3.256792
H	2.250552	-1.397126	-2.918131
H	2.386883	1.285613	-2.866153
H	-0.117543	2.235514	-3.143622
H	-1.822525	0.134381	-3.301883
Ru	0.259081	0.003040	-1.139097
P	0.313873	2.156943	-0.095926
P	1.717782	-1.237329	0.237005
C	-1.093241	3.352486	-0.435777
C	-2.011279	3.163119	-1.479101
C	-1.180824	4.536096	0.318264
C	-2.995604	4.116495	-1.753290
H	-1.986891	2.257141	-2.070185
C	-2.161278	5.490132	0.044971
H	-0.475993	4.721752	1.122006
C	-3.074932	5.282899	-0.991754
H	-3.701368	3.937431	-2.559348
H	-2.209300	6.395532	0.643499
H	-3.841791	6.023064	-1.201572
C	1.700964	3.199335	-0.782094
C	1.445252	4.225884	-1.709179
C	3.039407	2.908614	-0.475929
C	2.492861	4.938498	-2.297597
H	0.428694	4.478312	-1.984820
C	4.085482	3.633090	-1.049048
H	3.276101	2.103411	0.201299
C	3.817827	4.651137	-1.965954
H	2.266101	5.723298	-3.013944
H	5.108394	3.385812	-0.782073
H	4.631252	5.210527	-2.419246
C	0.395456	2.357614	1.748612
C	1.568308	2.641516	2.461987
C	-0.793791	2.141306	2.473923
C	1.561509	2.676597	3.860122
H	2.497192	2.839606	1.945268
C	-0.799078	2.183060	3.867089
H	-1.719678	1.934664	1.951563
C	0.383119	2.441172	4.568192
H	2.484596	2.892624	4.390839
H	-1.728129	2.009695	4.402715
H	0.381531	2.466808	5.654115
C	3.325692	-0.378267	0.606880
C	3.696938	0.082713	1.875749
C	4.212747	-0.185473	-0.468935

C	4.920889	0.735742	2.062624
H	3.042457	-0.059885	2.726818
C	5.440588	0.443451	-0.277000
H	3.942399	-0.532149	-1.461447
C	5.797018	0.914201	0.991771
H	5.187681	1.095168	3.052624
H	6.114256	0.577214	-1.118506
H	6.749939	1.413482	1.141661
C	2.465609	-2.806132	-0.474202
C	1.852881	-3.543703	-1.494348
C	3.663821	-3.306512	0.071692
C	2.416563	-4.732389	-1.968048
H	0.930501	-3.196183	-1.931887
C	4.227393	-4.493252	-0.395666
H	4.160133	-2.772463	0.874709
C	3.607059	-5.211121	-1.422116
H	1.917803	-5.278604	-2.763464
H	5.151978	-4.856355	0.044258
H	4.047789	-6.133373	-1.789764
C	1.200512	-1.869903	1.907874
C	1.261330	-3.227359	2.268724
C	0.687980	-0.955755	2.840405
C	0.827265	-3.649362	3.527858
H	1.631139	-3.967861	1.570219
C	0.273896	-1.374353	4.105745
H	0.602907	0.087869	2.576737
C	0.337757	-2.725083	4.453629
H	0.872896	-4.704775	3.781492
H	-0.113342	-0.641272	4.806641
H	0.003974	-3.055518	5.433109
C	-4.993114	0.869144	0.194161

C	-3.891349	0.177265	0.727072
C	-4.065429	-0.579368	1.904946
C	-5.332272	-0.670623	2.490883
C	-6.426612	-0.005927	1.933569
C	-6.251264	0.773060	0.784668
H	-4.854393	1.468602	-0.701527
H	-5.456850	-1.257183	3.398382
H	-7.406003	-0.085843	2.397338
H	-7.095260	1.302219	0.350254
C	-2.558003	0.340495	0.116692
C	-1.555172	-0.542984	-0.109715
H	-2.379558	1.353530	-0.199209
C	-2.846347	-1.221398	2.511049
C	-2.276272	-2.345073	1.656011
H	-3.074082	-1.650726	3.492126
H	-2.071319	-0.463317	2.661585
H	-1.324606	-2.688268	2.057662
H	-2.974947	-3.184152	1.646417
N	-2.061443	-1.978837	0.203537
C	-1.313409	-3.078722	-0.482703
C	-2.042122	-4.423518	-0.462418
H	-0.335522	-3.157120	-0.014905
H	-1.200282	-2.754341	-1.510560
C	-1.376689	-5.396274	-1.440987
H	-2.035924	-4.856806	0.543591
H	-3.083105	-4.256548	-0.756325
H	-1.874990	-6.371199	-1.421535
H	-1.434016	-5.009301	-2.464286
H	-0.320856	-5.554174	-1.194725
H	-2.995501	-2.001560	-0.290768
C	-3.753963	-1.711742	-2.619297

O	-4.145160	-2.136505	-1.498887
O	-2.876109	-2.598315	-3.280054
H	-2.672867	-2.167750	-4.124803
O	-4.022698	-0.654318	-3.219712

$TS_{VIIIa\text{-}XIa}$

C	-0.083578	-1.069444	-3.173155
C	1.328928	-0.879938	-3.027932
C	1.609298	0.510127	-2.992684
C	0.353612	1.192948	-3.114862
C	-0.685820	0.229127	-3.223959
H	-0.600656	-2.007116	-3.310276
H	2.070012	-1.664329	-2.976554
H	2.582056	0.972126	-2.902360
H	0.235017	2.265350	-3.159508
H	-1.742793	0.429267	-3.330082
Ru	0.287806	-0.017259	-1.176649
P	0.600568	2.106064	-0.116200
P	1.571921	-1.428027	0.207900
C	-0.624741	3.487574	-0.468832
C	-1.546277	3.431569	-1.524334
C	-0.549079	4.674330	0.281255
C	-2.373676	4.518975	-1.817554
H	-1.644085	2.529566	-2.113356
C	-1.372756	5.761952	-0.010134
H	0.163035	4.758058	1.095619
C	-2.290195	5.688416	-1.061368
H	-3.084925	4.443823	-2.635276
H	-1.295221	6.667360	0.585148
H	-2.933625	6.533973	-1.287062
C	2.119990	2.962694	-0.785073
C	2.006257	3.989534	-1.740705
C	3.409279	2.529920	-0.438283
C	3.141315	4.564070	-2.317193
H	1.032017	4.350959	-2.047207
C	4.544644	3.118197	-0.998251
H	3.537802	1.720033	0.262274
C	4.417160	4.137175	-1.943698
H	3.021924	5.351712	-3.056358
H	5.526181	2.763951	-0.697589
H	5.299802	4.590227	-2.386253
C	0.682370	2.297684	1.730198
C	1.869080	2.423582	2.465443
C	-0.537297	2.241273	2.434488
C	1.841456	2.460169	3.863337
H	2.826213	2.492262	1.967429
C	-0.562614	2.283585	3.827374
H	-1.472369	2.156860	1.895075
C	0.629980	2.383940	4.550143
H	2.775335	2.551096	4.410726
H	-1.516075	2.233756	4.345416
H	0.612182	2.409713	5.635928
C	3.267423	-0.762590	0.591179
C	3.683355	-0.356626	1.864822
C	4.178754	-0.673606	-0.477840
C	4.976943	0.140441	2.063642
H	3.010042	-0.422538	2.710742
C	5.472817	-0.200684	-0.274637
H	3.874448	-0.981628	-1.473525
C	5.875695	0.215918	0.999286
H	5.278787	0.459406	3.057488
H	6.163335	-0.144974	-1.111330

H	6.881604	0.593881	1.157819
C	2.147294	-3.069343	-0.504091
C	1.467430	-3.727634	-1.535838
C	3.279448	-3.701649	0.045963
C	1.906403	-4.964016	-2.019515
H	0.587391	-3.282604	-1.973047
C	3.717304	-4.937163	-0.429712
H	3.822061	-3.232074	0.859177
C	3.034304	-5.572887	-1.470388
H	1.358421	-5.447009	-2.823451
H	4.593004	-5.402069	0.014477
H	3.377641	-6.532815	-1.845486
C	0.980914	-1.995525	1.878038
C	0.912599	-3.347961	2.254778
C	0.552075	-1.026624	2.796313
C	0.436981	-3.712248	3.516511
H	1.213841	-4.128076	1.566391
C	0.096010	-1.388899	4.064719
H	0.564360	0.016576	2.518086
C	0.032803	-2.735007	4.429132
H	0.382948	-4.764156	3.782989
H	-0.224671	-0.614297	4.754453
H	-0.333466	-3.020943	5.411056
C	-4.782131	1.563683	0.143570
C	-3.792496	0.710742	0.664271
C	-4.074430	-0.017716	1.839641
C	-5.339481	0.073863	2.427921
C	-6.328126	0.894199	1.878088
C	-6.041201	1.649308	0.736149
H	-4.556249	2.153149	-0.742312
H	-5.546886	-0.493574	3.332859
H	-7.308297	0.954019	2.343594
H	-6.798113	2.302479	0.309472
C	-2.456709	0.660267	0.044423
C	-1.605391	-0.381761	-0.166746
H	-2.124022	1.630408	-0.278875
C	-2.959270	-0.829675	2.440201
C	-2.544870	-2.021643	1.584687
H	-3.246731	-1.221300	3.422113
H	-2.087927	-0.184886	2.594553
H	-1.681898	-2.515242	2.041085
H	-3.370162	-2.739787	1.569977
N	-2.238177	-1.706167	0.148092
C	-1.596072	-2.887342	-0.475591
C	-2.433175	-4.172413	-0.423034
H	-0.631560	-3.075007	-0.001101
H	-1.423512	-2.626115	-1.514510
C	-1.808481	-5.260183	-1.302401
H	-2.504144	-4.544020	0.605002
H	-3.453547	-3.962573	-0.759296
H	-2.397568	-6.182695	-1.263682
H	-1.757594	-4.940739	-2.350332
H	-0.790037	-5.498726	-0.977950
H	-3.458438	-1.682332	-0.506627
C	-4.555154	-1.604254	-2.262934
O	-4.497998	-1.730291	-0.973597
O	-3.321265	-1.694098	-2.866119
H	-3.473300	-1.570966	-3.817151
O	-5.566802	-1.438862	-2.929906

XIa-H_2CO_3

C	-0.119882	-1.091382	-3.159493

C	1.296938	-0.927228	-3.024193
C	1.603854	0.457428	-3.003273
C	0.360502	1.162734	-3.121104
C	-0.698098	0.217822	-3.216981
H	-0.654701	-2.020533	-3.286335
H	2.023418	-1.725039	-2.971184
H	2.585784	0.901249	-2.922211
H	0.262097	2.237078	-3.172617
H	-1.751457	0.437843	-3.320856
Ru	0.282134	-0.031068	-1.173265
P	0.627386	2.100493	-0.135237
P	1.549834	-1.454398	0.215462
C	-0.589294	3.491033	-0.483628
C	-1.511212	3.439541	-1.539322
C	-0.510127	4.676523	0.267992
C	-2.335424	4.529605	-1.830864
H	-1.612847	2.537802	-2.128146
C	-1.331368	5.766876	-0.021342
H	0.202218	4.757572	1.082274
C	-2.249167	5.697636	-1.072321
H	-3.047332	4.457534	-2.648320
H	-1.251685	6.670873	0.575967
H	-2.891030	6.544981	-1.295922
C	2.148023	2.932864	-0.831237
C	2.041452	3.981862	-1.762346
C	3.431937	2.456678	-0.524088
C	3.179316	4.536468	-2.353502
H	1.071350	4.375979	-2.039047
C	4.570334	3.023534	-1.099212
H	3.552274	1.629206	0.156930
C	4.450228	4.065753	-2.020121

H	3.065759	5.342555	-3.073219
H	5.547771	2.635082	-0.830071
H	5.334860	4.502727	-2.474694
C	0.746290	2.315372	1.706285
C	1.949007	2.464815	2.410095
C	-0.456598	2.263825	2.439230
C	1.954410	2.527532	3.807383
H	2.892263	2.537541	1.886365
C	-0.449259	2.334309	3.831187
H	-1.402605	2.159544	1.922010
C	0.759668	2.456524	4.523556
H	2.900152	2.637132	4.330864
H	-1.389763	2.288952	4.372916
H	0.767241	2.504190	5.608728
C	3.262187	-0.818171	0.577468
C	3.690898	-0.389654	1.839658
C	4.170437	-0.768772	-0.496949
C	4.992945	0.091524	2.021239
H	3.020548	-0.424355	2.689798
C	5.472546	-0.311010	-0.310479
H	3.857291	-1.094360	-1.484283
C	5.887707	0.128958	0.951593
H	5.304249	0.428882	3.006041
H	6.159994	-0.286011	-1.151216
H	6.899922	0.495229	1.096886
C	2.087834	-3.114833	-0.481386
C	1.393544	-3.766730	-1.507452
C	3.205572	-3.767460	0.074390
C	1.803721	-5.017491	-1.979181
H	0.524240	-3.304946	-1.948576
C	3.614436	-5.017584	-0.388833

H	3.758948	-3.302341	0.882979
C	2.916704	-5.647410	-1.423314
H	1.244963	-5.495320	-2.778795
H	4.478849	-5.498577	0.060322
H	3.237269	-6.618980	-1.788507
C	0.963795	-1.988774	1.898387
C	0.878973	-3.333118	2.299560
C	0.558316	-0.997979	2.803408
C	0.410932	-3.667896	3.572259
H	1.161128	-4.129815	1.622224
C	0.108662	-1.330593	4.082119
H	0.584452	0.039927	2.506888
C	0.029549	-2.668941	4.471127
H	0.344221	-4.714016	3.858065
H	-0.193890	-0.538931	4.760670
H	-0.331225	-2.932064	5.461457
C	-4.742720	1.648359	0.176312
C	-3.767717	0.777027	0.695709
C	-4.064243	0.053114	1.870539
C	-5.328586	0.164986	2.456954
C	-6.302807	1.002371	1.907080
C	-6.000667	1.755277	0.767346
H	-4.505648	2.235459	-0.708338
H	-5.546620	-0.399931	3.361045
H	-7.282773	1.077493	2.370746
H	-6.745432	2.422439	0.340754
C	-2.435766	0.698791	0.072680
C	-1.609826	-0.366248	-0.140560
H	-2.087117	1.659402	-0.260564
C	-2.962308	-0.773863	2.473979
C	-2.566900	-1.973760	1.620713
H	-3.255803	-1.157248	3.457506
H	-2.081277	-0.141497	2.625835
H	-1.718829	-2.485596	2.089477
H	-3.407304	-2.675031	1.607752
N	-2.243343	-1.669554	0.190194
C	-1.635990	-2.865411	-0.427408
C	-2.514341	-4.124208	-0.388131
H	-0.680896	-3.095094	0.052742
H	-1.442717	-2.612959	-1.464625
C	-1.921895	-5.227102	-1.271054
H	-2.604059	-4.499600	0.637124
H	-3.526895	-3.882408	-0.727594
H	-2.537414	-6.132447	-1.236742
H	-1.860570	-4.905107	-2.317698
H	-0.911005	-5.496470	-0.946834
H	-3.591966	-1.611293	-0.555626
C	-4.583216	-1.599103	-2.299262
O	-4.561396	-1.627950	-0.992452
O	-3.338447	-1.710517	-2.854365
H	-3.460127	-1.663322	-3.817100
O	-5.587272	-1.498713	-2.982380

$TS_{VIIIa\text{-}8a}$

C	0.935874	-0.813415	-3.268432
C	1.856138	0.220849	-2.945130
C	1.151185	1.460977	-2.849983
C	-0.221741	1.184588	-3.166751
C	-0.362393	-0.207273	-3.404258
H	1.155314	-1.860544	-3.411841
H	2.919040	0.097178	-2.800880
H	1.581578	2.431163	-2.651773

H	-1.011457	1.921061	-3.221170
H	-1.282695	-0.720182	-3.642607
Ru	0.338311	0.097365	-1.285589
P	-1.076889	1.716035	-0.209354
P	2.172273	-0.067581	0.267526
C	-2.898688	1.662946	-0.615831
C	-3.354060	1.028928	-1.780937
C	-3.837262	2.343951	0.178606
C	-4.703219	1.064115	-2.139365
H	-2.669284	0.467373	-2.400400
C	-5.187686	2.375432	-0.175049
H	-3.519703	2.862242	1.076467
C	-5.626654	1.736192	-1.336341
H	-5.029171	0.549518	-3.038674
H	-5.894387	2.903332	0.459233
H	-6.677881	1.757781	-1.609462
C	-0.686307	3.435931	-0.827919
C	-1.626195	4.229616	-1.505511
C	0.612027	3.944652	-0.661642
C	-1.276591	5.493237	-1.991629
H	-2.635819	3.872063	-1.666232
C	0.956994	5.212034	-1.131789
H	1.363231	3.343867	-0.170818
C	0.012699	5.992561	-1.803148
H	-2.020474	6.085565	-2.517431
H	1.967940	5.577987	-0.978742
H	0.280427	6.976346	-2.178280
C	-1.159571	1.961618	1.628381
C	-0.445164	2.960817	2.305508
C	-1.935316	1.063236	2.382661
C	-0.485286	3.040624	3.700851
H	0.149753	3.681962	1.760142
C	-1.983542	1.151100	3.772797
H	-2.509415	0.295142	1.882230
C	-1.250119	2.136645	4.439921
H	0.081825	3.817413	4.206019
H	-2.589949	0.442399	4.329495
H	-1.279864	2.202525	5.523781
C	2.938012	1.601491	0.605189
C	2.831763	2.283192	1.824499
C	3.622919	2.231059	-0.452489
C	3.366121	3.567681	1.974228
H	2.332277	1.828573	2.669462
C	4.165371	3.505399	-0.299452
H	3.742039	1.723149	-1.402656
C	4.030358	4.185740	0.915501
H	3.261223	4.078707	2.927290
H	4.689640	3.968674	-1.130523
H	4.445281	5.182456	1.034352
C	3.714860	-0.923437	-0.365629
C	3.678559	-1.900730	-1.367851
C	4.958874	-0.627958	0.223994
C	4.839750	-2.564589	-1.773533
H	2.742797	-2.149797	-1.846352
C	6.120146	-1.286892	-0.178998
H	5.022906	0.114235	1.012745
C	6.065302	-2.259784	-1.181131
H	4.778635	-3.318271	-2.553537
H	7.067172	-1.041120	0.293469
H	6.969424	-2.773529	-1.495576
C	2.086162	-0.817526	1.971184
C	2.906233	-1.886821	2.371581

C	1.193966	-0.282708	2.911553
C	2.836242	-2.394424	3.670980
H	3.607014	-2.334319	1.678373
C	1.143630	-0.770891	4.218622
H	0.527668	0.517123	2.626146
C	1.962230	-1.832872	4.604140
H	3.475086	-3.227159	3.951932
H	0.445776	-0.327187	4.921850
H	1.917346	-2.221497	5.617540
C	-4.558766	-1.615495	0.635720
C	-3.204103	-1.865636	0.961199
C	-2.911585	-2.333749	2.267341
C	-3.955060	-2.581265	3.164715
C	-5.289233	-2.349236	2.817715
C	-5.585836	-1.853680	1.543710
H	-4.791405	-1.221620	-0.349997
H	-3.713498	-2.948365	4.160188
H	-6.082513	-2.540999	3.535035
H	-6.615643	-1.651805	1.259832
C	-2.194842	-1.540447	-0.039546
C	-0.870654	-1.876634	-0.244101
H	-2.609713	-0.986000	-0.864464
C	-1.479545	-2.504686	2.681983
C	-0.649399	-3.402340	1.750921
H	-1.434777	-2.922790	3.693094
H	-0.996541	-1.525505	2.727124
H	0.223480	-3.772382	2.302509
H	-1.251281	-4.282534	1.478354
N	-0.129665	-2.731850	0.560220
C	0.977131	-3.454140	-0.059453
C	0.684892	-4.907540	-0.466606
H	1.840831	-3.446196	0.617210
H	1.251017	-2.895579	-0.950137
C	1.844038	-5.479993	-1.286721
H	0.525501	-5.526678	0.424240
H	-0.236126	-4.931302	-1.056376
H	1.678486	-6.533642	-1.536851
H	1.965588	-4.930153	-2.227356
H	2.792186	-5.412461	-0.739413
H	-0.811138	-2.307990	-1.601330
C	-1.998104	-3.098910	-3.143912
O	-0.856661	-3.085274	-2.534356
O	-2.146866	-4.236042	-3.885680
H	-3.008164	-4.156367	-4.328328
O	-2.879683	-2.237315	-3.128374

XIIa-HCO_3^-

C	2.482070	-0.696797	-2.977224
C	3.426279	-0.014424	-2.168702
C	3.063091	1.377812	-2.118247
C	1.923041	1.556653	-2.989972
C	1.550447	0.282489	-3.478600
H	2.419410	-1.758023	-3.162431
H	4.257421	-0.469099	-1.647692
H	3.606068	2.162364	-1.610182
H	1.419399	2.492681	-3.190244
H	0.678164	0.044429	-4.071209
Ru	1.443897	0.262163	-1.250892
P	0.146804	2.076622	-0.370943
P	2.160891	-0.911923	0.691481
C	-1.311794	2.456652	-1.438807
C	-1.608537	1.625360	-2.526793

C	-2.156937	3.547134	-1.160480
C	-2.709278	1.899466	-3.345417
H	-1.018717	0.736238	-2.718513
C	-3.255141	3.815453	-1.975932
H	-1.960193	4.182578	-0.302324
C	-3.529353	2.995808	-3.076828
H	-2.932143	1.237161	-4.176872
H	-3.898970	4.660836	-1.750753
H	-4.386636	3.205973	-3.710240
C	1.185231	3.610853	-0.421027
C	0.881401	4.720914	-1.222502
C	2.377404	3.625184	0.324075
C	1.737410	5.826414	-1.255349
H	-0.015904	4.733941	-1.829877
C	3.222036	4.734157	0.301898
H	2.662858	2.757162	0.905157
C	2.903864	5.841717	-0.489285
H	1.486035	6.676226	-1.883803
H	4.133372	4.722734	0.893143
H	3.563763	6.704183	-0.514765
C	-0.637829	2.139247	1.312083
C	0.075065	2.538524	2.453569
C	-1.960017	1.683637	1.469277
C	-0.515754	2.480502	3.717754
H	1.093199	2.896439	2.374538
C	-2.552139	1.641804	2.732912
H	-2.533299	1.346069	0.612763
C	-1.830649	2.034283	3.863055
H	0.056519	2.789912	4.587860
H	-3.579059	1.301613	2.826055
H	-2.290088	1.995506	4.846596

C	3.107333	0.180999	1.845881
C	2.697410	0.445689	3.159351
C	4.251871	0.833190	1.349460
C	3.406015	1.353601	3.952939
H	1.821395	-0.042190	3.570321
C	4.967092	1.723762	2.148617
H	4.585229	0.647177	0.334504
C	4.539212	1.995292	3.452470
H	3.067065	1.554213	4.965366
H	5.850980	2.212917	1.749274
H	5.087954	2.698927	4.071734
C	3.365682	-2.243286	0.250919
C	2.987549	-3.146377	-0.758436
C	4.590986	-2.422337	0.908027
C	3.833585	-4.199393	-1.108757
H	2.037298	-3.031694	-1.273395
C	5.439062	-3.472721	0.543792
H	4.891475	-1.751379	1.705291
C	5.064400	-4.361671	-0.465330
H	3.528915	-4.890605	-1.889817
H	6.389408	-3.596211	1.055729
H	5.724415	-5.177739	-0.746005
C	1.007408	-1.838058	1.804829
C	1.271759	-3.154653	2.220258
C	-0.128459	-1.187201	2.308683
C	0.422620	-3.796004	3.123975
H	2.139826	-3.685284	1.848053
C	-0.955693	-1.818473	3.238803
H	-0.357173	-0.177933	1.997468
C	-0.687802	-3.127208	3.644803
H	0.638683	-4.816277	3.427994

H	-1.806409	-1.281774	3.644369
H	-1.338129	-3.621215	4.361051
C	-6.125752	0.886910	-0.334754
C	-5.180138	-0.060249	0.127149
C	-5.385713	-0.619272	1.417947
C	-6.525724	-0.263460	2.149196
C	-7.456134	0.660001	1.667394
C	-7.241417	1.244551	0.415340
H	-5.966222	1.343945	-1.308848
H	-6.675236	-0.712346	3.129202
H	-8.325939	0.924103	2.262325
H	-7.944562	1.974980	0.022895
C	-4.048573	-0.363576	-0.737759
C	-3.084736	-1.334230	-0.693142
H	-3.942601	0.307762	-1.583823
C	-4.374364	-1.553137	2.029739
C	-3.932012	-2.735420	1.141060
H	-4.778428	-1.957201	2.964228
H	-3.471920	-0.993136	2.291968
H	-3.506195	-3.516309	1.776924
H	-4.809824	-3.165417	0.638617
N	-2.908330	-2.370409	0.173187
C	-1.831512	-3.334414	-0.064396
C	-2.268592	-4.575930	-0.853195
H	-1.427930	-3.635815	0.909163
H	-1.024519	-2.833998	-0.605424
C	-1.110255	-5.561123	-1.029244
H	-3.108024	-5.059695	-0.336429
H	-2.630930	-4.256106	-1.837589
H	-1.430923	-6.461223	-1.565733
H	-0.306497	-5.086642	-1.600367
H	-0.708609	-5.877023	-0.057740
H	-2.328434	-1.298291	-1.475649
C	-0.579946	-2.597623	-3.274327
O	0.402558	-3.048200	-2.647305
O	-1.235967	-3.540477	-4.074123
H	-1.978028	-3.059354	-4.472134
O	-1.068634	-1.435954	-3.290616

XIIa

C	-1.037116	-0.768057	-3.342534
C	-0.777135	-2.036702	-2.764244
C	0.642443	-2.188024	-2.609818
C	1.258536	-1.017434	-3.180623
C	0.230972	-0.136411	-3.599930
H	-2.007047	-0.361509	-3.591420
H	-1.523841	-2.763279	-2.479482
H	1.157352	-3.061288	-2.235534
H	2.323294	-0.844583	-3.254480
H	0.365889	0.841825	-4.044420
Ru	0.049874	-0.508540	-1.374351
P	2.133802	-0.099914	-0.254490
P	-1.272932	-1.459049	0.385412
C	3.127847	1.324445	-0.917177
C	2.926493	1.791497	-2.222747
C	4.168892	1.889454	-0.158808
C	3.735966	2.794827	-2.761119
H	2.123770	1.388936	-2.821591
C	4.968341	2.901922	-0.689754
H	4.362859	1.541032	0.849331
C	4.756075	3.357977	-1.993551
H	3.556372	3.142961	-3.774235

H	5.760978	3.330759	-0.083467
H	5.380748	4.145293	-2.405547
C	3.298427	-1.516145	-0.531080
C	4.529614	-1.360730	-1.186606
C	2.913769	-2.801513	-0.115554
C	5.355256	-2.465974	-1.412781
H	4.854254	-0.384452	-1.527134
C	3.745497	-3.900470	-0.329200
H	1.959602	-2.947109	0.371679
C	4.969738	-3.736706	-0.982933
H	6.303134	-2.327082	-1.925142
H	3.427554	-4.882122	0.009556
H	5.615566	-4.592321	-1.158055
C	2.240902	0.225313	1.563214
C	2.614531	-0.753231	2.495344
C	1.884056	1.504800	2.027224
C	2.616524	-0.461520	3.863013
H	2.904058	-1.745236	2.171219
C	1.903250	1.796423	3.389801
H	1.596654	2.279751	1.327062
C	2.263490	0.811527	4.314460
H	2.901396	-1.233571	4.572045
H	1.624594	2.791342	3.723428
H	2.270373	1.035392	5.377303
C	-0.476165	-2.970904	1.110855
C	0.014899	-3.063328	2.419610
C	-0.305294	-4.073579	0.250576
C	0.688018	-4.213091	2.846476
H	-0.119516	-2.252088	3.121774
C	0.352100	-5.223509	0.682491
H	-0.687418	-4.039217	-0.763290

C	0.864376	-5.292887	1.982119
H	1.068872	-4.258560	3.862782
H	0.470277	-6.061246	0.001354
H	1.387760	-6.183556	2.317147
C	-2.887744	-2.175469	-0.188992
C	-3.600350	-1.557449	-1.224799
C	-3.476985	-3.259963	0.480715
C	-4.866815	-2.013542	-1.596506
H	-3.164958	-0.710417	-1.736432
C	-4.739059	-3.723363	0.104417
H	-2.957610	-3.744423	1.300714
C	-5.437215	-3.102675	-0.935093
H	-5.403345	-1.516248	-2.399773
H	-5.178495	-4.566676	0.629217
H	-6.419978	-3.464319	-1.224143
C	-1.887923	-0.467325	1.829334
C	-3.251012	-0.140233	1.952358
C	-0.999656	0.004970	2.808741
C	-3.707480	0.613371	3.035535
H	-3.967812	-0.472845	1.212104
C	-1.461531	0.739656	3.901914
H	0.056851	-0.196476	2.725136
C	-2.817645	1.045700	4.021071
H	-4.764190	0.854664	3.108148
H	-0.750490	1.082155	4.646830
H	-3.176908	1.623672	4.867607
C	1.258353	4.281764	-0.503183
C	0.031199	3.637138	-0.229173
C	-0.596781	3.904921	1.013097
C	0.004051	4.793540	1.912171
C	1.224562	5.410835	1.630221

C	1.852550	5.147980	0.409417
H	1.753467	4.083347	-1.446991
H	-0.491798	4.989524	2.860314
H	1.676773	6.084544	2.352490
H	2.803184	5.615026	0.167130
C	-0.501790	2.710208	-1.226666
C	-1.789214	2.260262	-1.438582
H	0.184273	2.452651	-2.027489
C	-1.877546	3.213888	1.386340
C	-3.030628	3.394269	0.383053
H	-2.215958	3.577954	2.360108
H	-1.700045	2.141596	1.497034
H	-3.974473	3.166183	0.885910
H	-3.081133	4.439408	0.052438
N	-2.942449	2.499094	-0.769116
C	-4.194233	1.903708	-1.238702
C	-5.203100	2.911598	-1.799185
H	-4.648672	1.343488	-0.409568
H	-3.948961	1.176579	-2.016218
C	-6.469166	2.204488	-2.290604
H	-5.462946	3.645684	-1.027338
H	-4.730955	3.466223	-2.620022
H	-7.200581	2.920601	-2.678554
H	-6.238236	1.494132	-3.093759
H	-6.948310	1.643089	-1.479526
H	-1.931471	1.639880	-2.316546

TS$_{VIIIa\text{-}XIIIa}$

C	0.968916	-0.236922	-3.437238
C	1.699757	0.892921	-2.955595
C	0.798518	1.963728	-2.724613
C	-0.508334	1.494548	-3.067044
C	-0.411196	0.143869	-3.507660
H	1.386593	-1.177260	-3.768457
H	2.768790	0.935717	-2.809704
H	1.047425	2.950929	-2.364379
H	-1.405980	2.095494	-3.035443
H	-1.224306	-0.474588	-3.863629
Ru	0.213999	0.192541	-1.308795
P	-1.484818	1.388332	-0.063576
P	2.073511	0.084256	0.205064
C	-3.267624	1.125658	-0.559196
C	-3.614043	0.766125	-1.870090
C	-4.310314	1.421333	0.337399
C	-4.948835	0.664727	-2.266185
H	-2.844939	0.553111	-2.597325
C	-5.646086	1.318787	-0.054998
H	-4.090750	1.744056	1.348028
C	-5.972201	0.932663	-1.356155
H	-5.183228	0.373402	-3.285971
H	-6.430675	1.546533	0.660709
H	-7.011759	0.848769	-1.659223
C	-1.355728	3.224369	-0.332799
C	-2.409990	3.981950	-0.869012
C	-0.149331	3.876039	-0.034120
C	-2.255857	5.353005	-1.092913
H	-3.354768	3.516288	-1.120395
C	-0.001726	5.247166	-0.243567
H	0.683498	3.309707	0.354576
C	-1.055449	5.991778	-0.778593
H	-3.081942	5.919276	-1.513750
H	0.941956	5.723207	0.004913

H	-0.940907	7.057999	-0.951806
C	-1.619554	1.207004	1.770088
C	-1.037278	2.112456	2.668290
C	-2.283965	0.077219	2.281213
C	-1.095805	1.877836	4.044907
H	-0.532309	2.999905	2.308597
C	-2.346990	-0.150406	3.654939
H	-2.769962	-0.618323	1.605619
C	-1.744695	0.746453	4.542434
H	-0.633866	2.586512	4.726248
H	-2.863951	-1.029848	4.027743
H	-1.787583	0.567419	5.612774
C	2.507436	1.752761	0.899982
C	2.290409	2.123153	2.232847
C	3.056614	2.701095	0.015776
C	2.590357	3.419162	2.666379
H	1.887081	1.414600	2.944864
C	3.368915	3.986423	0.453404
H	3.243935	2.436049	-1.019480
C	3.127030	4.353967	1.781567
H	2.406379	3.689627	3.702294
H	3.794141	4.702670	-0.243665
H	3.361216	5.358467	2.121630
C	3.745169	-0.335712	-0.520434
C	3.900756	-1.091287	-1.688917
C	4.904443	0.057834	0.175200
C	5.170015	-1.443121	-2.156732
H	3.033973	-1.409424	-2.246954
C	6.171764	-0.292338	-0.289132
H	4.821097	0.631776	1.091846
C	6.310045	-1.044827	-1.458769

H	5.260102	-2.027868	-3.067622
H	7.050848	0.021820	0.266206
H	7.297337	-1.316516	-1.821019
C	2.079921	-1.022825	1.692095
C	3.072201	-1.996421	1.903735
C	1.047664	-0.906260	2.634195
C	3.031030	-2.819119	3.031341
H	3.875758	-2.130087	1.190325
C	1.015752	-1.718624	3.768583
H	0.259377	-0.184188	2.482770
C	2.006750	-2.680638	3.970733
H	3.803522	-3.569803	3.171719
H	0.206080	-1.600558	4.481809
H	1.979445	-3.319497	4.848752
C	-4.129645	-3.077328	-0.997262
C	-2.867491	-2.844981	-0.419521
C	-2.565935	-3.431867	0.831066
C	-3.526539	-4.242494	1.449113
C	-4.770050	-4.477882	0.861777
C	-5.073482	-3.882875	-0.366356
H	-4.369229	-2.603281	-1.945388
H	-3.291265	-4.688163	2.412577
H	-5.498310	-5.109960	1.361685
H	-6.043114	-4.042954	-0.829524
C	-1.932438	-1.945829	-1.155663
C	-0.589669	-1.574367	-0.829290
H	-2.462971	-1.241446	-1.779562
C	-1.257420	-3.135339	1.516370
C	-0.025846	-3.591408	0.749096
H	-1.234621	-3.597657	2.507446
H	-1.169531	-2.056570	1.678750

H	0.879352	-3.359668	1.317957
H	-0.061388	-4.674647	0.601631
N	0.045679	-2.973419	-0.598888
C	1.335390	-3.208529	-1.261465
C	1.582282	-4.679703	-1.603372
H	2.151621	-2.837798	-0.631759
H	1.332952	-2.627877	-2.182882
C	2.876202	-4.835008	-2.408872
H	1.651534	-5.279187	-0.689382
H	0.728120	-5.064078	-2.175151
H	3.069749	-5.886195	-2.645284
H	2.824027	-4.283252	-3.355198
H	3.737874	-4.453735	-1.848860
H	-1.065461	-2.953146	-1.484553

XIIIa

C	-1.554763	-0.728991	-3.212698
C	-1.497887	-1.988801	-2.529256
C	-0.157744	-2.429072	-2.492527
C	0.638998	-1.437054	-3.163341
C	-0.217449	-0.399720	-3.617174
H	-2.449650	-0.190643	-3.482664
H	-2.339807	-2.526019	-2.116650
H	0.202206	-3.349679	-2.060083
H	1.702863	-1.508026	-3.333389
H	0.081523	0.473513	-4.181982
Ru	-0.310287	-0.407901	-1.351501
P	1.850855	-0.820546	-0.257217
P	-1.769381	-0.602123	0.515209
C	3.373583	-0.036748	-1.016822
C	3.367158	0.438643	-2.337447
C	4.580298	0.017385	-0.298294
C	4.521509	0.967044	-2.918974
H	2.451292	0.425203	-2.914764
C	5.734464	0.547434	-0.877213
H	4.628237	-0.354733	0.718735
C	5.709427	1.027525	-2.188795
H	4.486134	1.336840	-3.939771
H	6.653836	0.583293	-0.299743
H	6.606964	1.444272	-2.636534
C	2.285181	-2.611699	-0.494095
C	3.253187	-3.029890	-1.418969
C	1.568505	-3.589472	0.214732
C	3.489709	-4.391846	-1.631829
H	3.825872	-2.303009	-1.983453
C	1.814295	-4.946580	0.012664
H	0.808896	-3.291351	0.922939
C	2.774686	-5.354375	-0.917999
H	4.239463	-4.695654	-2.356939
H	1.249677	-5.681040	0.580095
H	2.963898	-6.411132	-1.083376
C	2.264483	-0.542653	1.536168
C	2.622714	-1.551392	2.441591
C	2.306781	0.789173	1.977078
C	2.976069	-1.233652	3.756558
H	2.639534	-2.589935	2.139224
C	2.665749	1.109672	3.285225
H	2.072733	1.590906	1.290385
C	2.993304	0.093737	4.186816
H	3.243107	-2.033284	4.441633
H	2.694649	2.151881	3.589833
H	3.270469	0.334903	5.208995

C	-1.312054	-1.955392	1.705259
C	-0.680283	-1.741292	2.935094
C	-1.616590	-3.275881	1.321312
C	-0.354923	-2.822940	3.761011
H	-0.437880	-0.738993	3.266349
C	-1.303977	-4.350410	2.151161
H	-2.106394	-3.466242	0.371167
C	-0.664617	-4.126680	3.375563
H	0.143140	-2.636226	4.707780
H	-1.553060	-5.360765	1.839641
H	-0.414526	-4.962711	4.022195
C	-3.553898	-1.116237	0.269257
C	-4.220649	-1.065605	-0.959202
C	-4.285558	-1.532618	1.400026
C	-5.568230	-1.422847	-1.068427
H	-3.703613	-0.741164	-1.848030
C	-5.628818	-1.887610	1.295772
H	-3.806333	-1.572618	2.372384
C	-6.276763	-1.837431	0.057572
H	-6.056385	-1.372189	-2.037291
H	-6.169434	-2.202774	2.183624
H	-7.323107	-2.117035	-0.024467
C	-2.009551	0.899695	1.580638
C	-3.260903	1.492010	1.817224
C	-0.876666	1.485282	2.159897
C	-3.366401	2.618929	2.637249
H	-4.158962	1.086071	1.368135
C	-0.981930	2.592532	3.000232
H	0.097724	1.066329	1.960604
C	-2.232234	3.164910	3.243512
H	-4.342461	3.065717	2.804473
H	-0.084916	3.013956	3.443589
H	-2.321409	4.034168	3.888633
C	2.720096	3.531907	0.113708
C	1.360370	3.493125	-0.248661
C	0.475112	4.393100	0.368842
C	0.958024	5.249328	1.371173
C	2.299087	5.252315	1.747545
C	3.192762	4.396592	1.098097
H	3.412645	2.850557	-0.373558
H	0.261502	5.932636	1.851525
H	2.646152	5.925750	2.526110
H	4.246657	4.393956	1.360947
C	0.983439	2.469665	-1.318966
C	-0.333603	1.684704	-1.284726
H	1.788968	1.754467	-1.331274
C	-0.978142	4.502096	-0.029023
C	-1.288570	3.968300	-1.423253
H	-1.269474	5.558062	-0.000252
H	-1.618917	3.987211	0.696223
H	-2.252517	4.358793	-1.741304
H	-0.552474	4.305884	-2.157038
N	-1.385481	2.484099	-1.441400
C	-2.774468	2.031633	-1.613742
C	-3.331346	2.346974	-3.010202
H	-3.386936	2.515162	-0.845162
H	-2.789840	0.966370	-1.435581
C	-4.740858	1.779015	-3.198326
H	-3.361731	3.430090	-3.169133
H	-2.651198	1.944397	-3.769391
H	-5.161473	2.099405	-4.156730
H	-4.743271	0.685280	-3.182637

H	-5.414157	2.122793	-2.404628
H	1.019981	2.951335	-2.309565

TS$_{XIIIa\text{-}XII'a}$

C	1.344777	-0.012239	-3.381593
C	1.961847	1.103696	-2.739836
C	0.987852	2.121643	-2.543096
C	-0.247600	1.634147	-3.089089
C	-0.034488	0.323390	-3.595237
H	1.834417	-0.912085	-3.720651
H	3.003450	1.182805	-2.464992
H	1.155766	3.098089	-2.113978
H	-1.173221	2.190564	-3.131291
H	-0.771511	-0.298600	-4.086609
Ru	0.352038	0.302749	-1.357956
P	-1.383948	1.481341	-0.166921
P	1.949638	-0.008861	0.379260
C	-3.118104	1.267304	-0.837407
C	-3.344327	0.717514	-2.108027
C	-4.229006	1.709176	-0.096464
C	-4.638255	0.596120	-2.620462
H	-2.511179	0.353012	-2.695118
C	-5.521979	1.587730	-0.605904
H	-4.088939	2.153947	0.882726
C	-5.732204	1.027548	-1.868987
H	-4.786804	0.157004	-3.602823
H	-6.365192	1.932152	-0.014134
H	-6.739781	0.929186	-2.262430
C	-1.191839	3.320889	-0.374092
C	-2.121289	4.101413	-1.079667
C	-0.044000	3.949309	0.134270
C	-1.909145	5.472093	-1.257341
H	-3.013180	3.653514	-1.500722
C	0.159901	5.318904	-0.029413
H	0.706758	3.364223	0.644021
C	-0.773725	6.087736	-0.729119
H	-2.639586	6.056026	-1.810343
H	1.054872	5.776128	0.382512
H	-0.614759	7.153576	-0.865617
C	-1.733797	1.290198	1.650410
C	-1.218828	2.166272	2.617104
C	-2.497420	0.190774	2.087954
C	-1.435923	1.934735	3.978732
H	-0.643293	3.034599	2.327343
C	-2.719063	-0.034854	3.445188
H	-2.935822	-0.495546	1.376087
C	-2.179991	0.832519	4.399636
H	-1.020797	2.625136	4.707453
H	-3.311697	-0.891671	3.752621
H	-2.346514	0.654688	5.458085
C	2.340352	1.551636	1.313268
C	2.039639	1.789420	2.659076
C	3.003413	2.555950	0.581550
C	2.368538	3.014042	3.252281
H	1.553772	1.032234	3.260436
C	3.344507	3.767731	1.177557
H	3.250363	2.390018	-0.462280
C	3.017344	4.005564	2.517397
H	2.119087	3.183031	4.296045
H	3.856812	4.528540	0.595693
H	3.273663	4.952737	2.982982
C	3.733438	-0.422559	-0.043681

C	4.183074	-0.856005	-1.295780
C	4.682602	-0.304794	0.991725
C	5.530747	-1.161012	-1.515290
H	3.498293	-0.968730	-2.121443
C	6.024633	-0.610851	0.778014
H	4.370618	0.024242	1.977624
C	6.456421	-1.039682	-0.480992
H	5.847572	-1.493806	-2.499665
H	6.733209	-0.512953	1.595565
H	7.503130	-1.275476	-0.650240
C	1.638215	-1.296159	1.679106
C	2.419055	-2.461203	1.768316
C	0.574073	-1.138339	2.578940
C	2.163186	-3.416109	2.754352
H	3.234224	-2.630455	1.075266
C	0.329147	-2.084383	3.576560
H	-0.064977	-0.271172	2.512886
C	1.128524	-3.224640	3.673468
H	2.780086	-4.308944	2.804483
H	-0.492608	-1.924886	4.268034
H	0.939436	-3.963346	4.446955
C	-4.149884	-2.525486	-0.121270
C	-2.774599	-2.835862	-0.024840
C	-2.372826	-3.980251	0.696336
C	-3.352256	-4.792260	1.276253
C	-4.709228	-4.481739	1.167277
C	-5.111130	-3.341411	0.464577
H	-4.452253	-1.630465	-0.658859
H	-3.043340	-5.677522	1.826009
H	-5.449982	-5.126137	1.632080
H	-6.164026	-3.090270	0.378769
C	-1.849291	-1.857194	-0.606012
C	-0.466876	-1.798549	-0.947836
H	-2.348602	-0.920139	-0.777627
C	-0.915506	-4.315341	0.815722
C	-0.177480	-4.255203	-0.530826
H	-0.807706	-5.322563	1.229493
H	-0.422072	-3.632222	1.512103
H	0.698880	-4.904922	-0.474956
H	-0.825036	-4.658445	-1.322312
N	0.322947	-2.926338	-0.897913
C	1.586191	-2.982581	-1.624316
C	1.492981	-3.620076	-3.017099
H	2.314879	-3.531990	-1.015367
H	1.937689	-1.964698	-1.721937
C	2.862799	-3.671116	-3.700033
H	1.085738	-4.634973	-2.940220
H	0.785727	-3.041415	-3.625870
H	2.796935	-4.133299	-4.690332
H	3.286922	-2.668348	-3.827838
H	3.575731	-4.253487	-3.104551
H	-1.219988	-1.716020	-1.940837

XII’a

C	-0.808247	-0.675704	-3.409770
C	-0.485398	-1.948250	-2.874036
C	0.934059	-2.007964	-2.656943
C	1.489204	-0.772743	-3.147817
C	0.422561	0.053711	-3.577661
H	-1.792867	-0.325720	-3.685915
H	-1.190245	-2.736978	-2.655247
H	1.493452	-2.858522	-2.293765

H	2.540457	-0.521376	-3.165845
H	0.509804	1.053709	-3.982464
Ru	0.163056	-0.430266	-1.380322
P	2.166671	0.048880	-0.169715
P	-1.132174	-1.553029	0.295164
C	3.142569	1.519539	-0.749692
C	2.915467	2.097218	-2.003442
C	4.195424	2.019011	0.039341
C	3.720386	3.140647	-2.469527
H	2.092946	1.752355	-2.613224
C	4.991181	3.067713	-0.418854
H	4.400332	1.585618	1.012604
C	4.758604	3.630477	-1.677980
H	3.520562	3.578682	-3.443305
H	5.796572	3.442402	0.206294
H	5.380974	4.446151	-2.034576
C	3.399269	-1.315551	-0.408697
C	4.588633	-1.125270	-1.129905
C	3.105695	-2.600002	0.077607
C	5.463530	-2.193041	-1.349136
H	4.841449	-0.149247	-1.527383
C	3.987257	-3.661109	-0.129772
H	2.186271	-2.774773	0.618294
C	5.169334	-3.461937	-0.847614
H	6.377849	-2.026252	-1.911673
H	3.739593	-4.642070	0.264288
H	5.853552	-4.288613	-1.015970
C	2.169400	0.383881	1.646554
C	2.622965	-0.519609	2.617042
C	1.657482	1.627541	2.057006
C	2.550278	-0.188244	3.974478

H	3.038547	-1.478234	2.331574
C	1.598265	1.958681	3.408404
H	1.310372	2.343705	1.320787
C	2.040069	1.048394	4.373823
H	2.902555	-0.898987	4.716623
H	1.198982	2.924736	3.702638
H	1.989709	1.302735	5.428620
C	-0.288186	-3.054736	0.986284
C	0.107552	-3.211419	2.321124
C	0.005177	-4.086210	0.073478
C	0.809608	-4.352640	2.724452
H	-0.125694	-2.458794	3.062047
C	0.689964	-5.228847	0.480381
H	-0.306178	-4.002034	-0.961234
C	1.107245	-5.361256	1.808736
H	1.115541	-4.448924	3.762262
H	0.903794	-6.011904	-0.241224
H	1.651542	-6.246041	2.125585
C	-2.683961	-2.331734	-0.368457
C	-3.386816	-1.718445	-1.413504
C	-3.235708	-3.473339	0.235410
C	-4.606090	-2.233695	-1.858331
H	-2.980960	-0.827639	-1.873750
C	-4.450180	-3.994802	-0.213432
H	-2.722984	-3.957684	1.059665
C	-5.138745	-3.378163	-1.261642
H	-5.135885	-1.739092	-2.667639
H	-4.860161	-4.881874	0.260895
H	-6.084278	-3.785385	-1.607851
C	-1.848520	-0.661896	1.759913
C	-3.226314	-0.384211	1.823646

C	-1.032800	-0.218123	2.814108
C	-3.768421	0.291219	2.918459
H	-3.889177	-0.695070	1.026477
C	-1.580617	0.436326	3.918738
H	0.033611	-0.381294	2.784707
C	-2.950691	0.694188	3.976227
H	-4.835459	0.493342	2.943884
H	-0.924786	0.755223	4.722503
H	-3.376386	1.209806	4.832183
C	0.744194	4.262962	-0.900247
C	-0.355691	3.530526	-0.406086
C	-0.810497	3.818341	0.904131
C	-0.165378	4.806850	1.656508
C	0.938394	5.503190	1.159915
C	1.393086	5.224711	-0.131766
H	1.093636	4.061613	-1.906754
H	-0.528955	5.019262	2.659414
H	1.433466	6.251746	1.771828
H	2.249226	5.753297	-0.541427
C	-0.950249	2.510074	-1.271715
C	-2.228884	1.988301	-1.282420
H	-0.380396	2.277269	-2.164704
C	-1.957166	3.049554	1.496545
C	-3.256818	3.107843	0.676949
H	-2.173284	3.430987	2.498225
H	-1.680300	1.999241	1.614058
H	-4.096507	2.836832	1.322652
H	-3.431256	4.132211	0.323519
N	-3.277645	2.169070	-0.444890
C	-4.594616	1.647061	-0.819815
C	-5.498129	2.679294	-1.505002
H	-5.080449	1.268187	0.087401
H	-4.450592	0.791144	-1.482519
C	-6.851493	2.072826	-1.882975
H	-5.645906	3.537837	-0.839032
H	-4.986199	3.055876	-2.399876
H	-7.493490	2.810036	-2.375767
H	-6.727962	1.225399	-2.568173
H	-7.382329	1.707303	-0.995972
H	-2.463954	1.333415	-2.115218

2a

C	2.779727	1.433058	0.227677
C	1.618949	0.729984	-0.170588
C	1.689651	-0.687553	-0.221323
C	2.868700	-1.331627	0.171839
C	3.999920	-0.619849	0.576875
C	3.950907	0.776861	0.594513
H	2.745750	2.520016	0.253019
H	2.902705	-2.418747	0.142331
H	4.904833	-1.147110	0.865563
H	4.821159	1.353422	0.897585
C	0.437657	1.509988	-0.525220
C	-0.872464	1.159760	-0.679662
H	0.626099	2.570570	-0.673694
C	0.518830	-1.507143	-0.702894
C	-0.817876	-1.203530	0.008299
H	0.745651	-2.569035	-0.563127
H	-1.489394	-2.059081	-0.108232
H	-0.637291	-1.076126	1.084906

N	-1.505242	-0.045046	-0.548155
C	-2.967933	-0.077561	-0.555409
C	-3.604799	0.120467	0.827558
H	-3.286160	-1.040633	-0.974102
H	-3.325698	0.699525	-1.240246
C	-5.133128	0.074494	0.766100
H	-3.234531	-0.654825	1.509850
H	-3.269099	1.083791	1.232546
H	-5.575711	0.220183	1.757070
H	-5.526839	0.856906	0.106245
H	-5.485094	-0.890624	0.382399
H	-1.553476	1.959291	-0.962431
H	0.364949	-1.356301	-1.779183

f. Coordinates for aminoketene VIIb formation. 1b to VIIb.

1b

C	2.243223	-0.342807	-0.245066
C	1.121708	0.476774	-0.010451
C	-0.136568	-0.104917	0.283569
C	-0.221640	-1.498988	0.335358
C	0.892097	-2.308191	0.099700
C	2.129150	-1.729341	-0.192583
H	3.198361	0.122371	-0.469451
H	-1.177010	-1.963777	0.560128
H	0.791983	-3.389040	0.144177
H	2.998899	-2.352452	-0.378397
C	1.275139	1.899263	-0.064372
C	1.408556	3.103002	-0.108875
H	1.530862	4.163879	-0.147297
C	-1.344697	0.784123	0.580684
H	-1.294601	1.668767	-0.065041
H	-1.242440	1.159642	1.608078
N	-2.673515	0.202842	0.439053
C	-3.020020	-0.183164	-0.927526
H	-3.037683	0.711697	-1.561194
H	-4.023968	-0.619575	-0.936950
H	-2.781133	-0.591219	1.064954
H	-2.330346	-0.904703	-1.395012

IIb

C	-0.842007	1.655720	-2.724866
C	-0.379572	0.351286	-3.047916
C	1.039620	0.327370	-2.906682
C	1.455383	1.662010	-2.551351
C	0.304256	2.474203	-2.434605
H	-1.870594	1.985332	-2.731347
H	-1.005252	-0.481746	-3.334428
H	1.688283	-0.515027	-3.098087
H	2.476172	1.986486	-2.405133
H	0.286463	3.522645	-2.171899
Ru	0.177356	0.781433	-0.888405
P	2.293510	0.570649	0.239460
P	-0.878081	-1.260619	-0.253102
C	3.063306	2.210129	0.634528
C	2.477991	3.421340	0.245231
C	4.285056	2.244118	1.333730
C	3.097001	4.639559	0.541242
H	1.531926	3.421350	-0.279223
C	4.903601	3.458412	1.624769
H	4.756417	1.318379	1.648235

C	4.310089	4.661311	1.229042
H	2.624487	5.568473	0.235221
H	5.847228	3.464896	2.162326
H	4.790937	5.607553	1.459542
C	3.574187	-0.224114	-0.843097
C	4.677184	0.486265	-1.344671
C	3.392089	-1.554678	-1.254199
C	5.581873	-0.129580	-2.214036
H	4.840234	1.521400	-1.070289
C	4.303529	-2.171627	-2.110483
H	2.524063	-2.108739	-0.927357
C	5.404047	-1.460403	-2.594438
H	6.427062	0.438486	-2.591937
H	4.139334	-3.203556	-2.405609
H	6.111057	-1.936267	-3.267694
C	2.469148	-0.268417	1.883689
C	2.720205	-1.643172	1.997022
C	2.276986	0.477820	3.062899
C	2.750892	-2.261424	3.248899
H	2.899346	-2.247624	1.119165
C	2.307928	-0.142859	4.311572
H	2.115220	1.549050	3.017354
C	2.536846	-1.517539	4.409369
H	2.945157	-3.328397	3.310035
H	2.156732	0.451755	5.207747
H	2.557526	-2.000860	5.381536
C	0.106714	-2.779174	-0.622505
C	0.599218	-3.606439	0.395929
C	0.400737	-3.098680	-1.960544
C	1.393918	-4.714012	0.084363
H	0.376930	-3.385887	1.433485

C	1.178664	-4.212938	-2.269616
H	0.016973	-2.481360	-2.764906
C	1.687203	-5.018996	-1.245436
H	1.777343	-5.339080	0.885700
H	1.392764	-4.448009	-3.308090
H	2.301173	-5.882080	-1.485512
C	-2.426014	-1.592237	-1.228995
C	-3.261434	-0.522574	-1.581370
C	-2.827855	-2.895412	-1.568767
C	-4.473465	-0.742231	-2.237103
H	-2.966524	0.489551	-1.347727
C	-4.036126	-3.115483	-2.234440
H	-2.205740	-3.745750	-1.312564
C	-4.864825	-2.041786	-2.567157
H	-5.104408	0.104792	-2.491480
H	-4.328686	-4.130144	-2.488738
H	-5.805030	-2.216578	-3.082147
C	-1.452629	-1.570014	1.480031
C	-2.505876	-2.456320	1.763726
C	-0.810157	-0.925817	2.544333
C	-2.903946	-2.681375	3.081579
H	-3.030476	-2.964498	0.963595
C	-1.205882	-1.154063	3.862947
H	0.000849	-0.244067	2.346737
C	-2.257340	-2.030752	4.135495
H	-3.725506	-3.362830	3.282371
H	-0.687869	-0.645861	4.670532
H	-2.572584	-2.205474	5.160207
C	-2.649822	3.792640	-0.858608
C	-2.632843	2.659876	-0.019586
C	-3.853304	2.120706	0.461093

C	-5.049305	2.708790	0.036629
C	-5.057646	3.820180	-0.808493
C	-3.852992	4.373806	-1.247697
H	-1.708408	4.206082	-1.204190
H	-5.992984	2.290344	0.368753
H	-6.003652	4.255062	-1.117846
H	-3.849454	5.245972	-1.894434
C	-1.355951	2.090794	0.342671
C	-0.274687	1.908276	0.944526
H	0.303275	2.125409	1.824544
C	-3.827026	0.919568	1.394054
H	-3.544508	1.255568	2.400455
H	-3.008555	0.272979	1.075333
N	-5.038853	0.118934	1.506846
C	-5.996100	0.558051	2.519592
H	-5.552147	0.431238	3.514101
H	-6.886784	-0.076511	2.472046
H	-5.483060	0.023012	0.597878
H	-6.323082	1.607849	2.436506

$TS_{IIb\text{-}IIIb}$

C	-1.401369	0.677772	-2.755809
C	-0.398730	-0.279988	-3.056184
C	0.881392	0.362141	-3.004588
C	0.651542	1.755927	-2.720638
C	-0.740819	1.943720	-2.550635
H	-2.465690	0.497511	-2.702235
H	-0.570770	-1.325504	-3.268381
H	1.835804	-0.099543	-3.213419
H	1.414037	2.517811	-2.634078
H	-1.231062	2.873865	-2.296368
Ru	-0.011959	0.492739	-0.983337
P	1.876772	1.232722	0.247103
P	-0.081442	-1.730290	-0.173977
C	1.690342	2.945208	0.928167
C	0.781058	3.835329	0.336413
C	2.478111	3.393209	2.001904
C	0.659197	5.144669	0.806951
H	0.164039	3.502809	-0.489703
C	2.353087	4.701093	2.472453
H	3.192675	2.724458	2.470617
C	1.443195	5.579559	1.877477
H	-0.051642	5.820507	0.339934
H	2.967453	5.033141	3.304505
H	1.345594	6.595979	2.247945
C	3.365587	1.370737	-0.834966
C	3.985273	2.596811	-1.113306
C	3.868736	0.200741	-1.428419
C	5.085213	2.650080	-1.975378
H	3.617434	3.512635	-0.663294
C	4.973764	0.255393	-2.276540
H	3.398662	-0.752969	-1.224357
C	5.582948	1.482740	-2.556569
H	5.553955	3.607198	-2.185965
H	5.354220	-0.660731	-2.719108
H	6.439952	1.527091	-3.222508
C	2.499843	0.324209	1.726146
C	3.840817	-0.045481	1.902883
C	1.569478	0.003352	2.725776
C	4.231383	-0.752726	3.042738
H	4.582753	0.202094	1.152482
C	1.958023	-0.705783	3.861425

H	0.531101	0.290279	2.612762
C	3.291689	-1.094297	4.018005
H	5.271837	-1.040848	3.163106
H	1.217001	-0.964872	4.611724
H	3.596678	-1.654993	4.896894
C	1.535023	-2.558232	-0.501193
C	2.498329	-2.745596	0.501363
C	1.873554	-2.873423	-1.830785
C	3.763659	-3.246497	0.183145
H	2.276972	-2.493542	1.531050
C	3.134983	-3.378609	-2.144040
H	1.150601	-2.726556	-2.626067
C	4.086478	-3.566130	-1.136130
H	4.496789	-3.376298	0.973648
H	3.374979	-3.621127	-3.175244
H	5.070851	-3.954850	-1.379944
C	-1.316505	-2.778384	-1.078274
C	-2.579733	-2.227034	-1.338424
C	-1.071609	-4.115691	-1.426563
C	-3.568499	-2.987795	-1.964466
H	-2.807336	-1.211858	-1.034314
C	-2.061412	-4.872360	-2.058026
H	-0.111336	-4.572664	-1.213787
C	-3.309634	-4.309497	-2.335477
H	-4.541694	-2.543845	-2.153138
H	-1.855040	-5.903760	-2.329933
H	-4.076932	-4.899662	-2.828461
C	-0.554360	-2.221362	1.548257
C	-0.178564	-3.461998	2.088831
C	-1.413816	-1.395994	2.286992
C	-0.608645	-3.839075	3.361577
H	0.452343	-4.138473	1.522744
C	-1.849474	-1.777155	3.558234
H	-1.753986	-0.456176	1.871832
C	-1.438154	-2.994738	4.104482
H	-0.297208	-4.796146	3.770198
H	-2.511317	-1.121768	4.117120
H	-1.770027	-3.289463	5.095835
C	-4.145800	3.530574	-0.706850
C	-4.011025	2.331472	0.033465
C	-5.164857	1.684823	0.556190
C	-6.413200	2.254131	0.303695
C	-6.538604	3.428615	-0.444417
C	-5.402917	4.068967	-0.951184
H	-3.251598	4.019751	-1.080459
H	-7.302123	1.772540	0.702214
H	-7.523892	3.848065	-0.625583
H	-5.500000	4.985306	-1.524883
C	-2.718477	1.812324	0.251223
C	-1.560141	1.376642	0.342192
H	-0.786034	1.390768	1.102418
C	-5.067859	0.392060	1.330736
H	-5.898257	0.348803	2.057177
H	-4.137195	0.363514	1.907711
N	-5.063518	-0.755542	0.414940
H	-5.910259	-0.711467	-0.149756
C	-5.042989	-2.028921	1.136141
H	-5.855495	-2.131036	1.876990
H	-4.090793	-2.133757	1.666417
H	-5.118237	-2.853605	0.423006

IIIb

C	-0.988407	0.599904	-3.081553
C	-0.042213	-0.448641	-3.182359
C	1.266235	0.099930	-2.997193
C	1.118936	1.529515	-2.865386
C	-0.261497	1.832688	-2.895802
H	-2.062564	0.498876	-3.142672
H	-0.275157	-1.493462	-3.330473
H	2.197981	-0.445058	-3.040164
H	1.924793	2.240363	-2.745605
H	-0.702621	2.814460	-2.787535
Ru	0.141552	0.481356	-1.118717
P	1.974158	1.187916	0.221726
P	-0.162472	-1.671356	-0.170703
C	1.858834	2.968321	0.704372
C	0.985962	3.835363	0.030984
C	2.671728	3.479749	1.731145
C	0.924222	5.187840	0.376436
H	0.349317	3.454415	-0.758530
C	2.608609	4.830285	2.073159
H	3.354883	2.824616	2.262656
C	1.734134	5.687210	1.397572
H	0.239755	5.846397	-0.150582
H	3.241146	5.212955	2.868911
H	1.683811	6.737714	1.668990
C	3.568386	1.082991	-0.698265
C	4.301665	2.222887	-1.056946
C	4.038069	-0.183156	-1.086113
C	5.483783	2.095270	-1.792650
H	3.959533	3.210725	-0.768006
C	5.223696	-0.306897	-1.808903
H	3.480115	-1.071914	-0.820536
C	5.948343	0.833739	-2.168177
H	6.041143	2.986280	-2.067150
H	5.575958	-1.294479	-2.092340
H	6.869214	0.738573	-2.736185
C	2.336985	0.397024	1.844017
C	3.586892	-0.132268	2.194453
C	1.291019	0.354795	2.777098
C	3.771285	-0.723776	3.446647
H	4.416309	-0.097791	1.497950
C	1.473971	-0.241587	4.023397
H	0.328105	0.784927	2.525534
C	2.715178	-0.790905	4.357855
H	4.742050	-1.137057	3.704814
H	0.645879	-0.286504	4.724166
H	2.859125	-1.262457	5.325688
C	1.398490	-2.645514	-0.238651
C	2.198047	-2.824176	0.900704
C	1.872746	-3.103910	-1.481298
C	3.439621	-3.456735	0.799360
H	1.865417	-2.464259	1.866953
C	3.109263	-3.742094	-1.576559
H	1.276548	-2.969207	-2.377212
C	3.897905	-3.919197	-0.435179
H	4.048189	-3.578481	1.690365
H	3.457332	-4.097204	-2.542111
H	4.862943	-4.411595	-0.510461
C	-1.391523	-2.666257	-1.131904
C	-2.569723	-2.024785	-1.542818
C	-1.235364	-4.038627	-1.380319
C	-3.564088	-2.739004	-2.212776
H	-2.724937	-0.975597	-1.321824

C	-2.230105	-4.747434	-2.057649
H	-0.341974	-4.559569	-1.053715
C	-3.393532	-4.099507	-2.480310
H	-4.474329	-2.229187	-2.514414
H	-2.094353	-5.807677	-2.251597
H	-4.165443	-4.653599	-3.006737
C	-0.829998	-1.980614	1.528540
C	-0.733331	-3.262311	2.098604
C	-1.541559	-0.990324	2.215848
C	-1.301264	-3.528109	3.344066
H	-0.211569	-4.055781	1.573920
C	-2.116026	-1.258523	3.460850
H	-1.659857	-0.006498	1.783931
C	-1.990570	-2.525027	4.032440
H	-1.207925	-4.520961	3.774537
H	-2.663478	-0.474956	3.976469
H	-2.433214	-2.734120	5.002016
C	-4.253472	3.557817	-0.824883
C	-4.044243	2.347017	-0.127110
C	-5.129906	1.730003	0.548233
C	-6.384639	2.341506	0.492098
C	-6.584839	3.535140	-0.206095
C	-5.514310	4.144365	-0.866502
H	-3.413069	4.025916	-1.328708
H	-7.219155	1.872263	1.007001
H	-7.571630	3.988037	-0.232181
H	-5.661104	5.074145	-1.407938
C	-2.739157	1.785270	-0.118246
C	-1.601438	1.321930	-0.197655
H	-0.609590	1.402377	0.352969
C	-4.969085	0.409415	1.263278
H	-5.721036	0.341721	2.069668
H	-3.986022	0.351903	1.740558
N	-5.068081	-0.704419	0.310727
C	-4.975186	-2.007265	0.969907
H	-3.970206	-2.139662	1.381764
H	-5.140568	-2.799604	0.234873
H	-5.970200	-0.637391	-0.157612
H	-5.697749	-2.139707	1.795098

$TS_{IIIb\text{-}IVb}$

C	-0.494834	-0.177367	-3.403106
C	0.619150	-1.039710	-3.196245
C	1.784991	-0.246150	-2.971035
C	1.383833	1.130130	-3.058685
C	-0.012005	1.173241	-3.325863
H	-1.510774	-0.485833	-3.606538
H	0.575857	-2.119969	-3.205584
H	2.787921	-0.610148	-2.799681
H	2.034440	1.986642	-2.940939
H	-0.610003	2.065387	-3.455497
Ru	0.298842	0.182455	-1.303989
P	1.636297	1.433721	0.173507
P	0.074564	-1.823096	-0.105489
C	1.333540	3.262358	0.084983
C	0.466894	3.804428	-0.872843
C	1.977108	4.135010	0.982884
C	0.244016	5.183441	-0.936696
H	-0.043318	3.148597	-1.565023
C	1.759559	5.510268	0.915776
H	2.649865	3.738623	1.737069
C	0.889884	6.038774	-0.044109

H	-0.436407	5.583328	-1.683335
H	2.266266	6.169339	1.614793
H	0.717069	7.110115	-0.091371
C	3.433625	1.277178	-0.250204
C	4.228362	2.372731	-0.620686
C	4.003921	-0.007216	-0.276992
C	5.563726	2.186143	-0.990326
H	3.817593	3.375358	-0.631344
C	5.340666	-0.189077	-0.629782
H	3.401507	-0.872291	-0.036256
C	6.126289	0.908860	-0.990078
H	6.161645	3.046193	-1.278120
H	5.758216	-1.191714	-0.632924
H	7.165192	0.768654	-1.274273
C	1.543697	1.180278	1.988113
C	2.362236	0.269336	2.662262
C	0.556484	1.865828	2.723075
C	2.199485	0.038273	4.031189
H	3.140748	-0.266816	2.133982
C	0.399612	1.641773	4.089664
H	-0.091471	2.586302	2.234002
C	1.218343	0.721215	4.749703
H	2.848588	-0.674633	4.531618
H	-0.365102	2.185018	4.637460
H	1.093836	0.542526	5.813662
C	1.672007	-2.703339	0.194557
C	2.189576	-2.862669	1.488546
C	2.453835	-3.120272	-0.899218
C	3.462058	-3.407395	1.683876
H	1.613047	-2.549775	2.350808
C	3.715774	-3.679095	-0.701381

H	2.082622	-3.008472	-1.911506
C	4.229086	-3.816083	0.592414
H	3.849158	-3.510770	2.693572
H	4.300929	-3.999401	-1.558545
H	5.217066	-4.240349	0.745582
C	-0.933342	-3.038014	-1.080590
C	-2.167626	-2.592196	-1.581953
C	-0.554355	-4.371238	-1.294077
C	-2.997122	-3.458850	-2.293615
H	-2.494459	-1.574436	-1.397944
C	-1.384634	-5.235181	-2.015065
H	0.384128	-4.746539	-0.901547
C	-2.604534	-4.781950	-2.519713
H	-3.951519	-3.098835	-2.667697
H	-1.074907	-6.264124	-2.176517
H	-3.247763	-5.454980	-3.079491
C	-0.786900	-1.968364	1.522966
C	-1.032276	-3.242976	2.067841
C	-1.232871	-0.840777	2.215727
C	-1.702137	-3.375436	3.282224
H	-0.699774	-4.132569	1.541838
C	-1.904036	-0.973102	3.435328
H	-1.060369	0.143560	1.808903
C	-2.140991	-2.238356	3.970329
H	-1.883867	-4.365197	3.691196
H	-2.237079	-0.083606	3.961449
H	-2.665174	-2.342845	4.915896
C	-4.060484	3.249197	-1.115393
C	-3.858809	2.041464	-0.412995
C	-4.908080	1.472723	0.354424
C	-6.136805	2.134483	0.381004

C	-6.330861	3.331704	-0.314541
C	-5.291949	3.893289	-1.062305
H	-3.241199	3.667156	-1.692217
H	-6.952741	1.708138	0.957907
H	-7.296928	3.826313	-0.273031
H	-5.443482	4.823232	-1.601250
C	-2.585872	1.406906	-0.502182
C	-1.413165	0.951284	-0.611014
H	-1.405701	1.805331	0.224992
C	-4.724159	0.163025	1.081835
H	-5.496196	0.075689	1.865751
H	-3.753527	0.148644	1.587905
N	-4.746051	-0.963379	0.140334
H	-5.640178	-0.950486	-0.347878
C	-4.601312	-2.248590	0.826826
H	-4.701980	-3.061247	0.102649
H	-3.603316	-2.317558	1.268266
H	-5.339626	-2.404941	1.632990

IVb

C	0.796931	-0.949983	-3.107104
C	0.185351	0.334923	-3.225264
C	-1.223386	0.179028	-3.116609
C	-1.483602	-1.218734	-2.928883
C	-0.244445	-1.917150	-2.938320
H	1.857578	-1.150477	-3.156264
H	0.719972	1.262573	-3.373595
H	-1.961827	0.966376	-3.161367
H	-2.460513	-1.668427	-2.812267
H	-0.111124	-2.985709	-2.842470
Ru	-0.237925	-0.497888	-1.116155
P	-2.216609	-0.800623	0.178762
P	0.677969	1.449982	-0.160570
C	-2.692000	-2.576761	0.334781
C	-1.912537	-3.596953	-0.225161
C	-3.851257	-2.922044	1.055647
C	-2.288516	-4.935983	-0.082474
H	-1.004571	-3.349986	-0.759162
C	-4.226462	-4.257086	1.191881
H	-4.459996	-2.147753	1.512019
C	-3.446016	-5.268056	0.621247
H	-1.670873	-5.715305	-0.519456
H	-5.125255	-4.508327	1.747314
H	-3.738017	-6.308357	0.731814
C	-3.648823	0.000597	-0.675129
C	-4.744098	-0.731327	-1.159062
C	-3.598567	1.384012	-0.918110
C	-5.774798	-0.086002	-1.848260
H	-4.802928	-1.803045	-1.012177
C	-4.635925	2.026253	-1.593178
H	-2.741528	1.962803	-0.600771
C	-5.729066	1.292505	-2.061158
H	-6.613748	-0.668311	-2.218187
H	-4.577209	3.097300	-1.761829
H	-6.533881	1.790138	-2.594274
C	-2.331921	-0.255360	1.940180
C	-2.766132	1.027712	2.300097
C	-1.926483	-1.144802	2.951842
C	-2.783356	1.417133	3.641656
H	-3.099649	1.732107	1.549741
C	-1.951258	-0.753895	4.289770
H	-1.586971	-2.144152	2.699599

C	-2.375190	0.531102	4.639113
H	-3.122027	2.415865	3.901708
H	-1.636934	-1.454151	5.058054
H	-2.390450	0.836534	5.681080
C	-0.413248	2.927974	-0.292371
C	-0.990350	3.502548	0.850270
C	-0.770787	3.425217	-1.558651
C	-1.917893	4.540386	0.727238
H	-0.732583	3.136573	1.837000
C	-1.684765	4.471344	-1.676623
H	-0.336046	2.999867	-2.455449
C	-2.268761	5.026093	-0.533262
H	-2.363594	4.967087	1.621060
H	-1.945239	4.848239	-2.661281
H	-2.988939	5.833669	-0.626374
C	2.225829	1.911806	-1.063011
C	3.170312	0.900957	-1.310456
C	2.517588	3.226101	-1.456430
C	4.375428	1.196810	-1.946773
H	2.973346	-0.119685	-1.007310
C	3.724615	3.517946	-2.098911
H	1.812315	4.027022	-1.264253
C	4.654140	2.506717	-2.347702
H	5.092047	0.400859	-2.126476
H	3.935480	4.539829	-2.400878
H	5.590398	2.737152	-2.847739
C	1.259072	1.511537	1.586802
C	2.063358	2.585585	2.010727
C	0.891474	0.530995	2.512974
C	2.474609	2.674007	3.339462
H	2.366706	3.353658	1.306797
C	1.304081	0.621635	3.844316
H	0.287536	-0.306039	2.200466
C	2.092819	1.693169	4.260704
H	3.094916	3.507848	3.654859
H	1.003932	-0.147491	4.548919
H	2.417438	1.762938	5.294803
C	3.124570	-3.050003	-1.367259
C	3.069569	-2.588846	-0.040371
C	4.272918	-2.416201	0.682830
C	5.486014	-2.710927	0.046694
C	5.533723	-3.148230	-1.279440
C	4.344685	-3.316016	-1.990388
H	2.195224	-3.217673	-1.901017
H	6.410848	-2.583104	0.603949
H	6.490114	-3.361853	-1.748242
H	4.361806	-3.669556	-3.017499
C	1.745873	-2.270794	0.562793
C	0.854549	-1.519040	-0.058312
H	1.493350	-2.642523	1.556885
C	4.282608	-1.809996	2.076210
H	5.198157	-2.116663	2.595792
H	3.440041	-2.188120	2.664781
N	4.220817	-0.341844	2.106260
C	5.369931	0.329950	1.507614
H	6.270253	0.105397	2.093080
H	5.205210	1.411425	1.542936
H	3.370135	-0.033991	1.641746
H	5.577221	0.055941	0.461043

$TS_{IVb\text{-}Vb}$

C	0.257341	-1.775672	-3.050149

C	1.554137	-2.149904	-2.560299
C	2.424596	-1.048976	-2.699385
C	1.676447	0.030203	-3.297342
C	0.356302	-0.429235	-3.531817
H	-0.606039	-2.420772	-3.106301
H	1.810363	-3.111391	-2.137264
H	3.465030	-1.018368	-2.410865
H	2.061135	1.004184	-3.562550
H	-0.438571	0.149755	-3.982065
Ru	0.681669	-0.436645	-1.279807
P	1.966149	1.288888	-0.189121
P	0.364938	-1.953731	0.484679
C	1.902775	2.921131	-1.075671
C	0.997800	3.147416	-2.119914
C	2.791811	3.947633	-0.715704
C	0.977651	4.372917	-2.790477
H	0.304269	2.364951	-2.400062
C	2.766648	5.175118	-1.379048
H	3.505783	3.790263	0.087184
C	1.859541	5.390560	-2.420330
H	0.270498	4.530474	-3.600396
H	3.457674	5.959883	-1.084777
H	1.843059	6.343952	-2.940821
C	3.814724	1.019337	-0.268084
C	4.455575	1.222802	-1.505706
C	4.601824	0.622124	0.821268
C	5.824974	1.009349	-1.653551
H	3.888288	1.568142	-2.362324
C	5.976684	0.411747	0.674573
H	4.165887	0.479257	1.799786
C	6.593666	0.595488	-0.561755

H	6.291291	1.175545	-2.620591
H	6.560250	0.104404	1.537787
H	7.661431	0.430531	-0.673423
C	1.673741	1.763988	1.579697
C	1.916551	0.838363	2.605824
C	1.130868	3.012438	1.925130
C	1.653034	1.155420	3.939696
H	2.286503	-0.149993	2.373649
C	0.857621	3.326378	3.257310
H	0.898926	3.737537	1.155362
C	1.121860	2.401786	4.271974
H	1.849809	0.416075	4.710667
H	0.433634	4.296838	3.499963
H	0.908375	2.649005	5.307939
C	1.872010	-2.388866	1.467731
C	1.806011	-2.794661	2.810909
C	3.121295	-2.351056	0.830720
C	2.969095	-3.138813	3.501466
H	0.852830	-2.832923	3.326366
C	4.281212	-2.714124	1.518192
H	3.190289	-2.020534	-0.199161
C	4.209070	-3.102434	2.857096
H	2.904692	-3.438417	4.543680
H	5.240126	-2.670070	1.010986
H	5.112421	-3.371057	3.397198
C	-0.127101	-3.621624	-0.166501
C	-1.377761	-3.759976	-0.790336
C	0.714873	-4.740303	-0.085712
C	-1.776470	-4.986200	-1.321554
H	-2.045177	-2.910419	-0.858792
C	0.317604	-5.966574	-0.628414

H	1.681753	-4.667725	0.399283
C	-0.926518	-6.093869	-1.247432
H	-2.749937	-5.068833	-1.795422
H	0.983877	-6.821950	-0.559401
H	-1.234239	-7.048167	-1.665355
C	-0.909281	-1.658016	1.788321
C	-1.617100	-2.717886	2.380637
C	-1.115271	-0.354728	2.263423
C	-2.498589	-2.479103	3.437019
H	-1.477232	-3.733930	2.028270
C	-1.982243	-0.122876	3.333561
H	-0.612824	0.476648	1.788287
C	-2.675687	-1.182101	3.923114
H	-3.039384	-3.309025	3.882594
H	-2.120825	0.887768	3.705517
H	-3.356306	-0.996456	4.749037
C	-3.045713	-1.279604	-2.872122
C	-3.232342	-0.823764	-1.557768
C	-4.259441	-1.406059	-0.773630
C	-5.024270	-2.445581	-1.313783
C	-4.800003	-2.918527	-2.609094
C	-3.809810	-2.324399	-3.393493
H	-2.302213	-0.796307	-3.492584
H	-5.802933	-2.894947	-0.702490
H	-5.401120	-3.732448	-3.004277
H	-3.636764	-2.664496	-4.410765
C	-2.410517	0.272176	-0.990136
C	-1.074647	0.318532	-0.940853
C	-4.531841	-0.915347	0.629995
H	-3.576903	-0.711390	1.135596
H	-5.029190	-1.703603	1.207455
N	-5.418052	0.264004	0.627178
C	-5.509690	0.878350	1.950639
H	-5.990455	0.179558	2.645834
H	-6.130269	1.779074	1.896739
H	-5.045723	0.952136	-0.024627
H	-2.988907	1.050246	-0.504747
C	-2.370166	3.536983	-0.971776
C	-3.458912	4.371755	-0.807675
C	-4.038396	4.556825	0.457394
C	-3.451388	3.876446	1.533890
C	-2.364874	3.044174	1.337074
H	-1.882046	3.344215	-1.916299
H	-3.857977	4.878187	-1.679956
H	-3.842542	3.985028	2.539474
H	-1.859956	2.501775	2.121382
C	-5.251455	5.421429	0.643614
H	-5.253612	6.259431	-0.059017
H	-6.159933	4.834539	0.457826
H	-5.311153	5.810231	1.663483
O	-0.813379	2.056470	-0.078700
N	-1.842099	2.882694	0.095735
H	-4.534745	1.156432	2.385780

Vb

C	-0.349275	-1.735017	-3.000146
C	0.641306	-2.568376	-2.400553
C	1.909258	-1.929489	-2.503038
C	1.696814	-0.677821	-3.167753
C	0.316571	-0.559726	-3.482161
H	-1.395545	-1.968041	-3.119441
H	0.455524	-3.532410	-1.947071

H	2.858010	-2.323199	-2.172320
H	2.463924	0.046123	-3.407279
H	-0.137522	0.260683	-4.015807
Ru	0.519140	-0.606199	-1.219596
P	2.106254	0.984495	-0.481381
P	0.076782	-1.780025	0.767806
C	1.913804	2.655149	-1.273436
C	0.987923	2.829957	-2.311248
C	2.691026	3.758346	-0.875252
C	0.829564	4.074348	-2.928405
H	0.374517	1.994139	-2.621903
C	2.533214	5.001159	-1.489517
H	3.422385	3.649552	-0.081006
C	1.598409	5.164301	-2.516576
H	0.100376	4.189039	-3.725711
H	3.141280	5.841201	-1.165966
H	1.473796	6.133012	-2.992142
C	3.828575	0.495626	-1.000496
C	4.654511	1.302920	-1.797275
C	4.312244	-0.762301	-0.601926
C	5.930894	0.868279	-2.169087
H	4.313038	2.271522	-2.142364
C	5.591415	-1.188277	-0.957453
H	3.679211	-1.425217	-0.025668
C	6.407732	-0.372946	-1.745976
H	6.550865	1.507566	-2.791517
H	5.941675	-2.161955	-0.625891
H	7.401354	-0.705099	-2.033066
C	2.395978	1.462070	1.297454
C	3.199107	0.672151	2.135639
C	1.745623	2.574412	1.861336
C	3.336847	0.976232	3.492250
H	3.717305	-0.194866	1.748810
C	1.888514	2.882476	3.213993
H	1.125321	3.210317	1.244670
C	2.681054	2.079900	4.038967
H	3.956555	0.341450	4.118855
H	1.375998	3.749186	3.622154
H	2.787922	2.314532	5.094060
C	1.562469	-2.546485	1.581721
C	1.911396	-2.373564	2.927822
C	2.379025	-3.362651	0.777906
C	3.063871	-2.973090	3.446826
H	1.295423	-1.772404	3.584915
C	3.519874	-3.971429	1.298591
H	2.122146	-3.523910	-0.262376
C	3.873982	-3.768129	2.636206
H	3.321237	-2.816636	4.490677
H	4.134432	-4.598030	0.658330
H	4.768206	-4.232226	3.042067
C	-0.963965	-3.290332	0.468858
C	-2.001762	-3.224898	-0.471977
C	-0.803554	-4.467917	1.215584
C	-2.858060	-4.308707	-0.670340
H	-2.141241	-2.321769	-1.050663
C	-1.653929	-5.557316	1.010200
H	-0.019386	-4.540515	1.961894
C	-2.682916	-5.481856	0.068042
H	-3.655595	-4.231853	-1.404173
H	-1.512394	-6.464392	1.591175
H	-3.343012	-6.330339	-0.088291
C	-0.847569	-1.037448	2.200159

C	-2.108618	-1.507890	2.601171
C	-0.272882	0.020811	2.922614
C	-2.764918	-0.941921	3.699998
H	-2.589834	-2.318260	2.067665
C	-0.921497	0.577425	4.025041
H	0.689946	0.405661	2.628605
C	-2.173024	0.096915	4.420007
H	-3.739731	-1.322393	3.990491
H	-0.444680	1.388144	4.567433
H	-2.682295	0.530048	5.276054
C	-2.877950	0.257367	-3.672870
C	-3.064219	0.115521	-2.289953
C	-4.083529	-0.751310	-1.828968
C	-4.819482	-1.500109	-2.756778
C	-4.582847	-1.390096	-4.128510
C	-3.617259	-0.492750	-4.589007
H	-2.140072	0.968170	-4.030418
H	-5.594328	-2.171703	-2.394780
H	-5.161126	-1.984154	-4.830678
H	-3.441276	-0.373931	-5.654635
C	-2.292053	0.927161	-1.312035
C	-1.056377	0.679520	-0.822300
C	-4.422513	-0.835663	-0.355702
H	-3.497943	-0.836290	0.241204
H	-4.933180	-1.783391	-0.148173
N	-5.329548	0.256990	0.036921
C	-5.664016	0.210279	1.459522
H	-6.243544	-0.696773	1.669735
H	-6.287990	1.072014	1.718403
H	-4.859820	1.140666	-0.152445
H	-2.868551	1.759632	-0.914798
C	-1.402713	3.796888	0.357402
C	-2.257937	4.755295	0.866944
C	-3.285786	4.391734	1.753778
C	-3.410130	3.033970	2.090546
C	-2.534152	2.100368	1.571315
H	-0.595700	3.989523	-0.337965
H	-2.122440	5.787650	0.564945
H	-4.191413	2.692951	2.759604
H	-2.580508	1.038788	1.762852
C	-4.202787	5.423316	2.338764
H	-5.171004	4.991521	2.603526
H	-3.757061	5.830689	3.255035
H	-4.354037	6.256296	1.647213
O	-0.639385	1.580144	0.262933
N	-1.562464	2.513531	0.734155
H	-4.789429	0.202302	2.131514

TS_{Vb-VIb}

C	-0.863009	-1.683078	-2.896916
C	-0.172887	-2.720476	-2.209612
C	1.229611	-2.496445	-2.325379
C	1.404501	-1.304387	-3.106077
C	0.124848	-0.805898	-3.459548
H	-1.930296	-1.600105	-3.019810
H	-0.644832	-3.541509	-1.687922
H	2.015435	-3.121253	-1.928540
H	2.352915	-0.867569	-3.388087
H	-0.062630	0.066291	-4.067457
Ru	0.315466	-0.740915	-1.188121
P	2.348337	0.308551	-0.546867
P	-0.445599	-1.574637	0.879506

C	2.693410	1.852674	-1.516675
C	1.818646	2.257642	-2.533675
C	3.821487	2.646757	-1.242226
C	2.061047	3.426590	-3.261112
H	0.934277	1.668545	-2.742167
C	4.064889	3.812758	-1.968484
H	4.510579	2.357750	-0.455032
C	3.183729	4.207423	-2.979798
H	1.366774	3.727400	-4.040679
H	4.941327	4.413285	-1.742268
H	3.371747	5.117856	-3.541752
C	3.802110	-0.779778	-0.943260
C	4.831851	-0.409789	-1.820669
C	3.845477	-2.058709	-0.361765
C	5.883165	-1.291097	-2.093516
H	4.823696	0.559879	-2.304214
C	4.902346	-2.930409	-0.620728
H	3.038259	-2.385736	0.281722
C	5.927526	-2.548920	-1.490759
H	6.668391	-0.987266	-2.780107
H	4.913868	-3.909628	-0.150366
H	6.748072	-3.228545	-1.702228
C	2.774086	0.888290	1.168157
C	3.377324	0.040696	2.110619
C	2.427654	2.190577	1.569528
C	3.608798	0.477039	3.417907
H	3.665928	-0.967411	1.845167
C	2.663909	2.627450	2.872018
H	1.974498	2.871742	0.862947
C	3.250475	1.768854	3.805954
H	4.069564	-0.200707	4.130794
H	2.384811	3.638327	3.155725
H	3.430176	2.105246	4.822929
C	0.751760	-2.728396	1.709146
C	1.317875	-2.513197	2.972451
C	1.138630	-3.874795	0.988583
C	2.269612	-3.400487	3.486745
H	1.028528	-1.657736	3.568974
C	2.075311	-4.766157	1.508115
H	0.707958	-4.075183	0.014592
C	2.655948	-4.524505	2.757592
H	2.703341	-3.207410	4.463897
H	2.355914	-5.644868	0.934388
H	3.394760	-5.211716	3.159514
C	-1.924218	-2.681348	0.690681
C	-2.881556	-2.391015	-0.291264
C	-2.150461	-3.763749	1.556099
C	-4.026952	-3.178400	-0.426739
H	-2.733622	-1.548333	-0.952756
C	-3.298176	-4.548489	1.423850
H	-1.434117	-3.998907	2.336355
C	-4.237499	-4.261850	0.429955
H	-4.746020	-2.944325	-1.206901
H	-3.456231	-5.384827	2.098887
H	-5.125929	-4.877796	0.324290
C	-1.036519	-0.466654	2.248736
C	-2.380444	-0.452756	2.659605
C	-0.132277	0.380797	2.908244
C	-2.795610	0.369114	3.712575
H	-3.112748	-1.083939	2.171507
C	-0.541919	1.183188	3.973865
H	0.898947	0.409612	2.596722

C	-1.877308	1.179136	4.383029
H	-3.839899	0.364313	4.011054
H	0.186302	1.817021	4.470109
H	-2.200468	1.806156	5.209018
C	-2.907403	0.722107	-3.635316
C	-3.129220	0.811657	-2.252704
C	-4.385081	0.400113	-1.741619
C	-5.337718	-0.140702	-2.613898
C	-5.077191	-0.270133	-3.980648
C	-3.859443	0.174092	-4.496939
H	-1.970931	1.095666	-4.034918
H	-6.295125	-0.453232	-2.209939
H	-5.830305	-0.696608	-4.637792
H	-3.653212	0.105208	-5.561584
C	-2.123282	1.422868	-1.352135
C	-0.927372	0.947269	-0.942591
C	-4.684265	0.564358	-0.263282
H	-4.866577	1.629924	-0.055534
H	-3.779514	0.308072	0.311826
H	-2.432843	2.375260	-0.921121
C	-0.138948	4.204038	-0.064281
C	-0.426733	5.440070	0.496307
C	-1.309549	5.527935	1.585704
C	-1.877009	4.338602	2.070642
C	-1.557834	3.125567	1.478428
H	0.537214	4.055716	-0.898933
H	0.041663	6.328147	0.085769
H	-2.563647	4.349409	2.910281
H	-1.966806	2.173832	1.789291
C	-1.659278	6.855192	2.193537
H	-2.530097	7.278087	1.677127
H	-1.919326	6.752782	3.250358
H	-0.837359	7.568812	2.093057
O	-0.136156	1.649302	-0.091902
N	-0.714162	3.112219	0.444694
N	-5.862162	-0.181655	0.174885
H	-5.675993	-1.173772	0.040953
C	-6.174932	0.043978	1.583405
H	-6.457357	1.092574	1.735948
H	-5.341159	-0.178007	2.270589
H	-7.027529	-0.578602	1.873020

Xb

C	-1.193345	-0.798370	-3.190850
C	-0.785088	-2.030851	-2.610802
C	0.636401	-2.092522	-2.652097
C	1.106861	-0.901357	-3.322957
C	-0.015912	-0.102783	-3.634800
H	-2.209743	-0.455942	-3.306413
H	-1.442380	-2.783372	-2.200287
H	1.246375	-2.910520	-2.296970
H	2.137986	-0.662730	-3.542929
H	0.000028	0.869710	-4.108705
Ru	0.097952	-0.375173	-1.356413
P	2.363578	-0.179641	-0.487627
P	-0.866874	-1.346252	0.609928
C	3.164031	1.367032	-1.098254
C	2.690995	2.052238	-2.225701
C	4.332033	1.821396	-0.462241
C	3.381409	3.161463	-2.718934
H	1.778663	1.735769	-2.712149
C	5.018728	2.930912	-0.956436

H	4.713271	1.307185	0.413957
C	4.547983	3.600942	-2.089138
H	3.000229	3.685513	-3.590516
H	5.920278	3.270225	-0.455054
H	5.082509	4.465055	-2.472653
C	3.493562	-1.505525	-1.077648
C	4.469131	-1.258890	-2.055469
C	3.335409	-2.810155	-0.582603
C	5.268727	-2.302888	-2.528148
H	4.610213	-0.259293	-2.451647
C	4.141624	-3.846789	-1.050431
H	2.589592	-3.014375	0.172392
C	5.108654	-3.596505	-2.028296
H	6.019395	-2.099404	-3.286178
H	4.009487	-4.847496	-0.649801
H	5.734216	-4.404471	-2.396310
C	2.637860	-0.069104	1.323554
C	3.607384	-0.820383	2.004525
C	1.856956	0.853559	2.034357
C	3.762044	-0.673551	3.384288
H	4.236373	-1.524327	1.473108
C	2.006336	0.987440	3.414034
H	1.140244	1.469649	1.508195
C	2.953871	0.218206	4.093952
H	4.511724	-1.263538	3.903436
H	1.378955	1.691584	3.951251
H	3.069075	0.320178	5.169138
C	0.143733	-2.779410	1.181901
C	0.963038	-2.716094	2.317825
C	0.132670	-3.958907	0.414559
C	1.743741	-3.815431	2.686021
H	1.000831	-1.815655	2.918861
C	0.907099	-5.056048	0.789220
H	-0.491557	-4.029483	-0.469515
C	1.715896	-4.987230	1.928484
H	2.376728	-3.746627	3.565493
H	0.881228	-5.961724	0.190510
H	2.321090	-5.840687	2.219651
C	-2.514820	-2.140178	0.291526
C	-3.393215	-1.567313	-0.634764
C	-2.941622	-3.262366	1.023376
C	-4.668043	-2.101521	-0.838293
H	-3.090249	-0.695881	-1.196717
C	-4.215251	-3.794912	0.821459
H	-2.284657	-3.732667	1.746145
C	-5.082736	-3.218154	-0.110396
H	-5.330646	-1.633746	-1.559570
H	-4.527463	-4.664114	1.393164
H	-6.072776	-3.636526	-0.266671
C	-1.269540	-0.413518	2.160078
C	-1.608178	-1.129640	3.323083
C	-1.347944	0.985952	2.185705
C	-1.990077	-0.461395	4.485297
H	-1.571420	-2.212862	3.332170
C	-1.737324	1.651991	3.350646
H	-1.099479	1.568645	1.311559
C	-2.054215	0.934561	4.503231
H	-2.239990	-1.033305	5.374186
H	-1.789957	2.734855	3.349105
H	-2.354917	1.456221	5.407197
C	-3.614014	1.648455	-3.270865
C	-3.485787	1.683695	-1.873041

C	-4.632202	1.455487	-1.075276
C	-5.859537	1.203326	-1.700007
C	-5.967169	1.154908	-3.092202
C	-4.838155	1.370859	-3.883351
H	-2.737297	1.852299	-3.879746
H	-6.734666	1.032431	-1.081354
H	-6.929978	0.952914	-3.553705
H	-4.909803	1.340957	-4.967015
C	-2.194358	2.062757	-1.251367
C	-1.053799	1.376850	-1.103164
C	-4.499823	1.435834	0.433776
H	-4.462235	2.467058	0.817108
H	-3.521375	0.998324	0.684347
H	-2.125490	3.067616	-0.825827
C	0.516017	4.618200	-0.329559
C	1.743442	4.465088	0.308253
C	1.790772	4.387788	1.706155
C	0.570348	4.480837	2.385867
C	-0.608953	4.647114	1.657745
H	0.469861	4.659358	-1.415641
H	2.652223	4.390421	-0.278627
H	0.534920	4.429026	3.470228
H	-1.561839	4.732298	2.176695
C	3.101028	4.200395	2.423153
H	3.795172	5.012735	2.179429
H	2.969237	4.172898	3.507863
H	3.576121	3.264479	2.111575
O	0.108702	1.629132	-0.664191
N	-0.656377	4.713148	0.319060
N	-5.600985	0.740719	1.101593
H	-5.658726	-0.196856	0.706366
C	-5.361356	0.607587	2.538463
H	-5.325973	1.600718	3.001644
H	-4.417996	0.092944	2.787934
H	-6.185838	0.051953	2.997227

VIb

C	-1.193345	-0.798370	-3.190850
C	-0.785088	-2.030851	-2.610802
C	0.636401	-2.092522	-2.652097
C	1.106861	-0.901357	-3.322957
C	-0.015912	-0.102783	-3.634800
H	-2.209743	-0.455942	-3.306413
H	-1.442380	-2.783372	-2.200287
H	1.246375	-2.910520	-2.296970
H	2.137986	-0.662730	-3.542929
H	0.000028	0.869710	-4.108705
Ru	0.097952	-0.375173	-1.356413
P	2.363578	-0.179641	-0.487627
P	-0.866874	-1.346252	0.609928
C	3.164031	1.367032	-1.098254
C	2.690995	2.052238	-2.225701
C	4.332033	1.821396	-0.462241
C	3.381409	3.161463	-2.718934
H	1.778663	1.735769	-2.712149
C	5.018728	2.930912	-0.956436
H	4.713271	1.307185	0.413957
C	4.547983	3.600942	-2.089138
H	3.000229	3.685513	-3.590516
H	5.920278	3.270225	-0.455054
H	5.082509	4.465055	-2.472653
C	3.493562	-1.505525	-1.077648

C	4.469131	-1.258890	-2.055469
C	3.335409	-2.810155	-0.582603
C	5.268727	-2.302888	-2.528148
H	4.610213	-0.259293	-2.451647
C	4.141624	-3.846789	-1.050431
H	2.589592	-3.014375	0.172392
C	5.108654	-3.596505	-2.028296
H	6.019395	-2.099404	-3.286178
H	4.009487	-4.847496	-0.649801
H	5.734216	-4.404471	-2.396310
C	2.637860	-0.069104	1.323554
C	3.607384	-0.820383	2.004525
C	1.856956	0.853559	2.034357
C	3.762044	-0.673551	3.384288
H	4.236373	-1.524327	1.473108
C	2.006336	0.987440	3.414034
H	1.140244	1.469649	1.508195
C	2.953871	0.218206	4.093952
H	4.511724	-1.263538	3.903436
H	1.378955	1.691584	3.951251
H	3.069075	0.320178	5.169138
C	0.143733	-2.779410	1.181901
C	0.963038	-2.716094	2.317825
C	0.132670	-3.958907	0.414559
C	1.743741	-3.815431	2.686021
H	1.000831	-1.815655	2.918861
C	0.907099	-5.056048	0.789220
H	-0.491557	-4.029483	-0.469515
C	1.715896	-4.987230	1.928484
H	2.376728	-3.746627	3.565493
H	0.881228	-5.961724	0.190510
H	2.321090	-5.840687	2.219651
C	-2.514820	-2.140178	0.291526
C	-3.393215	-1.567313	-0.634764
C	-2.941622	-3.262366	1.023376
C	-4.668043	-2.101521	-0.838293
H	-3.090249	-0.695881	-1.196717
C	-4.215251	-3.794912	0.821459
H	-2.284657	-3.732667	1.746145
C	-5.082736	-3.218154	-0.110396
H	-5.330646	-1.633746	-1.559570
H	-4.527463	-4.664114	1.393164
H	-6.072776	-3.636526	-0.266671
C	-1.269540	-0.413518	2.160078
C	-1.608178	-1.129640	3.323083
C	-1.347944	0.985952	2.185705
C	-1.990077	-0.461395	4.485297
H	-1.571420	-2.212862	3.332170
C	-1.737324	1.651991	3.350646
H	-1.099479	1.568645	1.311559
C	-2.054215	0.934561	4.503231
H	-2.239990	-1.033305	5.374186
H	-1.789957	2.734855	3.349105
H	-2.354917	1.456221	5.407197
C	-3.614014	1.648455	-3.270865
C	-3.485787	1.683695	-1.873041
C	-4.632202	1.455487	-1.075276
C	-5.859537	1.203326	-1.700007
C	-5.967169	1.154908	-3.092202
C	-4.838155	1.370859	-3.883351
H	-2.737297	1.852299	-3.879746
H	-6.734666	1.032431	-1.081354

H	-6.929978	0.952914	-3.553705
H	-4.909803	1.340957	-4.967015
C	-2.194358	2.062757	-1.251367
C	-1.053799	1.376850	-1.103164
C	-4.499823	1.435834	0.433776
H	-4.462235	2.467058	0.817108
H	-3.521375	0.998324	0.684347
H	-2.125490	3.067616	-0.825827
C	0.516017	4.618200	-0.329559
C	1.743442	4.465088	0.308253
C	1.790772	4.387788	1.706155
C	0.570348	4.480837	2.385867
C	-0.608953	4.647114	1.657745
H	0.469861	4.659358	-1.415641
H	2.652223	4.390421	-0.278627
H	0.534920	4.429026	3.470228
H	-1.561839	4.732298	2.176695
C	3.101028	4.200395	2.423153
H	3.795172	5.012735	2.179429
H	2.969237	4.172898	3.507863
H	3.576121	3.264479	2.111575
O	0.108702	1.629132	-0.664191
N	-0.656377	4.713148	0.319060
N	-5.600985	0.740719	1.101593
H	-5.658726	-0.196856	0.706366
C	-5.361356	0.607587	2.538463
H	-5.325973	1.600718	3.001644
H	-4.417996	0.092944	2.787934
H	-6.185838	0.051953	2.997227

VIIb

C	-1.726553	-1.515205	0.430413
C	-0.484458	-0.981419	0.033792
C	-0.442240	0.335116	-0.490904
C	-1.637902	1.058296	-0.585353
C	-2.863177	0.522853	-0.184359
C	-2.902104	-0.775745	0.325883
H	-1.761807	-2.527308	0.825066
H	-1.600249	2.066506	-0.991165
H	-3.772453	1.110277	-0.272573
H	-3.844083	-1.216448	0.640354
C	0.684524	-1.860231	0.191467
C	1.965217	-1.615951	-0.021805
C	0.858442	0.998337	-0.913473
H	0.625394	1.826366	-1.592551
H	1.470051	0.295495	-1.492504
N	1.694056	1.545770	0.167929
C	1.040957	2.604245	0.937359
H	0.828758	3.454594	0.278069
H	1.722065	2.949513	1.721794
H	1.966150	0.802374	0.806672
O	3.118052	-1.454769	-0.174959
H	0.515454	-2.875277	0.549115
H	0.091684	2.309878	1.413780

g. Coordinates for Metal-free lactamization of VIIb. VIIb to 2b.

$TS_{VIIb\text{-}IXb}$

C	-1.808099	1.436840	0.279221

C	-0.604098	0.791867	-0.053259
C	-0.649472	-0.591038	-0.373286
C	-1.855441	-1.286411	-0.323520
C	-3.044904	-0.630396	0.014329
C	-3.014389	0.734893	0.309728
H	-1.789782	2.497078	0.519387
H	-1.867994	-2.347286	-0.563199
H	-3.981032	-1.180186	0.044917
H	-3.931730	1.255392	0.572155
C	0.673654	1.502812	-0.114856
C	1.814904	0.779078	-0.154473
C	0.639491	-1.255885	-0.822734
H	0.833107	-1.035860	-1.879773
H	0.611072	-2.340198	-0.693497
N	1.776252	-0.699738	-0.049122
C	1.815418	-1.178357	1.356648
H	0.870513	-0.974013	1.866964
H	2.632157	-0.669874	1.872099
H	2.963567	-0.321137	-0.432151
O	3.090178	0.982849	-0.355050
H	0.697783	2.585102	-0.179282
H	2.004765	-2.254073	1.349842

IXb

C	-1.841753	1.455733	0.206106
C	-0.661784	0.774876	-0.136593
C	-0.719103	-0.623348	-0.345506
C	-1.914835	-1.314065	-0.165829
C	-3.084189	-0.626876	0.183407
C	-3.042879	0.759467	0.358790
H	-1.810148	2.530826	0.366079
H	-1.939560	-2.390577	-0.320724
H	-4.018109	-1.167760	0.307378
H	-3.947445	1.300418	0.624376
C	0.636185	1.431729	-0.244391
C	1.765020	0.686569	-0.164998
C	0.557631	-1.264013	-0.844242
H	0.648620	-1.074580	-1.923536
H	0.552204	-2.347217	-0.700044
N	1.765762	-0.714006	-0.204031
C	2.159336	-1.368781	1.056733
H	1.366316	-1.318670	1.816556
H	3.051313	-0.882222	1.456810
H	3.657524	0.639891	-0.294771
O	2.981656	1.268043	0.011715
H	0.706481	2.513483	-0.278799
H	2.397449	-2.417644	0.860304

IX’b

C	2.020798	1.385362	-0.008119
C	0.777181	0.737169	0.116757
C	0.753036	-0.678848	0.116633
C	1.932771	-1.403739	-0.033107
C	3.163567	-0.747647	-0.159371
C	3.199570	0.649856	-0.137209
H	2.053810	2.472395	-0.011000
H	1.893418	-2.491038	-0.037313
H	4.079688	-1.320909	-0.268119
H	4.149320	1.170511	-0.230737
C	-0.483969	1.444010	0.204780
C	-1.650641	0.762474	-0.004595
C	-0.573334	-1.353163	0.382852

H	-0.730493	-1.457847	1.474544
H	-0.586714	-2.361859	-0.040147
N	-1.690637	-0.599950	-0.201769
C	-2.968033	-1.302276	-0.198990
H	-2.827944	-2.279149	-0.670138
H	-3.711263	-0.745482	-0.769033
H	-2.737090	2.320624	-0.091747
O	-2.864656	1.358690	-0.091670
H	-0.505317	2.525433	0.290669
H	-3.351387	-1.461740	0.820981

$TS_{IX'b\text{-}2b}$

C	2.008520	1.361931	-0.052736
C	0.763426	0.743671	0.147589
C	0.708736	-0.669471	0.169842
C	1.867881	-1.422269	-0.034456
C	3.099419	-0.792639	-0.233690
C	3.166087	0.604039	-0.232810
H	2.059329	2.447770	-0.073239
H	1.809927	-2.508528	-0.021587
H	3.996115	-1.386212	-0.387028
H	4.119508	1.103505	-0.384302
C	-0.498554	1.492792	0.319933
C	-1.703207	0.725368	0.091793
C	-0.600304	-1.365511	0.500912
H	-0.678268	-1.544208	1.583083
H	-0.648026	-2.341260	0.010555
N	-1.776973	-0.594334	0.057738
C	-2.967438	-1.307462	-0.390195
H	-2.691651	-2.044356	-1.151005
H	-3.672649	-0.589884	-0.810528

H	-1.624037	2.245462	-0.373534
O	-2.661826	1.534065	-0.273241
H	-0.563133	2.108396	1.221899
H	-3.441088	-1.825559	0.450366

$TS_{VIIb\text{-}2b}$

C	-1.988792	-1.337478	0.272201
C	-0.739537	-0.713771	0.119910
C	-0.707952	0.674129	-0.149575
C	-1.894836	1.394462	-0.273592
C	-3.133428	0.759206	-0.136816
C	-3.172644	-0.609515	0.140892
H	-2.025617	-2.402129	0.488857
H	-1.850004	2.462013	-0.477977
H	-4.053019	1.327507	-0.240850
H	-4.127926	-1.114929	0.255851
C	0.528581	-1.469436	0.277812
C	1.731809	-0.890025	-0.266524
C	0.642780	1.348337	-0.291247
H	0.642762	2.327761	0.196113
H	0.913724	1.505641	-1.348725
N	1.674618	0.511683	0.367386
C	2.970932	1.160510	0.561217
H	3.420075	1.434617	-0.400859
H	3.643104	0.477533	1.083520
H	1.103118	-0.425672	1.168947
O	2.738238	-1.322378	-0.782795
H	0.454321	-2.554320	0.276122
H	2.829756	2.060708	1.163995

VIIb-H_2O

C	-2.253039	-1.397385	-0.197619
C	-1.055728	-0.671537	-0.375233
C	-1.086809	0.720044	-0.117158
C	-2.250563	1.343897	0.327564
C	-3.426135	0.607637	0.507707
C	-3.418029	-0.764842	0.233286
H	-2.255827	-2.466925	-0.393723
H	-2.240140	2.414205	0.521884
H	-4.332045	1.097084	0.852515
H	-4.325911	-1.348392	0.364341
C	0.180885	-1.305942	-0.775409
C	1.428575	-0.748088	-0.639500
C	0.152463	1.496790	-0.446717
H	0.178037	2.471017	0.047149
H	0.230796	1.653968	-1.530167
N	1.391199	0.747634	-0.067508
C	2.616358	1.505908	-0.439443
H	2.609305	1.676528	-1.517008
H	3.491446	0.921660	-0.168064
H	1.435438	0.592471	0.952539
O	2.580863	-1.198249	-0.769126
H	0.163961	-2.349799	-1.073543
H	2.611099	2.457344	0.094487
O	2.704253	-0.642392	2.047705
H	2.968620	-1.036801	1.194434
H	2.118043	-1.309566	2.434205

$TS_{VIIb\text{-}IXb}$-H_2O

C	-2.330822	1.382797	0.095124
C	-1.080769	0.774328	-0.118793
C	-1.040312	-0.627334	-0.312914
C	-2.207765	-1.385211	-0.260376
C	-3.444105	-0.767534	-0.040541
C	-3.498446	0.619388	0.129898
H	-2.378427	2.459022	0.242028
H	-2.152416	-2.461908	-0.404298
H	-4.352489	-1.361865	-0.006172
H	-4.454422	1.108345	0.298326
C	0.165434	1.526171	-0.146789
C	1.371719	0.913269	-0.030030
C	0.304420	-1.221931	-0.645874
H	0.539709	-1.055717	-1.705193
H	0.343001	-2.296625	-0.452158
N	1.396007	-0.572495	0.125430
C	1.405037	-0.941918	1.568981
H	0.457399	-0.675917	2.043530
H	2.223173	-0.405741	2.052356
H	2.552110	-0.819139	-0.292537
O	2.556810	1.458323	0.012683
H	0.141363	2.608660	-0.218381
H	1.575082	-2.017636	1.653657
O	3.765955	-0.545396	-0.491551
H	3.334405	0.586822	-0.248400
H	3.950996	-0.596402	-1.441936

IX’b-H_2O

C	-2.396012	1.376566	0.042106
C	-1.147212	0.764204	-0.155478
C	-1.094264	-0.641772	-0.299935
C	-2.255786	-1.404267	-0.205905
C	-3.494869	-0.784668	-0.002128
C	-3.559262	0.607166	0.113111

H	-2.448547	2.457035	0.152308
H	-2.196221	-2.485406	-0.310491
H	-4.399909	-1.382429	0.058042
H	-4.518000	1.095480	0.266941
C	0.107361	1.507396	-0.182134
C	1.282602	0.864680	0.016855
C	0.262743	-1.216336	-0.635448
H	0.457569	-1.068265	-1.706764
H	0.312717	-2.289769	-0.435512
N	1.365455	-0.557075	0.092975
C	1.545586	-1.034704	1.482922
H	0.645067	-0.879455	2.091994
H	2.376853	-0.493749	1.939379
H	3.182211	-0.862194	-0.570801
O	2.438787	1.525093	0.227276
H	0.100201	2.589350	-0.262682
H	1.789204	-2.100130	1.466006
O	4.081402	-0.463168	-0.583421
H	3.192065	0.925974	-0.010937
H	4.281784	-0.331912	-1.522939

IXb-H_2O

C	1.759001	-1.739024	-0.011736
C	0.845018	-0.672873	-0.129583
C	1.353257	0.649543	-0.109460
C	2.716894	0.880491	0.052789
C	3.614519	-0.188182	0.172157
C	3.125939	-1.497837	0.130057
H	1.385438	-2.760527	-0.024726
H	3.085184	1.904238	0.072063
H	4.677533	0.000805	0.290804
H	3.812378	-2.336514	0.217418
C	-0.585428	-0.858126	-0.232537
C	-1.426886	0.204666	-0.014995
C	0.374333	1.770319	-0.370388
H	0.275412	1.937135	-1.461783
H	0.734903	2.707941	0.063164
N	-0.945740	1.480883	0.202027
C	-1.865925	2.610589	0.204003
H	-1.376813	3.458126	0.692847
H	-2.770889	2.363053	0.757706
H	-3.041200	-0.843613	0.050126
O	-2.763792	0.107660	0.059815
H	-1.003285	-1.852596	-0.327950
H	-2.149823	2.917219	-0.815335
O	-3.509275	-2.475108	0.055353
H	-3.242700	-2.932809	-0.757210
H	-3.045760	-2.946452	0.765509

$TS_{IXb\text{-}2b}$-H_2O

C	-1.915820	1.495001	-0.184923
C	-0.852471	0.576814	-0.241526
C	-1.137070	-0.787857	-0.049715
C	-2.443563	-1.208001	0.217969
C	-3.490282	-0.285842	0.270171
C	-3.221302	1.070701	0.057989
H	-1.705264	2.552244	-0.328499
H	-2.643067	-2.266482	0.371268
H	-4.503368	-0.621041	0.473187
H	-4.027979	1.798218	0.094197
C	0.540334	1.017459	-0.462993
C	1.581298	0.028107	-0.288010

C	-0.034938	-1.809957	-0.212215
H	-0.077565	-2.268032	-1.213012
H	-0.165506	-2.623463	0.508792
N	1.310289	-1.253080	-0.005647
C	2.359241	-2.214707	0.316757
H	2.059336	-2.797908	1.193247
H	3.287383	-1.687773	0.528559
H	2.765931	1.502720	0.117307
O	2.825790	0.420824	-0.269073
H	0.694869	1.617038	-1.364623
H	2.518633	-2.902390	-0.522574
O	2.266130	2.561566	0.674169
H	2.351267	3.308189	0.060913
H	1.225385	2.010808	0.338972

2b

C	-1.991918	1.351884	-0.074137
C	-0.774541	0.720548	0.200298
C	-0.711391	-0.678543	0.227069
C	-1.862469	-1.435742	-0.008151
C	-3.079776	-0.801248	-0.265885
C	-3.143177	0.594719	-0.302086
H	-2.035961	2.437614	-0.107674
H	-1.804226	-2.521359	0.005334
H	-3.971711	-1.393542	-0.449620
H	-4.085425	1.092579	-0.513489
C	0.491599	1.487258	0.484161
C	1.761680	0.811529	-0.023882
C	0.621769	-1.322210	0.518192
N	1.741252	-0.552238	-0.036231
C	2.946068	-1.301202	-0.363282
H	3.349640	-1.799472	0.527629
H	3.692705	-0.610743	-0.753576
H	0.464843	2.494324	0.062381
O	2.737050	1.479625	-0.376231
H	0.618116	1.603133	1.571778
H	2.723431	-2.064171	-1.117179
H	0.758437	-1.448088	1.604421
H	0.657923	-2.323570	0.079687

h. Coordinates for Metal-bound lactamization of VIb. VIb to XVb.

$TS_{VIb\text{-}XIVb}$

C	0.912462	-1.729466	-2.590041
C	0.349704	-0.478651	-2.966684
C	-1.076338	-0.577556	-2.897306
C	-1.395388	-1.935787	-2.523740
C	-0.177280	-2.623091	-2.303545
H	1.963763	-1.967876	-2.564192
H	0.911051	0.398481	-3.255303
H	-1.782869	0.196699	-3.160105
H	-2.390127	-2.344143	-2.408952
H	-0.078981	-3.647234	-1.965427
Ru	-0.313856	-0.857415	-0.877803
P	-2.450748	-0.592537	0.190657
P	0.668386	1.214964	-0.229471
C	-3.178156	-2.215231	0.711528
C	-2.597851	-3.441374	0.362730

C	-4.373773	-2.217433	1.454065
C	-3.197581	-4.645102	0.744809
H	-1.672454	-3.465587	-0.196163
C	-4.973588	-3.418150	1.828891
H	-4.840234	-1.278501	1.735589
C	-4.385352	-4.636883	1.475969
H	-2.730152	-5.586755	0.471339
H	-5.898599	-3.402160	2.397846
H	-4.851060	-5.572476	1.771990
C	-3.723699	0.067075	-0.972708
C	-4.726440	-0.759604	-1.505080
C	-3.632438	1.393459	-1.425419
C	-5.623032	-0.261642	-2.454186
H	-4.816700	-1.791881	-1.187496
C	-4.536603	1.891070	-2.363319
H	-2.847040	2.038736	-1.060479
C	-5.535679	1.064093	-2.882604
H	-6.391635	-0.915889	-2.855613
H	-4.448188	2.922493	-2.691384
H	-6.237085	1.448454	-3.617454
C	-2.635780	0.351243	1.770756
C	-3.160923	1.648037	1.849928
C	-2.209466	-0.279224	2.954738
C	-3.234821	2.307791	3.079945
H	-3.524370	2.154437	0.965908
C	-2.288237	0.380005	4.179786
H	-1.822360	-1.292669	2.923934
C	-2.794845	1.680929	4.245772
H	-3.643658	3.313344	3.120194
H	-1.955143	-0.124402	5.082108
C	-0.378679	2.676444	-0.641757
C	-0.952988	3.481364	0.351093
C	-0.648001	2.964540	-1.992753
C	-1.789139	4.546060	0.000896
H	-0.760598	3.279297	1.398080
C	-1.465814	4.038572	-2.339399
H	-0.212811	2.354730	-2.776993
C	-2.045780	4.829190	-1.341168
H	-2.235725	5.153417	0.782863
H	-1.657791	4.252294	-3.386765
H	-2.690927	5.660355	-1.610342
C	2.224516	1.574050	-1.175800
C	3.126756	0.525623	-1.401235
C	2.552014	2.858297	-1.640518
C	4.318324	0.743227	-2.093606
H	2.900707	-0.464966	-1.034188
C	3.747625	3.077124	-2.329783
H	1.880058	3.692632	-1.474325
C	4.630826	2.021031	-2.564018
H	4.999541	-0.086918	-2.253372
H	3.984114	4.076176	-2.684914
H	5.556802	2.193729	-3.105100
C	1.217560	1.550784	1.501114
C	2.009392	2.676956	1.788936
C	0.888824	0.672642	2.537691
C	2.460965	2.909391	3.086687
H	2.281509	3.370980	1.000878
C	1.345128	0.902461	3.838400
H	0.268194	-0.187131	2.337974
C	2.134695	2.018363	4.115621
H	3.071657	3.783213	3.294703
H	1.077941	0.209117	4.630495

H	2.492137	2.198088	5.125526
C	3.634862	-3.134753	-0.915428
C	3.711930	-2.443473	0.304707
C	4.960818	-1.935290	0.736057
C	6.075091	-2.103997	-0.092407
C	5.981692	-2.753588	-1.326548
C	4.753226	-3.273646	-1.737787
H	2.688268	-3.582232	-1.202945
H	7.033029	-1.709221	0.236729
H	6.862585	-2.862829	-1.952478
H	4.667393	-3.804061	-2.681897
C	2.516640	-2.300023	1.145698
C	1.251000	-2.091118	0.834140
C	5.098939	-1.171634	2.040551
H	6.165198	-1.154096	2.326721
H	4.571631	-1.699055	2.842660
H	2.620889	-2.353205	2.231947
O	0.052593	-2.065718	0.993440
N	4.526971	0.173410	1.933878
H	4.247147	0.502692	2.852900
C	5.427641	1.141485	1.319252
H	5.650680	0.838623	0.292284
H	6.389666	1.255112	1.851931
H	4.937052	2.118087	1.274723
H	-2.854706	2.196287	5.199895

XIVb

C	-1.100417	1.064975	-2.781778
C	-0.450348	-0.165520	-3.041537
C	0.971840	0.057017	-3.049504
C	1.179976	1.473177	-2.871614
C	-0.083220	2.084651	-2.680200
H	-2.166284	1.215540	-2.678823
H	-0.945733	-1.117197	-3.174213
H	1.734256	-0.681447	-3.251794
H	2.139897	1.971880	-2.852252
H	-0.260612	3.136107	-2.495647
Ru	0.253237	0.561655	-1.058326
P	2.289031	0.931100	0.097971
P	-0.387772	-1.513404	-0.120177
C	2.644300	2.721558	0.405117
C	1.775852	3.714264	-0.067017
C	3.793292	3.110403	1.119307
C	2.052497	5.067163	0.152868
H	0.877764	3.430320	-0.600319
C	4.071075	4.459355	1.334431
H	4.473054	2.357295	1.505522
C	3.201381	5.442157	0.849727
H	1.367367	5.823408	-0.219855
H	4.965014	4.743440	1.882237
H	3.418641	6.492779	1.019874
C	3.715187	0.362138	-0.940963
C	4.735496	1.220235	-1.379051
C	3.736481	-0.978854	-1.362769
C	5.759897	0.741013	-2.201543
H	4.741000	2.265119	-1.092361
C	4.767973	-1.458238	-2.170033
H	2.939731	-1.650510	-1.071718
C	5.784646	-0.598180	-2.593893
H	6.539344	1.421309	-2.533271
H	4.765324	-2.501125	-2.473147
H	6.584612	-0.967306	-3.229397

C	2.641464	0.236685	1.779518
C	3.281752	-0.996719	1.964290
C	2.200126	0.945085	2.911381
C	3.463687	-1.515780	3.249088
H	3.648296	-1.560934	1.115956
C	2.391965	0.429955	4.192474
H	1.704479	1.903901	2.795414
C	3.019688	-0.806566	4.366034
H	3.960864	-2.474013	3.371635
H	2.046824	0.993894	5.054316
C	0.784232	-2.881559	-0.516768
C	1.601550	-3.450132	0.471814
C	0.966491	-3.276755	-1.854767
C	2.588628	-4.378027	0.128966
H	1.486934	-3.161172	1.509423
C	1.944138	-4.212117	-2.192382
H	0.346303	-2.857560	-2.638873
C	2.765112	-4.760258	-1.201552
H	3.218986	-4.798507	0.907313
H	2.067221	-4.508790	-3.229995
H	3.532787	-5.481456	-1.466315
C	-2.009152	-2.070481	-0.828875
C	-3.032218	-1.114582	-0.938272
C	-2.275426	-3.392302	-1.214915
C	-4.291160	-1.464889	-1.425445
H	-2.853723	-0.092417	-0.630036
C	-3.535289	-3.741791	-1.711143
H	-1.506147	-4.152996	-1.136770
C	-4.544066	-2.782034	-1.818931
H	-5.067506	-0.708111	-1.492924
H	-3.725539	-4.767948	-2.012484

H	-5.521454	-3.058205	-2.204392
C	-0.719918	-1.749001	1.679413
C	-1.158559	-2.997556	2.158478
C	-0.590296	-0.683856	2.574771
C	-1.449157	-3.171780	3.509986
H	-1.272379	-3.832858	1.474394
C	-0.887856	-0.858078	3.930199
H	-0.261543	0.281852	2.220253
C	-1.316526	-2.099272	4.399747
H	-1.783304	-4.140954	3.868922
H	-0.777924	-0.022009	4.614390
H	-1.546848	-2.234754	5.452625
C	-4.969933	2.447453	-1.691236
C	-4.379804	2.372744	-0.418710
C	-5.122270	1.865041	0.666585
C	-6.419530	1.394495	0.433465
C	-6.992631	1.438292	-0.840025
C	-6.266758	1.978644	-1.904173
H	-4.399548	2.864244	-2.517439
H	-6.984341	0.986863	1.268181
H	-7.997995	1.058214	-0.997834
H	-6.702383	2.031007	-2.898093
C	-2.980315	2.839654	-0.291716
C	-1.954511	2.225542	0.250083
C	-4.507317	1.792411	2.045596
H	-5.305141	1.670016	2.797178
H	-3.986917	2.731532	2.263898
H	-2.669878	3.725505	-0.850215
O	-0.901858	1.787986	0.588854
N	-3.516820	0.710241	2.100751
H	-2.886396	0.867234	2.882563

C	-4.138865	-0.607319	2.251859
H	-4.729765	-0.842854	1.362350
H	-4.810445	-0.663441	3.126412
H	-3.369064	-1.373016	2.356547
H	3.164329	-1.210501	5.363801

XVb

C	-0.924770	-0.304048	-3.109972
C	0.320267	-0.991397	-3.118016
C	1.383632	-0.032868	-3.049479
C	0.772797	1.270262	-3.046552
C	-0.635797	1.107975	-3.069832
H	-1.903538	-0.759873	-3.155579
H	0.438781	-2.065623	-3.154832
H	2.443107	-0.243722	-3.071282
H	1.300700	2.214194	-3.012907
H	-1.361447	1.909785	-3.071350
Ru	0.188728	0.138066	-1.201702
P	1.551595	1.628604	0.024480
P	0.490221	-1.871671	-0.015431
C	0.863423	3.347122	0.038730
C	-0.410762	3.595334	-0.490000
C	1.571438	4.410771	0.628566
C	-0.959534	4.880860	-0.450892
H	-0.979735	2.778359	-0.916081
C	1.027407	5.694182	0.659274
H	2.547151	4.233378	1.070773
C	-0.240110	5.932776	0.117320
H	-1.950107	5.054075	-0.862263
H	1.589383	6.506328	1.111496
H	-0.664230	6.932474	0.145760
C	3.208583	1.786363	-0.792241
C	3.676848	2.976365	-1.369167
C	3.991966	0.625561	-0.920715
C	4.909132	3.008243	-2.029779
H	3.086676	3.883685	-1.320599
C	5.227579	0.662696	-1.565447
H	3.626612	-0.320466	-0.541758
C	5.692680	1.857252	-2.122346
H	5.252253	3.939124	-2.472483
H	5.815365	-0.247515	-1.642834
H	6.650840	1.887051	-2.633114
C	1.955238	1.433010	1.828243
C	2.976557	0.575706	2.265085
C	1.151352	2.063080	2.796393
C	3.186248	0.350986	3.627158
H	3.617625	0.072393	1.553704
C	1.363901	1.838299	4.157403
H	0.351179	2.729391	2.493376
C	2.380519	0.978078	4.578891
H	3.983185	-0.317771	3.940021
H	0.733249	2.337962	4.887512
C	2.221738	-2.503758	0.054047
C	2.904376	-2.646189	1.271002
C	2.916936	-2.744447	-1.145398
C	4.255600	-3.003879	1.287709
H	2.391815	-2.462961	2.208577
C	4.260302	-3.118146	-1.125198
H	2.412723	-2.636081	-2.099349

C	4.937092	-3.239810	0.092808
H	4.771829	-3.098658	2.238839
H	4.779971	-3.305581	-2.060473
H	5.986588	-3.518944	0.108042
C	-0.467562	-3.219006	-0.854928
C	-1.823031	-2.959355	-1.126238
C	0.065232	-4.469043	-1.194628
C	-2.624193	-3.928609	-1.727652
H	-2.250518	-1.993486	-0.873954
C	-0.738222	-5.436297	-1.808983
H	1.103474	-4.699323	-0.981736
C	-2.081389	-5.170010	-2.076744
H	-3.670027	-3.713326	-1.929020
H	-0.309697	-6.399352	-2.071984
H	-2.703047	-5.922972	-2.552647
C	-0.114504	-2.112037	1.719312
C	-0.443339	-3.394050	2.194351
C	-0.255631	-1.018382	2.581947
C	-0.910764	-3.568771	3.497626
H	-0.342100	-4.260090	1.548678
C	-0.722072	-1.193237	3.886748
H	-0.022550	-0.028074	2.229009
C	-1.055170	-2.468271	4.347525
H	-1.162944	-4.565727	3.847767
H	-0.819436	-0.329650	4.538434
H	-1.422981	-2.605950	5.360403
C	-5.795630	0.891400	-1.821240
C	-4.912599	0.962763	-0.728193
C	-5.462137	1.130202	0.564201
C	-6.840921	1.194729	0.752388
C	-7.705879	1.119411	-0.343776
C	-7.174614	0.973124	-1.629450
H	-5.390253	0.766559	-2.822303
H	-7.238382	1.314922	1.757250
H	-8.779984	1.173664	-0.195062
H	-7.839385	0.915162	-2.487086
C	-3.469983	0.870344	-0.888721
C	-2.622719	0.646756	0.153943
C	-4.493051	1.309156	1.698440
H	-4.930818	1.084424	2.672889
H	-4.088098	2.327624	1.714196
H	-3.043266	0.954125	-1.878026
O	-1.360786	0.544226	0.272737
N	-3.303294	0.406168	1.506942
H	-2.593213	0.653409	2.204252
C	-3.630703	-1.047695	1.694389
H	-4.425340	-1.314751	0.999691
H	-3.957898	-1.195638	2.724018
H	-2.734223	-1.630064	1.495393
H	2.544214	0.800613	5.637825

i. Coordinates for Ru(II)-vinylidene hydroamination. IVb to 8b.

$TS_{IVb\text{-}VIIIb}$

C	-0.904205	-0.295676	-3.540995
C	-1.154879	-1.602978	-3.009749

C	0.082404	-2.225285	-2.730285
C	1.121084	-1.297853	-3.089113
C	0.511530	-0.116414	-3.599547
H	-1.642074	0.401968	-3.906712
H	-2.129132	-2.040405	-2.842855
H	0.226975	-3.217047	-2.326662
H	2.182412	-1.493231	-3.027511
H	1.025686	0.757948	-3.976389
Ru	-0.041711	-0.302495	-1.394862
P	1.801918	-0.929667	0.000563
P	-1.872745	-0.497420	0.141887
C	3.417181	-0.147638	-0.486540
C	3.577685	0.564335	-1.682843
C	4.537267	-0.321173	0.347809
C	4.819826	1.099264	-2.035218
H	2.730797	0.729364	-2.334913
C	5.777077	0.208256	-0.006363
H	4.445234	-0.875739	1.275732
C	5.921923	0.923406	-1.198801
H	4.918450	1.656697	-2.962092
H	6.629182	0.062800	0.650966
H	6.886978	1.340625	-1.471209
C	2.192798	-2.728333	-0.201212
C	3.352085	-3.170121	-0.857847
C	1.262808	-3.683191	0.237112
C	3.575151	-4.536091	-1.054537
H	4.084380	-2.461003	-1.224623
C	1.493993	-5.045612	0.053822
H	0.344733	-3.364910	0.708688
C	2.653317	-5.477989	-0.595653
H	4.475869	-4.859591	-1.568278
H	0.761778	-5.763055	0.412559
H	2.833772	-6.538357	-0.747056
C	1.854823	-0.620744	1.828738
C	1.592843	-1.614958	2.780148
C	2.171405	0.675589	2.282120
C	1.620731	-1.317933	4.146069
H	1.372128	-2.629204	2.477468
C	2.195627	0.969148	3.644021
H	2.401867	1.463328	1.576109
C	1.912665	-0.026920	4.583341
H	1.412799	-2.105509	4.864379
H	2.440325	1.976370	3.968329
H	1.931067	0.200976	5.645055
C	-1.814048	-1.979981	1.245143
C	-1.591908	-1.908055	2.624626
C	-2.031953	-3.237667	0.651158
C	-1.577369	-3.074775	3.396799
H	-1.429684	-0.951349	3.107348
C	-2.031444	-4.396333	1.424771
H	-2.208405	-3.308307	-0.418698
C	-1.796645	-4.317513	2.802723
H	-1.396330	-3.004066	4.465535
H	-2.207597	-5.358865	0.953254
H	-1.790169	-5.220041	3.406891
C	-3.566313	-0.764168	-0.586313
C	-3.902708	-0.322159	-1.870648
C	-4.572722	-1.358863	0.199105
C	-5.196869	-0.480735	-2.372415
H	-3.156347	0.154715	-2.484407
C	-5.865842	-1.516071	-0.299303
H	-4.354331	-1.694781	1.206629

C	-6.182223	-1.082257	-1.589647
H	-5.428263	-0.133471	-3.375331
H	-6.626089	-1.978317	0.323784
H	-7.188368	-1.210563	-1.978177
C	-2.180860	0.921539	1.282956
C	-3.440383	1.523650	1.431077
C	-1.101482	1.423039	2.024468
C	-3.608666	2.597905	2.309336
H	-4.293443	1.170821	0.864204
C	-1.273136	2.486344	2.909891
H	-0.123233	0.981266	1.911644
C	-2.529724	3.079941	3.053692
H	-4.588113	3.057115	2.407815
H	-0.421387	2.851112	3.476588
H	-2.665892	3.914586	3.735313
C	2.265936	4.554479	0.075038
C	1.042025	3.931576	-0.255259
C	-0.139231	4.704005	-0.198684
C	-0.061411	6.048351	0.182521
C	1.153691	6.647432	0.517833
C	2.326751	5.891145	0.458066
H	3.179888	3.966720	0.029770
H	-0.977375	6.633500	0.215327
H	1.184135	7.692290	0.813689
H	3.284658	6.339719	0.707171
C	1.107735	2.504770	-0.580842
C	0.321482	1.504323	-0.969032
H	2.109899	2.099146	-0.427372
C	-1.483160	4.108392	-0.521943
H	-2.199155	4.922657	-0.723080
H	-1.870166	3.558145	0.342756
N	-1.409751	3.173901	-1.643989
C	-2.733735	2.809435	-2.131898
H	-3.271621	2.263776	-1.354197
H	-2.644313	2.172165	-3.012096
H	-0.874355	3.595517	-2.400058
H	-3.336809	3.690248	-2.406341

VIIIb

C	0.986183	-0.091378	-3.575111
C	1.849257	0.861627	-2.943457
C	1.089133	2.005076	-2.598934
C	-0.267110	1.757326	-3.007689
C	-0.329697	0.472934	-3.610506
H	1.292578	-1.006311	-4.060268
H	2.910293	0.738814	-2.774337
H	1.460119	2.905973	-2.134438
H	-1.087745	2.454171	-2.910221
H	-1.208863	0.014104	-4.043894
Ru	0.258665	0.210016	-1.418440
P	-1.034708	1.647851	0.032015
P	1.881615	-0.625450	0.077973
C	-2.818518	1.871162	-0.466644
C	-3.320940	1.314185	-1.650634
C	-3.682923	2.643252	0.330648
C	-4.650439	1.516414	-2.029121
H	-2.680389	0.695860	-2.266262
C	-5.010075	2.847248	-0.046764
H	-3.318670	3.091831	1.249152
C	-5.498816	2.282481	-1.228320
H	-5.021237	1.068022	-2.946514
H	-5.661080	3.446995	0.582807

H	-6.533538	2.438020	-1.520080
C	-0.426025	3.404385	-0.098809
C	-1.183353	4.425387	-0.694559
C	0.875953	3.709390	0.329961
C	-0.658082	5.714236	-0.832463
H	-2.183134	4.230158	-1.062190
C	1.394187	4.997503	0.207493
H	1.507612	2.931684	0.732959
C	0.626821	6.009239	-0.375664
H	-1.261942	6.486957	-1.300008
H	2.402794	5.200258	0.556639
H	1.029091	7.012771	-0.480441
C	-1.249210	1.414846	1.873690
C	-0.312945	1.916561	2.790607
C	-2.320102	0.650981	2.379727
C	-0.421926	1.641396	4.155815
H	0.515470	2.525125	2.459189
C	-2.426385	0.373660	3.742384
H	-3.091566	0.271625	1.722054
C	-1.471289	0.860290	4.638291
H	0.323089	2.040684	4.837942
H	-3.262483	-0.220194	4.100566
H	-1.551715	0.641437	5.699077
C	2.705037	0.636822	1.160235
C	2.632610	0.645810	2.557484
C	3.487493	1.611247	0.513290
C	3.310550	1.623920	3.293755
H	2.050376	-0.102582	3.082008
C	4.173263	2.576314	1.248099
H	3.555744	1.615655	-0.571140
C	4.078815	2.590323	2.644461

H	3.238371	1.621647	4.377680
H	4.774390	3.320051	0.732875
H	4.606659	3.345871	3.219134
C	3.454383	-1.357337	-0.623647
C	3.576411	-1.777717	-1.952631
C	4.562384	-1.527608	0.229478
C	4.763768	-2.344845	-2.425609
H	2.747275	-1.661379	-2.631722
C	5.745448	-2.098464	-0.236959
H	4.501372	-1.218086	1.267567
C	5.852229	-2.507699	-1.569613
H	4.832004	-2.655408	-3.464439
H	6.584648	-2.221232	0.441796
H	6.776063	-2.947172	-1.934597
C	1.365105	-1.962392	1.254525
C	1.970760	-3.228747	1.291643
C	0.261075	-1.724802	2.089247
C	1.483035	-4.225866	2.142213
H	2.819018	-3.453594	0.655826
C	-0.217187	-2.714928	2.946869
H	-0.232540	-0.764387	2.067634
C	0.389756	-3.973371	2.973183
H	1.962195	-5.200801	2.151196
H	-1.070833	-2.500286	3.582962
H	0.012563	-4.749800	3.632569
C	-4.431450	-2.362313	0.254196
C	-3.096435	-2.555260	-0.139223
C	-2.611146	-3.866236	-0.279680
C	-3.434249	-4.963721	-0.037788
C	-4.761504	-4.762078	0.355098
C	-5.255667	-3.460669	0.499756

H	-4.815411	-1.350717	0.361497
H	-3.040252	-5.970650	-0.150451
H	-5.406349	-5.615004	0.544715
H	-6.287014	-3.303781	0.803259
C	-2.184715	-1.441120	-0.374890
C	-0.995168	-1.444197	-1.025083
H	-2.533820	-0.499068	0.006954
C	-1.162946	-3.982379	-0.637067
H	-0.929896	-4.942745	-1.100738
H	-0.518166	-3.844845	0.235651
N	-0.775466	-2.895868	-1.610792
C	0.559277	-3.220013	-2.174438
H	1.279915	-3.279809	-1.363193
H	0.836370	-2.446971	-2.875844
H	-1.437068	-2.950053	-2.394302
H	0.491340	-4.183748	-2.682613

VIIIb-HCO_3^-

C	0.333828	-0.698762	-3.442618
C	1.541267	0.027021	-3.182814
C	1.205268	1.367456	-2.879961
C	-0.230414	1.470697	-2.936777
C	-0.765911	0.203736	-3.289842
H	0.271019	-1.703377	-3.832566
H	2.543942	-0.376272	-3.229724
H	1.896736	2.168853	-2.671182
H	-0.803588	2.373691	-2.777211
H	-1.815180	-0.026979	-3.432028
Ru	0.286699	0.023312	-1.256959
P	-0.180956	1.931216	0.144638
P	1.922117	-1.100876	0.001717
C	-1.946085	2.515689	0.081355
C	-2.854356	1.921939	-0.803416
C	-2.390037	3.583548	0.883893
C	-4.175190	2.369059	-0.886622
H	-2.547705	1.092954	-1.424701
C	-3.709084	4.030600	0.805198
H	-1.704671	4.070555	1.570323
C	-4.606271	3.422010	-0.079349
H	-4.839856	1.868651	-1.582862
H	-4.034517	4.854844	1.434014
H	-5.634253	3.769898	-0.135583
C	0.713089	3.450207	-0.472759
C	0.040427	4.566512	-0.993807
C	2.117479	3.459133	-0.489109
C	0.753325	5.663986	-1.487687
H	-1.041649	4.592188	-1.028052
C	2.829304	4.559273	-0.964519
H	2.666127	2.586510	-0.163921
C	2.148067	5.671490	-1.466335
H	0.208601	6.513799	-1.889635
H	3.915656	4.536294	-0.955351
H	2.698287	6.527765	-1.845929
C	0.152268	2.020597	1.984895
C	1.449851	2.248092	2.471297
C	-0.863842	1.765501	2.926783
C	1.726806	2.204776	3.839181
H	2.267321	2.449829	1.794863
C	-0.586227	1.716695	4.293358
H	-1.886674	1.609323	2.607313
C	0.713375	1.930743	4.757612
H	2.743917	2.376989	4.178711

H	-1.392441	1.514274	4.992750
H	0.930735	1.890229	5.821038
C	3.320381	-0.060853	0.646397
C	3.730889	-0.017803	1.984538
C	4.051769	0.665960	-0.309218
C	4.832773	0.756511	2.362766
H	3.197641	-0.581780	2.740676
C	5.157176	1.428546	0.066210
H	3.750286	0.638134	-1.352506
C	5.546124	1.483336	1.408816
H	5.132309	0.785074	3.406681
H	5.709258	1.983726	-0.687009
H	6.402429	2.082374	1.705063
C	2.989242	-2.379869	-0.850717
C	2.664334	-2.929175	-2.095522
C	4.152456	-2.840664	-0.204536
C	3.472604	-3.904956	-2.687570
H	1.778356	-2.598699	-2.612789
C	4.957621	-3.816797	-0.789252
H	4.431917	-2.438900	0.763950
C	4.621560	-4.352359	-2.036656
H	3.198630	-4.310241	-3.657687
H	5.849277	-4.157741	-0.270544
H	5.251584	-5.109689	-2.494480
C	1.351159	-2.066340	1.476814
C	1.382562	-3.469028	1.511355
C	0.748172	-1.384968	2.546037
C	0.820019	-4.168331	2.583697
H	1.842804	-4.028269	0.704696
C	0.201488	-2.081152	3.623341
H	0.699313	-0.305603	2.537225
C	0.228444	-3.478004	3.643376
H	0.851117	-5.254417	2.588581
H	-0.255707	-1.527233	4.438128
H	-0.207174	-4.022515	4.476309
C	-4.144033	-0.685012	1.981762
C	-3.181930	-1.384950	1.235525
C	-3.429757	-2.724199	0.893716
C	-4.619955	-3.348936	1.261022
C	-5.577173	-2.641825	1.999002
C	-5.330774	-1.313173	2.361743
H	-3.968182	0.356795	2.237360
H	-4.796395	-4.384130	0.979090
H	-6.506628	-3.124557	2.286261
H	-6.072917	-0.761791	2.932767
C	-1.964655	-0.757224	0.740683
C	-1.184854	-1.159548	-0.294508
H	-1.737240	0.189133	1.195756
C	-2.312206	-3.411750	0.177460
H	-2.656876	-4.306642	-0.345030
H	-1.518321	-3.698189	0.878978
N	-1.672818	-2.511561	-0.846177
C	-0.670166	-3.327073	-1.570992
H	0.126616	-3.622323	-0.890246
H	-0.267201	-2.745569	-2.384003
H	-2.435680	-2.252754	-1.573780
H	-1.168274	-4.213628	-1.968389
C	-4.139924	-0.860929	-2.559148
O	-4.134831	0.151245	-3.272897
O	-5.046090	-0.856798	-1.510771
H	-4.930973	-1.689156	-1.026093
O	-3.417320	-1.910438	-2.672544

$TS_{VIIIb-XIb}$

C	0.355887	-0.686956	-3.455507
C	1.561405	0.035115	-3.179834
C	1.226372	1.376228	-2.877071
C	-0.207538	1.483329	-2.946570
C	-0.741456	0.219003	-3.310096
H	0.293566	-1.690980	-3.846564
H	2.563299	-0.370841	-3.218648
H	1.917322	2.175304	-2.658657
H	-0.780155	2.387098	-2.789596
H	-1.785385	-0.012753	-3.471603
Ru	0.288307	0.026188	-1.264431
P	-0.207013	1.925085	0.137713
P	1.918345	-1.089116	0.003707
C	-1.981455	2.483217	0.066348
C	-2.868663	1.881786	-0.835105
C	-2.453468	3.537489	0.869661
C	-4.194078	2.310192	-0.935909
H	-2.529222	1.062017	-1.452002
C	-3.778306	3.965113	0.775070
H	-1.785567	4.030428	1.568854
C	-4.652693	3.350940	-0.127407
H	-4.849031	1.809844	-1.641776
H	-4.125605	4.779176	1.405359
H	-5.684738	3.683711	-0.197049
C	0.663074	3.461559	-0.475516
C	-0.025928	4.575009	-0.981326
C	2.067236	3.490208	-0.497330
C	0.670077	5.687422	-1.465838
H	-1.108345	4.587496	-1.010742
C	2.762450	4.604971	-0.963353
H	2.628939	2.622021	-0.182506
C	2.064626	5.713523	-1.450092
H	0.112209	6.534369	-1.855695
H	3.849053	4.596550	-0.958391
H	2.601854	6.581302	-1.822118
C	0.128852	2.025354	1.977507
C	1.427541	2.262941	2.456631
C	-0.880648	1.768460	2.925521
C	1.711041	2.229757	3.823401
H	2.241120	2.463480	1.775148
C	-0.596734	1.730185	4.291161
H	-1.902629	1.599625	2.610773
C	0.703353	1.955852	4.748281
H	2.728866	2.409189	4.156971
H	-1.398407	1.525689	4.995164
H	0.925765	1.923352	5.810951
C	3.308804	-0.037183	0.650803
C	3.726078	-0.000565	1.987256
C	4.027381	0.707066	-0.301019
C	4.819855	0.784155	2.367469
H	3.204249	-0.577930	2.741151
C	5.125011	1.480239	0.075959
H	3.721897	0.685249	-1.343039
C	5.519632	1.528573	1.417064
H	5.123654	0.807117	3.410309
H	5.666424	2.048805	-0.675065
H	6.369517	2.136007	1.714743
C	2.996928	-2.361268	-0.845407
C	2.672925	-2.918788	-2.086811
C	4.165288	-2.809897	-0.200249

C	3.487923	-3.890095	-2.676993
H	1.781687	-2.598716	-2.601741
C	4.977312	-3.781495	-0.783169
H	4.443668	-2.402222	0.766065
C	4.642571	-4.324904	-2.027507
H	3.214531	-4.302336	-3.644370
H	5.873076	-4.112920	-0.265318
H	5.277777	-5.078802	-2.483891
C	1.357656	-2.057155	1.480210
C	1.386648	-3.459637	1.506435
C	0.768207	-1.379994	2.559615
C	0.838856	-4.163615	2.583237
H	1.832160	-4.015028	0.688986
C	0.237255	-2.080937	3.641683
H	0.719741	-0.300418	2.556704
C	0.264572	-3.477767	3.655221
H	0.867975	-5.249778	2.582021
H	-0.208630	-1.530709	4.465235
H	-0.158707	-4.025898	4.492188
C	-4.088165	-0.705919	2.034865
C	-3.134363	-1.396326	1.265325
C	-3.402540	-2.727514	0.893042
C	-4.597188	-3.347944	1.259462
C	-5.540516	-2.650819	2.024397
C	-5.275422	-1.332817	2.414028
H	-3.904598	0.329819	2.309383
H	-4.786159	-4.374721	0.954850
H	-6.471495	-3.131016	2.310890
H	-6.006685	-0.786754	3.004323
C	-1.923615	-0.774747	0.758581
C	-1.180882	-1.196877	-0.305720
H	-1.687005	0.172756	1.204390
C	-2.291589	-3.407542	0.155806
H	-2.653512	-4.290662	-0.377427
H	-1.512073	-3.732353	0.863711
N	-1.655823	-2.499602	-0.847052
C	-0.683701	-3.313764	-1.595900
H	0.112167	-3.679734	-0.941026
H	-0.253517	-2.723773	-2.389418
H	-2.595955	-2.157487	-1.803162
H	-1.206469	-4.168557	-2.033984
C	-4.110549	-0.917428	-2.529404
O	-4.323249	-0.021707	-3.334197
O	-4.839983	-0.946035	-1.382975
H	-4.593838	-1.710179	-0.833187
O	-3.234193	-1.881612	-2.697033

XIb-H_2CO_3

C	0.349637	-0.686450	-3.459859
C	1.553933	0.038413	-3.188196
C	1.216924	1.378599	-2.883313
C	-0.217313	1.482328	-2.945488
C	-0.748985	0.216575	-3.307219
H	0.288700	-1.690060	-3.852000
H	2.556477	-0.365473	-3.231855
H	1.906826	2.179288	-2.667715
H	-0.791264	2.384666	-2.785422
H	-1.791777	-0.021074	-3.462812
Ru	0.288060	0.025066	-1.265702
P	-0.206963	1.920676	0.138228
P	1.926474	-1.085779	-0.006296
C	-1.985794	2.467466	0.078330

C	-2.871936	1.863229	-0.822652
C	-2.462489	3.515787	0.886131
C	-4.200193	2.283665	-0.919241
H	-2.524491	1.047172	-1.440882
C	-3.790502	3.935195	0.796495
H	-1.795927	4.011117	1.584851
C	-4.663084	3.318973	-0.105861
H	-4.858455	1.784066	-1.622828
H	-4.141391	4.744700	1.430602
H	-5.697397	3.645196	-0.171975
C	0.646760	3.464540	-0.481548
C	-0.054589	4.574716	-0.977627
C	2.050471	3.503670	-0.516341
C	0.628809	5.693553	-1.465283
H	-1.137268	4.579964	-0.996845
C	2.733318	4.624717	-0.985611
H	2.621451	2.638930	-0.208744
C	2.023183	5.729684	-1.462560
H	0.061162	6.537626	-1.847192
H	3.819956	4.624149	-0.990596
H	2.550717	6.602377	-1.836949
C	0.146509	2.025241	1.974551
C	1.450306	2.265492	2.438694
C	-0.851835	1.768986	2.934242
C	1.748903	2.236182	3.802289
H	2.256248	2.464496	1.747737
C	-0.552890	1.734767	4.296798
H	-1.876519	1.596501	2.630769
C	0.751750	1.963566	4.738981
H	2.770212	2.417296	4.124038
H	-1.346437	1.530533	5.010013

H	0.985873	1.934071	5.799222
C	3.319349	-0.029738	0.629738
C	3.753731	0.001038	1.961019
C	4.021847	0.724264	-0.326261
C	4.847796	0.789575	2.332194
H	3.245057	-0.584050	2.717913
C	5.119925	1.501344	0.041462
H	3.703100	0.707230	-1.364275
C	5.531274	1.543861	1.377674
H	5.164711	0.807835	3.371214
H	5.648519	2.077499	-0.712910
H	6.381343	2.154415	1.668372
C	3.000401	-2.356245	-0.863506
C	2.662192	-2.919273	-2.098632
C	4.177574	-2.799672	-0.231069
C	3.472341	-3.890548	-2.695484
H	1.763151	-2.603534	-2.602511
C	4.984889	-3.771185	-0.820707
H	4.466815	-2.388118	0.730397
C	4.636225	-4.319848	-2.058898
H	3.187901	-4.307267	-3.657757
H	5.887817	-4.098572	-0.312806
H	5.267726	-5.073726	-2.520446
C	1.380696	-2.054907	1.474568
C	1.403135	-3.457447	1.492896
C	0.809736	-1.380239	2.565482
C	0.869074	-4.164441	2.574562
H	1.832156	-4.010455	0.665039
C	0.293152	-2.084423	3.652415
H	0.765959	-0.300360	2.568859
C	0.315191	-3.481356	3.658953

H	0.892915	-5.250712	2.567374
H	-0.137735	-1.536510	4.485459
H	-0.096925	-4.031778	4.499997
C	-4.050763	-0.709365	2.078737
C	-3.100840	-1.398553	1.299893
C	-3.380441	-2.726919	0.917695
C	-4.577323	-3.343523	1.286812
C	-5.513051	-2.648158	2.061114
C	-5.238151	-1.333611	2.458805
H	-3.862493	0.323870	2.359364
H	-4.772680	-4.367124	0.975367
H	-6.445282	-3.125103	2.348816
H	-5.964098	-0.787866	3.056079
C	-1.893680	-0.781698	0.786236
C	-1.172051	-1.211351	-0.295423
H	-1.652049	0.167649	1.224166
C	-2.271157	-3.407841	0.177860
H	-2.637021	-4.290845	-0.353347
H	-1.493071	-3.738378	0.887706
N	-1.643148	-2.498977	-0.821702
C	-0.707894	-3.310060	-1.610821
H	0.094428	-3.719284	-0.986344
H	-0.275871	-2.709196	-2.395378
H	-2.704344	-2.093675	-1.895347
H	-1.256193	-4.140637	-2.065592
C	-4.230040	-0.919750	-2.501418
O	-4.604978	-0.114348	-3.333362
O	-4.819445	-0.949259	-1.286844
H	-4.449159	-1.641220	-0.707483
O	-3.270680	-1.807905	-2.730942

$TS_{VIIIb\text{-}8b}$

C	0.502476	-0.185474	-3.526912
C	1.791823	0.137783	-3.022915
C	1.786943	1.493298	-2.566890
C	0.476034	2.023958	-2.836331
C	-0.309289	0.998774	-3.419189
H	0.209020	-1.131811	-3.956402
H	2.635291	-0.537211	-2.991427
H	2.629327	2.038561	-2.168154
H	0.146734	3.032505	-2.624144
H	-1.345007	1.080509	-3.711301
Ru	0.326468	0.351901	-1.338125
P	-0.013634	2.017836	0.336260
P	1.638702	-1.226356	-0.136193
C	-1.616041	2.944869	0.202993
C	-2.443335	2.737211	-0.906085
C	-2.014437	3.882052	1.175171
C	-3.653667	3.424681	-1.039648
H	-2.155152	2.022184	-1.662888
C	-3.216335	4.575903	1.039238
H	-1.386474	4.071435	2.039859
C	-4.043214	4.343799	-0.065640
H	-4.290401	3.219627	-1.895414
H	-3.509929	5.294843	1.798995
H	-4.985105	4.876522	-0.162056
C	1.228162	3.394133	0.144488
C	0.861009	4.715676	-0.153199
C	2.596566	3.090602	0.241199
C	1.835555	5.703997	-0.326632
H	-0.182335	4.988360	-0.257856
C	3.567833	4.078468	0.084555

H	2.912146	2.070831	0.413288
C	3.190174	5.393455	-0.200686
H	1.527823	6.719622	-0.560062
H	4.617442	3.812131	0.173733
H	3.943786	6.164876	-0.331367
C	0.038559	1.685766	2.172272
C	1.257806	1.612187	2.862691
C	-1.140440	1.393605	2.884346
C	1.302010	1.236882	4.207192
H	2.189148	1.839792	2.363787
C	-1.096423	1.018004	4.227277
H	-2.106080	1.459200	2.399885
C	0.127401	0.930041	4.894476
H	2.262166	1.182785	4.712368
H	-2.022677	0.792032	4.747852
H	0.163303	0.631380	5.938099
C	3.208804	-0.551685	0.591200
C	3.473719	-0.601010	1.967366
C	4.156391	0.060215	-0.250727
C	4.639318	-0.032900	2.490484
H	2.766869	-1.068460	2.642464
C	5.327884	0.610373	0.269095
H	3.987713	0.103619	-1.320138
C	5.568556	0.575696	1.646219
H	4.818100	-0.072277	3.561416
H	6.048052	1.070881	-0.401264
H	6.475027	1.013638	2.054130
C	2.284010	-2.535070	-1.290816
C	1.342817	-3.179034	-2.110036
C	3.625946	-2.935714	-1.359989
C	1.734466	-4.187576	-2.989699

H	0.303157	-2.881294	-2.043402
C	4.019362	-3.940646	-2.250374
H	4.372788	-2.476358	-0.722798
C	3.078321	-4.566711	-3.069124
H	0.991264	-4.674111	-3.615707
H	5.064466	-4.234575	-2.296354
H	3.387479	-5.346288	-3.759651
C	1.032303	-2.300021	1.248401
C	1.489575	-3.619502	1.414403
C	0.124112	-1.781961	2.178695
C	1.024552	-4.401362	2.472852
H	2.200178	-4.046803	0.716003
C	-0.332528	-2.560385	3.244213
H	-0.241697	-0.773346	2.065895
C	0.108951	-3.875724	3.389592
H	1.378418	-5.423215	2.579608
H	-1.049037	-2.138228	3.941916
H	-0.257678	-4.489141	4.208013
C	-3.785986	-1.240381	2.236647
C	-2.981653	-1.629959	1.148833
C	-2.938009	-2.991090	0.777715
C	-3.707971	-3.925082	1.466987
C	-4.493267	-3.531751	2.557505
C	-4.526065	-2.186982	2.943585
H	-3.830126	-0.190221	2.514477
H	-3.674922	-4.971095	1.170185
H	-5.070904	-4.271336	3.104913
H	-5.135800	-1.877713	3.788447
C	-2.263161	-0.670940	0.328629
C	-1.731896	-0.976084	-0.910698
H	-2.267738	0.360366	0.648077

C	-1.964890	-3.380127	-0.311288
H	-2.310456	-4.281350	-0.824987
H	-0.998737	-3.626647	0.150990
N	-1.753918	-2.315704	-1.310099
C	-2.118567	-2.644277	-2.682436
H	-1.671206	-3.600176	-2.977511
H	-1.757845	-1.867813	-3.355780
H	-2.322030	-0.189497	-1.908334
H	-3.209198	-2.718772	-2.813944
C	-4.350865	0.439134	-2.478682
O	-5.160844	0.966570	-3.227109
O	-4.768885	0.015101	-1.254073
H	-4.009049	-0.333046	-0.747619
O	-3.088232	0.245138	-2.771388

XIIb-HCO_3^-

C	0.098608	-1.427646	-3.075395
C	0.627634	-0.116764	-3.238860
C	-0.432153	0.828180	-3.094363
C	-1.637411	0.077841	-2.863171
C	-1.317473	-1.300931	-2.846646
H	0.648556	-2.351270	-3.181217
H	1.661358	0.118122	-3.449106
H	-0.357215	1.897920	-3.213112
H	-2.626033	0.496154	-2.730733
H	-2.020548	-2.113309	-2.715278
Ru	-0.201315	-0.265665	-1.149460
P	-1.678370	1.134448	0.136098
P	1.909524	0.411225	-0.316381
C	-3.333126	0.383470	0.524981
C	-3.749534	-0.773914	-0.147001
C	-4.235126	0.992047	1.420064
C	-5.005185	-1.338429	0.092366
H	-3.114881	-1.259818	-0.874237
C	-5.490416	0.432650	1.658456
H	-3.964724	1.910680	1.929119
C	-5.876387	-0.741117	1.002378
H	-5.259013	-2.245456	-0.446959
H	-6.167874	0.917290	2.356207
H	-6.852006	-1.178286	1.196989
C	-2.190319	2.615783	-0.880666
C	-3.528272	2.897397	-1.195926
C	-1.195793	3.472954	-1.380726
C	-3.857786	4.012115	-1.974016
H	-4.324860	2.251480	-0.846585
C	-1.524672	4.594219	-2.141379
H	-0.152675	3.253763	-1.195676
C	-2.861262	4.868823	-2.443028
H	-4.900414	4.206550	-2.209912
H	-0.732135	5.242346	-2.505360
H	-3.121647	5.736325	-3.042830
C	-1.248158	1.964070	1.754831
C	-0.491523	3.146390	1.780032
C	-1.588921	1.378123	2.987411
C	-0.073713	3.709257	2.987521
H	-0.209956	3.643492	0.863195
C	-1.173535	1.940935	4.194792
H	-2.183039	0.477131	3.016190
C	-0.406678	3.107613	4.201414
H	0.517639	4.620307	2.971324
H	-1.450893	1.460454	5.128814
H	-0.077163	3.543597	5.140088

C	2.241965	2.234948	-0.485420
C	2.529194	3.061476	0.609025
C	2.199131	2.812683	-1.767726
C	2.738753	4.433017	0.430759
H	2.580224	2.647737	1.608784
C	2.420124	4.177407	-1.947115
H	1.995380	2.196113	-2.634720
C	2.681164	4.996656	-0.843752
H	2.950476	5.056574	1.294883
H	2.383945	4.600388	-2.947104
H	2.845414	6.061678	-0.980188
C	3.326479	-0.266127	-1.318847
C	3.247953	-1.574225	-1.817241
C	4.500199	0.469152	-1.553671
C	4.306037	-2.137442	-2.532047
H	2.344483	-2.145457	-1.658296
C	5.558787	-0.090830	-2.273410
H	4.598084	1.479189	-1.171180
C	5.465929	-1.394839	-2.765137
H	4.218666	-3.151420	-2.912515
H	6.457193	0.494871	-2.447018
H	6.289068	-1.827148	-3.326846
C	2.524963	0.101837	1.407411
C	3.854427	-0.253893	1.691006
C	1.643949	0.306004	2.477233
C	4.284851	-0.388781	3.012852
H	4.562330	-0.426930	0.888926
C	2.080474	0.198314	3.797976
H	0.612937	0.552397	2.281545
C	3.403525	-0.152068	4.070723
H	5.315064	-0.670333	3.213154

H	1.376158	0.370230	4.605070
H	3.744415	-0.248795	5.097742
C	-1.119499	-1.913220	2.752319
C	-0.222216	-2.264326	1.733570
C	0.981757	-2.902816	2.089827
C	1.288863	-3.135977	3.430473
C	0.412707	-2.727357	4.438509
C	-0.799969	-2.122649	4.093169
H	-2.083136	-1.498300	2.474965
H	2.221095	-3.633633	3.686504
H	0.665068	-2.896919	5.481225
H	-1.504228	-1.829979	4.867375
C	-0.590648	-2.145726	0.303967
C	0.025229	-3.119766	-0.520629
H	-1.660367	-2.104716	0.144432
C	1.911519	-3.342449	0.987602
H	2.522960	-4.190900	1.305043
H	2.597929	-2.534928	0.700923
N	1.149340	-3.744955	-0.204565
C	1.726618	-4.782027	-1.054066
H	2.709081	-4.471513	-1.425233
H	1.061069	-4.975802	-1.896356
H	-0.483241	-3.487171	-1.407898
H	1.848811	-5.703372	-0.475793
C	-2.947277	-4.139869	-1.754578
O	-3.838312	-3.330000	-2.083865
O	-3.093807	-4.682022	-0.467154
H	-2.340035	-5.282326	-0.361291
O	-1.930238	-4.519126	-2.396442

XIIb

C	0.032862	-1.816249	-2.849253
C	1.017080	-0.799791	-3.019372
C	0.357431	0.462239	-3.111122
C	-1.061135	0.216894	-3.026062
C	-1.254564	-1.173684	-2.849627
H	0.224207	-2.876344	-2.800897
H	2.085975	-0.959203	-3.068925
H	0.825291	1.419078	-3.285829
H	-1.836851	0.968638	-3.076104
H	-2.206773	-1.670136	-2.725532
Ru	-0.054205	-0.391165	-1.100725
P	-0.797687	1.638368	-0.153765
P	1.951249	-0.743284	0.109686
C	-2.629470	1.723219	0.134634
C	-3.454746	0.779879	-0.490469
C	-3.230540	2.797500	0.816965
C	-4.846701	0.907885	-0.448715
H	-3.009479	-0.063444	-1.005023
C	-4.619041	2.913086	0.877773
H	-2.614863	3.556419	1.288396
C	-5.432352	1.971421	0.238802
H	-5.467277	0.165292	-0.942500
H	-5.065831	3.746804	1.411835
H	-6.513415	2.069177	0.277281
C	-0.613861	3.059280	-1.359735
C	-1.726286	3.681074	-1.950710
C	0.664689	3.480495	-1.763449
C	-1.561486	4.700190	-2.894001
H	-2.732472	3.378070	-1.689760
C	0.830129	4.507722	-2.691958
H	1.545499	2.990697	-1.374027
C	-0.285319	5.124381	-3.263752
H	-2.440071	5.161139	-3.336711
H	1.833360	4.814809	-2.974609
H	-0.160235	5.919436	-3.993150
C	-0.104160	2.336123	1.421990
C	1.119827	3.025076	1.457483
C	-0.764732	2.099157	2.638114
C	1.653663	3.471465	2.668317
H	1.679321	3.214721	0.552490
C	-0.234080	2.547736	3.848231
H	-1.708708	1.573944	2.642380
C	0.980185	3.236533	3.869232
H	2.603117	3.998624	2.667097
H	-0.771690	2.355661	4.772517
H	1.397498	3.585508	4.809167
C	3.210371	0.610680	0.106224
C	4.077604	0.874130	1.177522
C	3.356131	1.354563	-1.074925
C	5.028701	1.893281	1.084951
H	4.020398	0.290503	2.088728
C	4.314584	2.364051	-1.172350
H	2.715610	1.145892	-1.922990
C	5.145019	2.646750	-0.085055
H	5.681607	2.093291	1.929662
H	4.404972	2.931853	-2.094040
H	5.883552	3.440259	-0.153060
C	2.937362	-2.151839	-0.606108
C	2.285999	-3.349689	-0.950175
C	4.322836	-2.063072	-0.810090
C	2.997415	-4.426872	-1.478735
H	1.216502	-3.446849	-0.803996

C	5.032922	-3.138305	-1.351972
H	4.858744	-1.156938	-0.551863
C	4.374982	-4.322092	-1.687953
H	2.472900	-5.344101	-1.731320
H	6.103940	-3.045918	-1.508562
H	4.929119	-5.156028	-2.108861
C	1.815903	-1.240352	1.893214
C	1.746330	-2.591991	2.270562
C	1.634662	-0.261389	2.885956
C	1.489713	-2.951703	3.595856
H	1.893559	-3.377107	1.538399
C	1.391554	-0.622760	4.211864
H	1.688146	0.787082	2.630267
C	1.308733	-1.969893	4.571417
H	1.437634	-4.003515	3.862283
H	1.261629	0.155685	4.957461
H	1.112344	-2.251126	5.601992
C	-2.018963	-3.858225	-1.119595
C	-2.279914	-2.874205	-0.149280
C	-3.594944	-2.754169	0.337789
C	-4.617952	-3.546561	-0.193616
C	-4.352150	-4.482708	-1.193167
C	-3.038028	-4.650125	-1.642730
H	-1.000086	-3.992705	-1.465142
H	-5.628600	-3.438933	0.194277
H	-5.155692	-5.089191	-1.600428
H	-2.808486	-5.397687	-2.397138
C	-1.203481	-2.034001	0.418194
C	-1.514358	-1.349277	1.603685
H	-0.726515	-0.891644	2.189536
C	-3.923663	-1.823768	1.483019
H	-4.466625	-2.375448	2.261081
H	-4.580618	-1.006182	1.164209
N	-2.734178	-1.236460	2.113893
C	-2.990347	-0.553429	3.376733
H	-3.613860	0.332916	3.211358
H	-2.047250	-0.262053	3.838352
H	-0.223003	-2.491822	0.436789
H	-3.522181	-1.230884	4.053433

$TS_{VIIIb\text{-}XII'b}$

C	-1.128040	-0.289688	-3.559776
C	-1.876486	-1.239935	-2.794946
C	-1.009790	-2.287723	-2.388835
C	0.295812	-1.983890	-2.908877
C	0.224370	-0.761230	-3.627602
H	-1.526098	0.561725	-4.092119
H	-2.933402	-1.183311	-2.575441
H	-1.284245	-3.166919	-1.825474
H	1.174630	-2.602989	-2.797254
H	1.039701	-0.282937	-4.154532
Ru	-0.279524	-0.349467	-1.440210
P	1.230574	-1.515898	0.040697
P	-1.925730	0.428779	0.065505
C	2.994307	-1.635282	-0.560114
C	3.368598	-1.171484	-1.828840
C	3.975752	-2.234983	0.250377
C	4.686192	-1.295197	-2.277795
H	2.634467	-0.687850	-2.461277
C	5.290063	-2.362273	-0.197627
H	3.712614	-2.608735	1.234598
C	5.650092	-1.890932	-1.463784

H	4.955498	-0.920923	-3.261366
H	6.032726	-2.828941	0.443121
H	6.675024	-1.986638	-1.810484
C	0.782497	-3.321954	0.117621
C	1.576871	-4.317904	-0.473065
C	-0.439333	-3.703738	0.695365
C	1.166351	-5.654528	-0.466098
H	2.516538	-4.065465	-0.949157
C	-0.841646	-5.038446	0.715706
H	-1.099701	-2.955729	1.108042
C	-0.038760	-6.022493	0.133256
H	1.796363	-6.406680	-0.932728
H	-1.789929	-5.299908	1.176463
H	-0.352742	-7.062273	0.140136
C	1.551275	-1.069803	1.825686
C	0.780430	-1.589850	2.876263
C	2.538306	-0.115446	2.143682
C	0.963290	-1.149335	4.189587
H	0.028261	-2.342480	2.691610
C	2.717621	0.326582	3.453996
H	3.186553	0.288293	1.377611
C	1.922725	-0.181392	4.484499
H	0.347729	-1.569073	4.980028
H	3.484234	1.066778	3.664834
H	2.058395	0.165560	5.504681
C	-2.543552	-0.843363	1.265516
C	-2.334910	-0.783895	2.647467
C	-3.281091	-1.917739	0.733369
C	-2.836141	-1.790453	3.480943
H	-1.780411	0.038215	3.084476
C	-3.792837	-2.910442	1.565961

H	-3.452516	-1.976819	-0.337571
C	-3.563411	-2.853566	2.945703
H	-2.659077	-1.734455	4.551331
H	-4.362640	-3.730872	1.139276
H	-3.955108	-3.630135	3.596272
C	-3.597180	0.926748	-0.612195
C	-3.803278	1.274110	-1.952519
C	-4.697758	0.991496	0.264665
C	-5.063175	1.668755	-2.411908
H	-2.982880	1.234738	-2.650985
C	-5.954359	1.390656	-0.188973
H	-4.574802	0.734847	1.311488
C	-6.143294	1.728817	-1.532033
H	-5.194393	1.925272	-3.459392
H	-6.786069	1.434831	0.508426
H	-7.123496	2.034062	-1.886907
C	-1.519103	1.886774	1.136393
C	-2.331646	3.027222	1.236407
C	-0.318426	1.854807	1.862711
C	-1.955486	4.096204	2.055300
H	-3.256746	3.096875	0.677115
C	0.049811	2.915059	2.691162
H	0.334113	0.998711	1.781989
C	-0.769358	4.041887	2.791093
H	-2.595131	4.972245	2.115368
H	0.981633	2.857475	3.245603
H	-0.482646	4.873463	3.428344
C	4.085308	2.852553	0.095831
C	2.737628	2.872368	-0.311649
C	2.053140	4.098279	-0.393145
C	2.710602	5.282823	-0.071623

C	4.044929	5.254344	0.349865
C	4.732596	4.038191	0.433267
H	4.612008	1.902500	0.138630
H	2.179831	6.229019	-0.138346
H	4.546864	6.181130	0.611941
H	5.769343	4.019411	0.755670
C	2.047051	1.653071	-0.664599
C	0.777230	1.537841	-1.222567
H	2.614965	0.746257	-0.556444
C	0.595692	4.075869	-0.775913
H	0.346416	4.964924	-1.361860
H	-0.041874	4.082730	0.118815
N	0.252775	2.897300	-1.596284
C	-0.995344	3.060976	-2.320353
H	-1.838131	3.156774	-1.627046
H	-1.154484	2.210596	-2.972319
H	1.187237	2.067828	-2.279727
H	-0.936448	3.970299	-2.926385

XII'b

C	-0.958185	-0.667159	-3.445401
C	-0.715566	-1.920664	-2.831872
C	0.695232	-2.055110	-2.600688
C	1.331681	-0.886670	-3.156703
C	0.319392	-0.024462	-3.642786
H	-1.923846	-0.285565	-3.744011
H	-1.472999	-2.644120	-2.565493
H	1.194585	-2.919524	-2.188431
H	2.396281	-0.695193	-3.169147
H	0.473132	0.951876	-4.085065
Ru	0.013269	-0.349193	-1.431442
P	1.958260	0.041282	-0.116684
P	-1.365672	-1.292329	0.248012
C	2.900242	1.576586	-0.571286
C	2.827546	2.079604	-1.879586
C	3.730025	2.232848	0.353668
C	3.539953	3.222455	-2.249222
H	2.202447	1.589425	-2.614650
C	4.436478	3.379937	-0.013213
H	3.825989	1.858002	1.366295
C	4.339007	3.882241	-1.313312
H	3.459871	3.599409	-3.265020
H	5.064445	3.878479	0.719980
H	4.884995	4.778333	-1.594386
C	3.220277	-1.284204	-0.393703
C	4.509476	-1.007150	-0.871755
C	2.866208	-2.618436	-0.132224
C	5.421778	-2.045128	-1.084800
H	4.811217	0.013196	-1.080021
C	3.783274	-3.650249	-0.330344
H	1.872919	-2.851521	0.228479
C	5.064278	-3.366641	-0.812849
H	6.414738	-1.814592	-1.460480
H	3.491102	-4.673140	-0.111210
H	5.777337	-4.169843	-0.975064
C	1.897654	0.195325	1.726582
C	2.423581	-0.766359	2.600944
C	1.299571	1.345867	2.267333
C	2.331881	-0.588882	3.984817
H	2.903641	-1.658686	2.217569
C	1.220176	1.527561	3.646766
H	0.913462	2.117724	1.610921

C	1.728964	0.554635	4.511595
H	2.738288	-1.348131	4.647385
H	0.756235	2.427381	4.040520
H	1.662091	0.691637	5.587241
C	-0.622726	-2.832371	0.944815
C	-0.055440	-2.850765	2.227534
C	-0.501359	-3.976580	0.134894
C	0.625760	-3.981636	2.685582
H	-0.129640	-1.982724	2.871145
C	0.170486	-5.107514	0.598321
H	-0.936265	-3.990290	-0.858472
C	0.742791	-5.111333	1.874697
H	1.066378	-3.972597	3.678341
H	0.249329	-5.983742	-0.038799
H	1.272024	-5.989508	2.232875
C	-2.995393	-1.856585	-0.436417
C	-3.661421	-0.996011	-1.323095
C	-3.619183	-3.054954	-0.060011
C	-4.915111	-1.329537	-1.835824
H	-3.188046	-0.066659	-1.617275
C	-4.871942	-3.392499	-0.580408
H	-3.134364	-3.732895	0.633778
C	-5.521592	-2.534667	-1.470131
H	-5.413142	-0.651142	-2.522573
H	-5.338910	-4.328194	-0.286146
H	-6.493734	-2.801908	-1.874346
C	-1.975917	-0.399539	1.742622
C	-2.718366	-1.069935	2.732324
C	-1.773581	0.976471	1.857625
C	-3.228916	-0.368638	3.822205
H	-2.895281	-2.138013	2.650508

C	-2.289942	1.682414	2.949358
H	-1.212200	1.499229	1.094673
C	-3.013984	1.010913	3.933101
H	-3.797264	-0.895558	4.583585
H	-2.118061	2.751729	3.021681
H	-3.413426	1.555610	4.784022
C	0.478099	4.131481	0.132946
C	-0.693729	3.534577	-0.368118
C	-1.938627	3.917469	0.181268
C	-1.988483	4.859427	1.209424
C	-0.814552	5.420327	1.722624
C	0.419455	5.053083	1.177520
H	1.435273	3.865934	-0.301971
H	-2.953096	5.147520	1.620643
H	-0.865329	6.141564	2.532977
H	1.338133	5.490208	1.558777
C	-0.681711	2.599792	-1.488585
C	-1.872844	2.337126	-2.134378
H	0.241518	2.412242	-2.016352
C	-3.205688	3.247578	-0.305413
H	-4.045050	3.950676	-0.279924
H	-3.475216	2.407155	0.356319
N	-3.073826	2.741398	-1.675974
C	-4.318393	2.407055	-2.348566
H	-4.898396	1.686167	-1.758181
H	-4.109229	1.975304	-3.329414
H	-1.885458	1.849691	-3.100986
H	-4.926031	3.308932	-2.482217

8b

C	3.000024	0.345555	0.103625
C	1.943428	1.251479	0.001306
C	0.615028	0.800779	-0.098201
C	0.369221	-0.593287	-0.096157
C	1.429179	-1.489727	0.024499
C	2.747759	-1.029016	0.122521
H	4.020282	0.713073	0.176820
H	2.139913	2.321093	0.006135
H	1.226049	-2.558491	0.030734
H	3.566825	-1.737204	0.209501
C	-1.776247	1.204464	0.059636
H	-2.637671	1.859581	0.155795
C	-0.527453	1.700363	-0.136964
C	-1.049013	-1.073414	-0.325439
H	-1.229549	-1.199922	-1.412419
H	-1.203652	-2.054861	0.135253
N	-2.034954	-0.138390	0.224790
H	-0.372940	2.772287	-0.192066
C	-3.409439	-0.607438	0.157694
H	-4.079204	0.132856	0.602431
H	-3.731697	-0.795767	-0.879631
H	-3.508607	-1.542455	0.718791

j. oordinates for Ru(II)-vinylidene oxidative amidation. 1a, XVIa to XVIIa.

9a

C	-2.423524	0.863795	-0.599532
C	-1.204820	0.844428	0.107334
C	-0.696601	-0.380854	0.604710
C	-1.422028	-1.552327	0.361726
C	-2.626267	-1.526670	-0.342574
C	-3.129926	-0.314484	-0.822801
H	-2.803191	1.811061	-0.969639
H	-1.029768	-2.495484	0.730961
H	-3.170393	-2.450773	-0.515105
H	-4.068068	-0.287282	-1.368882
C	-0.493018	2.069528	0.309198
C	0.121781	3.101037	0.477453
H	0.646039	4.020283	0.627917
C	0.626707	-0.451703	1.337294
H	0.604099	-1.261103	2.069916
H	0.828639	0.486567	1.861260
N	2.347749	0.296078	-0.227591
C	3.341130	0.140005	-1.277186
H	4.109857	-0.570895	-0.962511
H	3.807936	1.107699	-1.470202
H	1.964460	1.216165	-0.054847
C	1.759305	-0.777737	0.354145
O	2.083084	-1.938393	0.099226

H	2.895043	-0.231743	-2.208139

XVIa

C	-0.168502	-1.145509	-3.446778
C	0.998156	-1.819381	-2.971811
C	2.061572	-0.877665	-2.873916
C	1.537771	0.394845	-3.268672
C	0.170490	0.225792	-3.642822
H	-1.132103	-1.601420	-3.628202
H	1.051547	-2.875305	-2.746325
H	3.073365	-1.082545	-2.556261
H	2.092878	1.322927	-3.302150
H	-0.493539	0.998033	-4.006925
Ru	0.374543	-0.247461	-1.390969
P	1.435135	1.505011	-0.199725
P	0.410886	-1.906044	0.294682
C	0.798391	3.176717	-0.664565
C	-0.079487	3.354665	-1.741137
C	1.202453	4.303423	0.075974
C	-0.552268	4.628463	-2.070507
H	-0.409461	2.499266	-2.314250
C	0.732674	5.573114	-0.255512
H	1.881137	4.189606	0.914985
C	-0.149149	5.739029	-1.328306
H	-1.240674	4.746026	-2.902369
H	1.051508	6.432208	0.327344
H	-0.520029	6.728285	-1.580246
C	3.236323	1.571365	-0.606639
C	3.839069	2.708072	-1.166113
C	4.018843	0.424786	-0.388552
C	5.199716	2.697749	-1.486474
H	3.259660	3.603251	-1.359546
C	5.379063	0.423825	-0.695439
H	3.567068	-0.471835	0.013536
C	5.974002	1.561164	-1.248065
H	5.651319	3.584145	-1.922553
H	5.966112	-0.470322	-0.507870
H	7.031777	1.559398	-1.494574
C	1.355525	1.606205	1.639862
C	2.401114	1.167585	2.464615
C	0.202047	2.150427	2.229332
C	2.287258	1.261320	3.854169
H	3.308451	0.758325	2.038129
C	0.097452	2.250616	3.614784
H	-0.631111	2.489602	1.623866
C	1.138033	1.803130	4.432586
H	3.104790	0.915708	4.480504
H	-0.809614	2.663548	4.043974
H	1.054039	1.876728	5.513052
C	2.119868	-2.416501	0.755308
C	2.663500	-2.083365	2.005440
C	2.945303	-3.037246	-0.199674
C	4.005052	-2.352510	2.287725
H	2.052467	-1.600911	2.758101
C	4.280578	-3.315158	0.089658
H	2.547522	-3.308987	-1.170154

C	4.817201	-2.966034	1.333055
H	4.411204	-2.078510	3.257056
H	4.901803	-3.800419	-0.657386
H	5.859728	-3.174380	1.554783
C	-0.406740	-3.439061	-0.343684
C	-1.658992	-3.295886	-0.964887
C	0.122658	-4.725030	-0.165954
C	-2.362340	-4.414965	-1.408506
H	-2.089488	-2.309679	-1.100999
C	-0.580573	-5.844139	-0.622028
H	1.077861	-4.864510	0.328112
C	-1.820715	-5.693543	-1.244941
H	-3.331505	-4.284016	-1.881632
H	-0.156432	-6.834582	-0.483477
H	-2.364147	-6.565931	-1.596209
C	-0.454728	-1.745701	1.913451
C	-0.370749	-2.808256	2.835165
C	-1.235148	-0.631922	2.230590
C	-1.036954	-2.735718	4.055659
H	0.218183	-3.689598	2.601178
C	-1.896762	-0.557374	3.460930
H	-1.337336	0.183334	1.531278
C	-1.798917	-1.605123	4.374029
H	-0.960966	-3.560170	4.758834
H	-2.473492	0.334704	3.682298
H	-2.312381	-1.547256	5.329658
C	-3.986479	-0.597232	-2.174051
C	-3.779306	0.138916	-0.994130
C	-4.806139	0.190828	-0.021786
C	-6.014383	-0.468191	-0.288542
C	-6.210445	-1.195965	-1.463459
C	-5.184852	-1.271658	-2.406857
H	-3.195244	-0.619420	-2.917566
H	-6.807754	-0.426087	0.453402
H	-7.156632	-1.700274	-1.637110
H	-5.320753	-1.834286	-3.326108
C	-2.498289	0.868329	-0.819922
C	-1.293101	0.353453	-0.959008
C	-4.603272	0.805713	1.351562
H	-4.006064	0.121995	1.959166
H	-5.575784	0.902648	1.847777
N	-4.356589	3.125009	0.608766
C	-3.742126	4.442295	0.554726
H	-2.755837	4.410748	0.074387
H	-3.615816	4.836954	1.565826
H	-5.049271	2.881469	-0.086343
H	-2.519466	1.934564	-0.592491
C	-3.908063	2.162675	1.448812
O	-3.012045	2.374453	2.274615
H	-4.389062	5.111853	-0.014687

$TS_{XVIa\text{-}XVIIa}$

C	0.594576	-1.725186	-3.064259
C	1.922686	-1.962825	-2.576240
C	2.673183	-0.774330	-2.706876
C	1.813745	0.221151	-3.295594

C	0.547944	-0.371323	-3.531779
H	-0.195607	-2.457030	-3.125198
H	2.280606	-2.895615	-2.162957
H	3.706114	-0.634498	-2.423280
H	2.093172	1.231848	-3.553785
H	-0.304618	0.125150	-3.974764
Ru	0.877008	-0.360892	-1.279548
P	1.915703	1.516947	-0.189738
P	0.811707	-1.904371	0.489666
C	1.630459	3.121153	-1.081702
C	0.739712	3.205118	-2.158186
C	2.360921	4.264231	-0.716562
C	0.586606	4.402476	-2.863255
H	0.155545	2.336228	-2.435605
C	2.196523	5.464703	-1.408552
H	3.062655	4.217170	0.110868
C	1.311926	5.535668	-2.488410
H	-0.105320	4.447999	-3.699867
H	2.764872	6.340600	-1.108978
H	1.190845	6.467209	-3.033920
C	3.784246	1.507353	-0.290769
C	4.373484	1.771579	-1.542549
C	4.634558	1.255532	0.794491
C	5.757194	1.752014	-1.708019
H	3.752694	2.017187	-2.396373
C	6.023432	1.239470	0.629943
H	4.235842	1.076728	1.782935
C	6.591529	1.477958	-0.620290
H	6.182278	1.960847	-2.685739
H	6.656087	1.039158	1.490025
H	7.670249	1.463938	-0.746245
C	1.581718	1.942187	1.582924
C	1.974645	1.061003	2.601978
C	0.861838	3.094197	1.939348
C	1.684013	1.332760	3.940136
H	2.484643	0.139017	2.360286
C	0.559573	3.359383	3.275396
H	0.515593	3.778897	1.175379
C	0.974177	2.483501	4.283004
H	2.000141	0.630483	4.706049
H	-0.003225	4.253847	3.527750
H	0.738134	2.693376	5.322305
C	2.390378	-2.123679	1.432907
C	2.415412	-2.532145	2.776823
C	3.605935	-1.916567	0.764443
C	3.631891	-2.714505	3.436333
H	1.489930	-2.699152	3.316524
C	4.821854	-2.117356	1.420897
H	3.603375	-1.580270	-0.265555
C	4.838222	-2.510463	2.760243
H	3.635706	-3.019041	4.479045
H	5.752480	-1.943277	0.889394
H	5.783525	-2.653444	3.275830
C	0.539407	-3.628096	-0.147138
C	-0.655788	-3.911495	-0.827666
C	1.482354	-4.652983	0.017489
C	-0.902599	-5.187622	-1.332665
H	-1.396577	-3.134408	-0.965358

C	1.237715	-5.929766	-0.498806
H	2.409420	-4.467647	0.548055
C	0.047224	-6.201349	-1.174235
H	-1.835978	-5.382859	-1.852223
H	1.980470	-6.711272	-0.364951
H	-0.141150	-7.194475	-1.572059
C	-0.457970	-1.800129	1.824756
C	-0.984443	-2.954627	2.428770
C	-0.848392	-0.542464	2.307333
C	-1.873011	-2.850531	3.501042
H	-0.697969	-3.937786	2.071610
C	-1.721962	-0.443367	3.392812
H	-0.486675	0.355809	1.825861
C	-2.238397	-1.594872	3.990391
H	-2.274650	-3.752205	3.954398
H	-2.006262	0.536722	3.764195
H	-2.928076	-1.513331	4.825356
C	-2.739684	-1.661401	-2.963869
C	-2.924073	-1.258505	-1.633488
C	-3.788056	-2.016242	-0.802167
C	-4.406493	-3.159090	-1.325789
C	-4.187739	-3.566782	-2.643549
C	-3.352420	-2.810525	-3.466938
H	-2.117228	-1.056831	-3.611910
H	-5.059963	-3.743025	-0.682514
H	-4.672305	-4.461566	-3.023498
H	-3.184069	-3.105164	-4.498911
C	-2.281738	-0.032543	-1.089780
C	-0.967468	0.165725	-0.951104
C	-4.030389	-1.621712	0.635745
H	-3.093286	-1.341535	1.120652
H	-4.427719	-2.472166	1.199313
N	-5.701455	-0.000440	-0.155076
C	-6.679680	1.069654	-0.064593
H	-6.922135	1.232498	0.986280
H	-7.589334	0.791647	-0.604898
H	-5.557753	-0.442213	-1.052869
H	-2.992614	0.695204	-0.710717
C	-2.301529	3.688874	-0.535453
C	-3.443515	4.436866	-0.332790
C	-4.388957	4.060613	0.636674
C	-4.109962	2.910866	1.383089
C	-2.949563	2.189151	1.164693
H	-1.538262	3.922694	-1.263473
H	-3.599648	5.317536	-0.947084
H	-4.805593	2.528443	2.119622
H	-2.702150	1.283119	1.694224
C	-5.644897	4.856430	0.845803
H	-6.326027	4.354215	1.536489
H	-5.407015	5.845078	1.256078
H	-6.164169	5.019264	-0.104591
O	-0.951672	1.874873	0.007602
N	-2.063423	2.581945	0.217240
C	-4.991891	-0.456916	0.903950
O	-5.094949	0.009010	2.043579
H	-6.292147	2.004618	-0.484668

XVIIa

C	0.432293	-1.714287	-3.066203
C	1.756011	-2.008979	-2.597063
C	2.559801	-0.855441	-2.740605
C	1.736949	0.177274	-3.317414
C	0.440115	-0.357178	-3.525452
H	-0.386696	-2.412706	-3.125247
H	2.082638	-2.959076	-2.197634
H	3.606231	-0.771196	-2.486326
H	2.056557	1.174314	-3.584633
H	-0.395623	0.180445	-3.952057
Ru	0.832951	-0.373260	-1.288246
P	1.950275	1.432778	-0.204567
P	0.726373	-1.918044	0.464637
C	1.733883	3.075807	-1.058883
C	0.872116	3.218462	-2.152498
C	2.482663	4.189210	-0.641379
C	0.759513	4.442598	-2.819002
H	0.283799	2.369417	-2.480686
C	2.363741	5.414602	-1.297496
H	3.163592	4.098326	0.199833
C	1.503165	5.544155	-2.391899
H	0.088037	4.531636	-3.668834
H	2.947960	6.265499	-0.958635
H	1.416184	6.496007	-2.908091
C	3.821068	1.390220	-0.275883
C	4.418247	1.540678	-1.542111
C	4.666111	1.245421	0.832780
C	5.802631	1.507482	-1.698745
H	3.800736	1.706940	-2.416554
C	6.056132	1.214583	0.677753
H	4.262881	1.164435	1.832349
C	6.631312	1.334550	-0.586183
H	6.232531	1.626519	-2.689402
H	6.684562	1.097958	1.556262
H	7.710712	1.308591	-0.704429
C	1.594052	1.867400	1.564285
C	2.001865	1.009377	2.598924
C	0.815610	2.987601	1.900489
C	1.667758	1.273929	3.927937
H	2.557511	0.109666	2.373894
C	0.465333	3.242709	3.227729
H	0.469175	3.662987	1.127260
C	0.895003	2.390708	4.248755
H	1.997996	0.590938	4.705262
H	-0.143544	4.111656	3.461838
H	0.624257	2.592551	5.281115
C	2.301199	-2.168124	1.414571
C	2.322558	-2.578868	2.757539
C	3.519891	-1.967137	0.749616
C	3.536843	-2.764485	3.421045
H	1.395179	-2.744492	3.294576
C	4.733781	-2.169963	1.409155
H	3.518545	-1.629643	-0.280312
C	4.745701	-2.561815	2.749246
H	3.536907	-3.069642	4.463717

H	5.666386	-1.998571	0.880025
H	5.689229	-2.705392	3.268072
C	0.428486	-3.642856	-0.166126
C	-0.764099	-3.907804	-0.859224
C	1.347151	-4.686504	0.016721
C	-1.031488	-5.182715	-1.357003
H	-1.486480	-3.116147	-1.012039
C	1.083089	-5.962097	-0.493333
H	2.271828	-4.516049	0.556455
C	-0.104890	-6.214830	-1.180306
H	-1.962986	-5.362603	-1.885530
H	1.808647	-6.757267	-0.345640
H	-0.309165	-7.206909	-1.573039
C	-0.527636	-1.823803	1.821537
C	-1.104259	-2.973198	2.387465
C	-0.852584	-0.571334	2.361554
C	-1.978330	-2.868319	3.472121
H	-0.868558	-3.954754	1.991729
C	-1.711884	-0.469964	3.457572
H	-0.430686	0.322613	1.923533
C	-2.280096	-1.617632	4.014070
H	-2.418314	-3.767184	3.894627
H	-1.940423	0.507025	3.873175
H	-2.958007	-1.536473	4.858633
C	-2.801866	-1.535580	-2.950887
C	-2.956630	-1.137696	-1.615300
C	-3.813934	-1.901935	-0.778724
C	-4.433034	-3.048056	-1.292978
C	-4.229381	-3.454353	-2.613854
C	-3.414342	-2.688077	-3.447705
H	-2.202710	-0.922495	-3.612289
H	-5.076070	-3.635174	-0.641948
H	-4.713425	-4.351728	-2.988548
H	-3.262685	-2.976374	-4.484211
C	-2.352343	0.101324	-1.075063
C	-1.053354	0.413407	-0.861964
C	-4.044121	-1.504506	0.661237
H	-3.094695	-1.304399	1.159031
H	-4.518410	-2.326182	1.208743
N	-5.682413	0.164061	-0.096496
C	-6.551051	1.324740	-0.005265
H	-6.750369	1.530896	1.047290
H	-7.495279	1.123648	-0.518686
H	-5.626954	-0.327816	-0.978046
H	-3.116169	0.793225	-0.731017
C	-2.278173	3.493334	-0.783350
C	-3.340002	4.335485	-0.519179
C	-4.111662	4.165237	0.643252
C	-3.761204	3.120732	1.510388
C	-2.687641	2.299992	1.221339
H	-1.639495	3.561807	-1.651842
H	-3.568302	5.122768	-1.228747
H	-4.341823	2.905644	2.398163
H	-2.397802	1.452778	1.820665
C	-5.266964	5.071275	0.945195
H	-6.000121	4.578432	1.588334
H	-4.906786	5.963810	1.472249
H	-5.757784	5.406952	0.027805

O	-0.868307	1.711646	-0.175381
N	-1.983826	2.511911	0.093216
C	-4.903391	-0.262513	0.926406
O	-4.880250	0.288346	2.031998
H	-6.088442	2.211988	-0.454050

k. Coordinates for Ru(II)-vinylidene oxidative amidation. XVIa to 11a.

$TS_{XVIa-XVIIIa}$

C	0.186954	0.851472	-3.463318
C	-0.431672	1.958389	-2.815044
C	-1.773438	1.609598	-2.477591
C	-1.983676	0.271597	-2.933235
C	-0.790113	-0.196577	-3.545686
H	1.195204	0.825972	-3.851162
H	0.040183	2.911061	-2.622068
H	-2.501594	2.245018	-1.997141
H	-2.903988	-0.289553	-2.845436
H	-0.676025	-1.151318	-4.033544
Ru	-0.345220	0.213611	-1.332046
P	-1.951155	-0.939069	-0.003491
P	0.604617	1.735453	0.269879
C	-2.252083	-2.680776	-0.559553
C	-1.654057	-3.159993	-1.730412
C	-3.098404	-3.539448	0.166488
C	-1.888543	-4.464782	-2.173042
H	-0.998181	-2.506938	-2.289088
C	-3.335235	-4.840666	-0.275380
H	-3.575120	-3.192485	1.077576
C	-2.729140	-5.308147	-1.446022
H	-1.410665	-4.819116	-3.082137
H	-3.991950	-5.490361	0.295953
H	-2.911701	-6.323683	-1.785527
C	-3.625410	-0.148639	-0.165923
C	-4.746203	-0.833470	-0.659534
C	-3.763610	1.207973	0.173506
C	-5.974140	-0.178930	-0.796111
H	-4.674453	-1.875077	-0.948854
C	-4.993128	1.854893	0.051586
H	-2.906946	1.769292	0.521774
C	-6.104567	1.163198	-0.436734
H	-6.828448	-0.725486	-1.185520
H	-5.072609	2.901907	0.329026
H	-7.060847	1.667424	-0.541969
C	-1.814777	-1.206451	1.829332
C	-2.361256	-0.302371	2.754132
C	-1.105455	-2.319684	2.319833
C	-2.181139	-0.490397	4.126573
H	-2.926805	0.556988	2.419410
C	-0.923836	-2.502636	3.690943
H	-0.701934	-3.055640	1.633943
C	-1.455140	-1.584631	4.600442
H	-2.610588	0.224277	4.822733
H	-0.369920	-3.366607	4.046456

H	-1.313133	-1.727284	5.667655
C	-0.654885	2.855378	1.044825
C	-1.038699	2.784615	2.390863
C	-1.313003	3.776810	0.207656
C	-2.077516	3.584305	2.877955
H	-0.539794	2.109981	3.073350
C	-2.339483	4.582032	0.697564
H	-1.021636	3.874869	-0.831697
C	-2.736087	4.478932	2.034827
H	-2.364754	3.504706	3.922566
H	-2.831044	5.287074	0.033437
H	-3.543247	5.098026	2.415240
C	1.750022	2.969467	-0.507174
C	2.564869	2.572670	-1.575571
C	1.912897	4.258238	0.026233
C	3.518127	3.441686	-2.109237
H	2.453440	1.583775	-1.996116
C	2.857693	5.132065	-0.515109
H	1.309464	4.583828	0.866814
C	3.663289	4.727104	-1.582983
H	4.141745	3.110131	-2.934523
H	2.967352	6.128523	-0.096640
H	4.399357	5.408854	-1.999342
C	1.678629	1.169134	1.680017
C	3.050020	1.476377	1.717307
C	1.155265	0.365570	2.706424
C	3.865010	0.990962	2.743502
H	3.498624	2.094987	0.950115
C	1.966597	-0.109041	3.736832

H	0.111867	0.088372	2.700006
C	3.328470	0.197433	3.757501
H	4.922857	1.237645	2.743694
H	1.527529	-0.728597	4.512650
H	3.964313	-0.181021	4.552532
C	2.806485	-3.361074	1.615244
C	2.817551	-2.361632	0.622833
C	4.059582	-1.981336	0.052217
C	5.227594	-2.640628	0.453934
C	5.195205	-3.645650	1.420592
C	3.977732	-4.000081	2.009821
H	1.858672	-3.628117	2.074493
H	6.176189	-2.346229	0.012681
H	6.114573	-4.140171	1.719874
H	3.943187	-4.771499	2.773797
C	1.556225	-1.686248	0.291901
C	1.050868	-1.124463	-0.819930
H	0.908302	-1.529537	1.147055
C	4.153753	-0.821701	-0.911349
H	5.187592	-0.622644	-1.202961
H	3.787945	0.090319	-0.416545
N	2.249959	-1.831084	-2.139236
C	1.712999	-2.248618	-3.448264
H	1.538792	-1.380964	-4.073747
H	0.792534	-2.804490	-3.286104
H	2.530800	-2.665275	-1.621967
C	3.341435	-0.903587	-2.184280
O	3.504933	-0.180407	-3.145155
H	2.447991	-2.894167	-3.937085

XVIIIa

C	0.130150	0.987154	-3.472115
C	-0.449250	2.075122	-2.757142
C	-1.787835	1.738520	-2.397087
C	-2.034800	0.423472	-2.899453
C	-0.869964	-0.038756	-3.569528
H	1.118489	0.974580	-3.909272
H	0.046290	3.010893	-2.542422
H	-2.490253	2.366618	-1.871152
H	-2.962638	-0.124740	-2.807825
H	-0.793947	-0.972407	-4.104437
Ru	-0.357506	0.276040	-1.361230
P	-1.939627	-0.891786	-0.011558
P	0.670884	1.704814	0.270487
C	-2.300581	-2.614905	-0.598384
C	-1.782217	-3.058230	-1.820197
C	-3.123435	-3.486227	0.139205
C	-2.067745	-4.339465	-2.300011
H	-1.149179	-2.392153	-2.389572
C	-3.410370	-4.765003	-0.337939
H	-3.544121	-3.166629	1.087007
C	-2.881279	-5.196796	-1.558447
H	-1.651949	-4.664710	-3.249664
H	-4.047451	-5.424535	0.244370
H	-3.103544	-6.194516	-1.926030
C	-3.608874	-0.074689	-0.112285
C	-4.753945	-0.731572	-0.587841
C	-3.718801	1.275315	0.262361
C	-5.976271	-0.057536	-0.672274
H	-4.705886	-1.767126	-0.902798
C	-4.942101	1.941583	0.193575
H	-2.843864	1.816455	0.597397
C	-6.077527	1.276997	-0.277090
H	-6.849461	-0.583277	-1.048455
H	-4.998536	2.982353	0.499013
H	-7.029520	1.796174	-0.340576
C	-1.783638	-1.206196	1.816861
C	-2.293915	-0.310680	2.770365
C	-1.089136	-2.342537	2.275049
C	-2.091983	-0.528107	4.135664
H	-2.848307	0.566316	2.464122
C	-0.885637	-2.556093	3.638351
H	-0.711025	-3.072312	1.568577
C	-1.380080	-1.645299	4.575598
H	-2.493522	0.182330	4.852646
H	-0.341974	-3.437525	3.965996
H	-1.220446	-1.810912	5.637037
C	-0.546777	2.827114	1.115228
C	-0.899376	2.727665	2.467885
C	-1.204669	3.787862	0.322928
C	-1.908444	3.534894	3.003405
H	-0.398492	2.025432	3.120203
C	-2.201406	4.600164	0.860108
H	-0.934375	3.911574	-0.719000
C	-2.568476	4.466772	2.203164

H	-2.170929	3.431365	4.052501
H	-2.692613	5.334834	0.228513
H	-3.352480	5.091413	2.621152
C	1.819158	2.957863	-0.480435
C	2.582404	2.612449	-1.603262
C	2.029708	4.210479	0.118980
C	3.530182	3.495277	-2.124910
H	2.430816	1.652488	-2.076168
C	2.968897	5.098583	-0.408156
H	1.466751	4.495996	1.001280
C	3.722452	4.744208	-1.530697
H	4.112170	3.204713	-2.995008
H	3.114945	6.066251	0.063503
H	4.454045	5.436720	-1.937112
C	1.769496	1.089671	1.644688
C	3.147683	1.368559	1.656009
C	1.255425	0.277956	2.669335
C	3.977450	0.846399	2.652170
H	3.590073	1.995238	0.891505
C	2.081140	-0.233751	3.670171
H	0.206488	0.022001	2.683477
C	3.449321	0.043920	3.663589
H	5.039801	1.072382	2.631545
H	1.647945	-0.859373	4.444494
H	4.096171	-0.363249	4.435225
C	2.589344	-3.496297	1.519983
C	2.680975	-2.480836	0.547651
C	3.955063	-2.168957	0.008805
C	5.074753	-2.907399	0.411802
C	4.959791	-3.928047	1.354697
C	3.711539	-4.215181	1.918294
H	1.618217	-3.710453	1.957728
H	6.047863	-2.663708	-0.006578
H	5.839963	-4.487346	1.657684
H	3.616862	-4.998174	2.665291
C	1.479539	-1.713858	0.188707
C	1.097099	-1.183506	-0.998861
H	0.835796	-1.490722	1.025027
C	4.141794	-0.978155	-0.903533
H	5.183899	-0.857531	-1.208685
H	3.869347	-0.062748	-0.356326
N	2.087891	-1.775620	-2.106918
C	1.565853	-2.112147	-3.459161
H	1.406121	-1.207975	-4.031362
H	0.644049	-2.674250	-3.336935
H	2.398888	-2.666031	-1.699001
C	3.308604	-0.915573	-2.156947
O	3.505422	-0.212441	-3.111912
H	2.316566	-2.729213	-3.956220

11a

C	-2.600090	1.580091	-0.082127
C	-1.600924	0.756692	0.473697
C	-1.647975	-0.633533	0.226694
C	-2.635014	-1.153124	-0.619008

C	-3.598955	-0.321143	-1.188543
C	-3.586561	1.050236	-0.908495
H	-2.580493	2.646702	0.127226
H	-2.658161	-2.222849	-0.810200
H	-4.366088	-0.741289	-1.832760
H	-4.342855	1.702172	-1.336548
C	-0.539517	1.352773	1.280303
C	0.743031	0.940872	1.390476
H	-0.783282	2.273032	1.805386
C	-0.658846	-1.538058	0.922182
H	-0.904986	-2.587175	0.752926
N	1.372811	-0.148832	0.771948
C	2.789072	0.022939	0.398944
C	2.950822	0.643917	-0.991353
H	3.264216	-0.959473	0.430770
H	3.260805	0.655423	1.158221
C	4.422895	0.833212	-1.362474
H	2.456359	-0.009039	-1.719457
H	2.426039	1.607261	-1.014363
H	4.525276	1.273339	-2.359876
H	4.929979	1.496111	-0.651114
H	4.956936	-0.124325	-1.363785
H	1.430210	1.541721	1.980092
H	-0.674404	-1.339779	2.001813
C	0.756237	-1.331942	0.403399
O	1.335716	-2.173450	-0.279269

l. Coordinates for Ru(II)-vinylidene oxidative amidation. XVIa to XVIIa.

9b

C	-1.761737	1.131404	-0.052433
C	-0.380360	0.856371	-0.027903
C	0.050381	-0.491113	0.049146
C	-0.906843	-1.511779	0.111304
C	-2.271784	-1.223538	0.100374
C	-2.698412	0.103100	0.015603
H	-2.087946	2.163825	-0.126783
H	-0.577218	-2.543107	0.191808
H	-2.995699	-2.030529	0.161471
H	-3.758302	0.338969	0.002762
C	0.524261	1.961440	-0.126956
C	1.209781	2.953474	-0.238267
H	1.840968	3.811463	-0.323743
N	1.888908	-1.942280	-0.570699
H	1.288562	-2.360595	-1.266309
C	1.517521	-0.828181	0.118166
O	2.312279	-0.164201	0.781487
H	2.872206	-2.177522	-0.590409

XVIb

C	-0.121213	-0.987308	-3.548868
C	0.803821	-1.897335	-2.948763
C	2.025324	-1.215491	-2.702178
C	1.848989	0.140673	-3.133765
C	0.532800	0.274140	-3.673198
H	-1.129682	-1.221924	-3.860117
H	0.596693	-2.935276	-2.729061
H	2.918963	-1.636154	-2.264633
H	2.597568	0.920453	-3.088525
H	0.104019	1.173595	-4.094499
Ru	0.363985	-0.178328	-1.434794
P	1.692783	1.194131	-0.048465
P	-0.252772	-1.795370	0.198801
C	1.550568	2.989332	-0.463622
C	1.080554	3.412498	-1.714811
C	1.978967	3.954777	0.464244
C	1.027637	4.772515	-2.029722
H	0.743723	2.685385	-2.442659
C	1.925302	5.311891	0.147264
H	2.354691	3.649880	1.435361
C	1.447229	5.724711	-1.099555
H	0.653375	5.083838	-3.000739
H	2.256183	6.045990	0.876076
H	1.401739	6.782355	-1.342620
C	3.489895	0.848019	-0.275471
C	4.372525	1.810309	-0.788408
C	3.978853	-0.433050	0.029052
C	5.719185	1.492945	-0.987975
H	4.021015	2.805606	-1.035614
C	5.325483	-0.741325	-0.158602
H	3.307844	-1.189089	0.412987
C	6.199747	0.221055	-0.672086
H	6.391356	2.245973	-1.389195
H	5.685137	-1.734847	0.092419
H	7.247653	-0.019818	-0.825684
C	1.417780	1.221122	1.768194
C	2.375792	0.800077	2.700946
C	0.193558	1.738248	2.221695
C	2.098851	0.871537	4.068688
H	3.334496	0.415631	2.374267
C	-0.075405	1.810672	3.586932
H	-0.553905	2.092538	1.520664
C	0.873076	1.370410	4.514366
H	2.845618	0.536624	4.782890
H	-1.030808	2.205802	3.917468
H	0.660697	1.422480	5.578431
C	1.227242	-2.620171	0.918860
C	1.654291	-2.334328	2.224258
C	2.019163	-3.457447	0.111897
C	2.848187	-2.872771	2.710851
H	1.067923	-1.685255	2.862619
C	3.204539	-4.001719	0.604895
H	1.711481	-3.690268	-0.901180
C	3.625529	-3.706443	1.905716
H	3.167725	-2.633107	3.720725
H	3.801189	-4.651347	-0.028697
H	4.552756	-4.124332	2.286496
C	-1.220493	-3.160914	-0.594278

C	-2.253538	-2.802244	-1.474284
C	-1.032060	-4.514501	-0.279381
C	-3.074827	-3.777552	-2.039443
H	-2.417594	-1.757956	-1.711741
C	-1.849396	-5.490341	-0.856200
H	-0.253793	-4.816993	0.412314
C	-2.869507	-5.126695	-1.737564
H	-3.872999	-3.482196	-2.714695
H	-1.688271	-6.536026	-0.609968
H	-3.503390	-5.888421	-2.182211
C	-1.357440	-1.474137	1.641255
C	-1.526879	-2.483442	2.608689
C	-2.125366	-0.310258	1.732890
C	-2.430045	-2.310778	3.655006
H	-0.957711	-3.405348	2.545296
C	-3.035277	-0.142368	2.780419
H	-2.017902	0.481388	1.009065
C	-3.187423	-1.137174	3.744378
H	-2.546332	-3.094827	4.397777
H	-3.603087	0.780593	2.834550
H	-3.891570	-1.005590	4.561150
C	-3.970158	0.510030	-1.903321
C	-3.457038	1.484834	-1.031192
C	-4.341596	2.108680	-0.120641
C	-5.692124	1.731636	-0.096338
C	-6.177406	0.738194	-0.946866
C	-5.309801	0.127504	-1.854540
H	-3.304440	0.067056	-2.637519
H	-6.360951	2.198244	0.621009
H	-7.222379	0.447242	-0.901651
H	-5.676394	-0.637376	-2.533229
C	-2.023699	1.836352	-1.080171
C	-1.053845	0.944191	-1.177412
H	-1.716194	2.867879	-0.909489
N	-4.635689	4.167437	1.122919
H	-5.436368	4.374101	0.543936
C	-3.829623	3.104522	0.883502
O	-2.757258	2.946787	1.479011
H	-4.330056	4.863659	1.789558

$TS_{XVIb\text{-}XVIIb}$

C	-0.383503	-1.611595	-3.106265
C	0.717286	-2.403719	-2.640084
C	1.901852	-1.645616	-2.754751
C	1.550262	-0.363950	-3.310454
C	0.151770	-0.355190	-3.545183
H	-1.408983	-1.940023	-3.173565
H	0.644192	-3.410127	-2.252149
H	2.895189	-1.968994	-2.479666
H	2.235818	0.438047	-3.542131
H	-0.406592	0.467854	-3.970008
Ru	0.454332	-0.537564	-1.297686
P	2.196970	0.687993	-0.179274
P	-0.268210	-1.939066	0.445915
C	2.697576	2.240844	-1.071118

C	1.920425	2.768051	-2.110150
C	3.887188	2.898899	-0.716193
C	2.320345	3.926488	-2.780839
H	0.998498	2.273248	-2.387370
C	4.283033	4.061265	-1.379383
H	4.507158	2.505392	0.083511
C	3.500886	4.577457	-2.416102
H	1.707406	4.319088	-3.587592
H	5.203404	4.559607	-1.088472
H	3.811244	5.478870	-2.936986
C	3.855200	-0.173268	-0.200763
C	4.552022	-0.230077	-1.423531
C	4.446955	-0.765723	0.922665
C	5.781567	-0.878750	-1.522680
H	4.145128	0.252857	-2.305033
C	5.683379	-1.411904	0.824817
H	3.962393	-0.729735	1.888848
C	6.352438	-1.478959	-0.396123
H	6.297840	-0.905370	-2.478179
H	6.116880	-1.863984	1.712345
H	7.312055	-1.982405	-0.470198
C	2.039780	1.249405	1.582317
C	1.890482	0.301715	2.605497
C	2.000387	2.610544	1.925697
C	1.730974	0.695477	3.935519
H	1.878738	-0.754375	2.373325
C	1.832232	3.005462	3.253923
H	2.078857	3.370182	1.157832
C	1.700006	2.050383	4.266057
H	1.616050	-0.063034	4.704522
H	1.799486	4.064437	3.494725
H	1.568800	2.360801	5.298718
C	1.067074	-2.843699	1.361996
C	0.939387	-3.229966	2.706784
C	2.229761	-3.203460	0.663924
C	1.955674	-3.952139	3.334561
H	0.054601	-2.962095	3.272889
C	3.234764	-3.945849	1.286866
H	2.356596	-2.892500	-0.365923
C	3.103259	-4.317811	2.625693
H	1.846584	-4.232668	4.378396
H	4.128398	-4.210255	0.729811
H	3.890863	-4.882974	3.115741
C	-1.258802	-3.360216	-0.226831
C	-2.514558	-3.091343	-0.795456
C	-0.793808	-4.683122	-0.210629
C	-3.283759	-4.119861	-1.338743
H	-2.898218	-2.079115	-0.809025
C	-1.562865	-5.711750	-0.764486
H	0.165745	-4.923713	0.232876
C	-2.807730	-5.434412	-1.330482
H	-4.253609	-3.887720	-1.767863
H	-1.186135	-6.730597	-0.744319
H	-3.405274	-6.235092	-1.757048
C	-1.378291	-1.328061	1.785868
C	-2.249994	-2.204188	2.457814
C	-1.314854	0.011132	2.192339
C	-3.025178	-1.750843	3.523571

H	-2.321251	-3.242892	2.152697
C	-2.092485	0.460767	3.261908
H	-0.663349	0.699253	1.671619
C	-2.946436	-0.416730	3.929636
H	-3.696401	-2.437953	4.030435
H	-2.037162	1.501337	3.567800
H	-3.556619	-0.062700	4.755704
C	-3.360362	-0.160265	-2.792552
C	-3.393316	0.338404	-1.478377
C	-4.540054	0.052737	-0.692409
C	-5.545452	-0.777775	-1.209082
C	-5.476385	-1.285398	-2.504448
C	-4.379329	-0.960935	-3.304840
H	-2.519293	0.101339	-3.420359
H	-6.389098	-1.019114	-0.570515
H	-6.273299	-1.916396	-2.886382
H	-4.314951	-1.327687	-4.325374
C	-2.254528	1.126780	-0.953899
C	-0.973104	0.737592	-0.924403
N	-4.473744	1.813250	1.018811
H	-4.736140	2.144451	1.937524
H	-4.381241	2.504013	0.288492
H	-2.527889	2.062303	-0.488744
C	-1.131358	4.223908	-0.891377
C	-1.842144	5.384374	-0.658563
C	-2.279343	5.712029	0.635852
C	-1.955010	4.818140	1.665556
C	-1.243160	3.661785	1.400142
H	-0.768348	3.910080	-1.859588
H	-2.056068	6.038046	-1.497410
H	-2.254056	5.016115	2.689080
H	-0.941844	2.944999	2.148399
C	-3.072861	6.960985	0.890424
H	-2.628187	7.817378	0.373858
H	-4.093062	6.844872	0.504614
H	-3.136916	7.185733	1.957558
O	-0.153255	2.264129	-0.101199
N	-0.841622	3.383170	0.136206
C	-4.767788	0.513595	0.726793
O	-5.231676	-0.252210	1.571186

XVIIb

C	-1.399095	-0.858213	-2.950588
C	-0.728327	-2.092796	-2.668824
C	0.658472	-1.932920	-2.915020
C	0.858169	-0.581877	-3.366295
C	-0.399246	0.067104	-3.397757
H	-2.462203	-0.680713	-2.909002
H	-1.203370	-2.999518	-2.321328
H	1.420505	-2.692328	-2.815095
H	1.795332	-0.134850	-3.663819
H	-0.563103	1.086992	-3.707023
Ru	0.031609	-0.477875	-1.236897
P	2.214862	-0.080850	-0.374020

P	-0.864506	-1.703428	0.552573
C	3.102699	1.288753	-1.272633
C	2.431261	2.112746	-2.184512
C	4.477721	1.492593	-1.066064
C	3.115188	3.115377	-2.879993
H	1.370079	1.969446	-2.352639
C	5.159851	2.497754	-1.751916
H	5.018715	0.862443	-0.366351
C	4.480495	3.311180	-2.664047
H	2.577695	3.740772	-3.587621
H	6.222416	2.642005	-1.578057
H	5.013289	4.088789	-3.203873
C	3.471351	-1.435180	-0.674510
C	3.823773	-1.710819	-2.009464
C	4.099903	-2.178490	0.334303
C	4.738195	-2.714375	-2.324609
H	3.403427	-1.124103	-2.816344
C	5.020173	-3.183619	0.019930
H	3.892988	-1.986031	1.377821
C	5.336793	-3.464466	-1.308091
H	4.989220	-2.901932	-3.364949
H	5.486927	-3.744915	0.824580
H	6.049695	-4.247390	-1.550167
C	2.508899	0.380479	1.400948
C	2.292683	-0.561877	2.418762
C	2.846695	1.692426	1.773318
C	2.436066	-0.216240	3.763703
H	1.982876	-1.567934	2.171892
C	2.970416	2.044357	3.118411

H	3.000422	2.452562	1.016371
C	2.773265	1.089920	4.120307
H	2.263246	-0.968747	4.527680
H	3.224862	3.067454	3.381418
H	2.875552	1.364290	5.166338
C	0.211237	-3.057334	1.227101
C	0.183655	-3.458878	2.572773
C	1.055258	-3.737169	0.336558
C	0.996873	-4.504386	3.015398
H	-0.462168	-2.954292	3.282650
C	1.852684	-4.795158	0.775854
H	1.098901	-3.426351	-0.700451
C	1.831567	-5.176953	2.118836
H	0.973773	-4.794848	4.061996
H	2.505344	-5.302733	0.071960
H	2.462826	-5.990104	2.465666
C	-2.352332	-2.681368	0.021605
C	-3.415517	-1.984575	-0.571231
C	-2.470037	-4.066852	0.200427
C	-4.572022	-2.648212	-0.974870
H	-3.328540	-0.918792	-0.731613
C	-3.626045	-4.736305	-0.215857
H	-1.669533	-4.631999	0.664558
C	-4.679205	-4.031680	-0.801505
H	-5.382614	-2.083161	-1.426104
H	-3.701046	-5.810938	-0.074143
H	-5.576353	-4.555155	-1.119566
C	-1.519350	-0.928467	2.103129
C	-2.635754	-1.452533	2.776299

C	-0.855366	0.173820	2.657865
C	-3.076040	-0.884241	3.974049
H	-3.169501	-2.304274	2.370268
C	-1.283599	0.729074	3.866699
H	-0.002959	0.597371	2.145750
C	-2.399060	0.206766	4.525715
H	-3.945671	-1.298482	4.475832
H	-0.743423	1.570767	4.291564
H	-2.736996	0.644012	5.460743
C	-2.942443	2.106062	-2.991315
C	-2.983637	1.828889	-1.613338
C	-4.210944	1.362765	-1.072361
C	-5.287842	1.102608	-1.938387
C	-5.200009	1.325969	-3.307955
C	-4.020472	1.853311	-3.836687
H	-2.034097	2.533463	-3.402599
H	-6.205402	0.728413	-1.498311
H	-6.048827	1.113166	-3.951180
H	-3.938261	2.068934	-4.898332
C	-1.768155	2.155271	-0.808816
C	-0.678050	1.381474	-0.588330
N	-3.585334	1.466796	1.313611
H	-3.739981	1.118689	2.251480
H	-2.624223	1.583401	1.018907
H	-1.798907	3.168875	-0.410912
C	0.816186	4.307204	-0.038442
C	0.761270	5.613639	0.407308
C	0.059147	5.937575	1.579817
C	-0.569956	4.889418	2.271325
C	-0.493999	3.593234	1.799931
H	1.351981	3.980547	-0.918484
H	1.276485	6.378711	-0.162500
H	-1.121609	5.077011	3.185649
H	-0.959270	2.744519	2.277641
C	-0.034943	7.353850	2.060292
H	0.862774	7.920544	1.799921
H	-0.888397	7.845985	1.576877
H	-0.193124	7.399013	3.140489
O	0.355050	2.018512	0.240139
N	0.188565	3.345360	0.664810
C	-4.517460	1.107800	0.392018
O	-5.604394	0.631284	0.732741

m. Coordinates for Ru(II)-vinylidene oxidative amidation. XVIa to 11a.

XVIb’

C	-0.973995	0.367978	-3.250558
C	-0.209893	-0.838414	-3.183217
C	1.166001	-0.507338	-3.065138
C	1.257210	0.923502	-3.042916
C	-0.058972	1.460650	-3.171698
H	-2.049569	0.433358	-3.344259
H	-0.621985	-1.837449	-3.217476
H	1.990496	-1.201915	-2.998391

H	2.171856	1.496570	-2.971309
H	-0.317231	2.509938	-3.201364
Ru	0.061858	0.334645	-1.180140
P	1.937786	0.907324	0.143623
P	-0.581341	-1.633046	0.016472
C	2.100881	2.732488	0.379165
C	1.613059	3.625448	-0.587269
C	2.773190	3.244541	1.501069
C	1.787481	5.001844	-0.432370
H	1.087629	3.250912	-1.456735
C	2.941108	4.621523	1.655819
H	3.167867	2.573017	2.255951
C	2.448456	5.503419	0.691021
H	1.400798	5.680469	-1.187305
H	3.457664	5.002988	2.531759
H	2.578443	6.574645	0.814503
C	3.534354	0.433268	-0.643320
C	4.498953	1.388127	-0.996642
C	3.781874	-0.923143	-0.910889
C	5.689370	0.988227	-1.610829
H	4.331772	2.440623	-0.796150
C	4.975691	-1.318290	-1.512088
H	3.045457	-1.669648	-0.646688
C	5.931641	-0.362084	-1.868233
H	6.427634	1.736924	-1.882967
H	5.153838	-2.372497	-1.703056
H	6.859347	-0.668784	-2.342561
C	2.055199	0.284654	1.864911
C	3.211930	-0.315898	2.381946
C	0.937640	0.458240	2.694369
C	3.232760	-0.764620	3.704460
H	4.090348	-0.447004	1.760553
C	0.959766	0.003816	4.011964
H	0.047928	0.939585	2.306116
C	2.105978	-0.616431	4.517143
H	4.129457	-1.236310	4.095672
H	0.077480	0.120935	4.633858
H	2.122209	-0.978530	5.540987
C	0.818844	-2.821896	0.088766
C	1.604417	-2.959402	1.243859
C	1.197195	-3.509918	-1.078598
C	2.737395	-3.776053	1.233933
H	1.346120	-2.426793	2.150408
C	2.325681	-4.329781	-1.081710
H	0.611224	-3.409401	-1.985811
C	3.100018	-4.464523	0.075219
H	3.336740	-3.864291	2.135028
H	2.601056	-4.860108	-1.988553
H	3.981231	-5.099235	0.070319
C	-1.940341	-2.528155	-0.862626
C	-2.987383	-1.765793	-1.400435
C	-2.021097	-3.928320	-0.901100
C	-4.088225	-2.389804	-1.987126
H	-2.954390	-0.684194	-1.372843
C	-3.120825	-4.549479	-1.497894
H	-1.234823	-4.538802	-0.470084
C	-4.154561	-3.784186	-2.044272
H	-4.883169	-1.775258	-2.398591

H	-3.169141	-5.634285	-1.530507
H	-5.007199	-4.272816	-2.507148
C	-1.338777	-1.639985	1.701594
C	-1.293868	-2.784235	2.515461
C	-2.120292	-0.551540	2.107108
C	-1.972053	-2.807017	3.734753
H	-0.737919	-3.662036	2.206394
C	-2.806660	-0.579585	3.321647
H	-2.189514	0.325674	1.482246
C	-2.724323	-1.703454	4.145856
H	-1.918087	-3.694188	4.359128
H	-3.403417	0.279198	3.613759
H	-3.252254	-1.725958	5.094818
C	-3.458700	2.807805	1.939290
C	-3.034136	2.530329	0.627381
C	-4.004061	2.159353	-0.331200
C	-5.338366	1.983034	0.062556
C	-5.739403	2.241067	1.370837
C	-4.796345	2.677831	2.306704
H	-2.717050	3.094927	2.679461
H	-6.060136	1.665019	-0.683172
H	-6.779407	2.115182	1.655950
H	-5.098433	2.897089	3.326783
C	-1.579412	2.541771	0.343167
C	-0.931078	1.547704	-0.245181
H	-0.971331	3.354541	0.744608
N	-3.012318	2.977870	-2.392940
H	-2.769637	2.884641	-3.369796
H	-2.564978	3.704984	-1.854610
C	-3.722284	1.987676	-1.801067
O	-4.190670	1.040612	-2.444417

$TS_{XVIb\text{-}XVIIIb}$

C	-0.112871	-0.865250	-3.573449
C	0.567838	-1.955729	-2.944940
C	1.866419	-1.528710	-2.563411
C	1.994199	-0.154240	-2.959039
C	0.782480	0.250254	-3.586954
H	-1.096885	-0.893975	-4.019977
H	0.158909	-2.943617	-2.786810
H	2.624796	-2.125394	-2.077566
H	2.876733	0.457093	-2.827677
H	0.577103	1.222495	-4.015117
Ru	0.326060	-0.243585	-1.401467
P	1.887027	0.774439	0.083171
P	-0.833641	-1.720918	0.055329
C	2.454680	2.466206	-0.434654
C	2.129913	3.008336	-1.684560
C	3.281013	3.211097	0.427668
C	2.608264	4.265575	-2.064985
H	1.483192	2.462818	-2.357932
C	3.761258	4.463104	0.047119
H	3.554767	2.810813	1.398598
C	3.424387	4.995741	-1.201203
H	2.336271	4.671123	-3.035275

H	4.398213	5.022590	0.726208
H	3.795705	5.973088	-1.495233
C	3.491449	-0.156868	0.089203
C	4.665326	0.376748	-0.467198
C	3.520017	-1.472713	0.576433
C	5.836668	-0.384268	-0.514643
H	4.678668	1.381983	-0.870751
C	4.692741	-2.226098	0.541183
H	2.620043	-1.923744	0.966882
C	5.858057	-1.683892	-0.006253
H	6.733362	0.046101	-0.951596
H	4.684966	-3.239528	0.932067
H	6.771627	-2.270515	-0.041395
C	1.495295	1.101994	1.864957
C	1.788477	0.186666	2.884970
C	0.808515	2.285204	2.200959
C	1.385150	0.433491	4.200059
H	2.332630	-0.723381	2.675165
C	0.407630	2.528507	3.513766
H	0.583690	3.022112	1.440276
C	0.687427	1.598430	4.518720
H	1.621601	-0.291303	4.973575
H	-0.122755	3.446678	3.749048
H	0.371635	1.785435	5.540946
C	0.236879	-2.784982	1.111610
C	0.267519	-2.677272	2.506604
C	1.058908	-3.733178	0.475407
C	1.122245	-3.493755	3.254475
H	-0.364844	-1.956436	3.013119

C	1.894265	-4.558780	1.224833
H	1.044033	-3.821438	-0.607076
C	1.934205	-4.433796	2.618397
H	1.145363	-3.395491	4.335966
H	2.519812	-5.291429	0.723325
H	2.592211	-5.070080	3.202966
C	-1.832060	-3.035806	-0.813973
C	-2.473817	-2.768743	-2.031860
C	-2.036522	-4.294252	-0.220334
C	-3.291615	-3.720048	-2.643880
H	-2.318203	-1.820304	-2.527991
C	-2.850120	-5.249444	-0.832894
H	-1.570489	-4.534937	0.728193
C	-3.480750	-4.967127	-2.046571
H	-3.772084	-3.486221	-3.589515
H	-2.991737	-6.214727	-0.355331
H	-4.111935	-5.712155	-2.521688
C	-2.094689	-1.027694	1.216977
C	-3.279625	-1.708234	1.542558
C	-1.854826	0.223299	1.801947
C	-4.196977	-1.142254	2.429169
H	-3.502524	-2.673873	1.104416
C	-2.769746	0.785037	2.692522
H	-0.954917	0.764723	1.559648
C	-3.947015	0.104384	3.006255
H	-5.112754	-1.678082	2.662796
H	-2.557619	1.755698	3.130892
H	-4.667729	0.543207	3.690537
C	-2.084952	4.690017	-0.218924

C	-2.266831	3.325305	-0.523453
C	-3.585829	2.809763	-0.444400
C	-4.656236	3.626346	-0.053512
C	-4.452380	4.971455	0.231394
C	-3.157183	5.499383	0.143662
H	-1.082550	5.107862	-0.258547
H	-5.645292	3.183818	0.012075
H	-5.285957	5.603550	0.520925
H	-2.982699	6.547817	0.369098
C	-1.083501	2.514797	-0.792295
C	-0.898402	1.217711	-1.087629
H	-0.144308	3.041969	-0.644145
N	-2.827176	0.766555	-1.504770
H	-2.996381	-0.233325	-1.573389
H	-2.767401	1.174001	-2.439042
C	-3.847083	1.388698	-0.716056
O	-4.802831	0.749694	-0.31286

XVIIIb

C	-0.350139	-0.790190	-3.517718
C	0.415501	-1.886344	-3.000932

C	1.741310	-1.444817	-2.749857
C	1.798126	-0.052600	-3.096321
C	0.521735	0.345527	-3.581636
H	-1.366785	-0.830980	-3.883492
H	0.046383	-2.889863	-2.841116
H	2.560143	-2.043027	-2.378481
H	2.673710	0.578807	-3.026176
H	0.260977	1.328610	-3.951547
Ru	0.286775	-0.254249	-1.403557
P	1.924786	0.741139	-0.001885
P	-0.824091	-1.708387	0.099909
C	2.350157	2.506723	-0.408409
C	1.899969	3.091538	-1.599776
C	3.185744	3.261094	0.435652
C	2.263556	4.397266	-1.938784
H	1.243583	2.532988	-2.254125
C	3.547962	4.565278	0.098991
H	3.560590	2.828888	1.357433
C	3.086298	5.138414	-1.089564
H	1.897112	4.833740	-2.863690
H	4.192222	5.132310	0.764846
H	3.366486	6.155160	-1.349714
C	3.582475	-0.073652	-0.247791
C	4.702763	0.615557	-0.738219
C	3.707050	-1.449174	0.005023
C	5.912967	-0.052512	-0.949976
H	4.646345	1.673053	-0.965068
C	4.918396	-2.111794	-0.188308
H	2.848926	-2.013034	0.342485

C	6.029394	-1.414294	-0.669735
H	6.765476	0.500579	-1.334211
H	4.987593	-3.174211	0.028497
H	6.972732	-1.928318	-0.830285
C	1.762055	0.837831	1.850262
C	2.163003	-0.221809	2.677359
C	1.148806	1.954183	2.451994
C	1.954521	-0.170645	4.057866
H	2.636308	-1.101211	2.260824
C	0.937677	2.001748	3.829680
H	0.833983	2.798989	1.852067
C	1.337423	0.936080	4.640017
H	2.270624	-1.008149	4.672355
H	0.462246	2.875075	4.267170
H	1.172293	0.971102	5.712936
C	0.193716	-2.714496	1.264848
C	0.015400	-2.708835	2.653048
C	1.156130	-3.571285	0.702928
C	0.802038	-3.529142	3.467622
H	-0.730002	-2.066582	3.108043
C	1.935029	-4.393425	1.515914
H	1.290993	-3.598458	-0.375161
C	1.764441	-4.367856	2.904341
H	0.657760	-3.509052	4.543943
H	2.673870	-5.051395	1.067455
H	2.374124	-5.003690	3.539688
C	-1.818658	-3.069525	-0.699336
C	-2.528538	-2.829073	-1.885664
C	-1.980063	-4.316805	-0.072751
C	-3.362615	-3.800593	-2.440657
H	-2.428148	-1.878582	-2.394964
C	-2.807772	-5.293931	-0.630272
H	-1.469285	-4.532764	0.858386
C	-3.500547	-5.041513	-1.815899
H	-3.898068	-3.586822	-3.361279
H	-2.913287	-6.252452	-0.130234
H	-4.143771	-5.803003	-2.247187
C	-2.127505	-0.938463	1.169455
C	-3.474969	-1.332735	1.140414
C	-1.757919	0.141470	1.988345
C	-4.427585	-0.649615	1.902589
H	-3.795030	-2.163100	0.522213
C	-2.706339	0.810535	2.759629
H	-0.726412	0.461488	2.018465
C	-4.048466	0.422311	2.711782
H	-5.467497	-0.959947	1.858873
H	-2.395718	1.642747	3.384823
H	-4.792402	0.953258	3.298683
C	-2.112042	4.489718	0.520015
C	-2.262168	3.244016	-0.122028
C	-3.583968	2.814923	-0.410992
C	-4.703841	3.588670	-0.051073
C	-4.524899	4.811142	0.575603
C	-3.220510	5.256824	0.857106
H	-1.112111	4.841739	0.757020
H	-5.695631	3.212663	-0.280763
H	-5.381323	5.419523	0.847111
H	-3.076445	6.213494	1.351243

C	-1.106569	2.411482	-0.389352
C	-1.103414	1.191996	-0.982673
H	-0.169377	2.798180	-0.019627
N	-2.504925	0.869466	-1.531716
H	-2.679603	-0.135758	-1.475355
H	-2.459590	1.069642	-2.540872
C	-3.788647	1.535496	-1.041744
O	-4.813359	0.931198	-1.258644

11b

C	-2.732044	-0.374040	-0.000012
C	-1.719889	-1.321380	0.000002
C	-0.363422	-0.925451	0.000022
C	-0.064851	0.464345	0.000036
C	-1.103186	1.411947	0.000002
C	-2.427236	1.000475	-0.000016
H	-3.769955	-0.695309	-0.000032
H	-1.958720	-2.381445	-0.000021
H	-0.839361	2.464611	0.000011
H	-3.228046	1.733909	-0.000024
C	1.996800	-1.429883	-0.000030
H	2.860131	-2.084900	-0.000123
C	0.717495	-1.877500	0.000001
N	2.276741	-0.081418	0.000003
H	0.509835	-2.941263	-0.000092
C	1.330764	0.934609	0.000156

1

O	1.679872	2.118932	-0.000096
H	3.243373	0.224133	0.000061
